# Synthesis and immunological evaluation of the lipopolysaccharide outer core of *Salmonella* for potential broad-spectrum protection against multiple *Salmonella* serovars†

**DOI:** 10.1039/d5sc03944d

**Published:** 2025-07-10

**Authors:** Xingling Pan, Ting-An Chen, Fatemeh Shafieichaharberoud, Sharon M. Tennant, Scott M. Baliban, Xuefei Huang

**Affiliations:** a Department of Chemistry, Michigan State University 578 South Shaw Lane East Lansing MI 48824 USA huangxu2@msu.edu; b Institute for Quantitative Health Science & Engineering, Michigan State University 578 South Shaw Lane East Lansing MI 48824 USA; c Center for Vaccine Development and Global Health, University of Maryland School of Medicine, Baltimore 685 W. Baltimore St. Baltimore MD 21201 USA; d Department of Biomedical Engineering, Michigan State University East Lansing MI 48824 USA

## Abstract

*Salmonella* species (*Salmonella* spp.) are a major foodborne pathogen and a leading cause of gastroenteritis in humans. The emergence and continued spread of multidrug-resistant *Salmonella* spp. suggest the urgent need for novel vaccines to complement antibiotics. Currently, there are no approved vaccines against *Salmonella* spp. for human use beyond those for *Salmonella enterica* (*S. enterica*) serovar Typhi. To address this gap, oligosaccharides corresponding to the lipopolysaccharide (LPS) outer core of *S. enterica* were investigated as potential antigens that can target multiple pathogenic *Salmonella* serovars. An efficient chemical synthesis strategy was developed to stereoselectively construct the sterically congested outer core pentasaccharides with five 1,2-*cis* glycosidic linkages. These synthetic glycan antigens were conjugated to the mutant bacteriophage Qβ, a highly immunogenic carrier system. The resulting constructs elicited strong and durable anti-glycan IgG antibodies, which can robustly bind to *Salmonella* spp. with truncated LPS (rough strain). For smooth strains bearing long LPS chains, the combination of the antisera with the antimicrobial peptide thanatin, an inhibitor of the LPS transport system, exhibited enhanced cytotoxicity toward multiple serovars of pathogenic *Salmonella*, suggesting that combination therapy may serve as a strategy for enhancing protection against salmonellosis.

## Abstract 2

Salmonella lipopolysaccharide outer core glycans were synthesized. Antisera generated by the bacteriophage Qβ-outer core glycan conjugate in combination with thanatin, exhibited enhanced cytotoxicity toward multiple serovars of pathogenic *Salmonella*.

## Graphical abstract

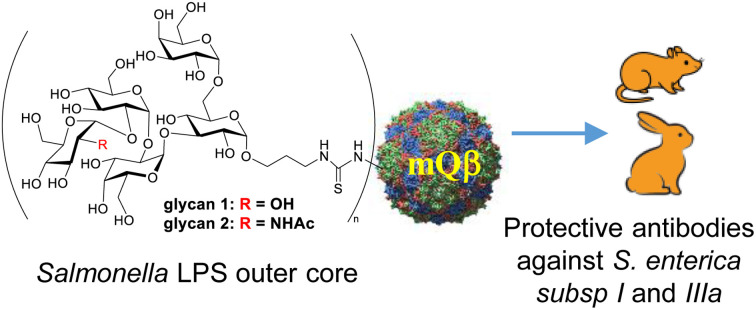

## Introduction

*Salmonella* is a genus of Gram-negative, facultatively intracellular anaerobes responsible for a range of diseases from localized infections and gastroenteritis to invasive disease including bacteremia and enteric fever. There are multiple types of *Salmonella* species (*Salmonella* spp.) that are pathogenic in humans, including *Salmonella enterica* serovar Typhi (*S.* Typhi), *Salmonella* Paratyphi A, B and C (*S.* Paratyphi A, B and C), and non-typhoidal *Salmonella* (NTS) such as *Salmonella* Enteritidis (*S.* Enteritidis) and *Salmonella* Typhimurium (*S.* Typhimurium). Healthy individuals typically contract *Salmonella* spp. through ingestion of contaminated food or water.^1^ Globally, *Salmonella* spp. are among the most common foodborne bacterial pathogens, causing an estimated 200 million to 1 billion infections annually, including 93 million cases of gastroenteritis and 155 000 deaths.^2–4^ In the United States, *Salmonella*-related foodborne illnesses were estimated to cost $17.1 billion in 2023, making it the most costly among the 31 major foodborne pathogens.^5^

Antibiotics are the main strategy to treat severe *Salmonella* infections. However, drug resistant *Salmonella* strains have become prevalent worldwide.^6–8^ For example, analysis of 16 *S.* Typhimurium strains isolated from Nepal showed all isolates were resistant to ampicillin, nalidixic acid, tetracycline, co-trimoxazole and chloramphenicol.^9^ In the US, out of 246 *S.* Paratyphi A isolates, 94% were resistant to nalidixic acid or less susceptible to ciprofloxacin.^10^ The Centers for Disease Control and Prevention (CDC) has classified drug resistant *Salmonella* including typhoidal and NTS strains as a serious threat, requiring “prompt and sustained action to ensure the problem does not grow”.^11^ Vaccines can be an attractive approach to prevent *Salmonella* infections. To date, the only licensed *Salmonella* vaccines in humans target *S.* Typhi.^12–17^ No vaccines are yet available against the other major, disease-causing serovars including *S.* Paratyphi A, *S.* Typhimurium, and *S.* Enteritidis.

Surface polysaccharides are suitable antigens for *Salmonella* vaccine development as exemplified by typhoid conjugate vaccines, which utilize the Vi capsular polysaccharide as the sole vaccine antigen with high success rates in preventing typhoid fever in children after a single dose.^14–16^ However, one major drawback in targeting such polysaccharides is that the polysaccharides are often serovar-specific. For example, Vi capsular polysaccharide-based conjugate vaccines are ineffective against Vi-negative *S.* Typhi strains as well as *S.* Paratyphi A, *S.* Typhimurium, or *S.* Enteritidis, since these *Salmonella* serovars do not express Vi. Similarly, the efficacy of a surface carbohydrate based vaccine derived from *S.* Enteritidis would be expected to have limited efficacy against serovars such as *S.* Typhimurium or *S.* Paratyphi A, which express different O-antigen structures.^18–22^ Therefore, in order to develop effective vaccines against the major pathogenic serovars of *Salmonella*, a combination of multiple polysaccharide vaccines would be needed, which presents significant hurdles for vaccine development. It would be highly desirable if a common antigen can be established that can enable the targeting of multiple serogroups of *Salmonella*.

**Fig. 1:**
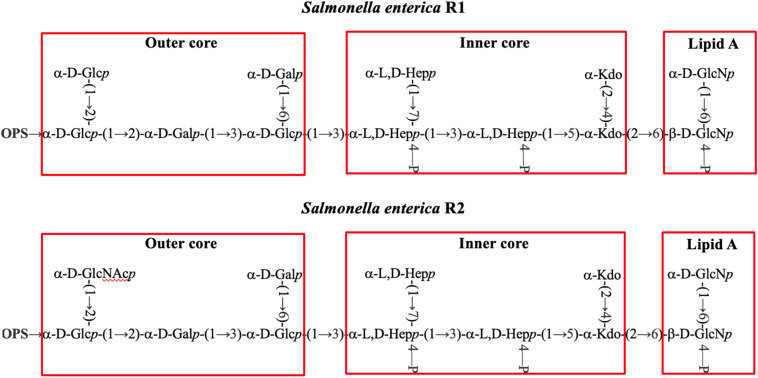
Chemical structures of *Salmonella* LPS including *S. enterica* subsp. IIIa (R1) and *S. enterica* subsp. I (R2).

As a Gram-negative bacterium, *Salmonella* abundantly expresses lipopolysaccharide (LPS) on the cell surface. Vaccines targeting LPS have demonstrated strong pre-clinical efficacy against various microbial infections, as host recognition of LPS is crucial for clearing Gram-negative bacterial infections.^23–27^ The structure of *Salmonella* LPS includes a lipid A moiety (endotoxin), which is embedded in the outer membrane, a conserved core oligosaccharide that bridges lipid A to an extended and highly variable O-polysaccharide (OPS) (Fig. 1 and S1†). Previously, we synthesized OPS-like antigens from *S.* Paratyphi A and *S.* Enteritidis, which were then conjugated with bacteriophage Qβ to create conjugate vaccines.^21,22^ The resulting immune sera provided substantial protection to mice against lethal challenge with the respective *Salmonella* strain, showcasing the potential efficacy of this synthetic glycan-based vaccine approach. However, the OPS chains exhibit significant variations in both structure and composition, contributing to the extensive diversity of serotypes.^28,29^ This variability makes anti-OPS vaccines inherently strain-specific and unable to provide protection against multiple *Salmonella* serotypes bearing different OPS structures.

The limitation associated with targeting OPS prompted us to explore the LPS core region as a potential target for vaccine development. The *Salmonella* LPS core displays two chemotypes (R1 and R2) (Fig. 1 and S1†)^28,30^ with an outer region and an inner region linked to lipid A. The inner core and lipid A structures are well-conserved across different species within the Enterobacteriaceae family, including *Escherichia coli* (*E. coli*) and *Salmonella* spp.^31^ Immunization with a lipo-oligosaccharide containing the inner core and lipid A^32^ may cause cross-reactivities with commensal bacteria such as *E. coli*. In comparison, the outer core sequence is unique to each species. To establish a vaccine that can potentially target multiple types of *Salmonella* without affecting commensal bacteria, we decided to focus our investigation on the unique outer core structure of *Salmonella*. Herein, we report the chemical synthesis and immunological evaluation of the outer core domain of *Salmonella* LPS to target multiple types of pathogenic *Salmonella*.

## Results and discussion

### Stereoselective synthesis of *Salmonella* LPS outer core-like pentasaccharides 1 and 2

**Scheme 1:**
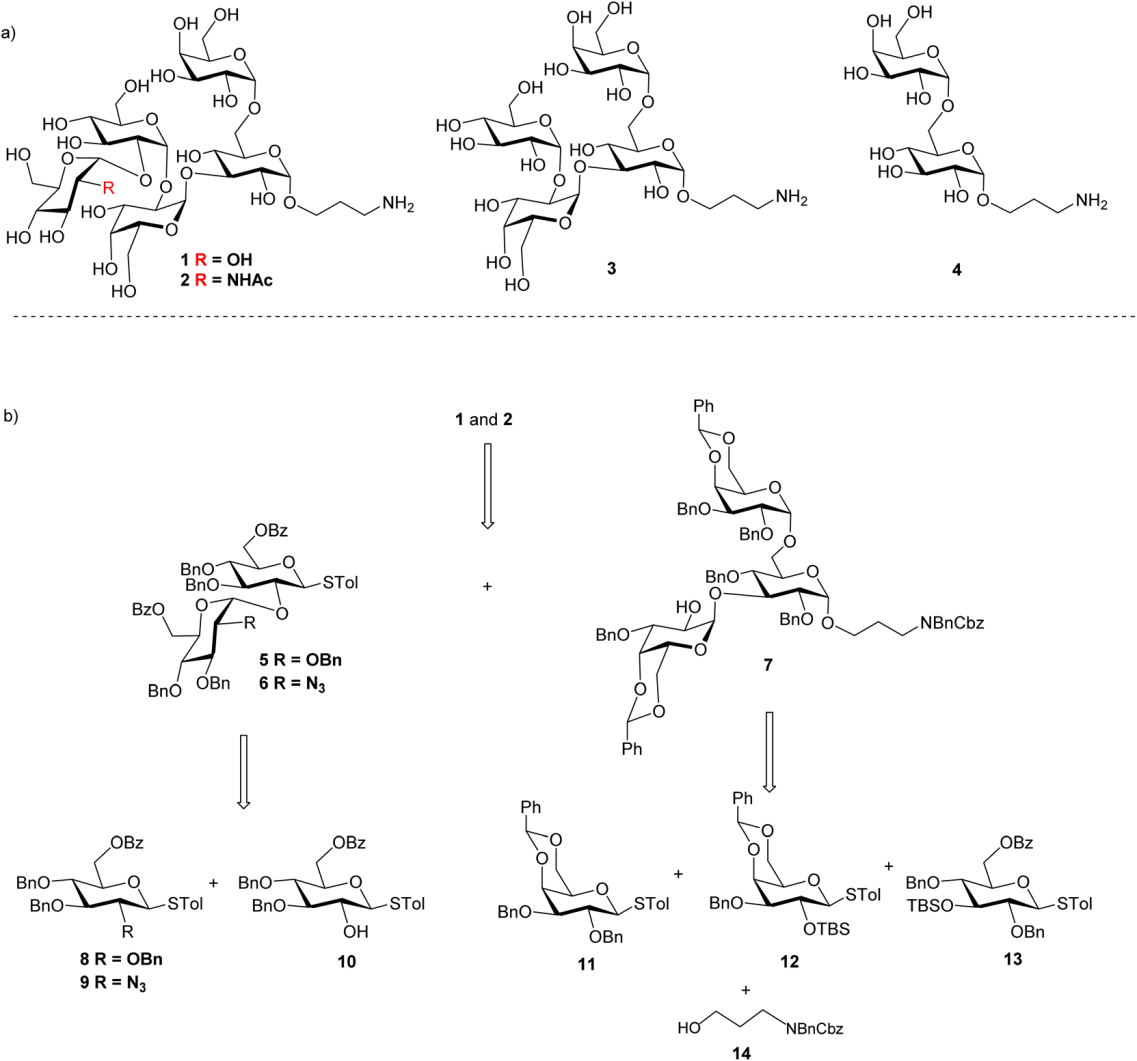
(a) Structures of *Salmonella* outer core-like glycan targets 1–4; and (b) retrosynthetic analysis of pentasaccharides 1 and 2.

As it is difficult to purify the LPS outer core from bacteria, chemical synthesis is critical to prepare the LPS glycans for bioconjugation. We focused on the outer core pentasaccharides 1 and 2 from chemotypes R1 and R2 respectively, as well as their structural fragments, *i.e.*, tetrasaccharide 3 and disaccharide 4. The pentasaccharides 1 and 2 are challenging targets as they contain five 1,2-*cis* linkages (Scheme 1a). Furthermore, the non-reducing end trisaccharides are connected *via* two consecutive α1-2 linkages, posing severe steric hindrance to glycosidic bond formation. Our initial strategy for syntheses of pentasaccharides 1 and 2 was based on a [2 + 3] convergent coupling reaction between disaccharide donor 5 or 6 and trisaccharide acceptor 7, which in turn could be derived from building blocks 8–14 (Scheme 1b). The aminopropyl linker at the reducing end of 1–4 was designed to conjugate the final antigen to a protein to enable immunological studies and analysis.

**Scheme 2:**
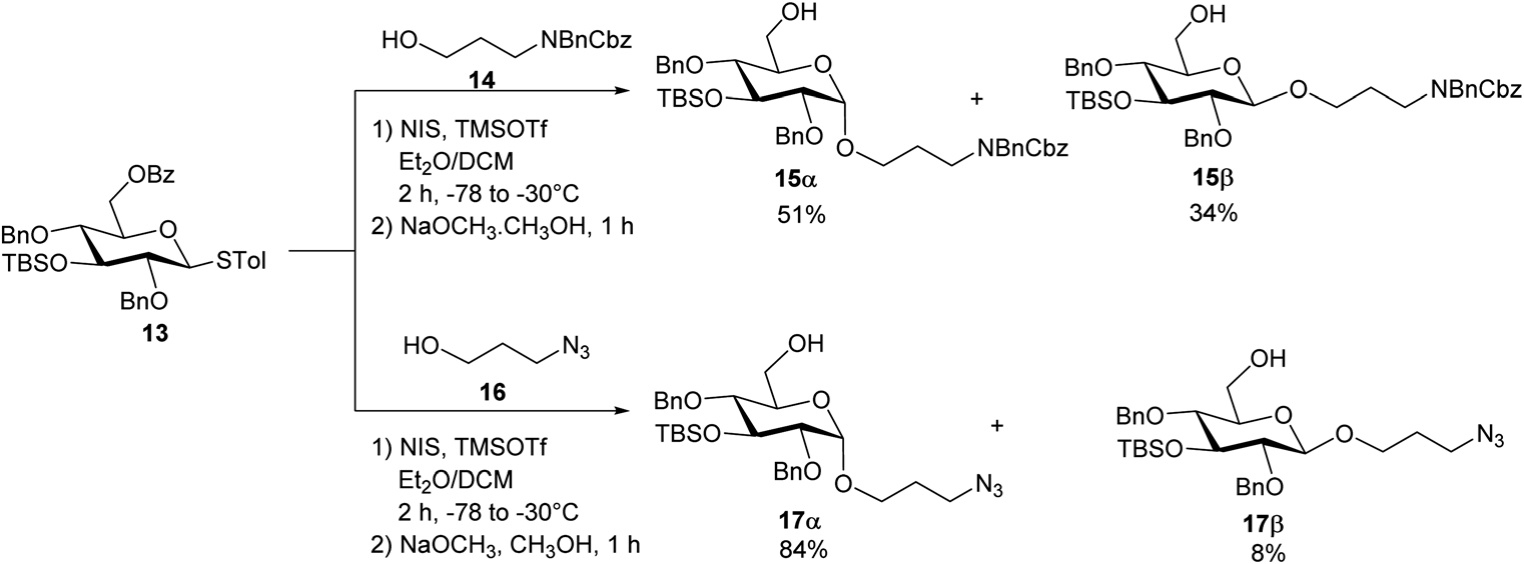
Syntheses of the non-reducing end monosaccharide acceptors 15α and 17α.

To construct the α-glycosidic bond at the reducing end of the target pentasaccharides, we started with the thioglycoside donor 13,^33^ which contains a 6-*O* Bz (benzoyl) group potentially helping to enhance the α-selectivity.^34^ Thioglycoside 13 was treated with 3-amino-1-propanol derivative 14 in the presence of *N*-iodosuccinimide (NIS) and triflic acid (TfOH) as the promoter and diethyl ether (Et_2_O)/dichloromethane (DCM) as the solvent, which resulted in an inseparable anomeric mixture of the products (Scheme 2). Removal of the Bz moiety from the products enabled the isolation of glucosides 15α and 15β in 51% and 34% yields respectively. Interestingly, when the acceptor was changed to 3-azidopropan-1-ol 16 for the glycosylation reaction by donor 13 followed by subsequent treatment with NaOCH_3_, compound 17α was produced in 84% yield, along with the β-anomer 17β as a minor product (8%) (Scheme 2). In compound 17α, the α-configuration of the glucopyranosidic linkage was established by its H-1/H-2 coupling constant (^3^*J*_H-1/H-2_ = 3.7 Hz). The reason for higher α-stereoselectivity achieved with 3-azidopropan-1-ol 16 is not clear, which may be due to its better matching with the 1,2-*cis* reaction trajectory toward the activated donor.

**Scheme 3:**
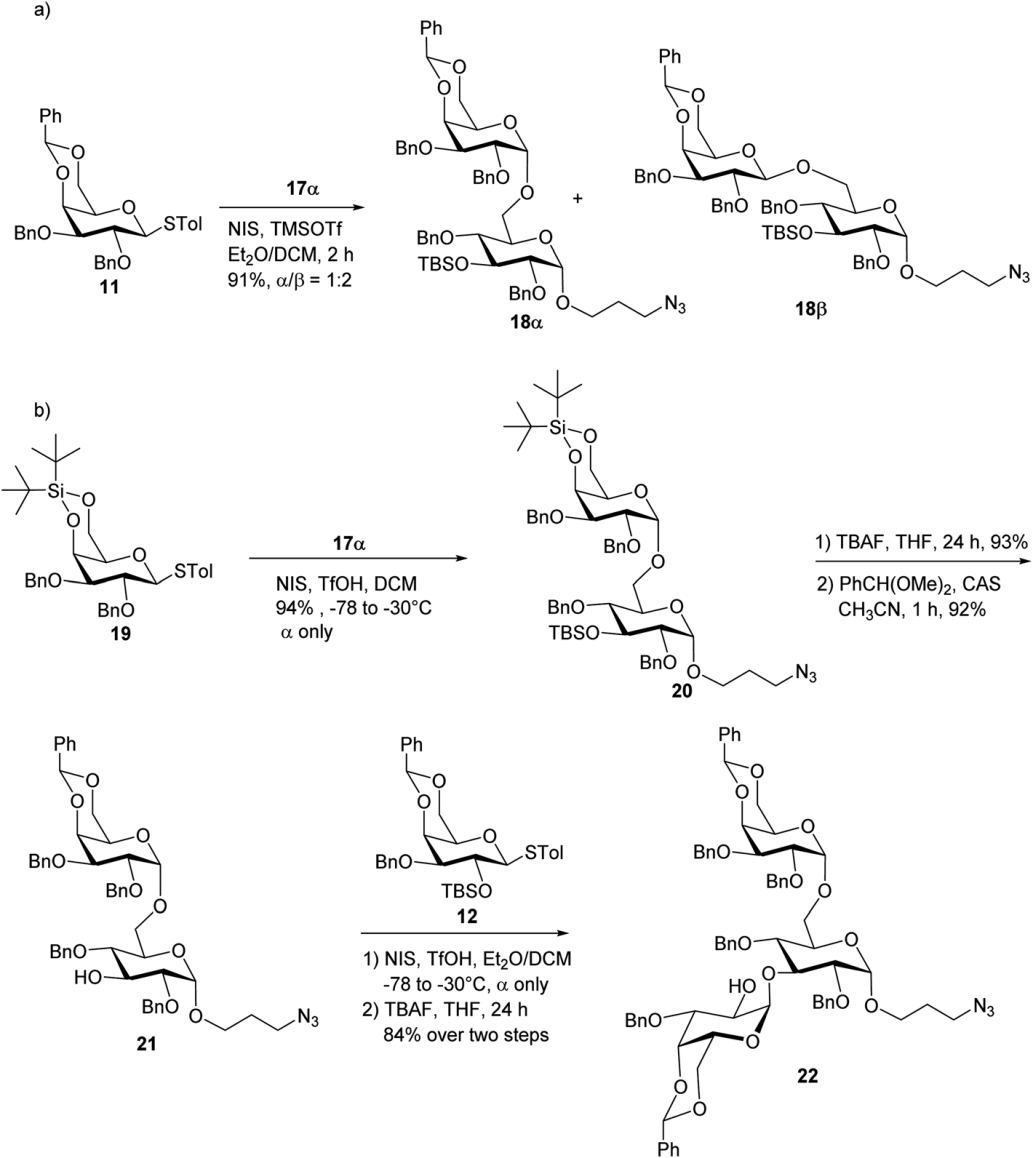
(a) Syntheses of disaccharide acceptor 18α and 18β; and (b) synthesis of trisaccharide acceptor 22.

With the reducing end building blocks prepared, glycosylation between compounds 11^35^ and 17α was initiated using NIS and TfOH as the promoter. Although donor 11 lacked a classical participating neighboring group at the 2-*O* position, this reaction led to the β-configured disaccharide 18β as the major product (Scheme 3a). To achieve the desired α-linked disaccharide, 4,6-*O*-silylidene protected donor 19^36^ was subsequently explored.^37^ Glycosylation of 19 with 17α led to compound 20 exclusively as the α-anomer in 94% yield. The α-configuration of the new galactopyranosidic linkage in 20 was determined by its H-1/H-2 coupling constant (^3^*J*_H-1/H-2_ = 3.6 Hz). Transformation of compound 20 into the disaccharide acceptor 21 involved the deprotection of the silylidene and *tert*-butyldimethylsilyl (TBS) groups with tetrabutylammonium fluoride (TBAF), followed by benzylidene protection at galactose's 4-*O* and 6-*O* positions generating disaccharide acceptor 21. The glycosylation of compound 21 with thiogalactosyl donor 12 proceeded efficiently, affording the desired trisaccharide. Subsequent removal of the TBS protecting group using TBAF furnished trisaccharide acceptor 22 in an overall yield of 84% over two steps. The newly formed galactopyranosidic linkage was confirmed by its characteristic ^3^*J*_H-1/H-2_ coupling constant of 3.8 Hz (Scheme 3b).

**Scheme 4:**
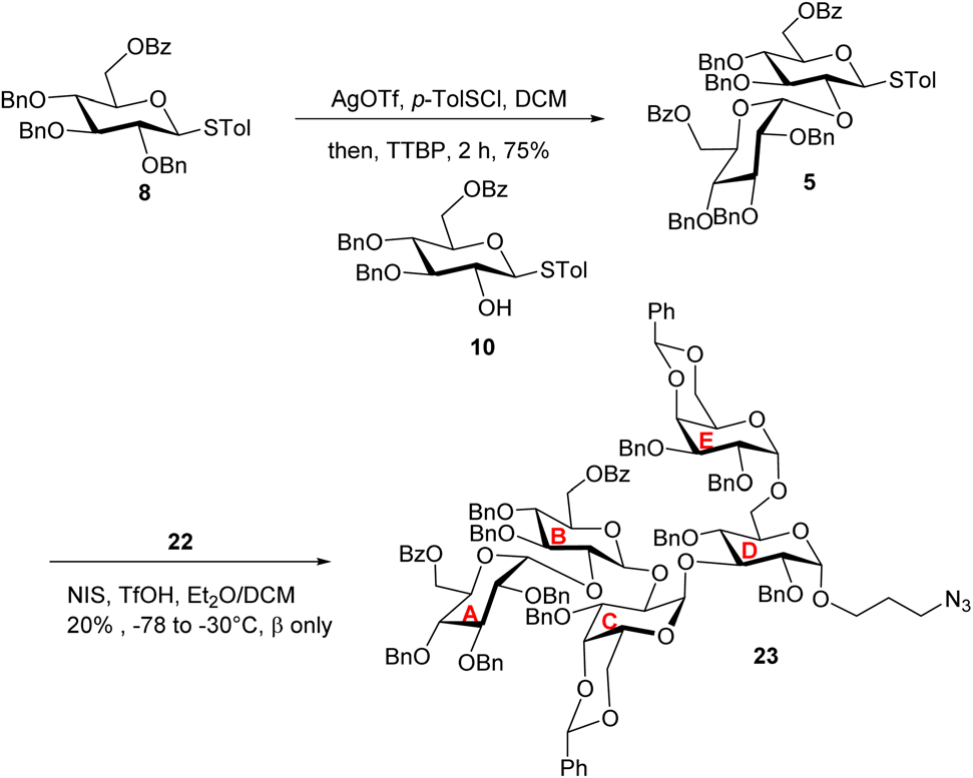
[2 + 3] glycosylation between disaccharide donor 5 and acceptor 22 gave the pentasaccharide 23 with the undesired β-linkage between B and C units.

To prepare the non-reducing end disaccharide donor, the 6-*O* Bz bearing thioglucosyl donor 8^38^ was pre-activated with *p*-TolSCl/AgOTf^39^ at −78 °C and glycosylated thioglycosyl acceptor 10 producing disaccharide 5 in 75% yield (Scheme 4). The H-1/H-2 coupling constant of the newly formed glucopyranosidic linkage in 5 was 3.8 Hz indicating α-bond formation. With disaccharide donor 5 and trisaccharide 22 in hand, we proceeded to construct the pentasaccharide framework *via* the [2 + 3] glycosylation reaction (Scheme 4). Unexpectedly, the resulting glycosidic bond had the β-configuration with no α-glycoside product isolated. The configuration of the newly formed glycosidic linkage was determined by ^1^H-coupled gHSQC-NMR, with a coupling constant of ^3^*J*_H-1/H-2_ at 7.8 Hz and ^3^*J*_H-1/C-1_ at 164.4 Hz.^40^

**Scheme 5:**
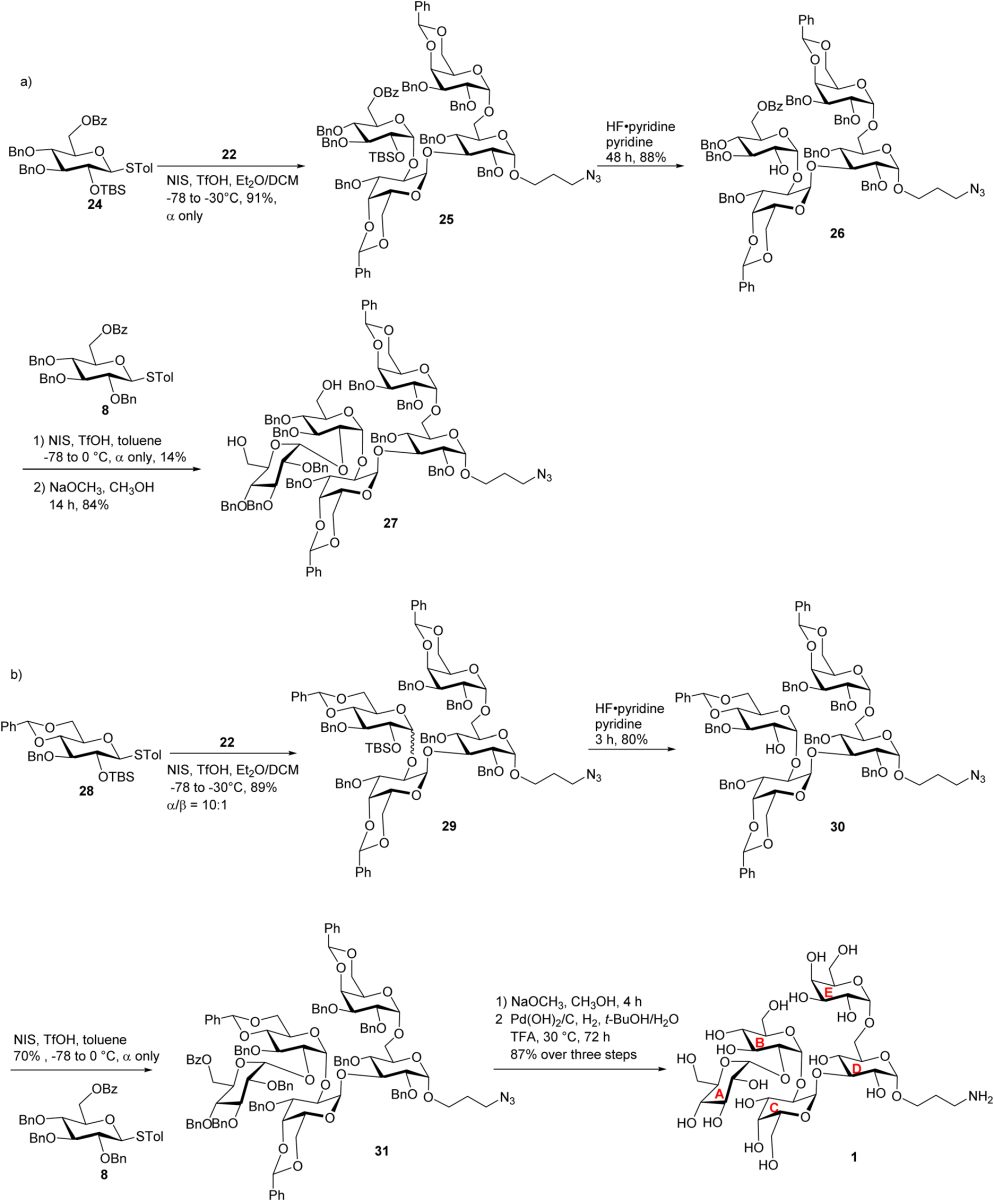
(a) Synthesis of the fully protected pentasaccharide 27; and (b) synthesis of target pentasaccharide 1.

To overcome the stereoselectivity problem in forming the glycosidic linkage between the B and C units in 23, an alternative stepwise sugar chain extension strategy was investigated. The 2-*O* TBS protected glucoside donor 24 underwent glycosylation with the trisaccharide acceptor 22 in the presence of NIS and TfOH with DCM and Et_2_O as solvents, yielding tetrasaccharide 25 (Scheme 5a). Unlike in the synthesis of pentasaccharide 23, the newly formed glucopyranosidic linkage in 25 has a ^3^*J*_H-1/H-2_ of 3.2 Hz supporting the α linkage. The divergent stereochemical outcome with disaccharide donor 5*vs.* monosaccharide 24 was presumably due to the higher steric hindrance posed by the glycan ring at the 2-*O* position of 5, which precluded the nucleophilic attack by the acceptor from the α face. Deprotection of the TBS group from tetrasaccharide 25 by HF·pyridine afforded tetrasaccharide acceptor 26 with a yield of 88%. This reaction progressed slowly requiring 48 hours for completion. Acceptor 26 was glycosylated with monosaccharide donor 8 in toluene at room temperature for 2 hours, affording the glycoside product in 14% yield. It is noteworthy that when DCM was used as the solvent, no desired product was isolated. Subsequent Bz deprotection gave exclusively pentasaccharide 27 with the α-configuration in 84% yield.

To better understand the low yield in the formation of 27, we performed conformational analysis of compound 26. Density Functional Theory (DFT) calculation indicated that in compound 26, its free hydroxyl group (OH) is well shielded by neighboring groups (Fig. S2†), thus presenting low nucleophilicities. To reduce the steric hindrance, we designed the 4,6-*O* benzylidene protected glucosyl acceptor 28 (Scheme 5b). Computational analysis showed that in the corresponding tetrasaccharide (compound 30), its free OH group is more accessible (Fig. S2a†), which should enhance the glycosylation yield. To test this hypothesis, we started with the glycosylation of trisaccharide acceptor 22 by monosaccharide donor 28 under NIS and TfOH condition, which led to an α/β ratio of 10 : 1 of tetrasaccharide 29 in 89% yield (Scheme 5b). The subsequent TBS deprotection of this mixture was completed within 2 hours, which was significantly faster than the TBS deprotection from 25 supporting the reduced steric hindrance around the 2-*O*-TBS moiety in 29. Following TBS deprotection, the α and β isomers were separated using silica gel column chromatography yielding the pure α anomer 30 in 80% yield. In compound 30, the newly formed glucosidic bond had a chemical shift at 4.92 ppm with a coupling constant of ^3^*J*_H-1/H-2_ of 3.9 Hz. The modified tetrasaccharide acceptor 30 was then glycosylated with monosaccharide donor 8, yielding the pentasaccharide 31 in 70% yield. The change of 4-*O* and 6-*O* benzyl groups in tetrasaccharide 26 to the 4,6-*O* benzylidene protective group in tetrasaccharide acceptor 30 increased the yield of the pentasaccharide product by fivefold.

After achieving the fully protected pentasaccharide framework as 31, its Bz groups were removed using sodium methoxide, followed by catalytic hydrogenation and azide reduction, ultimately yielding the target product 1 with an overall yield of 87% in two steps (Scheme 5b). The structure of compound 1 was confirmed by NMR and high-resolution mass spectrometry (HRMS) analyses. All glycosidic bond configurations in 1 were verified based on NMR data (^3^*J*_H-1/H-2_ coupling constants): ^3^*J*_H-1/H-2_ of 3.5 Hz corresponds to unit A α-Glc*p* linkage; ^3^*J*_H-1/H-2_ of 3.8 Hz corresponds to unit B α-Glc*p* linkage; the ^3^*J*_H-1/H-2_ of unit C α-Gal*p* linkage is 3.8 Hz; ^3^*J*_H-1/H-2_ of 3.8 Hz corresponds to unit D α-Glc*p*; and the ^3^*J*_H-1/H-2_ of 2.2 Hz is associated with unit E α-Gal*p*. Further support for the structure came from the HRMS data, which provided an (M + H)^+^ signal at *m*/*z* 886.3397 (calcd 886.3397). In addition, ROESY-NMR analysis was performed on pentasaccharide 1. Correlations were observed between the anomeric protons of the three monosaccharide units from the non-reducing end (Fig. S2b†), suggesting that these three residues were close to each other in space confirming the sterically congested nature of pentasaccharide 1.

**Scheme 6:**
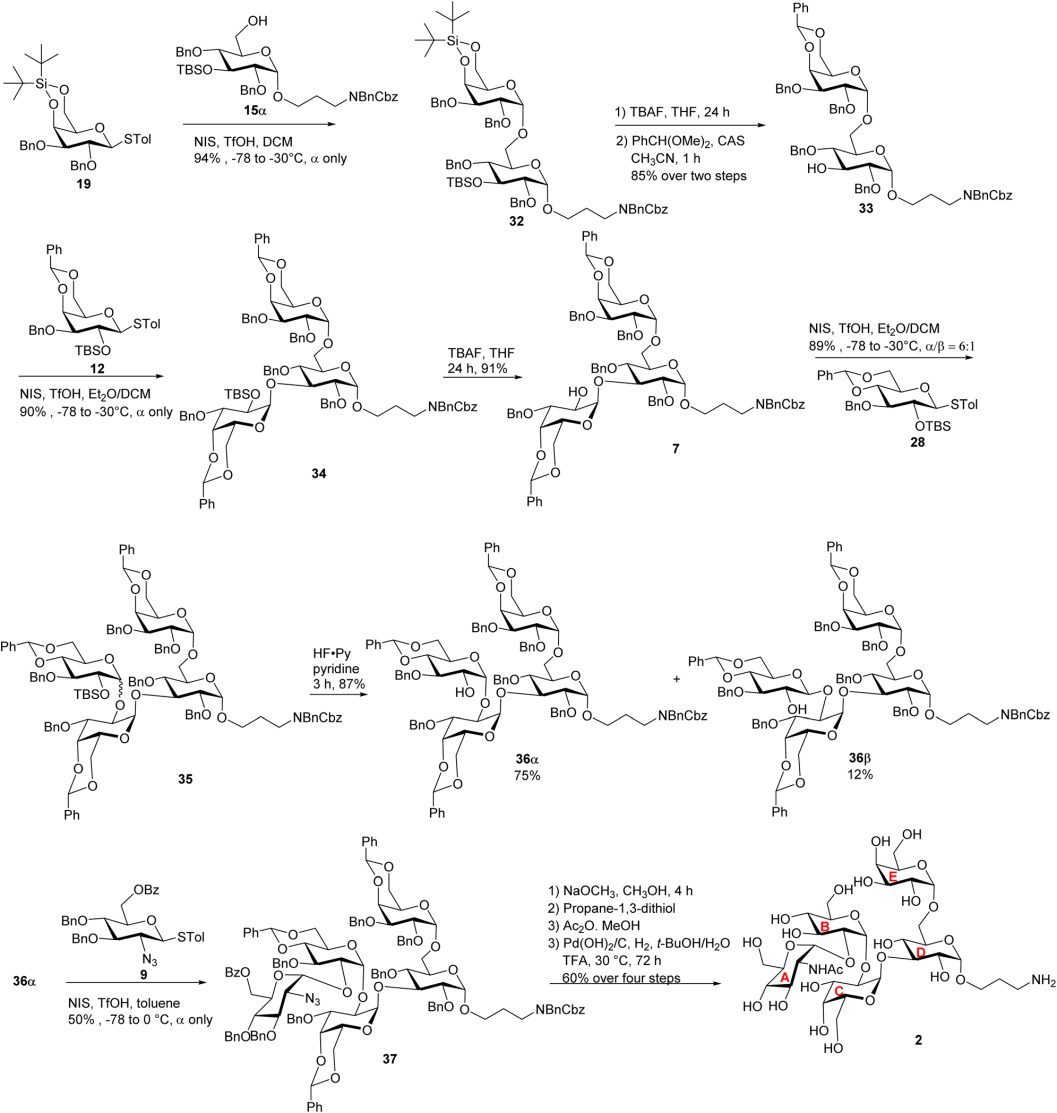
Synthesis of the target pentasaccharide 2.

Pentasaccharides 1 and 2 share similar structural features, enabling the synthesis strategy developed for compound 1 to be adapted to compound 2. For compound 2, as its non-reducing end unit (unit A) is 1,2-*cis* linked *N*-acetyl (NHAc) glucosamine, donor 9 bearing a 2-N_3_ moiety was selected to form the non-reducing end linkage between units A and B. To avoid the need to differentiate multiple azido groups, 15α was utilized as the reducing end building block. 15α was glycosylated by galactose donor 19 producing compound 32 in 94% yield (Scheme 6). The newly formed galactopyranosyl linkage was determined to be α as it displayed a coupling constant (^3^*J*_H-1/H-2_) of 3.5 Hz. Compound 32 was then converted into 33 through TBS and silylidene deprotection, followed by benzylidene protection at the 4,6-*O* positions of the galactose residue. With disaccharide 33 in hand, glycosylation was performed between 33 and 12 resulting in compound 34, which was subsequently deprotected to yield acceptor 7. Under NIS/TfOH promotion, 28 reacted with compound 7, generating an α/β mixture of glycosides (α/β = 6 : 1, compound 35). The TBS group of 35 was removed using HF·pyridine, and after purification by flash column chromatography, the resulting compounds 36α and 36β were obtained in 75% and 12% yields, respectively. Using acceptor 36α, the final glycosidic bond was then formed with donor 9,^41^ leading to compound 37 with exclusive α-selectivity and a 50% yield. The coupling constants of the five α-glycosidic bonds were confirmed by the ^1^H–^13^C decoupled gHSQC spectrum to be *J* = 171.9 Hz, 169.8 Hz, 171.7 Hz, 171.9 Hz, and 169.6 Hz. Global deprotection of compound 37 was achieved through three steps: (1) removal of the Bz group with NaOCH_3_, (2) reduction of the azido group to an amine using propane-1,3-dithiol, followed by acetylation of the free amine, and (3) catalytic hydrogenation over Pd(OH)_2_/C to remove all remaining protecting groups. After purification through size exclusion chromatography, the deprotected pentasaccharide was obtained as a white solid, yielding 2 in 60% over four steps. The glycosidic bond configurations in 2 were confirmed by NMR data (^3^*J*_H-1/H-2_ coupling constants): the ^3^*J*_H-1/H-2_ of the unit A α-Glc*p* linkage is 3.7 Hz; the ^3^*J*_H-1/H-2_ of the unit B α-Glc*p* is 3.7 Hz; the ^3^*J*_H-1/H-2_ of the unit C α-Gal*p* linkage is 3.6 Hz; the ^3^*J*_H-1/H-2_ of unit D α-Glc*p* is 3.9 Hz, and the ^3^*J*_H-1/H-2_ of the unit E α-Gal*p* linkage is 3.7 Hz. Further confirmation of the structure came from HRMS data, showing an (M + H)^+^ signal at *m*/*z* 927.3633 (calcd 927.3669). In parallel to the syntheses of pentasaccharides 1 and 2, outer core tetrasaccharide 3 and disaccharide 4 were synthesized (Scheme S8†).

### Bioconjugation of *Salmonella* LPS outer core glycans to enable vaccine studies

**Scheme 7:**
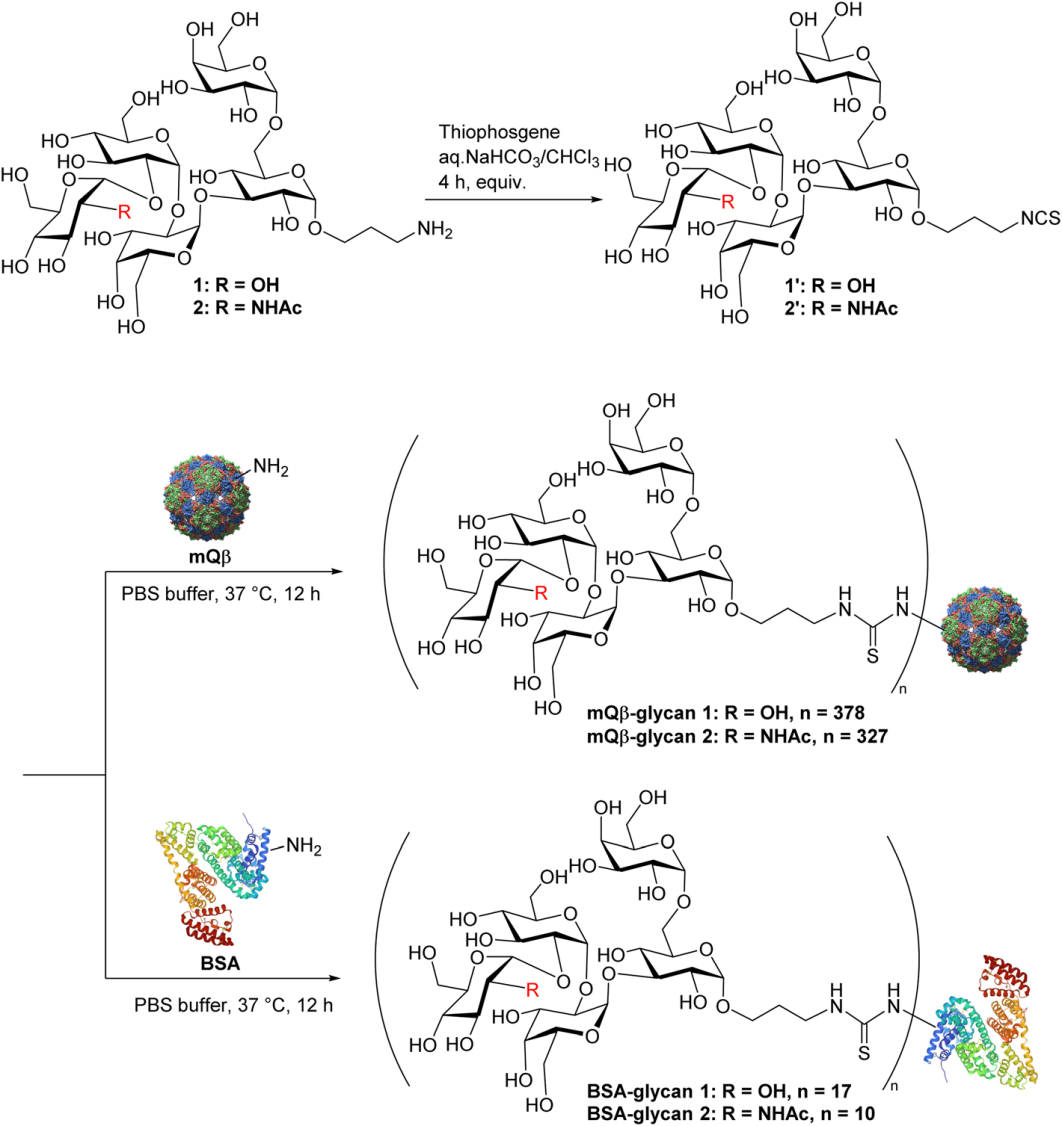
Conjugation of glycans with mQβ and BSA.

Carbohydrate antigens are generally T cell-independent B cell antigens, resulting in weak immunogenicity when administered alone. Conjugation with an appropriate carrier is a well-established strategy to enhance the anti-carbohydrate antibody responses.^42,43^ The mutant bacteriophage Qβ (mQβ) has shown considerable potential as a carrier for carbohydrate-based conjugate vaccines.^44–46^ To enable efficient bio-conjugation of antigens 1 and 2 with mQβ, an isothiocyanate (NCS) group was introduced at the N-terminus of each antigen, producing pentasaccharides 1′ and 2′, which were incubated overnight with mQβ (6 equiv. per amine) (Scheme 7). LC-MS analysis indicated that an average of 378 copies of 1 and 327 copies of 2 were loaded onto each mQβ particle respectively. To measure anti-glycan responses while minimizing interference from anti-mQβ antibodies, bovine serum albumin (BSA)-glycan 1 and 2 conjugates were synthesized (Scheme 7) and used as the coating antigen in ELISA analyses. In addition, BSA conjugates with glycans 3 and 4 were synthesized for epitope mapping, bearing an average of 13 and 20 glycan moieties per BSA molecule, respectively, as quantified by MALDI-MS (Fig. S3†).

### Both mQβ-glycan 1 and mQβ-glycan 2 induced high antibody responses in mice and rabbits

**Fig. 2:**
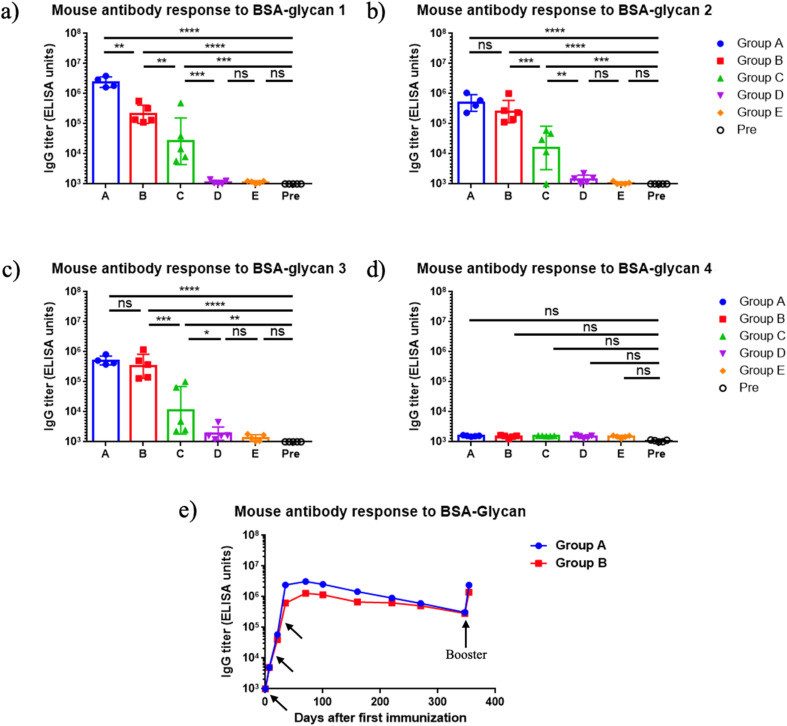
(a–d) Endpoint values of serum IgG titers at day 35 after immunization against various oligosaccharides. Groups A–E refer to the groups in Table 1. Pre: pre-immunization mouse sera. The endpoint value represents the reciprocal of the last serum dilution that yields an absorbance value three times the standard deviation above the plate blank. Each symbol corresponds to an individual animal. Data are presented as geometric mean values ± standard deviation. Statistical significance was determined using one-way ANOVA followed by Tukey's multiple comparisons test. ns: *p* > 0.05; *: *p* ≤ 0.05; **: *p* ≤ 0.01; ***: *p* ≤ 0.001; ****: *p* ≤ 0.0001. (e) Persistence of antibody responses. Each symbol represents pooled sera from group A (measured against BSA-glycan 1) or group B (measured against BSA-glycan 2) at various time points. Arrows indicate the days of vaccination (days 0, 14, 28, 360).

The immunogenicity of mQβ-glycan 1 and mQβ-glycan 2 constructs was evaluated in female C57BL/6 mice (*n* = 5 per group) through a three-dose subcutaneous immunization schedule on days 0, 14, and 28 at 1 μg or 5 μg of glycan antigen per dose with or without aluminum hydroxide (Alum) adjuvant (Table 1). Thirty-five days post-initial immunization, mouse serum antibody titers were assessed by enzyme-linked immunosorbent assay (ELISA). Group A, immunized with mQβ-glycan 1 (5 μg of glycan 1 per dose), demonstrated high anti-glycan 1 IgG titers, averaging 2.4 × 10^6^ ELISA units (Fig. 2a). Similarly, Group B, receiving mQβ-glycan 2 (5 μg of glycan 2 per dose), achieved a high geometric mean anti-glycan 2 IgG titer of 6.2 × 10^5^ ELISA units (Fig. 2b). In contrast, group C with 1 μg of glycan 2 per dose showed inconsistent responses, with significant variability across mice and a geometric mean anti-glycan 2 IgG titer of 1.6 × 10^4^ ELISA units (Fig. 2b). Group D (mQβ-glycan 2, 1 μg of glycan 2 per dose, no Alum adjuvant) and group E (admixture of mQβ with 5 μg of glycans 1 and 2 each) exhibited low anti-glycan IgG titers below 1000 ELISA units (Fig. 2a and b), which were similar to the pre-vaccination (Pre) levels. These findings suggest that adjuvant inclusion is essential for eliciting robust antibody responses at the 1 μg dose, and more consistent immune responses were induced with 5 μg of antigen. In addition, it is critical to covalently conjugate the mQβ carrier with the antigen.

**Table 1:** Experimental mouse groups and immunization parameters

Group	Vaccine construct	Glycan per dose (μg)	Adjuvant	Immunization schedule (days)
A	mQβ-glycan 1	5	Alum	0, 14, 28
B	mQβ-glycan 2	5	Alum	0, 14, 28
C	mQβ-glycan 2	1	Alum	0, 14, 28
D	mQβ-glycan 2	1	—	0, 14, 28
E	mQβ, glycan 1, glycan 2 admixture	5	Alum	0, 14, 28

To profile the epitope specificity of antibodies induced by mQβ-glycan 1 and mQβ-glycan 2, cross-recognition of the two pentasaccharides was examined using sera collected on day 35 post-primary immunization (Fig. 2a and b), and the binding of the post-immunization sera with BSA conjugates of tetrasaccharide 3 and disaccharide 4 was also analyzed (Fig. 2c and d). Group A (immunized with mQβ-glycan 1) exhibited high levels of anti-glycan 2 IgG antibodies, with a geometric mean IgG titer of 5 × 10^5^ ELISA units (Fig. 2b). Conversely, Group B (immunized with mQβ-glycan 2) demonstrated an average anti-glycan 1 IgG titer of 2 × 10^5^ ELISA units. The ELISA titers against tetrasaccharide 3 were measured as 5.1 × 10^5^ ELISA units for group A and 3.3 × 10^5^ ELISA units for group B, suggesting Glc and GlcNHAc at the non-reducing ends of pentasaccharides 1 and 2 did not significantly influence antigen binding. No binding was detected for disaccharide 4, indicating that the reducing end disaccharide is not a major epitope for mouse antibody recognition.

For an effective vaccine, the ability to generate long-term immune responses is highly desired. Monitoring the serum IgG titers over more than 360 days demonstrated that the antibody responses induced by mQβ-glycan 1 and mQβ-glycan 2 were durable. As shown in Fig. 2e, average IgG titers in mice immunized with mQβ-glycan 1 and mQβ-glycan 2 peaked on day 56, reaching 3.1 × 10^6^ and 1.3 × 10^6^ ELISA units, respectively. Strong IgG responses were maintained with average IgG titers of 3.1 × 10^5^ and 2.9 × 10^5^ ELISA units on day 360. Following a booster on day 360, antibody titers returned to near peak values, suggesting that vaccine-induced immune memory was generated and could be effectively recalled even after a prolonged period. In contrast, Groups D and E showed low anti-glycan titers (<10^3^ ELISA units) throughout the study. These results suggest that mQβ-glycan 1 and mQβ-glycan 2, when administered with Alum, elicit robust, long-lasting antibody response and durable immunity.

**Fig. 3:**
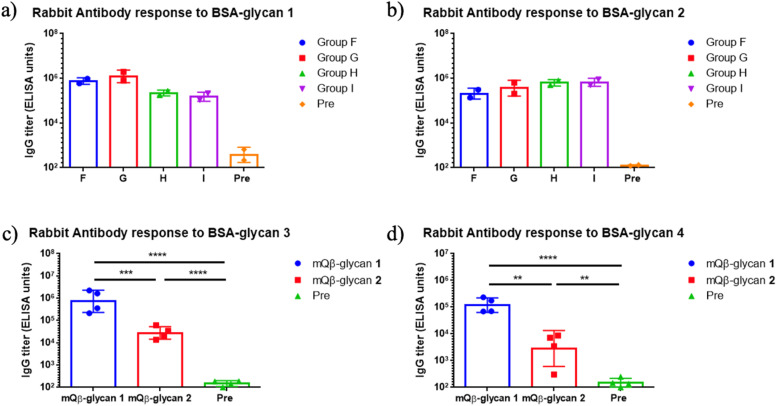
(a–d) Endpoint values of IgG titers against various oligosaccharides. The endpoint value represents the reciprocal of the last serum dilution that yields a signal three times the standard deviation above the plate blank. Each symbol corresponds to an individual animal. Data are presented as geometric mean values ± standard deviation. Statistical significance was determined using one-way ANOVA followed by Tukey's multiple comparisons test. **: *p* ≤ 0.01; ***: *p* ≤ 0.001; ****: *p* ≤ 0.0001. Pre: pre-immunization rabbit sera.

To evaluate immunogenicity across multiple species and aid in future translation, rabbits were immunized subcutaneously with mQβ-glycan 1 and mQβ-glycan 2. Four groups of rabbits received various vaccines as outlined in Table 2. Injections were administered on days 0, 14, 28, and 42, with the final blood sample collected on day 49. ELISA analysis of post-immune sera demonstrated robust anti-glycan IgG responses in all four groups with endpoint titers exceeding 10^5^ ELISA units (Fig. 3a and b), while the anti-glycan IgG titers in pre-immune sera were around 200 ELISA units. No adverse effects from the vaccinations were observed in either rabbits or mice.

**Table 2:** Experimental rabbit groups and immunization details

Group	Vaccine construct	Glycan per dose (μg)	Adjuvant	Immunization schedule (days)
F	mQβ-glycan 1	5	Alum	0, 14, 28, 42
G	mQβ-glycan 1	15	Alum	0, 14, 28, 42
H	mQβ-glycan 2	5	Alum	0, 14, 28, 42
I	mQβ-glycan 2	5	MPLA	0, 14, 28, 42

To further assess antibody specificity, BSA conjugates of glycan 3 and glycan 4 were used as coating antigens in ELISA. Post-immune rabbit sera showed a marked increase in binding to glycan 3 compared to pre-immune sera, similar to results observed in mice (Fig. 3c). However, unlike mouse sera, post-immune rabbit sera exhibited a significant increase in binding to glycan 4 (Fig. 3d). These results suggest that, even with the same antigenic construct, the epitope profiles recognized by antibodies can differ across animal species.

Next, we analyzed vaccine-induced IgG antibody binding to the native polysaccharide (*i.e.*, core and O-polysaccharide (COPS)) isolated from four *Salmonella* strains. The rabbit sera on day 49 post-vaccination exhibited significantly enhanced binding (by 10- to 50-fold) to COPS compared to pre-immunization levels (see ESI Fig. S5†). In contrast, mouse serum showed no recognition of native COPS. Similar species-specific differences in immune responses were also observed in previous studies.^21,22^ This may be due to the rabbit's distinct evolutionary lineage, limited inbreeding, and unique immune repertoire development, which together enable rabbits to mount broader and more sensitive antibody responses than rodents.^47^

### Immunization with mQβ-glycan 1 and mQβ-glycan 2 elicited high titers of anti-glycan IgG antibodies capable of binding *Salmonella* belonging to different serogroups

**Fig. 4:**
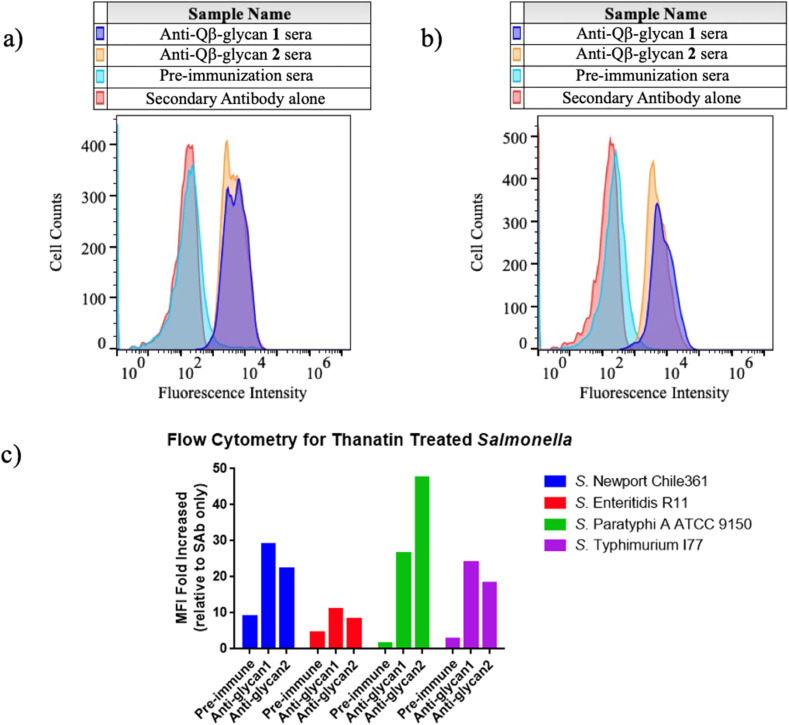
mQβ-glycan 1 and mQβ-glycan 2 induced rabbit antibodies bind *Salmonella* rough strains. (a) Flow cytometry graphs of post-immunization sera (1 : 100 dilution) binding with the *S.* Enteritidis R11 Δ*invA* Δ*rfaL* strain, as compared to the anti-rabbit IgG secondary antibody alone or pre-immunization rabbit sera (1 : 100 dilution). (b) Flow cytometry graphs of post-immunization sera (1 : 100 dilution) binding with the *S.* Newport Δ*rfaL* strain, as compared to the anti-rabbit IgG secondary antibody alone or pre-immunization rabbit sera (1 : 100 dilution). (c) Mean fluorescence intensity (MFI) flow cytometry graphs of pre-immunization rabbit sera (1 : 50 dilution) and post-immunization sera (1 : 50 dilution) binding with thanatin-treated *S.* Enteritidis R11, *S.* Typhimurium I77, *S.* Newport Chile 361, *S.* Paratyphi A ATCC 9150. The results were compared to the fold increase relative to the FTIC-conjugated anti-rabbit IgG secondary antibody alone group. Secondary alone refers to cells treated with secondary antibodies only without any sera.

The binding capacity of rabbit antisera to live *Salmonella* bacteria was assessed next by flow cytometry. As shown in Fig. 4, minimal binding to *Salmonella* rough strains (which lack long-chain OPS but express the core oligosaccharide) was observed for pre-immunization sera at a 1 : 100 dilution, compared to samples without sera. In contrast, IgG antibodies induced by either mQβ-glycan 1 or mQβ-glycan 2 in rabbits showed strong binding to rough strains: *S.* Enteritidis R11 Δ*invA* Δ*rfaL* (Fig. 4a, ESI Fig. S6†) and *S.* Newport Chile 361 Δ*rfaL* (Fig. 4b, ESI Fig. S6†). In contrast, all sera exhibited slight binding to smooth *Salmonella* strains, including *S.* Enteritidis R11, *S.* Typhimurium I77, *S.* Newport Chile 361 and *S.* Paratyphi A ATCC 9150, which share an outer core structure identical to glycan 1. This indicates that the high density of OPS possibly conceals the core oligosaccharide, preventing antibody access and binding in our experiments.

As a major difference between the smooth and rough strains of *Salmonella* is that smooth strains express long LPS on the surface; we hypothesized that reducing the LPS density on the bacteria may enhance the access and the binding of the core LPS by antibodies. To test this, the four smooth *Salmonella* strains were treated with 0.5 μM thanatin (50% of the minimum inhibitory concentration for Gram-negative bacteria), an inhibitor of LPS transport proteins LptA and LptD.^48^ After thanatin treatment, post-immunization sera showed significantly increased binding compared to pre-immunization sera (Fig. 4c), with no notable difference observed between anti-glycan 1 and anti-glycan 2 rabbit sera. These results suggest that treatment with thanatin significantly improved access to the outer core by antibodies.

**Fig. 5:**
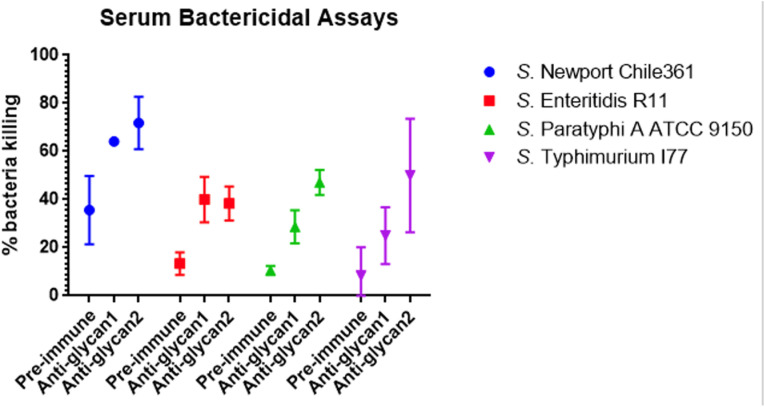
Rabbit anti-mQβ-glycan 1 or anti-mQβ-glycan 2 pooled sera (*n* = 2 per group) enhanced complement-mediated *Salmonella* killing. Log-phase cultures of *Salmonella* were incubated with thanatin, opsonized with heat-inactivated rabbit sera, followed by the addition of baby rabbit complement to eliminate opsonized *Salmonella*. Viable bacterial counts, expressed as colony-forming units (CFUs), were determined through serial dilution and plating.

With enhanced binding following LPS transport inhibition, a serum bactericidal activity assay was conducted to evaluate antibody-dependent, complement-mediated killing. *Salmonella* cells were incubated with thanatin, grown to the logarithmic phase (OD = 0.4), opsonized with sera (diluted by 50-fold) at 4 °C overnight, and incubated with baby rabbit complement at 37 °C for one hour. Anti-glycan sera significantly enhanced the killing of all four *Salmonella* strains compared to pre-immune sera (Fig. 5).

## Conclusions

Oligosaccharides 1–4 associated with *Salmonella* common outer core oligosaccharides have been synthesized using concise synthetic strategies, marking the first successful syntheses of these sequences. The stereoselective formation of all five 1,2-*cis* glycosidic linkages was assisted by judicious choice of protective groups and matching the donor and the acceptor structures. The low yield observed in the key glycosylation steps of compounds 8 and 26 was attributed to steric hindrance around the free OH in acceptor 26 as deciphered by computational studies. Following synthesis, the two outer core pentasaccharides 1 and 2 were successfully conjugated to a powerful immunogenic carrier, bacteriophage mQβ. Parenteral immunization with these conjugates effectively induced potent serum IgG antibody responses in both mice and rabbits. Antibodies produced in immunized rabbits were able to recognize multiple rough strains of *Salmonella*. For the major pathogenic smooth *Salmonella* strains tested, the addition of an LPS transport protein inhibitor thanatin facilitated effective bacterial binding with antiserum. The improved binding of thanatin-treated bacteria to the antiserum was accompanied by significantly more efficient serum bactericidal activities with both anti-glycan 1 and anti-glycan 2 sera compared to pre-immune sera. The induction of a robust antibody response against the LPS core through vaccination followed by treatment with a low concentration of thanatin, an LPS transport inhibitor upon infection, represents a new strategy for further investigation.^49^

## Experimental section

Detailed experimental procedures are provided in the ESI.†

## Ethical statement

All animal procedures were performed in accordance with the Guidelines for Care and Use of Laboratory Animals of Michigan State University and approved by the Institutional Animal Care and Use Committee (IACUC) of Michigan State University (protocol number: 202200444).

## Author contributions

X. H. and X. P. conceived and designed the project. X. P. performed the experimental work. F. S. assisted with mouse cardiac puncture. X. P. and T.-A. C. conducted the computational studies. X. H. supervised both experimental and computational work. S. M. T. and S. M. B. extracted and provided the *Salmonella* COPS and all *Salmonella* strains used in the study. X. P. and X. H. wrote the manuscript. All authors reviewed and approved the final version of the manuscript.

## Conflicts of interest

Xuefei Huang is the founder of Iaso Therapeutics Inc., which is dedicated to the development of the next generation of vaccines. There are no conflicts to declare for other authors.

## Supplementary Material

SC-OLF-D5SC03944D-s001

## Data Availability

The data supporting this article have been included as part of the ESI.†
