# Supplementary material for: Synthesis and immunological evaluation of the lipopolysaccharide outer core of Salmonella for potential broad-spectrum protection against multiple Salmonella serovars
Source: Chem Sci. 2025 Jul 10;16(32):14710–23. doi: 10.1039/d5sc03944d (PMC12261950; doi:10.1039/d5sc03944d)
Supplement: SC-016-D5SC03944D-s001 [file SC-016-D5SC03944D-s001.pdf]

Supplementary Information (SI) for Chemical Science.
This journal is © The Royal Society of Chemistry 2025

Supporting Information

Table of contents

Animal study ethics statement S5
General experimental procedures S5
Figure S1. Chemical structures of *Salmonella* LPS S6
Scheme S1. Synthesis of monosaccharide donor **1** S6
Scheme S2. Synthesis of monosaccharide donors **24** and **28** S7
Scheme S3. Synthesis of monosaccharide acceptors **15** and **17** S9
Scheme S4. Synthesis of trisaccharide acceptor **22** S11
Scheme S5. Synthesis of pentasaccharide donor **23** S14
Scheme S6. Synthesis of pentasaccharide **1** S16
Scheme S7. Synthesis of pentasaccharide **2** S22
Scheme S8. Synthesis of fragments of the pentasaccharides **3** and **4** S28
Computational Methods and Results S29
Figure S2. Geometry optimized structures of **26** and **30**,
and ROESY-NMR spectra of compound **1** S30
Synthesis of BSA-glycan conjugates S30
Synthesis of mQβ-glycan conjugates S31
Scheme S9: Synthesis of BSA-glycan **3** and BSA-glycan **4** conjugates
Immunization protocol S31
Evaluation of antibody titers by ELISA S32
Figure S3. MALDI-TOF mass spectra of representative BSA-glycan conjugates S36
Figure S4. ESI-TOF HRMS mass spectra of mQβ-glycan and conjugates S38
Figure S5. ELISA analysis for antibody binding to native *Salmonella* COPS S39
Figure S6. mQβ-glycans induced rabbit antibodies bind *Salmonella* bacteria S40
Figure S7. Rabbit antibodies bound thanatin treated *Salmonella* strains S41
Table S1. NMR data of compound **10** S42
Table S2. NMR data of compound **12** S42
Table S3. NMR data of compound **17α** S43
Table S4. NMR data of compound **5** S43
Table S5. NMR data of compound **32** S44
Table S6. NMR data of compound **22** S45
Table S7. NMR data of compound **26** S46
Table S8. NMR data of compound **31** S47
Table S9. NMR data of compound **3** S48
Table S10. NMR data of compound **4** S49
Table S11. NMR data of compound **1** S50
Table S12. NMR data of compound **2** S52
References S54

NMR spectra **See NMR spectra file**

Compound No.	Spectra	Page
**10**	^{1}H-NMR, ^{13}C-NMR, gCOSY, gHSQC, gHMBC	**S55**
**12**	^{1}H-NMR, ^{13}C-NMR, gCOSY, gHSQC, gHMBC	**S58**
**28**	^{1}H-NMR, ^{13}C-NMR	**S61**
**24**	^{1}H-NMR, ^{13}C-NMR	**S62**
**5**	^{1}H-NMR, ^{13}C-NMR, gCOSY, gHMBC, coupled gHSQC	**S63**
**15α**	^{1}H-NMR, ^{13}C-NMR, coupled gHSQC	**S66**
**15β**	^{1}H-NMR, ^{13}C-NMR, coupled gHSQC	**S68**
**17α**	^{1}H-NMR, ^{13}C-NMR, gCOSY, gHSQC, gHMBC	**S70**
**18α**	^{1}H-NMR, ^{13}C-NMR	**S73**
**18β**	^{1}H-NMR, ^{13}C-NMR	**S74**
**20**	^{1}H-NMR, ^{13}C-NMR	**S75**
**21**	^{1}H-NMR, ^{13}C-NMR, coupled gHSQC	**S76**
**32**	^{1}H-NMR, ^{13}C-NMR, gCOSY, gHSQC, gHMBC, coupled gHSQC	**S78**
**33**	^{1}H-NMR, ^{13}C-NMR, coupled gHSQC	**S81**
**22**	^{1}H-NMR, ^{13}C-NMR, gCOSY, gHSQC, gHMBC	**S83**
**34**	^{1}H-NMR, ^{13}C-NMR, gCOSY, gHSQC, gHMBC, coupled gHSQC	**S86**
**7**	^{1}H-NMR, ^{13}C-NMR	**S89**
**25**	^{1}H-NMR, ^{13}C-NMR, gHSQC, coupled gHSQC	**S90**
**26**	^{1}H-NMR, ^{13}C-NMR, gCOSY, gHSQC, gHMBC, coupled gHSQC	**S92**
**29**	^{1}H-NMR	**S95**
**30**	^{1}H-NMR, ^{13}C-NMR, gCOSY, gHSQC, gHMBC, coupled gHSQC	**S96**
**35**	^{1}H-NMR, ^{13}C-NMR	**S99**
**36α**	^{1}H-NMR, ^{13}C-NMR, gCOSY, gHSQC, gHMBC, coupled gHSQC	**S100**
**36β**	^{1}H-NMR, ^{13}C-NMR, coupled gHSQC	**S103**
**23**	^{1}H-NMR, ^{13}C-NMR, gCOSY, coupled gHSQC	**S105**

**31**	^{1}H-NMR, ^{13}C-NMR, gCOSY, gHSQC, gHMBC, coupled gHSQC	**S107**
**37**	^{1}H-NMR, ^{13}C-NMR, gCOSY, gHSQC, gHMBC, coupled gHSQC	**S110**
**4**	^{1}H-NMR, ^{13}C-NMR, gCOSY, gHSQC, gHMBC	**S113**
**3**	^{1}H-NMR, ^{13}C-NMR, gCOSY, gHSQC, gHMBC	**S116**
**1**	^{1}H-NMR, ^{13}C-NMR, gCOSY, gHSQC, gHMBC, coupled gHSQC, HRMS	**S119**
**2**	^{1}H-NMR, ^{13}C-NMR, gCOSY, gHSQC, gHMBC, coupled gHSQC, HRMS	**S123**

Animal study ethics statement

All animal care procedures and experimental protocols have been approved by the Institutional Animal Care and Use Committee (IACUC) of Michigan State University (**protocol number:** 202200444).

General experimental procedures

All chemical reactions were carried out under nitrogen with anhydrous solvents in flame-dried glassware, unless otherwise noted. Glycosylation reactions were performed in the presence of molecular sieves, which were flame–dried right before the reaction under high vacuum. Glycosylation solvents were dried using a solvent purification system and used directly without further drying. Chemicals used were reagent grade as supplied except where noted. Analytical thin-layer chromatography was performed using silica gel 60 F254 glass plates. Compounds were visualized by UV light (254 nm) and by staining with a yellow solution containing $Ce(NH_4)_2(NO_3)_6$ (0.5 g) and $(NH_4)_6Mo_7O_{24}•4H_2O$ (24.0 g) in 6% H_2SO_4 (500 mL). Flash column chromatography was performed on silica gel 60 (230-400 Mesh). Optical rotations were recorded on a Perkin Elemer 341 Polarimeter (λ= 589 nm, 1 dm cell). No unexpected or unusually high safety hazards were encountered during this work.

Mass spectrometry (MS) analysis

ESI-MS measurements were performed according to the published procedures[1] on a Q-TOF Ultima API LC-MS instrument with Waters 2795 Separation Module (Waters Corporation, Milford, MA). MALDI mass spectra were recorded on a Shimadzu Axima-CFR plus MALDI-TOF. The matrix used was 2,5-dihydroxy-benzoic acid (DHB) as the calibration compound.

Nuclear magnetic resonance analysis

Proton and carbon nuclear magnetic resonance spectra (^{1}H NMR and ^{13}C NMR) were recorded on an Agilent-500MHz spectrometer at ambient temperature with $CDCl_3$ as the solvent unless otherwise stated. Chemical shifts are reported in parts per million (ppm) relative to residual protic solvent internal standard $CDCl_3$: ^{1}H NMR at δ 7.26 ppm, ^{13}C NMR at δ 77.36 ppm. All ^{13}C NMR spectra were recorded with complete proton decoupling. Peak and coupling constants assignments are based on ^{1}H-NMR, ^{13}C-NMR, ^{1}H-^{1}H gCOSY and (or) ^{1}H-^{13}C gHSQC and ^{1}H-^{13}C gHMBC experiments.

Characterization of anomeric stereochemistry

The stereochemistry of the newly formed glycosidic linkages in the oligosaccharides and intermediates are determined by $^3J_{(H1, H2)}$ through ^{1}H-NMR and/or $^1J_{(C1, H1)}$ through gHSQC 2-D NMR (without ^{1}H decoupling). For galactosyl and glucosyl building blocks, the smaller coupling constants of $^3J_{(H1, H2)}$ (around 3 Hz) indicate α linkages and larger coupling constants $^3J_{(H1, H2)}$ (7.5 Hz or larger) indicate β linkages. For all glycosyl linkages, the stereochemistry can be further

confirmed as larger $^{1}J_{(C1,\ H1)}$ (around 170 Hz) suggests α linkages and smaller $^{1}J_{(C1,\ H1)}$ (around 160 Hz) for β linkages.[2]

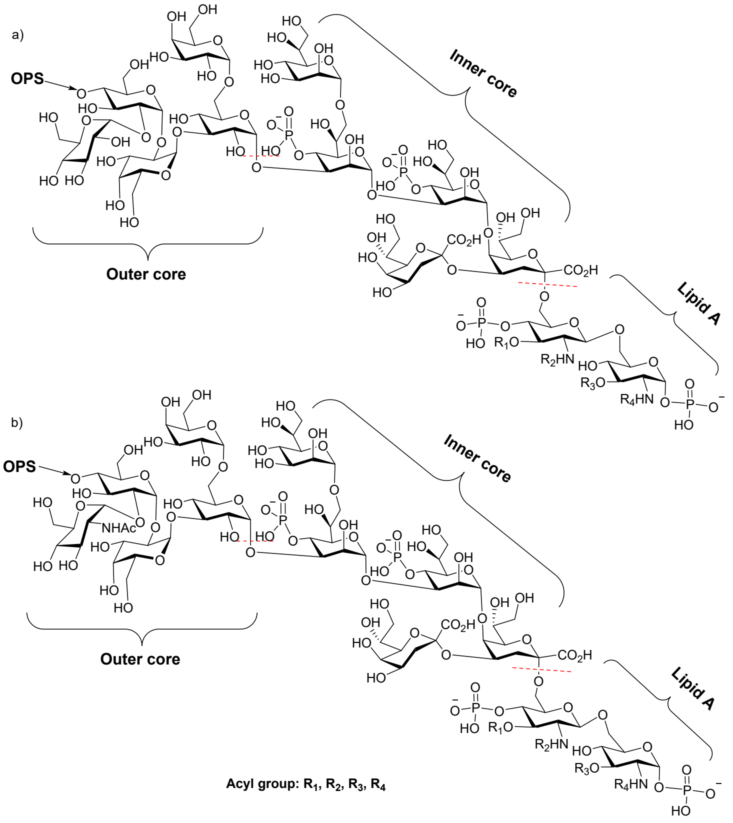

Figure S1. Chemical structures of *Salmonella* LPS including: a) *S. enterica* subsp. IIIa (R1) and b) *S. enterica* subsp. I (R2).

Experimental procedures and characterization data:

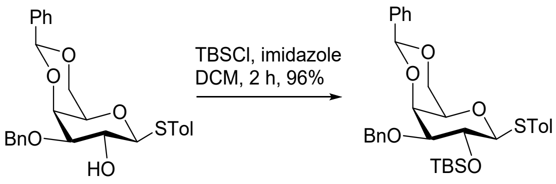

S1 **12**

Scheme S1. Synthesis of monosaccharide donor **12**

p-Tolyl 3-*O*-benzyl-4,6-*O*-benzylidene-2-*O*-tert-butyldimethylsilyl-1-thio-β-D-galactopyranoside (12)

To a solution of compound **S1**[3] (2 g, 3.5 mmol) in CH_2Cl_2 (DCM) (35 mL), imidazole (0.6 g, 8.8 mmol) and TBSCl (0.8 g, 5.3 mmol) were added. The reaction was stirred at room temperature for

2 hours, then quenched with MeOH. The mixture was extracted with DCM and washed with water and brine. The organic phase was dried over Na_2SO_4, filtered, and concentrated under reduced pressure. The residue was purified by flash column chromatography (10:1, hexanes/ethyl acetate), yielding compound **12** (2.4 g, 96% yield). $[\alpha]^{20}_{D}$ -65.7 (*c* 0.21, $CHCl_3$); ^{1}H NMR (500 MHz, $CDCl_3$) δ 7.55-7.53 (m, 2H), 7.50-7.49 (m, 2H), 7.45-7.35 (m, 5H), 7.33-7.25 (m, 3H), 7.01 (d, *J* = 7.7 Hz, 2H), 5.36 (s, 1H), 4.67 (d, *J* = 12.1 Hz, 1H), 4.63 (d, *J* = 12.1 Hz, 1H), 4.52 (d, *J* = 9.0 Hz, 1H), 4.32 (dd, *J* = 12.3, 1.6 Hz, 1H), 4.08 (dd, *J* = 3.5, 1.1 Hz, 1H), 4.02 (t, *J* = 9.0 Hz, 1H), 3.93 (dd, *J* = 12.3, 1.8 Hz, 1H), 3.42 (dd, *J* = 9.0, 3.5 Hz, 1H), 3.37-3.35 (m, 1H), 2.31 (s, 3H), 0.94 (s, 9H), 0.08 (s, 3H), 0.03 (s, 3H). ^{13}C NMR (125 MHz, $CDCl_3$) δ 138.52, 138.17, 136.99, 132.09, 130.65, 129.56, 129.00, 128.34, 128.23, 127.97, 127.75, 126.60, 101.18, 89.52, 82.43, 73.01, 71.25, 69.77, 69.57, 69.21, 26.30, 21.26, 18.58, -3.54, -4.42. HRMS (ESI): *m/z* calcd for $C_{33}H_{42}O_{5S}Si[M+NH_4]^+$: 596.2866, found: 596.2881.

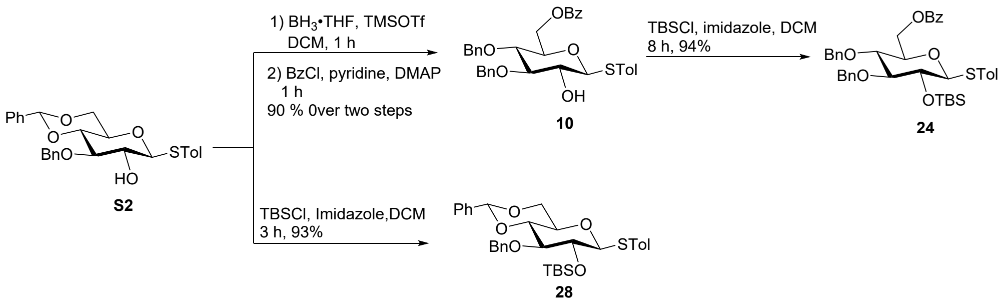

Scheme S2. Synthesis of monosaccharide donors **24** and **28**

***p*-Tolyl 3,4-di-*O*-benzyl-6-*O*-benzoyl-1-thio-β-D-glucopyranoside (10)**

To a solution of compound **S2** (4.7 g, 10.1 mmol) in DCM (100 mL) was added BH_3·THF (53.3 mL, 53.3 mmol) and TMSOTf (0.3 mL, 1.5 mmol) at 0 °C. The mixture was stirred at room temperature for 3 h until TLC showed complete conversion of the starting material. The mixture was cooled down to 0 °C and quenched with MeOH. Organic layer was washed with saturated $NaHCO_3$, dried over Na_2SO_4 and concentrated. The crude mixture was purified by silica gel flash chromatography (3:1, hexanes/ethyl acetate) to give colorless syrup. The obtained compound was dissolved in pyridine (50 mL), cooled to 0 °C and BzCl (0.8 mL, 10.2 mmol) was added dropwise. After 1 h, the solution was diluted with DCM and washed with 1 M HCl, dried over Na_2SO_4, and filtered. The filtrate was concentrated *in vacuo* and the residue was purified by flash chromatography (5:1, hexanes/ethyl acetate) to afford **10** (5.2 g, 90% over two steps) as a white solid. $[\alpha]^{20}_{D}$ +24.1 (*c* 0.1, $CHCl_3$); ^{1}H NMR (500 MHz, $CDCl_3$) δ 8.02-8.0 (m, 2H), 7.62-7.57 (m, 1H), 7.47-7.42 (m, 2H), 7.41-7.35 (m, 4H), 7.34-7.30 (m, 2H), 7.30-7.20 (m, 6H), 6.90 (d, *J* = 7.9 Hz, 2H), 4.96 (d, *J* = 11.0 Hz, 1H), 4.87 (d, *J* = 11.0 Hz, 1H), 4.84 (d, *J* = 11.0 Hz, 1H), 4.69 (dd, *J* = 11.9, 2.1 Hz, 1H), 4.59 (d, *J* = 11.0 Hz, 1H), 4.46 (d, *J* = 9.3 Hz, 1H), 4.44 (dd, *J* = 11.9, 4.5 Hz), 3.69-3.63 (m, 2H), 3.59-3.55 (m, 1H), 3.43 (t, *J* = 9.3 Hz, 1H), 2.25 (s, 3H). ^{13}C NMR (125 MHz, $CDCl_3$) δ 166.16, 138.67, 138.34, 137.65, 134.14, 133.21, 130.04, 129.90, 129.76, 128.67,

128.62, 128.47, 128.26, 128.24, 128.12, 128.04, 126.84, 87.75, 85.96, 77.31, 76.99, 75.63, 75.33, 72.44, 63.35, 21.26. HRMS (ESI): *m/z* calcd for $C_{34}H_{34}O_6S[M+NH_4]^+$: 588.2420, found: 588.2431.

***p*-Tolyl 3,4-di-*O*-benzyl-6-*O*-benzoyl-2-*O*-tert-butyldimethylsilyl-1-thio-β-D-glucopyranoside (24)**

To a solution of compound **10** (3 g, 5.3 mmol) in DCM (50 mL), imidazole (0.9 g, 13.2 mmol) and TBSCl (1.2 g, 8 mmol) were added. The reaction was stirred at room temperature for 8 hours, then quenched with MeOH. The mixture was extracted with DCM and washed with water and brine. The organic phase was dried over Na_2SO_4, filtered, and concentrated under reduced pressure. The residue was purified by flash column chromatography (10:1, hexanes/ethyl acetate), yielding compound **24** (3.5 g, 94% yield). $[\alpha]_D^{20}$ -61.4 (*c* 0.05, $CHCl_3$); ^1H NMR (500 MHz, $CDCl_3$) δ 8.04 (d, *J* = 7.0 Hz, 2H), 7.63-7.59 (m, 1H), 7.51-7.37 (m, 4H), 7.37-7.31 (m, 4H), 7.29-7.26 (m, 1H), 7.25-7.21 (m, 3H), 7.15 (d, *J* = 7.9, 2H), 6.92 (d, *J* = 7.9 Hz, 2H), 4.98 (d, *J* = 11.7 Hz, 1H), 4.89 (d, *J* = 11.7 Hz, 1H), 4.76 (d, *J* = 10.7 Hz, 1H), 4.63 (dd, *J* = 11.8, 2.0 Hz, 1H), 4.55 (dd, *J* = 10.0, 5.7 Hz, 2H), 4.38 (dd, *J* = 11.8, 6.6 Hz, 1H), 3.70-3.68 (m, 2H), 3.62 (t, *J* = 9.2 Hz, 1H), 3.56 (t, *J* = 8.5 Hz, 1H), 2.26 (s, 3H), 0.94 (s, 9H), 0.25 (s, 3H), 0.04 (s, 3H). ^{13}C NMR (125 MHz, $CDCl_3$) δ 166.36, 138.66, 137.52, 137.48, 133.21, 132.14, 131.06, 130.12, 129.96, 129.70, 128.63, 128.51, 128.40, 128.20, 128.16, 127.39, 126.87, 90.38, 87.32, 78.89, 77.21, 75.43, 75.26, 73.88, 64.17, 26.31, 21.26, 18.39, -3.22, -3.60. HRMS (ESI): *m/z* calcd for $C_{40}H_{48}O_6SSi[M+NH_4]^+$: 702.3285, found: 702.3291.

***p*-Tolyl 3-*O*-benzyl-4,6-*O*-benzylidene-2-*O*-tert-butyldimethylsilyl-1-thio-β-D-glucopyranoside (28)**

To a solution of compound **S2**[4] (1 g, 2.2 mmol) in DCM (22 mL), imidazole (0.4 g, 5.5 mmol) and TBSCl (0.5 g, 2.6 mmol) were added. The reaction was stirred at room temperature for 2 hours, then quenched with MeOH. The mixture was extracted with DCM and washed with water and brine. The organic phase was dried over Na_2SO_4, filtered, and concentrated under reduced pressure. The residue was purified by flash column chromatography (10:1, hexanes/ethyl acetate), yielding compound **28** (1.2 g, 93% yield). $[\alpha]_D^{20}$ -81.1 (*c* 0.34, $CHCl_3$); ^1H NMR (500 MHz, $CDCl_3$) δ 7.46-7.40 (m, 4H), 7.40-7.25 (m, 8H), 7.15 (d, *J* = 7.9 Hz, 2H), 5.56 (s, 1H), 5.03 (d, *J* = 11.1 Hz, 1H), 4.72 (d, *J* = 11.1 Hz, 1H), 4.69-4.65 (m, 1H), 4.37 (dd, *J* = 10.5, 5.0 Hz, 1H), 3.81 (t, *J* = 10.5 Hz, 1H), 3.78-3.73 (m, 1H), 3.71-3.64 (m, 2H), 3.54-3.45 (m, 1H), 2.36 (s, 3H), 0.96 (s, 9H), 0.21 (s, 3H), 0.09 (s, 3H). ^{13}C NMR (125 MHz, $CDCl_3$) δ 138.64, 137.78, 137.36, 132.14, 130.63, 129.83, 129.07, 128.35, 128.29, 127.96, 127.53, 126.10, 126.09, 101.31, 90.96, 83.38, 82.20, 74.62, 73.87, 69.95, 68.90, 26.30, 21.27, 18.49, -3.43, -4.09. HRMS (ESI): *m/z* calcd for $C_{33}H_{42}O_5SSi[M+NH_4]^+$: 596.2866, found:596.2879.

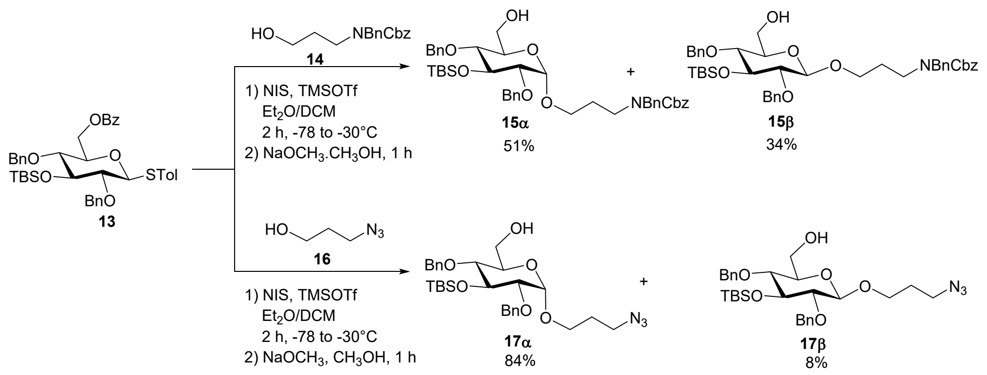

Scheme S3. Synthesis of monosaccharide acceptors **15** and **17**.

***N*-(Benzyl)benzyloxycarbonyl-3-aminopropyl 2,4-di-*O*-benzyl-3-*O*-tert-butyldimethylsilyl-α/β-D-glucopyranoside (15α/β)**

A mixture of galactose donor **13**[5] (680 mg, 1 mmol), acceptor protected 3-amino-1-propanol **14** (359 mg, 1.2 mmol), and freshly activated 4 Å molecular sieves in CH_2Cl_2/Et_2O (v/v = 1:1, 2 mL) were stirred for 15 minutes at room temperature. The suspension was cooled to -78 °C and then NIS (338 mg, 1.5 mmol) and TfOH (9 μL, 0.1 mmol) were added. The reaction mixture was gradually warmed to -30 °C and stirred for 1 h at the same temperature. Then, the mixture was quenched with triethylamine, diluted with CH_2Cl_2 and filtered. The filtrate was concentrated *in vacuo*. The obtained residue was dissolved in MeOH (5 mL). The solution was cooled to 0 °C and NaOMe (32 mg, 0.5 mmol) was added. After stirring 1 h at room temperature, the mixture was neutralized with DOWEX-H^+ ion exchange resins, filtered and concentrated. The residue was purified by flash chromatography (3:1, hexanes/ethyl acetate) to afford **15β** and **15α** (638 mg, 85% over two steps). **15β:** $[\alpha]_D^{20}$ -34.1 (*c* 0.2, $CHCl_3$); ^{1}H NMR (500 MHz, $CDCl_3$) δ 7.36-7.06 (m, 20H), 5.18-5.10 (m, 2H), 4.85 (d, *J* = 11.4 Hz, 1H), 4.89-4.74 (m, 1H), 4.61 (d, *J* = 11.4 Hz, 1H), 4.69-4.54 (m, 1H), 4.50-4.39 (m, 2H), 4.38-4.25 (m, 1H), 3.88-3.74 (m, 2H), 3.71-3.64 (m, 2H), 3.57-3.44 (m, 1H), 3.40 (t, *J* = 9.3 Hz, 2H), 3.31-3.09 (m, 3H), 1.91-1.69 (m, 2H), 0.91 (s, 9H), 0.04 (s, 3H), -0.00 (s, 3H). ^{13}C NMR (125 MHz, $CDCl_3$) δ 156.84, 156.29, 138.82, 138.19, 137.83, 136.68, 128.69, 128.65, 128.55, 128.53, 128.48, 128.33, 128.25, 128.08, 127.96, 127.87, 127.77, 127.63, 127.42, 127.31, 127.24, 104.10, 103.71, 82.58, 82.48, 78.80, 78.63, 76.59, 75.19, 75.03, 74.61, 68.17, 67.35, 62.00, 50.54, 44.80, 43.50, 28.43, 26.14, 18.15, -3.88, -4.06. HRMS (ESI): *m*/*z* calcd for $C_{44}H_{57}NO_8Si[M+NH_4]^+$: 773.4197, found: 773. 4215.

15α : $[\alpha]_D^{20}$ +50.3 (*c* 0.18, $CHCl_3$); ^{1}H NMR (500 MHz, $CDCl_3$) δ 7.51-7.15 (m, 20H), 5.15 (d, *J* = 12.9 Hz, 2H), 4.86 (d, *J* = 11.4 Hz, 1H), 4.67 (d, *J* = 12.2 Hz, 1H), 4.58 (d, *J* = 11.4 Hz, 1H), 4.55-4.39 (m, 4H), 4.00 (t, *J* = 9.0 Hz, 1H), 3.71-3.46 (m, 4H), 3.37-3.10 (m, 5H), 1.84-1.70 (m, 2H), 0.92 (s, 9H), 0.07 (s, 3H), 0.05 (s, 3H). ^{13}C NMR (125 MHz, $CDCl_3$) δ 138.57, 138.34, 137.98, 128.70, 128.51, 128.46, 128.18, 128.07, 127.90, 127.86, 127.51, 97.21, 80.61, 78.93, 75.12, 73.91, 73.41, 71.00, 67.33, 65.41, 62.02, 51.05, 44.77, 28.46, 26.22, 18.27, -3.77, -4.11. HRMS (ESI): *m*/*z* calcd for $C_{44}H_{57}NO_8Si[M+NH_4]^+$: 773.4197, found: 773. 4226.

3-Azidopropyl 2,4-di-*O*-benzyl-3-*O*-*tert*-butyldimethylsilyl-α-D-glucopyranoside (17α)

A mixture of galactose donor **11** (960 mg, 1.4 mmol), acceptor 3-azidopropan-1-ol **16** (170 mg, 1.7 mmol), and freshly activated 4 Å molecular sieves in CH_2Cl_2/Et_2O (v/v = 1:1, 2 mL) were stirred for 15 minutes at room temperature. The suspension was cooled to -78 °C and then NIS (473 mg, 0.66 mmol) and TfOH (51 μL, 0.28 mmol) were added. The reaction mixture was gradually warmed to -30 °C and stirred for 1 h at the same temperature. Then, the mixture was quenched with triethylamine, diluted with CH_2Cl_2 and filtered. The filtrate was concentrated *in vacuo*. The obtained residue was dissolved in MeOH (2 mL), the solution was cooled to 0 °C and NaOMe (112 mg, 1.6 mmol) was added. After stirring 1 h at room temperature, the mixture was neutralized with DOWEX-H^+ ion exchange resins, filtered and concentrated. The residue was purified by flash chromatography (3:1, hexanes/ethyl acetate) to afford **17α** (790 mg, 84%) as white solid. $[\alpha]_D^{20}$ +48.4 (*c* 0.14, $CHCl_3$); ^1H NMR (500 MHz, $CDCl_3$) δ 7.49-7.20 (m, 10H), 4.89 (d, *J* = 11.5 Hz, 1H), 4.73 (d, *J* = 12.1 Hz, 1H), 4.61 (d, *J* = 11.5 Hz, 1H), 4.56 (d, *J* = 3.7 Hz, 1H, H-1), 4.49 (d, *J* = 12.1 Hz, 1H), 4.03 (t, *J* = 9.0 Hz, 1H), 3.71 (dd, *J* = 11.8, 2.8 Hz, 1H), 3.68-3.61 (m, 2H), 3.60-3.56 (m, 1H), 3.43-3.36 (m, 3H), 3.34-3.30 (m, 1H), 3.28 (dd, *J* = 9.3, 3.5 Hz, 1H), 1.91-1.78 (m, 2H), 0.94 (s, 9H), 0.10 (s, 3H), 0.09 (s, 3H). ^{13}C NMR (125 MHz, $CDCl_3$) δ 138.64, 138.32, 128.56, 128.52, 128.15, 127.96, 127.92, 127.87, 97.34, 80.72, 78.75, 77.42, 77.17, 76.91, 75.17, 73.93, 73.50, 71.11, 64.71, 61.96, 48.46, 28.97, 26.24, 18.29, -3.77, -4.10. HRMS (ESI): *m*/*z* calcd for $C_{29}H_{43}N_3O_6Si$ $[M+NH_4]^+$: 575.3265, found: 575.3275.

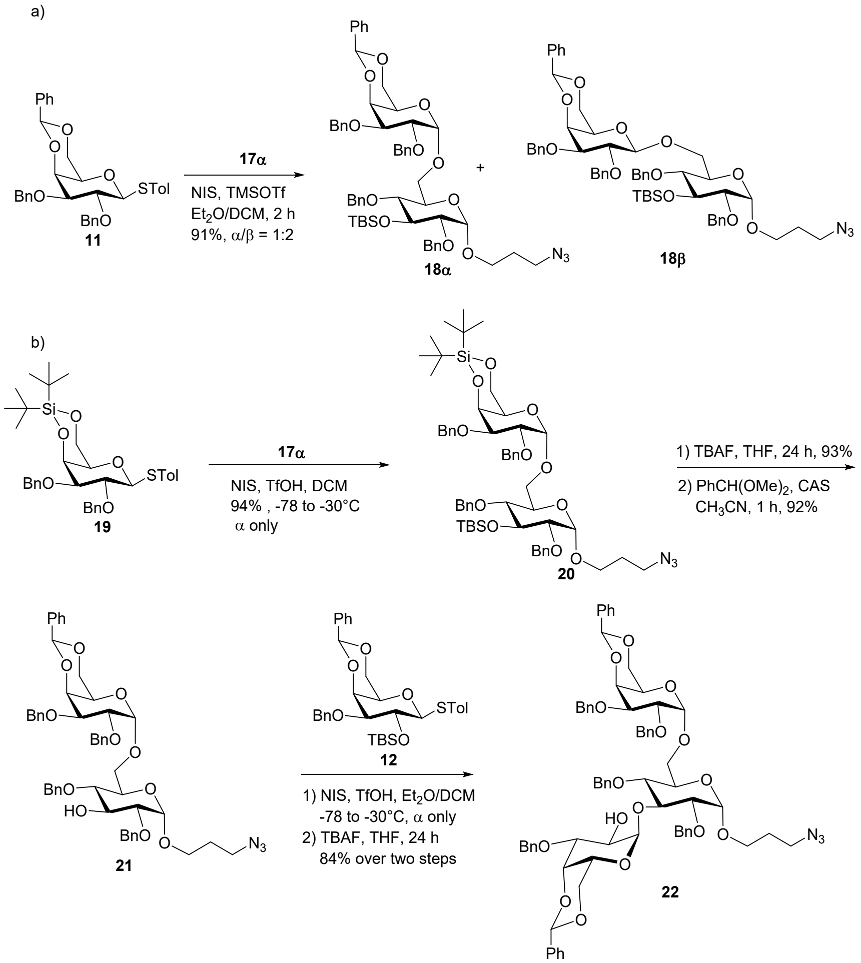

Scheme S4. Synthesis of trisaccharide acceptor **22**.

3-Azidopropyl 2,3-di-*O*-benzyl-4,6-*O*-benzylidene-α/β-D-galactopyranosyl-(1→6)-2,4-di-*O*-benzyl-3-*O*-*tert*-butyldimethylsilyl-α-D-glucopyranoside (18)

A mixture of galactose donor **11**[6] (244 mg, 0.44 mmol), acceptor **17α** (206 mg, 0.37 mmol), and freshly activated 4 Å molecular sieves in CH_2Cl_2/Et_2O (v/v = 1:1, 7 mL) were stirred for 15 minutes at room temperature. The suspension was cooled to -78 °C and then NIS (149 mg, 0.66 mmol) and TfOH (4 μL, 44 μmol) were added. The reaction mixture was gradually warmed to -30 °C and stirred for 1 h at the same temperature. Then, the mixture was quenched with triethylamine, diluted with CH_2Cl_2 and filtered. The filtrate was concentrated *in vacuo*. The obtained residue was purified by silica gel column chromatography (10:1, hexanes/ethyl acetate) to afford **18** (294 mg, 91%) as colorless syrups. **18β**: $[\alpha]_D^{20}$ -14.1 (*c* 0.14, $CHCl_3$); ^1H NMR (500 MHz, $CDCl_3$) δ 7.54-7.53 (m, 2H), 7.40-7.17 (m, 23H), 5.47 (s, 1H), 4.96 (d, *J* = 11.7 Hz, 1H), 4.82 (d, *J* = 11.0 Hz, 1H), 4.75 (d, *J* = 2.5 Hz, 2H), 4.71 (d, *J* = 12.2 Hz, 2H), 4.64 (d, *J* = 3.5 Hz, 1H), 4.53 (d, *J* = 11.7 Hz, 1H), 4.48 (d, *J* = 12.2 Hz, 1H), 4.27-4.23 (m, 2H), 4.16 (dd, *J* = 11.0, 2.2 Hz, 1H), 4.07 (dd, *J* = 3.7, 1.0 Hz, 1H), 4.01 (t, *J* = 9.0 Hz, 1H), 3.97 (dd, *J* = 12.3, 1.8 Hz, 1H), 3.90-3.88 (m, 1H), 3.78-3.76 (m, 1H), 3.70-3.60 (m, 2H), 3.51 (dd, *J* = 9.6, 3.6 Hz, 1H), 3.40 (dd, *J* = 10.1, 8.7 Hz, 1H), 3.34-

3.24 (m, 4H), 3.20 (s, 1H), 1.77 (p, *J* = 6.5 Hz, 2H), 0.92 (s, 9H), 0.08 (s, 3H), 0.01 (s, 3H). ^{13}C NMR (125 MHz, $CDCl_3$) δ 139.03, 138.77, 138.65, 138.50, 137.97, 129.05, 128.49, 128.47, 128.43, 128.40, 128.31, 128.25, 128.12, 127.93, 127.90, 127.88, 127.84, 127.59, 127.39, 127.36, 126.59, 104.12, 101.32, 97.08, 80.60, 79.47, 79.44, 78.24, 75.38, 74.83, 73.97, 73.87, 73.38, 72.03, 70.19, 69.20, 68.56, 66.53, 64.62, 48.46, 28.84, 26.25, 18.27, -3.78, -4.16; **18α**: $[\alpha]_D^{20}$ +34.1 (*c* 0.15, $CHCl_3$); ^{1}H NMR (500 MHz, $CDCl_3$) δ 7.51-7.49 (m, 2H), 7.41-7.20 (m, 23H), 5.44 (s, 1H), 5.11 (d, *J* = 3.5 Hz, 1H), 4.89 (d, *J* = 11.8 Hz, 1H), 4.77 (d, *J* = 5.0 Hz, 2H), 4.74 (s, 2H), 4.60 (d, *J* = 12.1 Hz, 1H), 4.55-4.50 (m, 2H), 4.35 (d, *J* = 12.1 Hz, 1H), 3.98 (t, *J* = 9.1 Hz, 1H), 3.92 (dd, *J* = 10.1, 3.5 Hz, 1H), 3.84 (dd, *J* = 12.5, 1.8 Hz, 1H), 3.77-3.64 (m, 3H), 3.61-3.59 (m, 1H), 3.47 (t, *J* = 9.3 Hz, 1H), 3.40 (d, *J* = 1.6 Hz, 1H), 3.36-3.33 (m, 2H), 3.27-3.25 (m, 1H), 3.15 (dd, *J* = 9.3, 3.6 Hz, 1H), 1.82-1.70 (m, 2H), 0.95 (s, 9H), 0.07 (s, 3H), 0.06 (s, 3H). ^{13}C NMR (125 MHz, $CDCl_3$) δ 139.07, 138.81, 138.80, 138.70, 137.96, 129.02, 128.48, 128.44, 128.42, 128.25, 127.97, 127.92, 127.83, 127.72, 127.57, 127.52, 127.50, 127.29, 126.51, 101.21, 98.43, 97.12, 80.89, 79.51, 75.81, 75.04, 74.89, 74.86, 74.05, 73.33, 72.60, 71.90, 70.75, 69.48, 66.31, 64.57, 62.70, 48.47, 28.91, 26.25, 18.31, -3.80, -4.14. HRMS (ESI): *m*/*z* calcd for: $C_{56}H_{69}N_3O_{11}Si[M+NH_4]^+$: 1005.5045, found: 1005.5081.

3-Azidopropyl 2,3-di-*O*-benzyl-4,6-*O*-di-*tert*-butylsilanediyl-α-D-galactopyranosyl-(1→6)-2,4-di-*O*-benzyl-3-*O*-*tert*-butyldimethylsilyl-α-D-glucopyranoside (20)

A mixture of galactose donor **19**[7] (264 mg, 0.44 mmol), acceptor **17α** (218 mg, 0.39 mmol), and freshly activated 4 Å molecular sieves in CH_2Cl_2/Et_2O (v/v = 1:1, 4 mL) were stirred for 15 minutes at room temperature. The suspension was cooled to -78 °C and then NIS (148 mg, 0.66 mmol) and TfOH (5 µL, 44 µmol) were added. The reaction mixture was gradually warmed to -30 °C and stirred for 1 h at the same temperature. Then, the mixture was quenched with triethylamine, diluted with CH_2Cl_2 and filtered. The filtrate was concentrated *in vacuo*. The obtained residue was purified by silica gel column chromatography (10:1, hexanes/ethyl acetate) to afford **20** (385 mg, 94%) as colorless syrups. $[\alpha]_D^{20}$ +51.3 (*c* 0.3, $CHCl_3$);^{1}H NMR (500 MHz, $CDCl_3$) δ 7.41-7.38 (m, 4H), 7.33-7.27 (m, 13H), 7.25-7.20 (m, 3H), 4.96 (d, *J* = 3.6 Hz, 1H), 4.89 (d, *J* = 12.2 Hz, 1H), 4.81-4.75 (m, 1H), 4.74 (d, *J* = 8.1 Hz, 2H), 4.69 (d, *J* = 12.2 Hz, 1H), 4.62 (d, *J* = 12.2 Hz, 1H), 4.52 (dd, *J* = 7.7, 4.0 Hz, 2H), 4.42 (dd, *J* = 3.0, 1.1 Hz, 1H), 4.37 (d, *J* = 12.2 Hz, 1H), 4.07-3.95 (m, 4H), 3.76 (dd, *J* = 10.0, 3.1 Hz, 1H), 3.72 (dd, *J* = 11.3, 4.1 Hz, 1H), 3.70-3.66 (m, 2H), 3.63-3.59 (m, 1H), 3.47 (t, *J* = 9.4 Hz, 1H), 3.43 (s, 1H), 3.37-3.31 (m, 2H), 3.30-3.22 (m, 1H), 3.16 (dd, *J* = 9.3, 3.6 Hz, 1H), 1.84-1.72 (m, 2H), 1.04 (s, 9H), 0.99 (s, 9H), 0.94 (s, 9H), 0.07 (s, 3H), 0.05 (s, 3H). ^{13}C NMR (125 MHz, $CDCl_3$) δ 139.03, 139.01, 138.78, 138.73, 128.43, 128.40, 128.36, 128.34, 127.97, 127.95, 127.81, 127.78, 127.77, 127.75, 127.65, 127.62, 127.59, 127.54, 127.48, 127.23, 98.27, 97.14, 80.84, 79.56, 76.74, 75.02, 74.49, 74.04, 73.34, 72.62, 71.20, 70.74, 70.67, 67.28, 67.24, 66.19, 64.57, 48.48, 28.93, 27.79, 27.44, 26.24, 23.53, 20.78, 18.29, -3.81, -4.16; HRMS (ESI): *m*/*z* calcd for $C_{57}H_{81}N_4O_{11}Si_2[M+NH_4]^+$: 1057.5753, found: 1057.5785.

3-Azidopropyl 2,3-di-*O*-benzyl-4,6-*O*-benzylidene-α-D-galactopyranosyl-(1→6)-2,4-di-*O*-benzyl-α-D-glucopyranoside (21)

To a solution of **20** (220 mg, 0.21 mmol) in pyridine (2 ml) and HF·pyridine (70% HF in pyridine, 0.2 mL), and the solution was stirred for 3 h at room temperature. The reaction mixture was diluted with DCM, washed with saturated aqueous $NaHCO_3$, dried over Na_2SO_4, and filtered. The filtrate was concentrated *in vacuo* and the residue was purified by flash chromatography (DCM/MeOH, 40:1). The obtained product was dissolved in CH_3CN and camphorsulfonic acid (CSA) (24 mg, 0.1 mmol) and $PhCH(OMe)_2$ (63 μL, 0.42 mmol) were added. After stirring for 1 hour at room temperature, the reaction mixture was diluted with DCM, washed with saturated aqueous $NaHCO_3$, dried over Na_2SO_4, and filtered. The solvent was removed under reduced pressure, and the residue was purified by flash chromatography (3:1, hexanes/ethyl acetate) to yield compound **21** as a white solid (157 mg, 83% yield over two steps). $[\alpha]_D^{20}$ +26.7 (*c* 0.1, $CHCl_3$); ^{1}H NMR (500 MHz, $CDCl_3$) δ 7.53-7.51 (m, 2H), 7.42-7.26 (m, 23H), 5.46 (s, 1H), 5.10 (d, *J* = 3.4 Hz, 1H), 4.93 (d, *J* = 12.0 Hz, 1H), 4.82 (dd, *J* = 12.0, 2.3 Hz, 2H), 4.76 (d, *J* = 12.0 Hz, 1H), 4.71 (d, *J* = 12.0 Hz, 1H), 4.69 (d, *J* = 3.5 Hz, 1H), 4.60 (d, *J* = 12.0 Hz, 2H), 4.54 (d, *J* = 12.0 Hz, 1H), 4.16-4.03 (m, 4H), 3.96 (dd, *J* = 10.1, 3.4 Hz, 1H), 3.86 (dd, *J* = 12.5, 1.8 Hz, 1H), 3.82 (dd, *J* = 11.4, 4.4 Hz, 1H), 3.77-3.65 (m, 3H), 3.58 (t, *J* = 9.3 Hz, 1H), 3.51 (d, *J* = 1.5 Hz, 1H), 3.42-3.30 (m, 3H), 3.26 (dd, *J* = 9.6, 3.5 Hz, 1H), 1.83-1.76 (m, 2H); ^{13}C NMR (125 MHz, $CDCl_3$) δ 139.07, 138.84, 138.72, 138.11, 137.96, 129.01, 128.71, 128.57, 128.45, 128.43, 128.24, 128.22, 128.05, 127.82, 127.80, 127.70, 127.59, 126.49, 101.20, 98.53, 96.50, 80.05, 77.68, 75.79, 75.35, 74.86, 74.50, 73.50, 73.04, 72.96, 72.00, 70.27, 69.48, 66.46, 64.64, 62.74, 48.35, 28.93. HRMS (ESI): *m*/*z* calcd for $C_{50}H_{55}N_3O_{11}[M+NH_4]^+$: 891.4180, found:891.4190.

3-Azidopropyl 3-*O*-benzyl-4,6-*O*-benzylidene-α-D-galactopyranosyl-(1→3)-2,4-di-*O*-benzyl-6-*O*-(2,3-di-*O*-benzyl-4,6-*O*-benzylidene-α-D-galactopyranosyl)-α-D-glucopyranoside (22)

A mixture of galactose donor **12** (460 mg, 0.8 mmol), acceptor **21** (628 mg, 0.7 mmol), and freshly activated 4 Å molecular sieves in CH_2Cl_2/Et_2O (v/v = 1:1, 1.5 mL) were stirred for 15 minutes at room temperature. The suspension was cooled to -78 °C and then NIS (270 mg, 1.2 mmol) and TfOH (7 μL, 0.08 mmol) were added. The reaction mixture was gradually warmed to -30 °C and stirred for 1 h at the same temperature. Then, the mixture was quenched with triethylamine, diluted with CH_2Cl_2 and filtered. The filtrate was concentrated *in vacuo*. The obtained residue was dissolved in THF (2 mL). The solution was cooled to 0 °C and TBAF (0.2 mL) was added. After stirring 1 h at room temperature, the mixture was concentrated *in vacuo*. The obtained residue was purified by silica gel column chromatography (5:1, hexanes/ethyl acetate) to afford **22** (815 mg, 84% over two steps) as colorless syrups. $[\alpha]_D^{20}$ +71.2 (*c* 0.25, $CHCl_3$); ^{1}H NMR (500 MHz, $CDCl_3$) δ 7.63-7.04 (m, 35H), 5.60 (d, *J* = 3.8 Hz, 1H), 5.49 (s, 1H), 5.27 (s, 1H), 5.19 (d, *J* = 3.5 Hz, 1H), 5.03 (d, *J* = 10.8 Hz, 1H), 4.87-4.81 (m, 2H), 4.83 (d, *J* = 3.6 Hz, 1H), 4.81-4.75 (m, 3H), 4.73 (d, *J* = 11.9 Hz, 1H), 4.57 (d, *J* = 10.8 Hz, 1H), 4.51 (d, *J* = 10.7 Hz, 1H), 4.26 (d, *J* = 10.7 Hz, 1H), 4.24-4.15 (m, 4H), 4.11 (dd, *J* = 10.0, 3.5 Hz, 1H), 3.98 (dd, *J* = 10.0, 3.5 Hz, 1H), 3.96-3.91 (m,

2H), 3.90-3.83 (m, 3H), 3.80-3.71 (m, 5H), 3.54 (s, 1H), 3.49-3.35 (m, 3H), 3.33-3.25 (m, 2H), 2.36 (d, J = 6.2 Hz, 1H), 1.84 (p, J = 6.3 Hz, 2H). ^{13}C NMR (125 MHz, $CDCl_3$) δ 139.23, 138.76, 138.49, 138.20, 138.04, 138.01, 137.92, 129.00, 128.83, 128.63, 128.58, 128.51, 128.48, 128.45, 128.42, 128.23, 128.20, 128.15, 128.14, 127.96, 127.88, 127.78, 127.77, 127.75, 127.48, 127.42, 126.47, 126.27, 101.19, 100.73, 98.79, 98.77, 96.38, 79.34, 78.93, 76.62, 75.83, 75.20, 74.79, 74.42, 73.39, 72.66, 72.59, 72.02, 71.12, 70.87, 69.49, 69.45, 67.99, 66.23, 64.53, 62.87, 62.38, 48.29, 28.89. HRMS (ESI): *m/z* calcd for $C_{70}H_{75}N_3O_{16}[M+NH_4]^+$: 1231.5491, found:1231.5533.

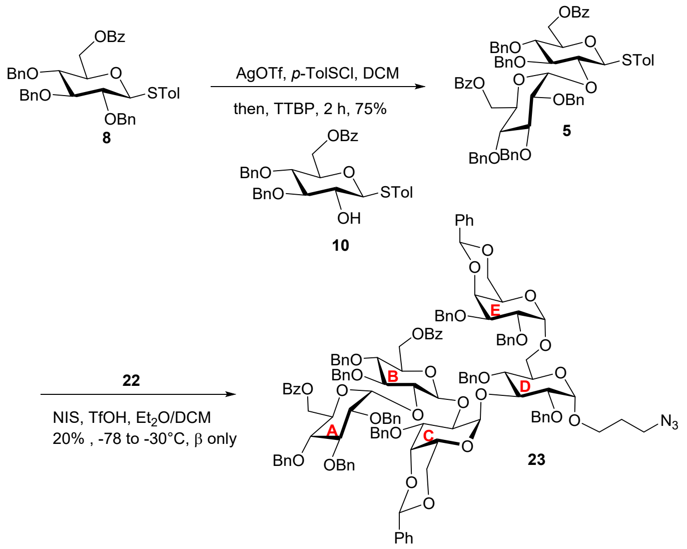

Scheme S5. Synthesis of pentasaccharide **23.**

4-Tolyl 6-*O*-benzoyl-2,3,4-tri-*O*-benzyl-α-D-glucopyranosyl-(1→2)- 6-*O*-benzoyl-3,4-di-*O*-benzyl-1-thio-β-D-galactopyranoside (5)

A solution of donor **8**[8] (55.3 mg, 83 μmol) and freshly activated molecular sieve MS 4Å (200 mg) in DCM (2 mL) was stirred for 5 minutes at room temperature, and then cooled to -78 °C. A solution of AgOTf (65 mg, 249 μmol) in anhydrous Et_2O/DCM (0.8 mL/0.2 mL) was added to reaction solution. After 5 min, orange colored *p*-TolSCl (13 μL, 83 μmol) was added to the reaction mixture through a microsyringe. The characteristic orange color of *p*-TolSCl in the reaction solution disappeared rapidly in a few seconds indicating depletion of *p*-TolSCl. After the donor was completely activated according to TLC analysis (about 5 minutes), a solution of acceptor **10** (43 mg, 75 μmol) with one equivalent of TTBP in DCM (1.2 mL) was slowly added via a syringe along the flask wall. The reaction was warmed up to -20 °C under stirring in 2 h. Upon reaction completion, the reaction mixture was quenched by Et_3N and filtered over Celite. The Celite was washed with DCM. After removal of the solvent, the desired oligosaccharide was purified by silica gel flash chromatography (5:1, hexanes/ethyl acetate) to afford **5** (62 mg, 75%) as colorless syrups. $[\alpha]_D^{20}$ +107 (c 0.1, $CHCl_3$); ^{1}H NMR (500 MHz, $CDCl_3$) δ 8.07-8.05 (m, 2H), 8.04-8.02 (m, 2H), 7.66-7.63 (m, 1H), 7.58-7.47 (m, 3H), 7.45-7.24 (m, 23H), 7.23-7.18 (m, 3H), 7.16-7.05 (m, 4H), 6.88 (d, J = 8.2 Hz, 2H), 6.05 (d, J = 3.8 Hz, 1H), 5.06 (d, J = 11.1, 1H), 5.05 (d, J = 11.6 Hz, 1H), 5.00 (d, J = 11.6 Hz, 1H), 4.92 (d, J = 11.0 Hz, 1H), 4.89-4.84 (m, 3H), 4.82 (d, J = 9.4 Hz, 1H),

4.80-4.74 (m, 1H), 4.70 (dd, J = 12.0, 2.1 Hz, 1H), 4.61 (d, J = 10.6 Hz, 1H), 4.52 (d, J = 11.0 Hz, 1H), 4.46 (dd, J = 12.0, 5.2 Hz, 1H), 4.38-4.36 (m, 1H), 4.31 (dd, J = 12.1, 2.1 Hz, 1H), 4.07 (t, J = 9.3 Hz, 1H), 3.95 (t, J = 9.2 Hz, 1H), 3.91 (dd, J = 12.1, 3.9 Hz, 1H), 3.85 (t, J = 8.9 Hz, 1H), 3.75-3.71 (m, 1H), 3.71-3.65 (m, 2H), 3.65-3.59 (m, 1H), 2.23 (s, 3H). ^{13}C NMR (125 MHz, $CDCl_3$) δ 166.25, 166.20, 138.61, 138.26, 138.07, 138.04, 137.90, 137.57, 137.29, 133.25, 133.03, 132.81, 130.24, 130.01, 129.95, 129.80, 129.76, 129.74, 128.67, 128.63, 128.62, 128.60, 128.55, 128.54, 128.53, 128.50, 128.43, 128.40, 128.36, 128.31, 128.28, 128.27, 128.22, 128.21, 128.19, 127.94, 127.92, 127.85, 127.70, 127.68, 127.53, 127.48, 95.29, 86.97, 84.88, 81.88, 80.04, 78.80, 77.80, 76.91, 76.04, 75.99, 75.39, 75.16, 74.28, 73.28, 68.96, 63.51, 63.26, 21.20. HRMS (ESI): *m/z* calcd for $C_{68}H_{66}O_{12}S$ $[M+NH_4]^+$: 1124.4619, found:1124.4634.

3-Azidopropyl 6-*O*-benzoyl-2,3,4-tri-*O*-benzyl-α-D-glucopyranosyl-(1→2)-6-*O*-benzoyl-3,4-di-*O*-benzyl-β-D-glucopyranosyl-(1→2)-3-*O*-benzyl-4,6-*O*-benzylidene-α-D-galactopyranosyl-(1→3)-2,4-di-*O*-benzyl-6-*O*-(2,3-di-*O*-benzyl-4,6-*O*-benzylidene-α-D-galactopyranosyl)-α-D-glucopyranoside (23)

A solution of donor **5** (55.3 mg, 50 μmol), acceptor **22** (51 mg, 42 μmol) and freshly activated molecular sieve MS 4Å (200 mg) in CH_2Cl_2 (DCM) (2 mL) was stirred for 5 minutes at room temperature, and then the suspension was cooled to -78 °C and then NIS (34 mg, 0.15 mmol) and TfOH (1.3 μL, 15 μmol) were added. The reaction mixture was gradually warmed to -10 °C and stirred for 3 h at the same temperature. Then, the mixture was quenched with triethylamine, diluted with CH_2Cl_2 and filtered. The filtrate was concentrated *in vacuo*. The residue was purified by flash chromatography (2:1, hexanes/ethyl acetate) to afford **23** (19 mg, 20%) as colorless syrups. $[\alpha]_D^{20}$ +83.9 (c 0.26, $CHCl_3$); ^{1}H NMR (500 MHz, $CDCl_3$) δ 7.98-7.93 (m, 4H), 7.53-7.50 (m, 3H), 7.36-7.25 (m, 29H), 7.24-7.08 (m, 32H), 7.00 (t, J = 7.6 Hz, 2H), 6.17 (d, J = 3.5 Hz, 1H), 5.80 (d, J = 3.6 Hz, 1H), 5.45 (s, 1H), 5.31 (s, 1H), 5.13 (d, J = 10.9 Hz, 1H), 5.01 (d, J = 7.8 Hz, 1H), 4.95-4.92 (m, 1H), 4.89 (d, J = 4.0 Hz, 1H), 4.86-4.82 (m, 2H), 4.81-4.72 (m, 6H), 4.72-4.62 (m, 5H), 4.52-4.48 (m, 2H), 4.46-4.38 (m, 6H), 4.38-4.34 (m, 2H), 4.33-4.30 (m, 1H), 4.19 (dd, J = 9.2, 3.5 Hz, 2H), 4.13 (d, J = 3.5 Hz, 1H), 4.11-4.06 (m, 2H), 4.05-4.01 (m, 3H), 3.96 (d, J = 3.7 Hz, 2H), 3.92 (dd, J = 10.1, 3.4 Hz, 1H), 3.86 (dd, J = 12.5, 1.8 Hz, 1H), 3.83-3.78 (m, 1H), 3.71-3.61 (m, 3H), 3.57-3.49 (m, 5H), 3.47-3.38 (m, 5H), 3.28-3.19 (m, 3H), 1.66-1.62 (m, 2H). ^{13}C NMR (125 MHz, $CDCl_3$) δ 166.24, 139.03, 138.96, 138.66, 138.50, 138.26, 138.38, 138.36, 138.2, 138.02, 137.96, 137.74, 137.61, 133.08, 129.96, 129.74, 128.61, 128.55, 128.51, 128.46, 128.45, 128.43, 128.40, 128.37, 128.35, 128.28, 128.26, 128.24, 128.17, 128.01, 127.99, 127.92, 127.90, 127.64, 127.63, 127.60, 126.49, 126.24, 107.11, 103.53, 101.16, 101.0, 98.43, 98.14, 96.22, 95.85, 94.44, 83.53, 81.73, 80.88, 80.33, 79.89, 79.27, 78.81, 77.36, 75.79, 75.67, 75.13, 73.10, 72.38, 71.80, 70.83, 70.19, 69.52, 64.50, 48.34, 28.65. HRMS (ESI): *m/z* calcd for $C_{131}H_{133}N_3O_{28}[M+NH_4]+$: 2213.9419, found: 2213.9460.

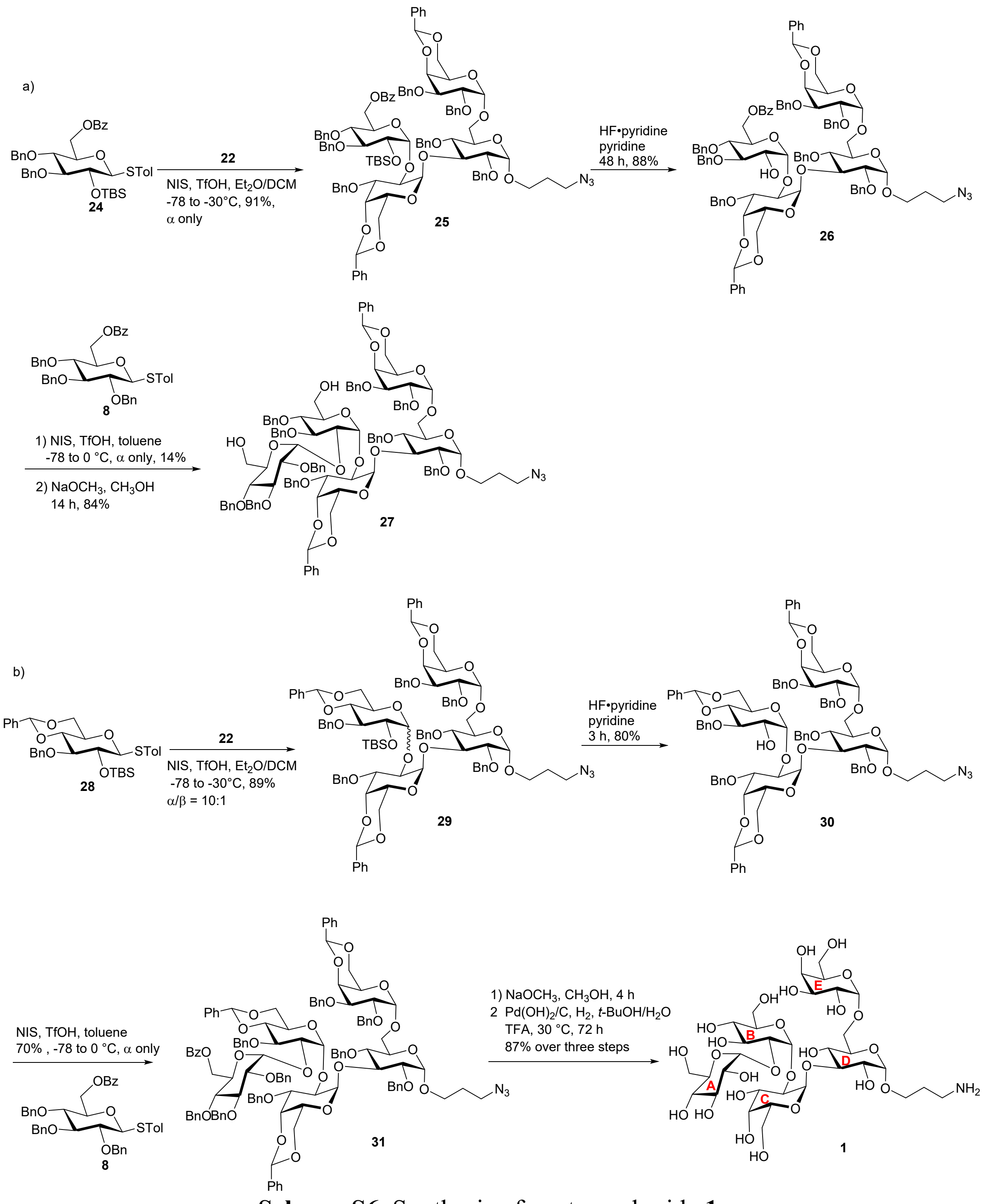

Scheme S6. Synthesis of pentasaccharide **1**.

3-Azidopropyl 6-*O*-benzoyl-3,4-di-*O*-benzyl-2-*O*-tert-butyldimethylsilyl-α-D-glucopyranosyl-(1→2)-3-*O*-benzyl-4,6-*O*-benzylidene-α-D-galactopyranosyl-(1→3)-2,4-di-*O*-benzyl-6-*O*-(2,3-di-*O*-benzyl-4,6-*O*-benzylidene-α-D-galactopyranosyl)-α-D-glucopyranoside (25)

A mixture of galactose donor **24** (273.6 mg, 0.4 mmol), acceptor **22** (400 mg, 0.33 mmol), and freshly activated 4 Å molecular sieves in CH_2Cl_2/Et_2O (v/v = 1:1, 6 mL) were stirred for 15 minutes at room temperature. The suspension was cooled to -78 °C and then NIS (135 mg, 0.6 mmol) and TfOH (4 μL, 0.04 mmol) were added. The reaction mixture was gradually warmed to -30 °C and stirred for 1 h at the same temperature. Then, the mixture was quenched with triethylamine, diluted with CH_2Cl_2 and filtered. The obtained residue was purified by silica gel column chromatography (5:1, hexanes/ethyl acetate) to afford **25** (532 mg, 91%) as colorless syrups. $[\alpha]_D^{20}$ +24.9 (*c* 0.1, $CHCl_3$); ^1H NMR (500 MHz, $CDCl_3$) δ 7.99 (d, *J* = 8.1 Hz, 2H), 7.52-7.47 (m, 5H), 7.41-7.34 (m, 12H), 7.33-7.27 (m, 14H), 7.25-7.20 (m, 10H), 7.19-7.12 (m, 7H), 5.85 (d, *J* = 3.3 Hz, 1H), 5.44 (s, 1H), 5.35 (s, 1H), 5.31 (d, *J* = 3.5 Hz, 1H), 5.13 (d, *J* = 11.5 Hz, 1H), 4.92 (d, *J* = 3.5 Hz, 1H), 4.79 (dd, *J* = 12.1, 4.1 Hz, 3H), 4.75 (d, *J* = 3.6 Hz, 1H), 4.71 (d, *J* = 3.7 Hz, 1H), 4.70 (d, *J* = 12.1 Hz, 1H), 4.66 (d, *J* = 12.1 Hz, 2H), 4.62-4.48 (m, 4H), 4.45 (d, *J* = 8.9 Hz, 3H), 4.39-4.30 (m, 5H), 4.15-4.09 (m, 3H), 4.08-4.00 (m, 4H), 3.91 (dd, *J* = 10.0, 3.5 Hz, 2H), 3.88-3.80 (m, 3H), 3.69 (dd, *J* = 9.5, 3.3 Hz, 1H), 3.65-3.60 (m, 2H), 3.58-3.53 (m, 3H), 3.43 (s, 1H), 3.39 (dd, *J* = 9.6, 3.6 Hz, 1H), 3.34-3.29 (m, 1H), 3.28-3.22 (m, 1H), 1.69 (s, 2H), 0.87 (s, 9H), 0.08 (s, 3H), 0.03 (s, 3H). ^{13}C NMR (125 MHz, $CDCl_3$) δ 166.44, 139.06, 138.93, 138.84, 138.31, 138.17, 138.12, 137.94, 130.08, 129.81, 129.00, 128.60, 128.59, 128.50, 128.41, 128.39, 128.32, 128.29, 128.24, 128.10, 128.08, 127.98, 127.93, 127.80, 127.77, 127.63, 127.60, 127.35, 126.50, 126.44, 101.13, 100.92, 98.83, 98.46, 97.53, 96.36, 82.68, 79.93, 79.43, 78.18, 75.82, 75.60, 75.35, 74.98, 74.73, 74.46, 73.29, 73.01, 72.06, 71.82, 69.97, 69.62, 64.53, 63.80, 62.61, 48.30, 28.81, 26.20, 18.25, -4.08, -4.45; HRMS (ESI): *m/z* calcd for $C_{103}H_{115}N_3O_{22}Si[M+ NH_4]^+$: 1791.8085, found: 1791.8093.

3-Azidopropyl 6-*O*-benzoyl-3,4-di-*O*-benzyl-α-D-glucopyranosyl-(1→2)-3-*O*-benzyl-4,6-*O*-benzylidene-α-D-galactopyranosyl-(1→3)-2,4-di-*O*-benzyl-6-*O*-(2,3-di-*O*-benzyl-4,6-*O*-benzylidene-α-D-galactopyranosyl)-α-D-glucopyranoside (26)

To a solution of **25** (550 mg, 0.31 mmol) in pyridine (3 mL), HF·pyridine (70% HF in pyridine, 0.3 mL) was added, and the solution was stirred for 48 h at room temperature. The reaction mixture was diluted with DCM, washed with saturated aqueous $NaHCO_3$, dried over Na_2SO_4, and filtered. The filtrate was concentrated *in vacuo* and the residue was purified by flash chromatography (4:1, hexanes/ethyl acetate) to afford **26** (453 mg, 88%) as a white solid. $[\alpha]_D^{20}$ +92.6 (*c* 0.34, $CHCl_3$); ^1H NMR (500 MHz, $CDCl_3$) δ 8.01-7.95 (m, 2H), 7.60-7.48 (m, 5H), 7.43-7.17 (m, 49H), 5.84 (d, *J* = 3.2 Hz, 1H), 5.46 (s, 1H), 5.45 (s, 1H), 5.07 (d, *J* = 11.1 Hz, 1H), 5.03 (d, *J* = 3.2 Hz, 1H), 5.02 (d, *J* = 3.1 Hz, 1H), 4.86-4.80 (m, 2H), 4.80-4.76 (m, 2H), 4.75 (d, *J* = 3.6 Hz, 1H), 4.74-4.68 (m, 5H), 4.67-4.48 (m, 8H), 4.39 (dt, *J* = 10.1, 2.7 Hz, 1H), 4.33 (dd, *J* = 9.4, 3.3 Hz, 1H), 4.29 (dd, *J* = 12.2, 2.1 Hz, 1H), 4.26-4.20 (m, 2H), 4.17-4.06 (m, 7H), 3.96-3.94 (m, 2H), 3.90 (dd, *J* = 12.2, 1.7 Hz, 1H), 3.79 (d, *J* = 12.3 Hz, 1H), 3.74-3.58 (m, 5H), 3.56-3.45 (m, 3H), 3.35-3.33 (m, 1H), 3.29-3.24 (m, 1H), 3.21-3.19 (m, 1H), 1.68 (p, *J* = 6.3 Hz, 2H). ^{13}C NMR (125 MHz, $CDCl_3$) δ 166.29, 139.09, 138.90, 138.71, 138.42, 138.37, 138.36, 138.27, 138.21, 138.09, 137.92, 132.94,

130.08, 129.75, 128.98, 128.91, 128.67, 128.63, 128.55, 128.48, 128.47, 128.44, 128.38, 128.36, 128.34, 128.24, 128.22, 128.15, 128.11, 128.07, 128.04, 128.01, 127.94, 127.79, 127.77, 127.74, 127.68, 127.61, 127.57, 127.53, 126.43, 126.34, 101.11, 100.86, 98.40, 96.80, 96.59, 96.38, 82.26, 80.28, 79.33, 78.76, 77.72, 75.81, 75.65, 75.60, 75.11, 74.98, 74.80, 74.69, 74.26, 73.82, 73.26, 73.02, 72.78, 72.54, 71.85, 71.38, 69.60, 69.47, 69.23, 69.00, 66.67, 64.59, 63.40, 62.77, 62.63, 48.19, 28.75. HRMS (ESI): *m/z* calcd for $C_{97}H_{101}N_3O_{22}[M+NH_4]^+$: 1677.7220, found:1677.7235.

3-Azidopropyl 2,3,4-tri-*O*-benzyl-α-D-glucopyranosyl-(1→2)-3,4-di-*O*-benzyl-α-D-glucopyranosyl-(1→2)-3-*O*-benzyl-4,6-*O*-benzylidene-α-D-galactopyranosyl-(1→3)-2,4-di-*O*-benzyl-6-*O*-(2,3-di-*O*-benzyl-4,6-*O*-benzylidene-α-D-galactopyranosyl)-α-D-glucopyranoside (27)

A mixture of galactose donor **8** (33 mg, 0.05 mmol), acceptor **26** (33 mg, 0.02 mmol), and freshly activated 4 Å molecular sieves in toluene (8 mL) was stirred for 15 minutes at room temperature. The suspension was cooled to -78 °C and then NIS (34 mg, 0.15 mmol) and TfOH (1.3 µL, 15 µmol) were added. The reaction mixture was gradually warmed to room temperature and stirred for 5 h at the same temperature. Then, the mixture was quenched with triethylamine, diluted with CH_2Cl_2 and filtered. The filtrate was concentrated *in vacuo*. The obtained residue was dissolved in MeOH/CH_2Cl_2 (v/v = 1:1, 2 mL). The solution was cooled to 0 °C and NaOMe (14 mg, 0.2 mmol) was added. After stirring overnight at room temperature, the mixture was neutralized with DOWEX-H^+ ion exchange resins, filtered and concentrated. The residue was purified by flash chromatography (2:1, hexanes/ethyl acetate) to afford **27** (5 mg, 12% over two steps) as colorless syrups. $[\alpha]_D^{20}$ +63.1 (*c* 0.26, $CHCl_3$); ^1H NMR (500 MHz, $CDCl_3$) δ 7.51-7.49 (m, 2H), 7.45-7.00 (m, 58H), 5.79 (d, *J* = 3.4 Hz, 1H), 5.45 (s, 1H), 5.42 (d, *J* = 3.5 Hz, 1H), 5.26 (s, 1H), 4.98 (d, *J* = 12.1 Hz, 1H), 4.94 (d, *J* = 3.5 Hz, 1H), 4.88 (d, *J* = 11.4 Hz, 1H), 4.85-4.81 (m, 3H), 4.79-4.74 (m, 2H), 4.71 (dd, *J* = 8.5, 5.0 Hz, 2H), 4.69-4.64 (m, 3H), 4.61-4.54 (m, 4H), 4.52 (d, *J* = 4.8 Hz, 1H), 4.48 (d, *J* = 15.8 Hz, 1H), 4.45-4.39 (m, 2H), 4.35 (dd, *J* = 10.4, 3.3 Hz, 1H), 4.23 (d, *J* = 10.7 Hz, 1H), 4.18-4.12 (m, 2H), 4.09-3.99 (m, 5H), 3.98-3.88 (m, 4H), 3.84 (t, *J* = 9.9 Hz, 2H), 3.77 (s, 1H), 3.71-3.52 (m, 11H), 3.49 (t, *J* = 9.5 Hz, 1H), 3.43-3.23 (m, 6H), 1.73-1.71 (m, 2H). ^{13}C NMR (200 MHz, $CDCl_3$) δ 139.06, 138.96, 138.84, 138.75, 138.66, 138.62, 138.37, 138.35, 137.93, 137.86, 137.29, 129.00, 128.74, 128.68, 128.65, 128.64, 128.61, 128.54, 128.51, 128.46, 128.44, 128.42, 128.39, 128.33, 128.27, 128.25, 128.18, 128.11, 128.08, 128.00, 127.94, 127.88, 127.81, 127.78, 127.71, 127.69, 127.65, 127.63, 127.60, 127.51, 127.22, 126.49, 126.45, 126.20, 126.15, 101.14, 100.69, 98.53, 96.43, 96.09, 93.56, 81.93, 81.82, 80.93, 79.93, 79.65, 77.86, 75.92, 75.75, 75.69, 75.53, 75.22, 74.89, 74.87, 74.70, 73.86, 73.27, 72.80, 72.74, 71.97, 71.71, 71.51, 71.49, 71.25, 69.57, 69.54, 69.31, 66.88, 64.57, 62.70, 62.19, 61.76, 61.71, 61.61, 52.11, 48.29, 28.77. HRMS (ESI): *m/z* calcd for $C_{117}H_{125}N_3O_{26}[M+NH_4]^+$: 2005.8895, found: 2005.8868.

3-Azidopropyl 3-*O*-benzyl-4,6-*O*-benzylidene-2-*O*-tert-butyldimethylsilyl-α-D-glucopyranosyl-(1→2)-3-*O*-benzyl-4,6-*O*-benzylidene-α-D-galactopyranosyl-(1→3)-2,4-di-

***O*-benzyl-6-*O*-(2,3-di-*O*-benzyl-4,6-*O*-benzylidene-α-D-galactopyranosyl)-α/β-D-glucopyranoside (29)**

A solution of donor **28** (125 mg, 0.22 mmol), acceptor **22** (218.4 mg, 0.18 mmol) and freshly activated molecular sieve MS 4Å (400 mg) in CH_2Cl_2 (DCM) (4 mL) was stirred for 5 minutes at room temperature. The suspension was cooled to -78 °C and then NIS (74 mg, 0. 33 mmol) and TfOH (2 µL, 22 µmol) were added. The reaction mixture was gradually warmed to -10 °C and stirred for 2 h at the same temperature. Then, the mixture was quenched with triethylamine, diluted with CH_2Cl_2 and filtered. The filtrate was concentrated *in vacuo*. The residue was purified by flash chromatography (2:1, hexanes/ethyl acetate) to afford **29** (267 mg, 89%) as α/β mixture. ^{1}H NMR (500 MHz, $CDCl_3$) δ 7.99 (d, *J* = 8.1 Hz, 2H), 7.52-7.47 (m, 5H), 7.41-7.34 (m, 12H), 7.33-7.27 (m, 14H), 7.25-7.20 (m, 10H), 7.19-7.12 (m, 7H), 5.85 (d, *J* = 3.3 Hz, 1H), 5.44 (s, 1H), 5.35 (s, 1H), 5.31 (d, *J* = 3.5 Hz, 1H), 5.13 (d, *J* = 11.5 Hz, 1H), 4.92 (d, *J* = 3.5 Hz, 1H), 4.79 (dd, *J* = 12.2, 4.1 Hz, 3H), 4.75 (d, *J* = 3.6 Hz, 1H), 4.72-4.68 (m, 2H), 4.66 (d, *J* = 12.2 Hz, 2H), 4.62-4.48 (m, 4H), 4.45 (d, *J* = 8.9 Hz, 3H), 4.39-4.30 (m, 5H), 4.15-4.09 (m, 3H), 4.08-4.00 (m, 4H), 3.91 (dd, *J* = 10.0, 3.5 Hz, 2H), 3.88-3.80 (m, 3H), 3.69 (dd, *J* = 9.5, 3.3 Hz, 1H), 3.65-3.60 (m, 2H), 3.58-3.53 (m, 3H), 3.43 (s, 1H), 3.39 (dd, *J* = 9.6, 3.6 Hz, 1H), 3.34-3.29 (m, 1H), 3.28-3.22 (m, 1H), 1.69 (s, 2H), 0.87 (s, 9H), 0.08 (s, 3H), 0.03 (s, 3H). ^{13}C NMR (125 MHz, $CDCl_3$) δ 166.44, 139.06, 138.93, 138.84, 138.31, 138.17, 138.12, 137.94, 130.08, 129.81, 129.00, 128.60, 128.59, 128.50, 128.41, 128.39, 128.32, 128.29, 128.24, 128.10, 128.08, 127.98, 127.93, 127.80, 127.77, 127.63, 127.60, 127.35, 126.50, 126.44, 101.13, 100.92, 98.46, 97.53, 96.36, 82.68, 79.93, 79.43, 78.18, 75.82, 75.60, 75.35, 74.98, 74.73, 74.46, 73.29, 73.01, 72.06, 71.82, 69.97, 69.62, 64.53, 63.80, 62.61, 48.30, 28.81, 26.20, 18.25, -4.08, -4.45. HRMS (ESI): *m*/*z* calcd for $C_{96}H_{109}N_3O_{21}Si[M+NH_4]^+$: 1685.7667, found: 1685.7706.

3-Azidopropyl 3-*O*-benzyl-4,6-*O*-benzylidene-α-D-glucopyranosyl (1→2)-3-*O*-benzyl-4,6-*O*-benzylidene-α-D-galactopyranosyl-(1→3)-2,4-di-*O*-benzyl-6-*O*-(2,3-di-*O*-benzyl-4,6-*O*-benzylidene-α-D-galactopyranosyl)-α-D-glucopyranoside (30)

To a solution of **29** (217 mg, 0.13 mmol) in pyridine (1.5 mL), HF·pyridine (70% HF in pyridine, 0.2 mL) was added. The solution was stirred for 3 h at room temperature. The reaction mixture was diluted with DCM, washed with saturated aqueous $NaHCO_3$, dried over Na_2SO_4, and filtered. The filtrate was concentrated *in vacuo* and the residue was purified by flash chromatography (4:1, hexanes/ethyl acetate) to afford **30** (162 mg, 80%) as a white solid. $[\alpha]_D^{20}$ +95.9 (*c* 0.18, $CHCl_3$); ^{1}H NMR (500 MHz, CDCl3) δ 7.63-7.58 (m, 4H), 7.53-7.47 (m, 8H), 7.45-7.35 (m, 21H), 7.35-7.26 (m, 11H), 7.23 (t, *J* = 7.3 Hz, 1H), 5.67 (d, *J* = 3.4 Hz, 1H), 5.55-5.48 (m, 3H), 5.22 (d, *J* = 3.4 Hz, 1H), 5.07 (d, *J* = 11.7 Hz, 1H), 4.92 (d, *J* = 3.9 Hz, 1H), 4.89-4.79 (m, 5H), 4.75 (d, *J* = 2.3 Hz, 1H), 4.75-4.69 (m, 3H), 4.62 (d, *J* = 11.7 Hz, 1H), 4.57 (d, *J* = 11.1 Hz, 1H), 4.46 (d, *J* = 11.1 Hz, 1H), 4.43-4.39 (m, 1H), 4.39-4.34 (m, 2H), 4.28-4.14 (m, 6H), 4.10-4.02 (m, 3H), 3.98 (d, *J* = 12.2 Hz, 1H), 3.90-3.73 (m, 6H), 3.71-3.59 (m, 4H), 3.54 (s, 1H), 3.48-3.41 (m, 3H), 3.36 (dd, *J* = 9.5, 3.7 Hz, 1H), 3.24 (d, *J* = 10.3 Hz, 1H), 1.94-1.84 (m, 2H).^{13}C NMR (125 MHz,

$CDCl_3$) δ 139.21, 138.98, 138.78, 138.09, 137.98, 137.88, 137.86, 137.80, 128.87, 128.82, 128.72, 128.61, 128.54, 128.49, 128.31, 128.29, 128.20, 128.16, 128.11, 128.02, 127.81, 127.72, 127.62, 127.54, 127.33, 127.21, 126.37, 126.23, 126.20, 101.05, 101.00, 100.69, 98.42, 96.80, 96.64, 96.27, 81.56, 80.12, 79.94, 78.66,76.43(C-3A), 75.76 (C-2B), 75.12, 74.72, 74.55, 74.03, 73.77, 73.27, 72.73, 72.53, 71.95, 71.80, 70.71, 70.07, 69.40, 69.37, 68.77, 66.00, 64.41, 62.66, 62.15, 48.17, 28.65. HRMS (ESI): *m*/*z* calcd for $C_{90}H_{95}N_3O_{21}[M+NH_4]^+$: 1571.6802, found:1571.6835.

3-Azidopropyl 6-*O*-benzoyl-2,3,4-tri-*O*-benzyl-α-D-glucopyranosyl-(1→2)-3-*O*-benzyl-4,6-*O*-benzylidene-α-D-glucopyranosyl-(1→2)-3-*O*-benzyl-4,6-*O*-benzylidene-α-D-galactopyranosyl-(1→3)-2,4-di-*O*-benzyl-6-*O*-(2,3-di-*O*-benzyl-4,6-*O*-benzylidene-α-D-galactopyranosyl)-α-D-glucopyranoside (31)

A mixture of galactose donor **8** (40 mg, 0.14 mmol), acceptor **30** (73 mg, 47 μmol), and freshly activated 4 Å molecular sieves in toluene (2 mL) were stirred for 15 minutes at room temperature. The suspension was cooled to -78 °C and then NIS (94.5 mg, 0.42 mmol) and TfOH (4 μL, 42 μmol) were added. The reaction mixture was gradually warmed to room temperature and stirred for 3 h at the same temperature. Then, the mixture was quenched with triethylamine, diluted with CH_2Cl_2 and filtered. The filtrate was concentrated *in vacuo*. The residue was purified by flash chromatography (3:1, hexanes/ethyl acetate) to afford **31** (68 mg, 70%) as colorless syrups. $[\alpha]_D^{20}$ +80.1 (*c* 0.12, $CHCl_3$); ^1H NMR (600 MHz, $CDCl_3$) δ 7.95 (d, *J* = 7.7 Hz, 2H), 7.56-7.52 (m, 3H), 7.47-7.45 (m, 4H), 7.41-7.23 (m, 40H), 7.22-7.09 (m, 14H), 7.07-7.05 (m, 2H), 5.86 (d, *J* = 3.3 Hz, 1H), 5.55 (s, 1H), 5.53 (d, *J* = 3.6 Hz, 1H), 5.46 (s, 1H), 5.27 (s, 1H), 5.09 (d, *J* = 11.4 Hz, 2H), 4.93-4.77 (m, 6H), 4.7-4.63 (m, 6H), 4.62-4.55 (m, 3H), 4.55-4.46 (m, 3H), 4.41-4.33 (m, 3H), 4.29-4.21 (m, 4H), 4.19-4.01 (m, 8H), 4.00-3.87 (m, 4H), 3.84-3.75 (m, 2H), 3.74-3.65 (m, 2H), 3.56-3.54 (m, 6H), 3.49-3.22 (m, 6H), 1.77-1.64 (m, 2H). ^{13}C NMR (150 MHz, $CDCl_3$) δ 166.13, 139.02, 138.96, 138.82, 138.70, 138.36, 138.24, 138.14, 138.01, 137.93, 137.77, 137.70, 133.03, 130.13, 129.79, 129.03, 128.97, 128.70, 128.65, 128.62, 128.57, 128.48, 128.41, 128.32, 128.28, 128.23, 128.17, 128.10, 128.04, 127.98, 127.77, 127.72, 127.62, 127.59, 126.47, 126.25, 126.07, 101.43, 101.12, 100.61, 98.36, 96.86, 96.04, 95.53, 94.67, 82.79, 82.16, 80.48, 79.37, 78.84, 77.75, 76.76, 75.89, 75.86, 75.73, 75.56, 75.05, 74.88, 74.79, 73.75, 73.24, 72.70, 72.27, 71.83, 71.57, 69.85, 69.53, 69.31, 69.28, 69.08, 67.16, 64.45, 63.43, 62.90, 62.67, 62.04, 48.31, 28.79. HRMS (ESI): *m*/*z* calcd for $C_{124}H_{127}N_3O_{27}[M+NH_4]^+$: 2107.9001, found:2107.8940.

3-Aminopropyl α-D-glucopyranosyl-(1→2)-α-D-glucopyranosyl-(1→2)-α-D-galactopyranosyl-(1→3)-3-*O*-(α-D-galactopyranosyl)-α-D-glucopyranoside (1)

To a solution of **31** (50 mg, 24 μmol) in MeOH/DCM (5 mL, v/v = 1:1) was added NaOMe (13 mg, 0.19 mmol) at 0 °C, and the resulting mixture was warmed gradually to room temperature. The mixture was stirred for 12 h at the same temperature, at the end of which time TLC indicated the reaction was finished. The reaction was quenched with Amberlite IR120 H+ resin. After

filtration, the resulting mixture was concentrated to dryness. The obtained residue was purified by silica gel column chromatography (3:1, hexanes/ethyl acetate) to afford a white solid. To a solution of the obtained solid in *t*-BuOH/H_2O/TFA (5 mL, v/v/v = 4:1:0.04) was added 20% $Pd(OH)_2/C$ (100 mg), and the reaction mixture was stirred under a hydrogen atmosphere at 30 °C. The mixture was stirred for 72 h. The reaction mixture was filtered and the filtrate was concentrated *in vacuo*. The obtained residue was purified by Sephadex G-10 column (H_2O) to afford **1** (18 mg, 87% over two steps) as a white solid. $[\alpha]_D^{20}$ +11.6 (*c* 0.1, H_2O); ^1H NMR (800 MHz, D_2O) δ 5.69 (d, *J* = 3.8 Hz, 1H), 5.39 (d, *J* = 3.5 Hz, 1H), 5.10 (d, *J* = 3.8 Hz, 1H), 4.94 (d, *J* = 2.2 Hz, 1H), 4.92 (d, *J* = 3.8 Hz, 1H), 4.23 (t, *J* = 6.4 Hz, 1H), 4.03 (dd, *J* = 10.3, 3.5 Hz, 1H), 3.99 (d, *J* = 3.3 Hz, 1H), 3.97-3.78 (m, 14H), 3.77-3.67 (m, 11H), 3.61-3.56 (m, 1H), 3.51 (dd, *J* = 9.9, 3.7 Hz, 1H), 3.47 (t, *J* = 9.5 Hz, 1H), 3.41 (t, *J* = 9.7 Hz, 1H), 3.15 (dt, *J* = 13.5, 6.8 Hz, 1H), 3.10 (dt, *J* = 12.9, 7.2 Hz, 1H), 2.02-1.94 (m, 2H). ^{13}C NMR (200 MHz, D_2O) δ 98.31, 98.16, 95.81, 95.19, 92.80, 77.14, 74.61, 72.81, 72.50, 71.81, 71.43, 71.02, 71.00, 70.58, 70.37, 70.34, 69.42, 69.35, 69.33, 69.18, 69.11, 68.19, 67.58, 65.94, 65.76, 61.12, 61.02, 60.28, 60.16, 37.79, 26.43; HRMS (ESI): *m*/*z* calcd for $C_{33}H_{59}NO_{26}[M+H]^+$: 886.3404, found:886.3397.

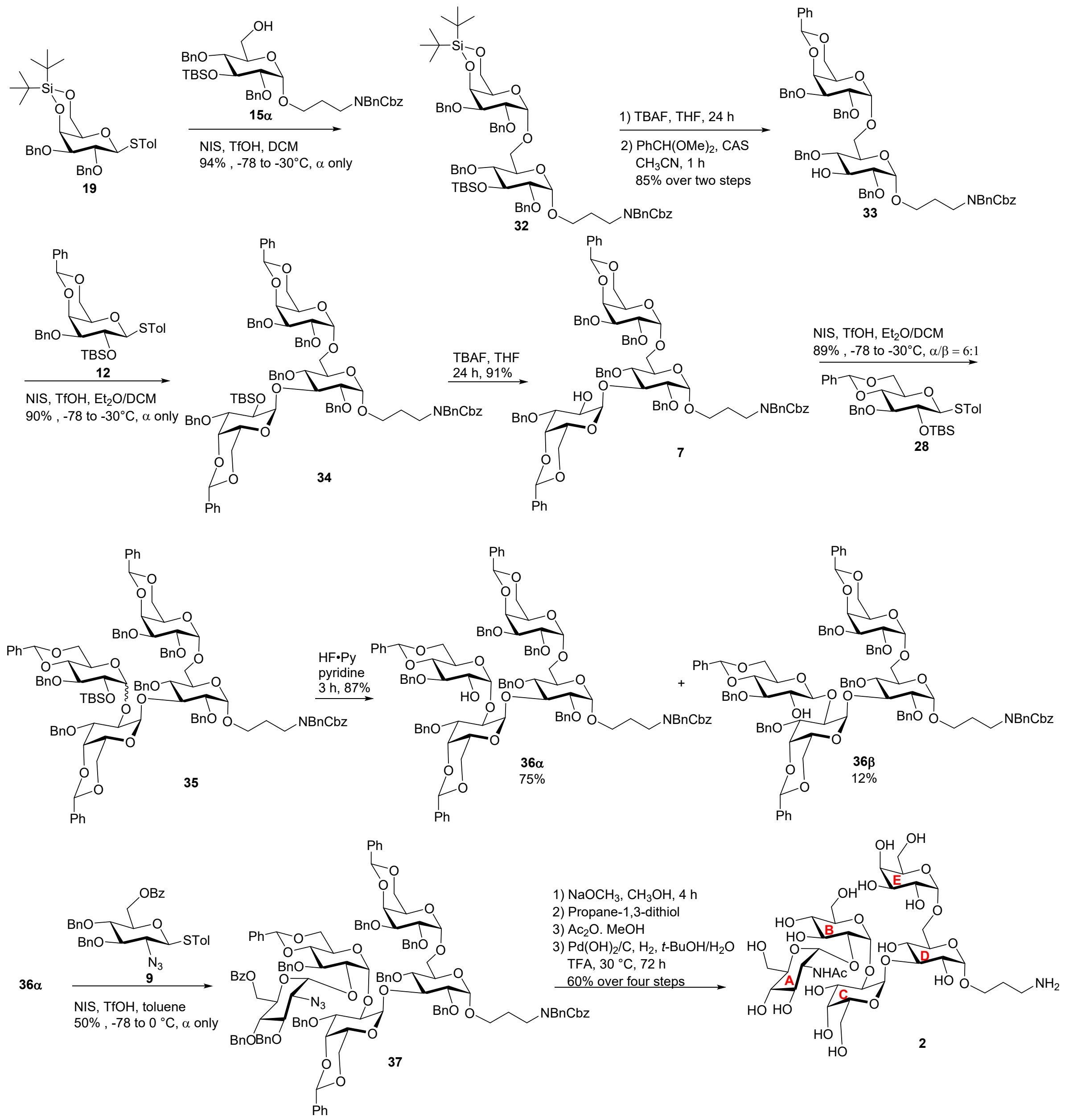

Scheme S7. Synthesis of pentasaccharide **2**.

N-(Benzyl)benzyloxycarbonyl-3-aminopropyl 2,3-di-*O*-benzyl-4,6-*O*-di-tert-butylsilanediyl-α-D-galactopyranosyl-(1→6)-2,4-di-*O*-benzyl-3-*O*-tert-butyldimethylsilyl-α-D-glucopyranoside (32)

A mixture of galactose donor **19** (300 mg, 0.49 mmol), acceptor **15α** (336 mg, 0.45 mmol), and freshly activated 4 Å molecular sieves in CH_2Cl_2 (4 mL) were stirred for 15 minutes at room temperature. The suspension was cooled to -78 °C and then NIS (165 mg, 0.74 mmol) and TfOH (5 μL, 49 μmol) were added. The reaction mixture was gradually warmed to -30 °C and stirred for 1 h at the same temperature. Then, the mixture was quenched with triethylamine, diluted with

CH_2Cl_2 and filtered. The filtrate was concentrated *in vacuo*. The obtained residue was purified by silica gel column chromatography (8:1, hexanes/ethyl acetate) to afford **32** (529 mg, 94%) as colorless syrups. $[\alpha]_D^{20}$ +64 (*c* 0.25, $CHCl_3$); ^1H NMR (500 MHz, $CDCl_3$) δ 7.44-7.18 (m, 30H), 5.20 (d, *J* = 10.7 Hz, 2H), 5.00 (d, *J* = 3.5 Hz, 1H), 4.92 (d, *J* = 11.8 Hz, 1H), 4.74 (d, *J* = 13.7 Hz, 4H), 4.60 (d, *J* = 11.8 Hz, 1H), 4.56 (d, *J* = 11.8 Hz, 2H), 4.53-4.47 (m, 2H), 4.44 (s, 1H), 4.39-4.32 (m, 1H), 4.07-3.97 (m, 4H), 3.81-3.71 (m, 2H), 3.71-3.63 (m, 2H), 3.62-3.50 (m, 2H), 3.43 (s, 1H), 3.39-3.11 (m, 4H), 1.87-1.73 (m, 2H), 1.07 (s, 9H), 1.01 (s, 9H), 0.97 (s, 9H), 0.11-0.08 (m, 3H), 0.06 (s, 3H). ^{13}C NMR (125 MHz, $CDCl_3$) δ 156.76, 156.20, 138.99, 138.89, 138.65, 138.03, 136.98, 128.67, 128.63, 128.54, 128.40, 128.38, 128.36, 128.35, 128.14, 128.04, 127.97, 127.77, 127.72, 127.55, 127.52, 127.48, 127.39, 127.12, 98.22, 97.02, 80.78, 79.47, 77.16, 76.61, 74.95, 74.42, 74.03, 73.33, 72.44, 71.18, 70.75, 70.63, 67.25, 67.21, 66.05, 65.90, 65.37, 65.18, 51.07, 50.75, 44.79, 43.86, 28.46, 27.89, 27.77, 27.43, 26.22, 23.50, 20.76, 18.27, -3.82, -4.16; (28.46 and 27.89 are from the same carbon atoms due to rotamers; 44.79 and 43.86 are from the same carbon atoms due to rotamers; 51.07 and 50.75 are from the same carbon atoms due to rotamers; 65.37 and 65.18 are from the same carbon atoms due to rotamers; 66.05 and 65.90 are from the same carbon atoms due to rotamers; 156.76 and 156.20 are from the same carbon atoms due to rotamers); HRMS (ESI): *m*/*z* calcd for $C_{72}H_{95}NO_{13}Si_2[M+NH_4]^+$: 1255.6686, found:1255.6725.

***N*-(Benzyl)benzyloxycarbonyl-3-aminopropyl 2,3-di-*O*-benzyl-4,6-*O*-benzylidene-α-D-galactopyranosyl-(1→6)-2,4-di-*O*-benzyl-α-D-glucopyranoside (33)**

A solution of **32** (260 mg, 0.21 mmol) in pyridine (2 mL) and HF·pyridine (70% HF in pyridine, 0.2 mL) was stirred for 3 h at room temperature. The reaction mixture was diluted with DCM, washed with saturated aqueous $NaHCO_3$, dried over Na_2SO_4, and filtered. The filtrate was concentrated *in vacuo* and the residue was purified by flash chromatography (DCM/MeOH, 40:1). The obtained product was dissolved in CH_3CN and camphorsulfonic acid (CSA) (24 mg, 0.1 mmol) and $PhCH(OMe)_2$ (63 μL, 0.42 mmol) were added. After stirring for 1 hour at room temperature, the reaction mixture was diluted with DCM, washed with saturated aqueous $NaHCO_3$, dried over Na_2SO_4, and filtered. The solvent was removed under reduced pressure, and the residue was purified by flash chromatography (3:1, hexanes/ethyl acetate) to yield compound **33** as a white solid (191 mg, 85% yield over two steps). $[\alpha]_D^{20}$ +37.1 (*c* 0.19, $CHCl_3$); ^1H NMR (500 MHz, $CDCl_3$) δ 7.55-7.49 (m, 2H), 7.47-7.08 (m, 33H), 5.45 (s, 1H), 5.18 (d, *J* = 10.5 Hz, 2H), 5.10 (d, *J* = 3.6 Hz, 1H), 4.93 (d, *J* = 11.7 Hz, 1H), 4.80 (d, *J* = 12.2 Hz, 1H), 4.79 (d, *J* = 12.2 Hz, 1H), 4.75 (d, *J* = 12.2 Hz, 1H), 4.70 (d, *J* = 12.2 Hz, 1H), 4.59 (d, *J* = 11.7 Hz, 1H), 4.63-4.42 (m, 5H), 4.16-4.00 (m, 4H), 3.96 (dd, *J* = 10.1, 3.4 Hz, 1H), 3.84-3.80 (m, 2H), 3.78-3.65 (m, 2H), 3.65-3.52 (m, 2H), 3.48 (d, *J* = 10.1 Hz, 1H), 3.35-3.33 (m, 2H), 3.23 (t, *J* = 11.4 Hz, 2H), 2.35 (s, 1H), 1.90-1.68 (m, 2H). ^{13}C NMR (125 MHz, $CDCl_3$) δ 139.10, 138.86, 138.18, 137.98, 128.97, 128.67, 128.62, 128.52, 128.43, 128.40, 128.22, 128.08, 128.00, 127.93, 127.83, 127.71, 127.67, 127.52, 127.38, 126.48, 101.16, 98.50, 96.27, 80.01, 77.67, 75.77, 75.31, 74.87, 74.33, 73.43, 72.83, 72.02, 70.22, 69.46, 67.31, 66.42, 65.56, 62.72, 50.99, 50.68, 44.78, 43.85, 28.44, 27.93. (28.44 and 27.93

are from the same carbon atoms due to rotamers; 44.78 and 43.85are from the same carbon atoms due to rotamers; 50.99 and 50.68 are from the same carbon atoms due to rotamers); HRMS (ESI): *m*/*z* calcd for $C_{65}H_{69}NO_{13}[M+NH_4]^+$: 1089.5113, found: 1089.5146.

_N_-(Benzyl)benzyloxycarbonyl-3-aminopropyl 2-_O_-tert-butyldimethylsilyl-3-_O_-benzyl-4,6-_O_-benzylidene-α-D-galactopyranosyl-(1→3)-2,4-di-_O_-benzyl-6-_O_-(2,3-di-_O_-benzyl-4,6-_O_-benzylidene-α-D-galactopyranosyl)-α-D-glucopyranoside (34)

A mixture of galactose donor **12** (200 mg, 0.35 mmol), acceptor **33** (334 mg, 0.32 mmol), and freshly activated 4 Å molecular sieves in CH_2Cl_2 (3 mL) were stirred for 15 minutes at room temperature. The suspension was cooled to -78 °C and then NIS (165 mg, 0.74 mmol) and TfOH (5 μL, 49 μmol) were added. The reaction mixture was gradually warmed to -30 °C and stirred for 1 h at the same temperature. Then, the mixture was quenched with triethylamine, diluted with CH_2Cl_2 and filtered. The filtrate was concentrated *in vacuo*. The obtained residue was purified by silica gel column chromatography (5:1, hexanes/ethyl acetate) to afford **34** (433 mg, 90%) as white solid. $[\alpha]_D^{20}$ = +88.9 (*c* 0.31, $CHCl_3$); ^1H NMR (500 MHz, $CDCl_3$) δ 7.59-7.52 (m, 4H), 7.46-7.44 (m, 6H), 7.42-7.36 (m, 9H), 7.36-7.30 (m, 14H), 7.29-7.24 (m, 9H), 7.20-7.11 (m, 3H), 5.54 (d, *J* = 3.3 Hz, 1H), 5.49 (s, 1H), 5.30 (s, 1H), 5.21 (d, *J* = 12.1 Hz, 2H), 5.12 (s, 1H), 5.09-5.03 (m, 1H), 4.90-4.80 (m, 2H), 4.76 (dd, *J* = 12.4, 3.8 Hz, 2H), 4.73-4.59 (m, 5H), 4.58-4.40 (m, 3H), 4.36-4.24 (m, 4H), 4.16 (dt, *J* = 7.3, 3.0 Hz, 1H), 4.12-4.08 (m, 2H), 4.07-3.96 (m, 3H), 3.94-3.79 (m, 5H), 3.78-3.71 (m, 2H), 3.69-3.55 (m, 2H), 3.48 (t, *J* = 11.4 Hz, 2H), 3.44-3.24 (m, 4H), 1.89-1.75 (m, 2H), 0.91 (s, 9H), 0.04 (s, 3H), 0.00 (s, 3H). ^{13}C NMR (125 MHz, $CDCl_3$) δ 156.67, 156.19, 139.22, 139.01, 138.52, 138.32, 138.19, 137.94, 136.78, 128.94, 128.71, 128.64, 128.53, 128.48, 128.44, 128.38, 128.27, 128.18, 128.15, 128.07, 127.94, 127.73, 127.67, 127.60, 127.53, 127.45, 127.40, 127.32, 127.27, 126.96, 126.44, 126.21, 101.11, 100.38, 99.14, 98.49, 96.18, 79.49, 79.13, 75.80, 75.78, 75.65, 74.94, 74.04, 73.75, 72.93, 72.64, 72.26, 71.32, 69.82, 69.53, 69.42, 67.27, 66.02, 65.43, 62.65, 62.45, 51.19, 50.78, 44.93, 44.04, 28.42, 27.90, 26.15,18.47, -4.14, -4.39. (28.42 and 27.90 are from the same carbon atoms due to rotamers; 44.93 and 44.04 are from the same carbon atoms due to rotamers; 51.19 and 50.78 are from the same carbon atoms due to rotamers; 156.67 and 156.19 are from the same carbon atoms due to rotamers); HRMS (ESI): *m*/*z* calcd for $C_{91}H_{103}NO_{18}Si[M+NH_4]^+$: 1543.7288, found: 1543.7324.

_N_-(Benzyl)benzyloxycarbonyl-3-aminopropyl 3-_O_-benzyl-4,6-_O_-benzylidene-α-D-galactopyranosyl-(1→3)-2,4-di-_O_-benzyl-6-_O_-(2,3-di-_O_-benzyl-4,6-_O_-benzylidene-α-D-galactopyranosyl)-α-D-glucopyranoside (7)

A solution of **34** (287 mg, 0.19 mmol) in THF (2 mL) and TBAF (0.2 mL) was added. After stirring 2 h at room temperature, the mixture was concentrated *in vacuo*. The obtained residue was purified by silica gel column chromatography (2:1, hexanes/ethyl acetate) to afford **7** (244 mg, 91%) as colorless syrups. $[\alpha]_D^{20}$ = +79.6 (*c* 0.61, $CHCl_3$); ^1H NMR (500 MHz, $CDCl_3$) δ 7.68-6.92 (m, 45H), 5.58 (d, *J* = 3.8 Hz, 1H), 5.47 (s, 1H), 5.25 (s, 1H), 5.19-5.15 (m, 3H), 5.01 (d, *J* = 10.8 Hz,

1H), 4.82-4.71 (m, 7H), 4.57 (d, *J* = 10.8 Hz, 1H), 4.54- 4.36 (m, 3H), 4.17 (td, *J* = 13.0, 5.9 Hz, 5H), 4.08 (dd, *J* = 10.1, 3.5 Hz, 1H), 3.96 (dd, *J* = 10.1, 3.5 Hz, 1H), 3.92 (d, *J* = 12.3 Hz, 1H), 3.89-3.80 (m, 4H), 3.78-3.69 (m, 3H), 3.66 (q, *J* = 6.4 Hz, 2H), 3.52 (s, 1H), 3.37-3.18 (m, 4H), 2.33 (s, 1H), 1.79-1.61 (m, 2H). ^{13}C NMR (125 MHz, $CDCl_3$) δ 156.73, 156.26, 139.29, 138.79, 138.55, 138.30, 138.09, 137.96, 136.82, 129.00, 128.82, 128.76, 128.65, 128.59, 128.56, 128.51, 128.49, 128.45, 128.42, 128.24, 128.21, 128.16, 128.14, 127.98, 127.96, 127.92, 127.89, 127.80, 127.75, 127.45, 127.39, 126.49, 126.29, 101.20, 100.69, 98.78, 96.11, 79.35, 78.98, 76.66, 75.85, 75.27, 75.18, 74.83, 74.36, 73.45, 72.56, 72.06, 71.13, 70.77, 69.51, 69.45, 68.04, 67.32, 66.19, 65.52, 65.38, 62.90, 62.40, 51.23, 50.84, 44.96, 44.03, 28.48, 27.94. (28.48 and 27.94 are from the same carbon atoms due to rotamers; 44.96 and 44.03 are from the same carbon atoms due to rotamers; 51.23 and 50.84 are from the same carbon atoms due to rotamers; 156.73 and 156.26 are from the same carbon atoms due to rotamers); HRMS (ESI): *m/z* calcd for $C_{85}H_{89}NO_{18}[M+NH_4]^+$: 1429.6423, found: 1429.6450.

_N_-(Benzyl)benzyloxycarbonyl-3-aminopropyl 2-_O_-tert-butyldimethylsilyl-3-_O_-benzyl-4,6-_O_-benzylidene-α/β-D-glucopyranosyl-3-_O_-benzyl-4,6-_O_-benzylidene-α-D-galactopyranosyl-(1→3)-2,4-di-_O_-benzyl-6-_O_-(2,3-di-_O_-benzyl-4,6-_O_-benzylidene-α-D-galactopyranosyl)-α-D-glucopyranoside (35)

A mixture of galactose donor **28** (480 mg, 0.81 mmol), acceptor **7** (1.03 g, 0.73 mmol), and freshly activated 4 Å molecular sieves in CH_2Cl_2 (13 mL) were stirred for 15 minutes at room temperature. The suspension was cooled to -78 °C and then NIS (273 mg, 1.22 mmol) and TfOH (8 μL, 81 μmol) were added. The reaction mixture was gradually warmed to -20 °C and stirred for 2 h at the same temperature. Then, the mixture was quenched with triethylamine, diluted with CH_2Cl_2 and filtered. The filtrate was concentrated *in vacuo*. The obtained residue was purified by silica gel column chromatography (3:1, hexanes/ethyl acetate) to afford an inseparable mixture of α/β isomers **35** (1.2 g, α/β = 6:1, 89%) as colorless syrup. Selected analytical data for α-isomer of **35**: ^{1}H NMR (500 MHz, $CDCl_3$) δ 5.81 (d, *J* = 3.3 Hz, 1H), 5.53 (s, 1H), 5.43 (s, 1H), 5.36 (d, *J* = 3.7 Hz, 1H), 5.30 (s, 1H), 0.86 (s, 9H), 0.04 (s, 3H), -0.02 (s, 3H); ^{13}C NMR (125 MHz, $CDCl_3$) δ 101.22, 101.04, 100.78, 98.47, 51.18, 50.77, 44.99, 44.08, 28.40, 27.88, 26.11, 18.27, -4.37, -4.49. (28.40 and 27.88 are from the same carbon atoms due to rotamers; 44.99 and 44.08 are from the same carbon atoms due to rotamers; 51.18 and 50.77 are from the same carbon atoms due to rotamers are from the same carbon atoms due to rotamers); Selected analytical data for β-isomer of **35**: ^{1}H NMR (500 MHz, $CDCl_3$) δ 5.62 (s, 1H), 5.47 (s, 1H), 5.29 (s, 1H), 0.96 (s, 9H), 0.33 (s, 3H), 0.18 (s, 3H). ^{13}C NMR (125 MHz, $CDCl_3$) δ 26.29, 18.51, -3.21, -4.09; HRMS (ESI): *m/z* calcd for $C_{111}H_{123}NO_{23}Si[M+NH_4]^+$: 1883.8599, found: 1883.8611.

_N_-(Benzyl)benzyloxycarbonyl-3-aminopropyl 3-_O_-benzyl-4,6-_O_-benzylidene-α-D-glucopyranosyl-3-_O_-benzyl-4,6-_O_-benzylidene-α-D-galactopyranosyl-(1→3)-2,4-di-_O_-

benzyl-6-*O*-(2,3-di-*O*-benzyl-4,6-*O*-benzylidene-α-D-galactopyranosyl)-α-D-glucopyranoside (36α)

A solution of **35** (392 mg, 0.21 mmol) in pyridine (2 mL) and HF·pyridine (70% HF in pyridine, 0.2 mL) was stirred for 3 h at room temperature. The reaction mixture was diluted with DCM, washed with saturated aqueous $NaHCO_3$ solution, dried over Na_2SO_4, and filtered. The filtrate was concentrated *in vacuo* and the residue was purified by flash chromatography (DCM/MeOH, 40:1) to afford **36α** (274 mg, 75%) as white solid. $[\alpha]_D^{20}$ +104 (*c* 0.04, $CHCl_3$); ^{1}H NMR (500 MHz, $CDCl_3$) δ 7.59-7.56 (m, 4H), 7.52-6.99 (m, 51H), 5.64 (d, *J* = 3.3 Hz, 1H), 5.52 (s, 1H), 5.48 (s, 1H), 5.46 (s, 1H), 5.28-5.16 (m, 3H), 5.04 (d, *J* = 12.0 Hz, 1H), 4.88-4.77 (m, 6H), 4.71 (dd, *J* = 12.0, 4.3 Hz, 3H), 4.66-4.45 (m, 5H), 4.38-4.30 (m, 3H), 4.21-4.12 (m, 6H), 4.03 (dt, *J* = 10.4, 5.2 Hz, 3H), 3.95 (dd, *J* = 12.5, 1.8 Hz, 1H), 3.89-3.79 (m, 3H), 3.78-3.50 (m, 8H), 3.48-3.27 (m, 4H), 3.16 (d, *J* = 8.3 Hz, 1H), 1.92-1.78 (m, 2H). ^{13}C NMR (125 MHz, $CDCl_3$) δ 156.19, 139.31, 139.04, 138.88, 138.21, 138.15, 138.07, 137.94, 137.86, 128.91, 128.85, 128.76, 128.68, 128.64, 128.60, 128.54, 128.53, 128.34, 128.21, 128.16, 128.07, 127.91, 127.82, 127.79, 127.75, 127.65, 127.56, 127.37, 127.36, 127.27, 127.18, 126.42, 126.28, 126.24, 101.12, 101.05, 100.69, 98.48, 96.64, 96.09, 81.65, 79.99, 78.79, 75.82, 75.18, 74.81, 74.60, 73.87, 73.38, 73.10, 72.75, 72.52, 71.88, 70.80, 69.98, 69.44, 68.84, 67.25, 66.10, 65.51, 62.73, 62.65, 62.19, 50.67, 44.84, 28.38. HRMS (ESI): *m*/*z* calcd for $C_{105}H_{109}NO_{23}[M+NH_4]^+$: 1769.7734, found:1769.7737.

***N*-(Benzyl)benzyloxycarbonyl-3-aminopropyl 3-*O*-benzyl-4,6-*O*-benzylidene-β-D-glucopyranosyl-3-*O*-benzyl-4,6-*O*-benzylidene-α-D-galactopyranosyl-(1→3)-2,4-di-*O*-benzyl-6-*O*-(2,3-di-*O*-benzyl-4,6-*O*-benzylidene-α-D-galactopyranosyl)-α-D-glucopyranoside (36β)**

A solution of **35** (392 mg, 0.21 mmol) in pyridine (2 mL) and HF·pyridine (70% HF in pyridine, 0.2 mL) was stirred for 3 h at room temperature. The reaction mixture was diluted with DCM, washed with saturated aqueous $NaHCO_3$ solution, dried over Na_2SO_4, and filtered. The filtrate was concentrated *in vacuo* and the residue was purified by flash chromatography (DCM/MeOH, 40:1) to afford **36β** (46 mg, 12%) as white solid. $[\alpha]_D^{20}$ +33.3 (*c* 0.12, $CHCl_3$); ^{1}H NMR (500 MHz, $CDCl_3$) δ 7.55-7.47 (m, 6H), 7.46-7.32 (m, 28H), 7.32-7.26 (m, 12H), 7.26-7.11 (m, 9H), 5.61 (s, 1H), 5.46 (s, 1H), 5.33-5.07 (m, 6H), 4.92-4.68 (m, 10H), 4.58-4.43 (m, 4H), 4.28 (d, *J* = 10.6 Hz, 1H), 4.24-4.13 (m, 3H), 4.12-4.01 (m, 4H), 3.96 (dd, *J* = 10.1, 3.4 Hz, 1H), 3.91-3.83 (m, 3H), 3.82-3.61 (m, 6H), 3.62-3.54 (m, 3H), 3.44-3.29 (m, 4H), 3.27-3.20 (m, 2H), 3.12 (d, *J* = 12.7 Hz, 1H), 2.93 (s, 1H), 2.78 (t, *J* = 9.9 Hz, 1H), 1.85-1.80 (m, 2H). ^{13}C NMR (125 MHz, $CDCl_3$) δ 139.25, 138.67, 138.20, 137.94, 137.44, 128.98, 128.89, 128.75, 128.63, 128.60, 128.52, 128.48, 128.46, 128.40, 128.29, 128.22, 128.18, 128.17, 128.09, 128.07, 127.98, 127.81, 127.74, 127.71, 127.54, 127.41, 127.10, 126.47, 126.33, 126.31, 126.08, 104.65, 101.17, 100.86, 100.83, 98.83, 98.74, 96.00, 80.86, 80.47, 79.05, 75.78, 75.57, 75.42, 75.16, 74.79, 74.60, 74.20, 73.88, 72.71, 72.04, 71.38, 69.43, 68.05, 67.30, 65.98, 62.69, 62.00, 51.26, 45.11, 27.93. HRMS (ESI): *m*/*z* calcd for $C_{105}H_{109}NO_{23}[M+NH_4]^+$: 1769.7734, found: 1769.7749.

_N_-(Benzyl)benzyloxycarbonyl-3-aminopropyl 2-azido-6-_O_-benzoyl-3,4-di-_O_-benzyl-2-deoxy-α-D-glucopyranosyl-3-_O_-benzyl-4,6-_O_-benzylidene-α-D-glucopyranosyl-3-_O_-benzyl-4,6-_O_-benzylidene-α-D-galactopyranosyl-(1→3)-2,4-di-_O_-benzyl-6-_O_-(2,3-di-_O_-benzyl-4,6-_O_-benzylidene-α-D-galactopyranosyl)-α-D-glucopyranoside (37)

A mixture of donor **9**[9] (100 mg, 0.17 mmol), acceptor **36α** (59 mg, 33 μmol), and freshly activated 4 Å molecular sieves in toluene (3 mL) was stirred for 15 minutes at room temperature. The suspension was cooled to -78 °C and then NIS (191 mg, 0.85 mmol) and TfOH (8 μL, 85 μmol) were added. The reaction mixture was gradually warmed to room temperature and stirred for 5 h at the same temperature. Then, the mixture was quenched with triethylamine, diluted with CH_2Cl_2 and filtered. The filtrate was concentrated *in vacuo*. The obtained residue was purified by silica gel column chromatography (2:1, hexanes/ethyl acetate) to afford **37** (37 mg, 50%) as white solid. $[\alpha]_D^{20}$ +37.6 (*c* 0.1, $CHCl_3$); ^1H NMR (500 MHz, $CDCl_3$) δ 8.01 (d, *J* = 7.1 Hz, 2H), 7.56-7.48 (m, 5H), 7.48-7.42 (m, 4H), 7.42-7.26 (m, 37H), 7.26-7.13 (m, 20H), 7.09 (t, *J* = 7.2 Hz, 2H), 5.73 (s, 1H), 5.49 (s, 1H), 5.44 (s, 1H), 5.32-5.29 (m, 1H), 5.28 (d, *J* = 3.5 Hz, 1H), 5.23 (d, *J* = 3.7 Hz, 1H), 5.18 (d, *J* = 10.6 Hz, 2H), 4.90-4.84 (m, 3H), 4.82-4.76 (m, 4H), 4.75-4.46 (m, 12H), 4.45-4.30 (m, 5H), 4.25-4.09 (m, 5H), 4.09-3.82 (m, 10H), 3.77 (d, *J* = 9.4 Hz, 1H), 3.71 (d, *J* = 3.7 Hz, 1H), 3.69-3.56 (m, 6H), 3.53 (d, *J* = 9.2 Hz, 1H), 3.47 (t, *J* = 9.4 Hz, 1H), 3.41-3.26 (m, 4H), 3.22 (dd, *J* = 10.4, 3.4 Hz, 1H), 1.83-1.70 (m, 2H). ^{13}C NMR (125 MHz, $CDCl_3$) δ 166.14, 139.23, 138.88, 138.15, 138.04, 137.88, 137.64, 133.19, 130.09, 129.81, 129.01, 128.96, 128.77, 128.66, 128.53, 128.51, 128.48, 128.42, 128.38, 128.30, 128.22, 128.18, 128.16, 128.09, 127.98, 127.95, 127.82, 127.63, 127.59, 127.55, 127.49, 127.24, 126.44, 126.30, 126.27, 101.38, 101.06, 100.66, 98.39, 95.02, 95.90, 92.09, 82.75, 79.68, 79.21, 78.74, 76.14, 75.53, 75.16, 75.11, 74.81, 74.70, 74.38, 73.16, 72.52, 71.78, 69.45, 69.05, 67.35, 65.32, 63.23, 62.85, 62.75, 62.30, 62.19, 51.14, 44.96, 27.84; HRMS (ESI): *m/z* calcd for $C_{132}H_{134}N_4O_{28}[M+NH_4]^+$: 2240.9528, found: 2240.9553.

3-Aminopropyl 2-acetamino-2-deoxy-α-D-glucopyranosyl-(1→2)-α-D-glucopyranosyl-(1→2)-α-D-galactopyranosyl-(1→3)-3-_O_-(α-D-galactopyranosyl)-α-D-glucopyranoside (2)

To a solution of **37** (55 mg, 25 μmol) in MeOH/DCM (5 mL, v/v = 1:1) was added NaOMe (14 mg, 0.2 mmol) at 0 °C, and the resulting mixture was warmed gradually to room temperature. The mixture was stirred for 12 h at the same temperature, at the end of which time TLC indicated the reaction was finished. The reaction was quenched with Amberlite IR120 H+ resin. After filtration, the resulting mixture was concentrated to dryness. The obtained residue was purified by silica gel column chromatography (3:1, hexanes/ethyl acetate) to afford a white solid. 1,3-Propanedithiol was added to a solution of the obtained solid in pyridine/H_2O. The mixture was stirred at 50 °C for 2 days. Afterward, the solvent was evaporated without purification. The residue was dissolved in MeOH, followed by the addition of Ac_2O, and stirred at room temperature overnight. The solvent was then evaporated to dryness, and the product was purified by silica gel column chromatography

to yield a white solid. To a solution of the obtained solid in *t*-BuOH/H_2O/TFA (5 mL, v/v/v = 4:1:0.04) was added 20% $Pd(OH)_2/C$ (50 mg), and the reaction mixture was stirred under a hydrogen atmosphere at 30 °C. The mixture was stirred for 72 h. The reaction mixture was filtered and the filtrate was concentrated *in vacuo*. The obtained residue was purified by Sephadex G-10 column (H_2O) to afford **2** (14 mg, 60% over four steps) as a white solid. $[\alpha]_D^{20}$ -2.9 (*c* 0.1, $CHCl_3$); ^1H NMR (500 MHz, D_2O) δ 5.59 (d, *J* = 3.6 Hz, 1H), 5.53 (d, *J* = 3.7 Hz, 1H), 5.04 (d, *J* = 3.7 Hz, 1H), 4.90 (d, *J* = 3.7 Hz, 1H), 4.86 (d, *J* = 3.9 Hz, 1H), 4.33 (t, *J* = 6.4 Hz, 1H), 3.96-3.73 (m, 15H), 3.73-3.52 (m, 12H), 3.47-3.31 (m, 4H), 3.13-3.02 (m, 2H), 2.00 (s, 3H), 1.94 (p, *J* = 6.1 Hz, 2H). ^{13}C NMR (125 MHz, D_2O) δ 173.54, 98.35, 98.28, 94.96, 93.02, 90.16, 76.23, 72.89, 72.08, 71.83, 71.41, 71.09, 70.95, 70.90, 70.38, 70.10, 69.89, 69.69, 69.46, 69.37, 69.11, 68.85, 68.78, 68.27, 67.91, 66.18, 65.80, 61.08, 60.89, 60.64, 60.11, 53.15, 37.72, 26.41, 22.16; HRMS (ESI): *m*/*z* calcd for $C_{35}H_{62}N_2O_{26}[M+H]^+$: 927.3669, found:927.3645.

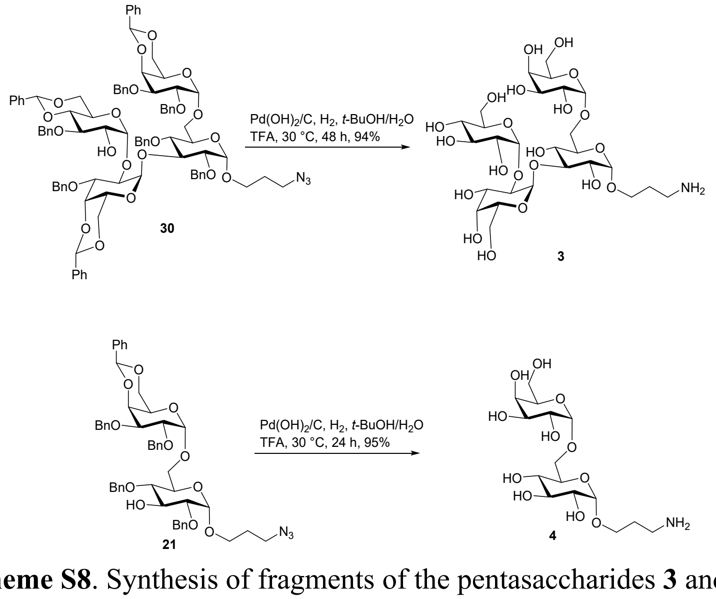

Scheme S8. Synthesis of fragments of the pentasaccharides **3** and **4**.

3-Aminopropyl α-D-glucopyranosyl-(1→2)-α-D-galactopyranosyl-(1→3)-3-*O*-(α-D-galactopyranosyl)-α-D-glucopyranoside (3)

To a solution of **30** (100 mg, 0.06 mmol) in *t*-BuOH/H_2O/TFA (5 mL, v/v/v = 4:1:0.04) was added 20% $Pd(OH)_2/C$ (100 mg), and the reaction mixture was stirred under a hydrogen atmosphere at 30 °C. The mixture was stirred for 48 h. The reaction mixture was filtered and the filtrate was concentrated *in vacuo*. The obtained residue was purified by Sephadex G-10 column (H_2O) to afford **3** (36 mg, 94%) as a white solid. $[\alpha]_D^{20}$ +10.1 (*c* 0.09, H_2O); ^1H NMR (500 MHz, D_2O) δ 5.46 (d, *J* = 3.3 Hz, 1H), 5.12 (d, *J* = 3.9 Hz, 1H), 4.92 (d, *J* = 3.7 Hz, 1H), 4.91 (d, *J* = 3.8 Hz, 1H), 4.28-4.22 (m, 1H), 4.00 (dd, *J* = 3.0, 1.3 Hz, 1H), 3.99-3.94 (m, 4H), 3.91 (tt, *J* = 5.9, 2.2 Hz, 2H), 3.88-3.76 (m, 7H), 3.75-3.72 (m, 2H), 3.71-3.65 (m, 6H), 3.64-3.63 (m, 1H), 3.58 (td, *J* = 5.3, 2.4 Hz, 1H), 3.54 (dd, *J* = 9.8, 3.9 Hz, 1H), 3.39 (dd, *J* = 10.1, 9.2 Hz, 1H), 3.08-2.95 (m, 2H),

1.97-1.87 (m, 2H). ^{13}C NMR (125 MHz, D_2O) δ 98.50, 97.99, 96.75, 95.71, 80.31, 72.79, 72.47, 71.65, 71.26, 70.91, 70.50, 70.06, 69.50, 69.30, 69.19, 69.10, 69.05, 68.29, 67.39, 65.94, 65.08, 61.00, 60.66, 60.28, 37.72, 26.38; HRMS (ESI): *m*/*z* calcd for $C_{27}H_{49}NO_{21}[M+H]^+$: 724.2875, found:724.2860.

3-Aminopropyl α-D-galactopyranosyl-(1→6)-α-D-glucopyranoside (4)

To a solution of **21** (50 mg, 57 μmol) in *t*-BuOH/H_2O/TFA (5 mL, v/v/v = 4:1:0.04) was added 20% $Pd(OH)_2/C$ (50 mg), and the reaction mixture was stirred under a hydrogen atmosphere at 30 °C. The mixture was stirred for 24 h. The reaction mixture was filtered and the filtrate was concentrated *in vacuo*. The obtained residue was purified by Sephadex G-10 column (H_2O) to afford **4** (22 mg, 95%) as a white solid. $[\alpha]_D^{20}$ +22.2 (*c* 0.05, H_2O); ^1H NMR (500 MHz, D_2O) δ 4.93 (d, *J* = 3.6 Hz, 1H), 4.90 (d, *J* = 3.8 Hz, 1H), 3.98-3.90 (m, 3H), 3.87 (dt, *J* = 10.8, 5.6 Hz, 1H), 3.82 (dd, *J* = 10.3, 3.1 Hz, 1H), 3.80-3.76 (m, 2H), 3.73-3.67 (m, 3H), 3.64 (t, *J* = 9.4 Hz, 1H), 3.61-3.56 (m, 1H), 3.55 (dd, *J* = 9.9, 3.4 Hz, 1H), 3.48 (t, *J* = 9.8 Hz, 1H), 3.18-3.03 (m, 2H), 1.97 (p, *J* = 6.2 Hz, 2H). ^{13}C NMR (125 MHz, D_2O) δ 98.26, 97.95, 73.09, 70.89, 70.85, 70.30, 69.34, 69.22, 69.07, 68.25, 65.80, 65.42, 60.97, 37.65, 26.38; HRMS (ESI): *m*/*z* calcd for $C_{15}H_{29}NO_{11}[M+H]^+$: 400.1819, found: 400.1818.

Computational Methods and Results

All computational results were obtained from the Gaussian 16 program.[10] Chemcraft 1.6 and Chimera were used to visualize structures and molecular orbitals. Calculations were performed adopting the B3LYP functional,[11, 12] and the 6-31+G(d) basis set for structure optimizations, single point geometries calculations, and free energy calculations. Calculations were conducted on closed-shell singlet. Geometry optimizations were also performed without symmetry constraints at 298.15 K and 1 atmosphere with unscaled vibrational frequencies.

a)

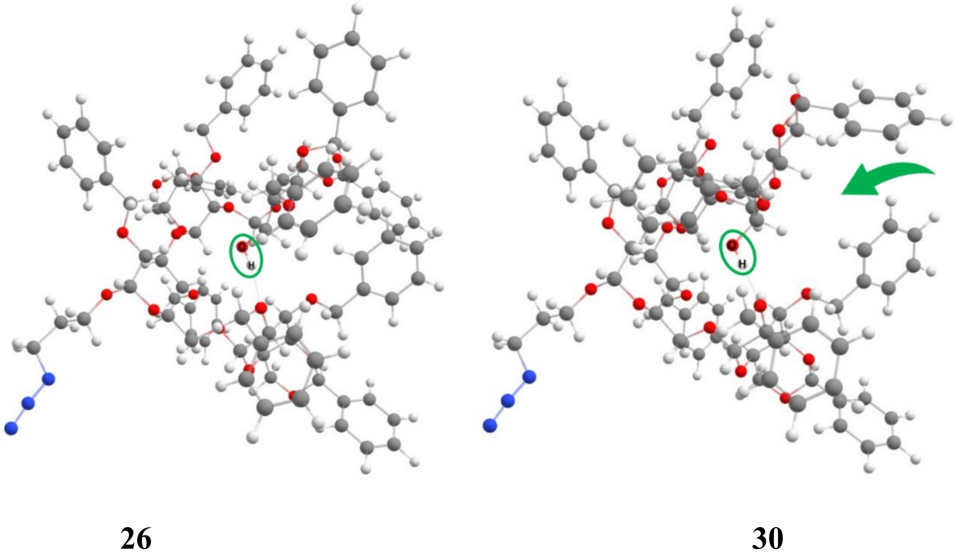

26 **30**

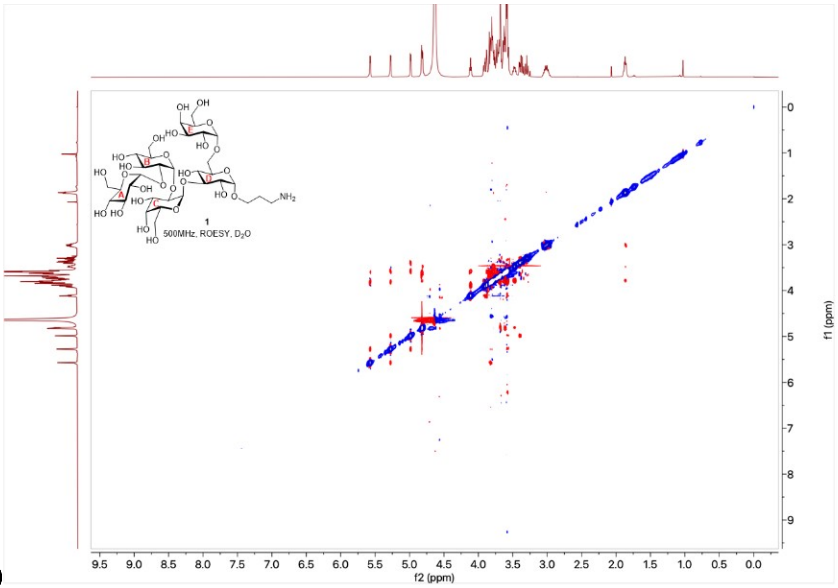

b)

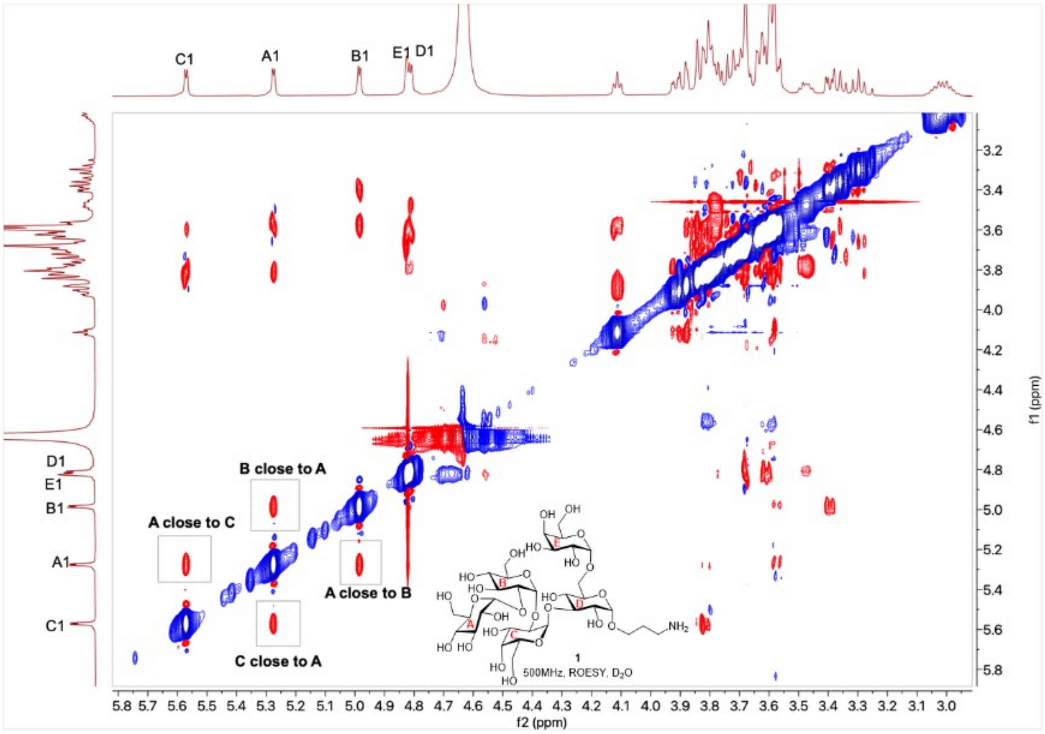

Figure S2. a) Geometry optimized structure of pentasaccharides **26** and **30** with charge = 0, multiplicity = 1. The OH in the compound **26** is deeply embedded within the center of molecule. Red atom: oxygen; white atom: hydrogen; grey atom: carbon; blue atom: nitrogen. b) ROESY-NMR spectrum and expansion of the ROESY-NMR spectrum of pentasaccharide **1**. Correlations

were observed between the anomeric protons of residues A, B and C suggesting these residues are close to each other in space confirming the sterically congested nature of pentasaccharide **1**.

Synthesis of BSA-glycan conjugates

An aqueous solution of compound **1** (5 mg, 5.6 μmol) in $NaHCO_3$ was prepared (500 μL, 10 mg/mL), then chloroform (750 μL) containing thiophosgene (1.67 μL, 21.8 μmol) was added. The reaction mixture was stirred vigorously at room temperature until complete consumption of starting material **1**, as monitored by ESI-HRMS. Upon completion, the reaction mixture was diluted with 2 mL of water, and the aqueous layer was extracted twice with 1 mL chloroform to remove excess thiophosgene. The aqueous layer was collected and lyophilized to afford compound **1'**. To a solution of 10 mg/ml BSA in PBS (100 mM, pH=8.0), **1'** (90 equiv to per BSA molecule) was added. The solution was gently mixed and nutated under 37 °C overnight. The protein was recovered with a 0.5 ml Amicon filter (MWCO = 30 kDa) under 13,000 g and washed with Milli-Q water four times, then lyophilized and stored in -20 °C. Conjugation reactions of BSA with **2', 3', 4'** were conducted in similar manners. The final conjugates were analyzed by matrix assisted laser desorption ionization mass spectrometry (MALDI-MS) (**Figure S2**).

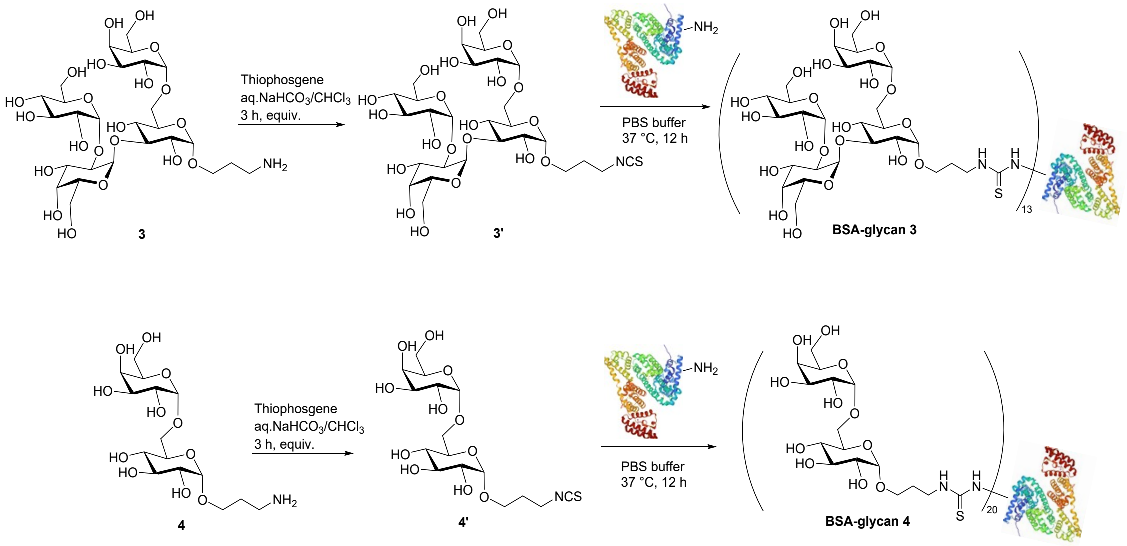

Scheme S9: Syntheses of BSA-glycan **3** and BSA-glycan **4** conjugates.

Synthesis of mQβ-glycan conjugates

To a solution of mQβ particle (Qβ A38K/A40C/D102C, 5 mg/ml) in PBS (100 mM, pH=7.4), **1'** (30 equiv to mQβ subunits) was added. The solution was gently mixed and nutated under 37 °C overnight. The protein was recovered with a 0.5 ml Amicon filter (MWCO= 50 kDa) under 13,000 g and washed with PBS (100 mM, pH=7.4) four times. Conjugation of mQβ with **2'** was conducted in similar manners. The final conjugates were analyzed by electrospray high resolution mass spectrometry (ESI-HRMS) (**Figure S3**).

Procedure for mouse immunization.

Pathogen-free C57BL6 female mice aged 6 weeks were purchased from Charles River and maintained in the University Laboratory Animal Resources facility of Michigan State University. All animal experiments were performed in accordance with the guidelines of the Institutional Animal Care and Use Committee (IACUC) of Michigan State University. C57BL6 mice were injected subcutaneously under the scruff with 0.2 mL vaccine constructs. Immunization procedures were performed on days 0, 14 and 28. Blood samples were collected on days 0, 7, 21 and 35.

Procedure for rabbit immunization.

Rabbit immunization studies were performed by ProSci Inc (Poway, CA). Each dose of the vaccine construct (with an equivalent glycan amount of 15 nmol glycan for group I and 5 nmol for all other groups) was injected subcutaneously. The vaccination was adjuvanted with Alum in group F, G, I. In Group J, the vaccination was adjuvanted with MPLA. Immunization procedures were performed on day 0, 14, 28 and 42. Blood samples were collected on days 0, 7, 21 and 35. The terminal bleeding was performed on day 49.

Evaluation of antibody titters and subtypes by ELISA

Immulon 4 HBX 384 well plates (Thermo Fisher 8755) were coated with a solution of BSA-glycan conjugates or COPS (10 μg/mL, 50 μL/well) in PBS buffer and incubated at 4 °C overnight. The plate was washed with PBST (4 × 100 μL) and blocked with 1% BSA/PBS (100 μL/well) for 1 h at room temperature. The liquid was discarded. The plates were washed with PBST (4 × 100 μL), and incubated with serial dilutions of anti-sera from immunized mice or rabbits in 0.1% BSA/PBS (50 μL/well, 3 wells for each dilution). The plates were incubated for 2 h and then liquid was discarded. The plates were washed with PBST (4 × 100 μL). A 1:2000 dilution of HRP-conjugated goat anti-mouse IgG (Cat # 115-035-003, Jackson Immuno Research Laboratory) or a 1:5000 dilution of HRP-conjugated goat anti-rabbit IgG (Cat # 111-035-003, Jackson Immuno Research Laboratory) in 0.1% BSA/PBS (50 μL) was added to each well and incubated for 1 h. The liquid was discarded, and the plates were washed with PBST (4 × 100 μL). Then, the substrate solution (75 μL) was added to each well, and the reaction was incubated for 15 minutes. To stop the reaction, 0.5 M H_2SO_4 (50 μL) was added. OD450 nm was immediately recorded and fitted into 4PL nonlinear logistic model via GraphPad Prism 6 with least squares algorism. The near-background (endpoint) titer was determined through regression analysis, where the log10 dilution was plotted against optical density. It was reported as the highest dilution fold (ELISA units) that yielded an optical absorbance at the threshold level. The threshold should be 3 times standard deviation above the blank wells on the plate.

Bacterial strains and growth conditions

All the subtypes of *Salmonella* have been described previously (*S*. Enteritidis R11[13], *S*. Enteritidis R11 Δ*invA* Δ*rfaL*[14], *S*. Typhimurium I77[13], *S*. Paratyphi A ATCC 9150[15], *S*. Newport Chile 361, *S*. Newport Chile 361 Δ*rfaL* [16]) and was maintained in Hy-Soy media (Teknova, CA) as described.[17]

Procedure for flow cytometry

A single colony of *S.* Paratyphi A ATCC 9150 was grown in HS broth overnight at 37°C with shaking at 220 rpm. The following day, bacteria were adjusted to an OD600 of 0.4 and placed on ice. For samples requiring thanatin treatment, the overnight culture (10 mL) was inoculated into fresh HS broth (10 mL) in a 50 mL tube. Thanatin (HY-P5601, MedChemExpress, NJ) was then added to achieve a final concentration of 0.1 μM. The cells were incubated at 37°C for 6 hours. After incubation, the bacterial culture was adjusted to an OD600 of 0.4 and placed on ice. The bacterium suspension (250 μL) was washed once with flow buffer (1% heat-inactivated FBS in PBS) and incubated with various dilutions of pooled rabbit sera (heat-inactivated for 30 min at 56 °C) for 1 h at 4°C. Rabbit sera tested included both the pre-immune and the post-immune (D49) sera. Bacteria were then washed two times with flow buffer, followed by incubation with FITC-conjugated donkey anti-rabbit IgG (Cat # 406403, Biolegend, CA, 1 μg/mL) diluted in flow buffer for 1 h at 4 °C. Bacteria were then washed twice with PBS, fixed with 2% formaldehyde, and read using BD Accuri C6 with 1×10^4 events recorded. As a negative control, bacteria were incubated with the secondary antibody alone. Flow cytometry analysis of the other strains was conducted in similar manners.

Serum bactericidal antibody assay

Bacteria were prepared as follows: a single colony of *S.* Typhimurium I77 was used to inoculate an overnight HS broth culture incubated at 37 °C with shaking at 220 rpm. On the following day, log-phase cultures were prepared by diluting the overnight culture 1:50 in fresh HS broth (37 °C, 220 rpm) with thanatin added to the broth (final concentration of thanatin was 0.5 μM). Bacteria were harvested when the OD600 value reached 0.4. Heat-inactivated serum (56 °C for 30 minutes) was diluted 1:50 in Hanks' Balanced Salt Solution (HBSS) and mixed with *S.* Typhimurium I77 (250 CFU). The sera and bacteria were incubated at 4 °C overnight. After incubation, the mixture was supplemented with 15% (final concentration) baby rabbit complement (31061-1, Pel-Freez Biologicals). Negative controls included *S.* Typhimurium I77 only and *S.* Typhimurium I77 with complement alone. To confirm that the serum was free of active complement following heat inactivation, a subset of samples containing *S.* Typhimurium I77 and serum (without exogenous complement) was tested for bactericidal activity. The mixtures were then incubated for 1 hour at 37 °C, plated on HS agar plates, and incubated overnight at 37 °C. The bactericidal percentage was calculated as the number of bacteria that survived divided by the number of bacteria in the bacteria-plus-complement control group × 100%. All samples were assayed in duplicate, and the average results were reported. The same procedure was followed for *S.* Enteritidis R11, *S.* Newport Chile 361, and *S.* Paratyphi A ATCC 9150, with the following modifications: (1) for *S.*

Newport Chile 361, the final complement concentration used was 10%; (2) for *S.* Paratyphi A ATCC 9150, 1,000 CFU were used instead of 250 CFU.

a)

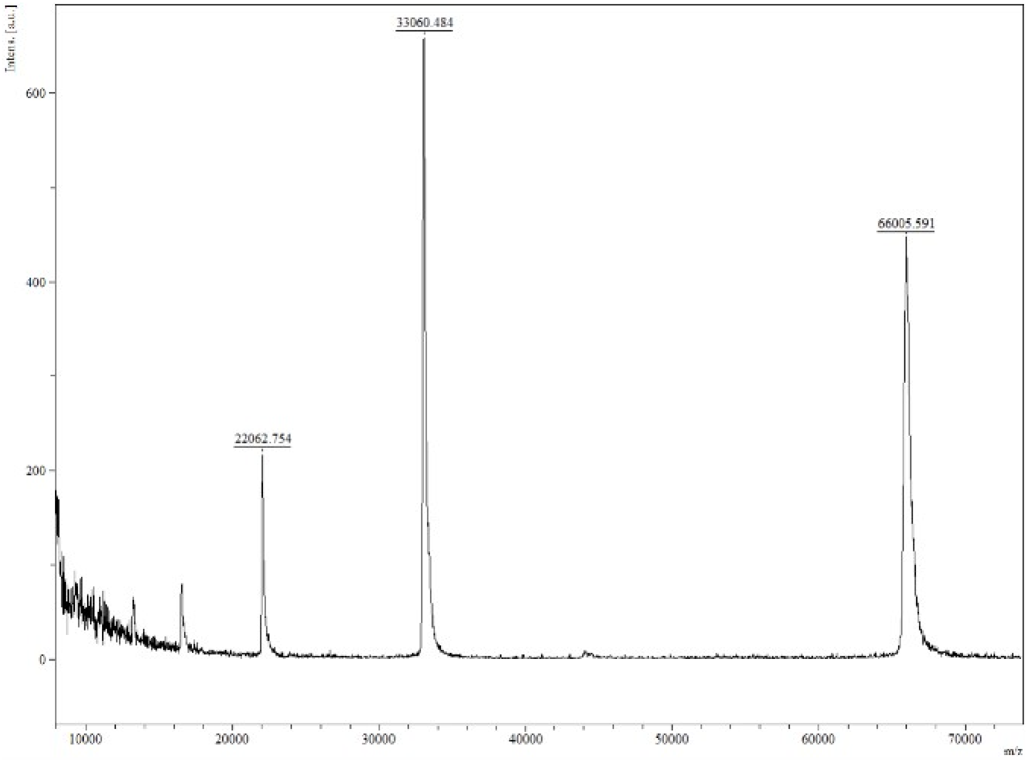
Intens. [a.u.]
600
400
200
0
33060.484
66005.591
22062.754
10000
20000
30000
40000
50000
60000
70000
m/z

b)

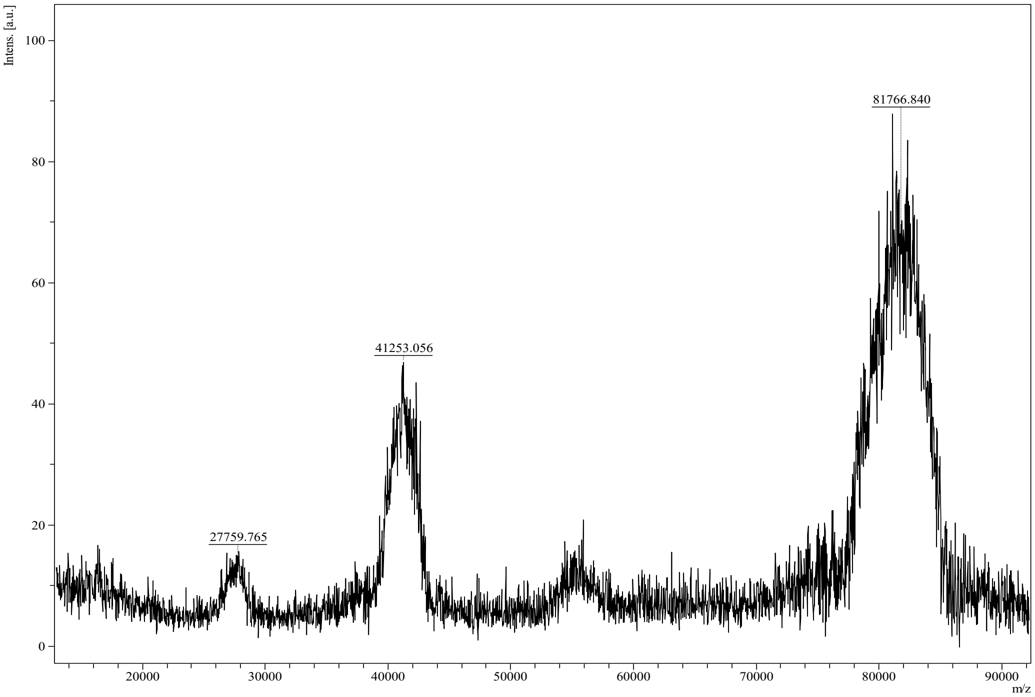
Intens. [a.u.]
100
80
60
40
20
0
81766.840
41253.056
27759.765
20000
30000
40000
50000
60000
70000
80000
90000
m/z

c)

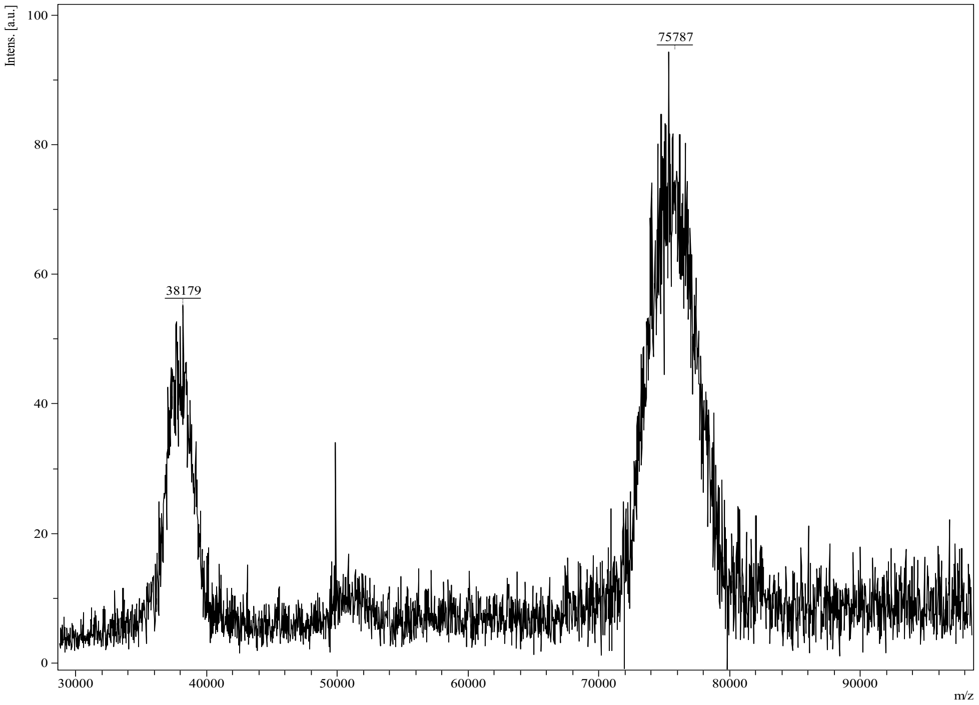

Intens. [a.u.]
100
80
60
40
20
0
38179
75787
30000
40000
50000
60000
70000
80000
90000
m/z

d)

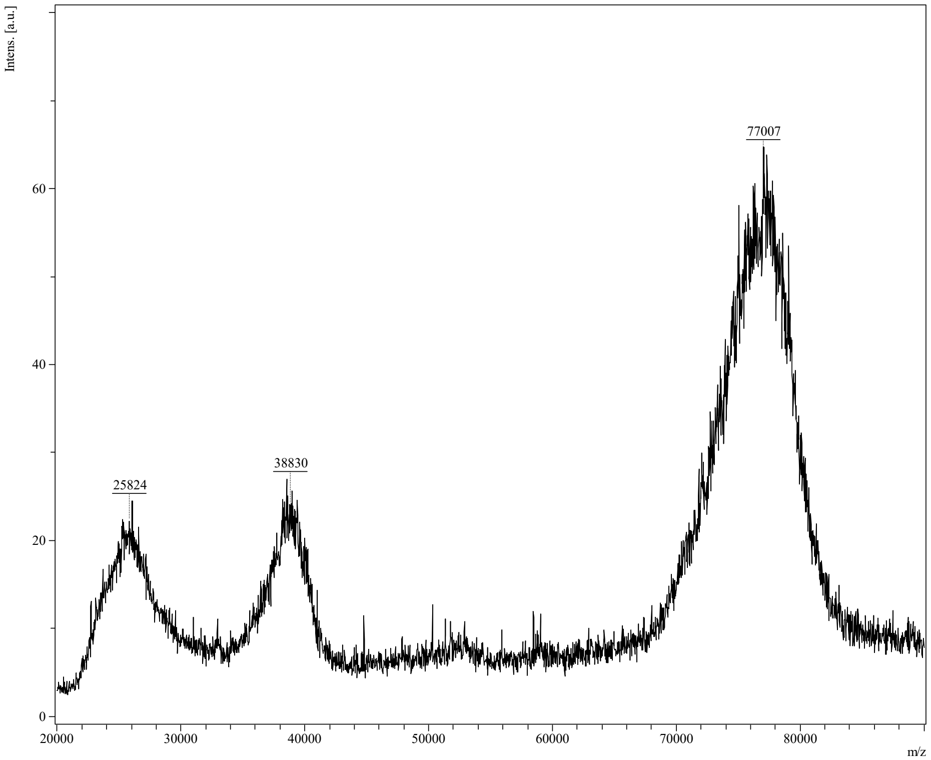

Intens. [a.u.]
60
40
20
0
25824
38830
77007
20000
30000
40000
50000
60000
70000
80000
m/z

e)

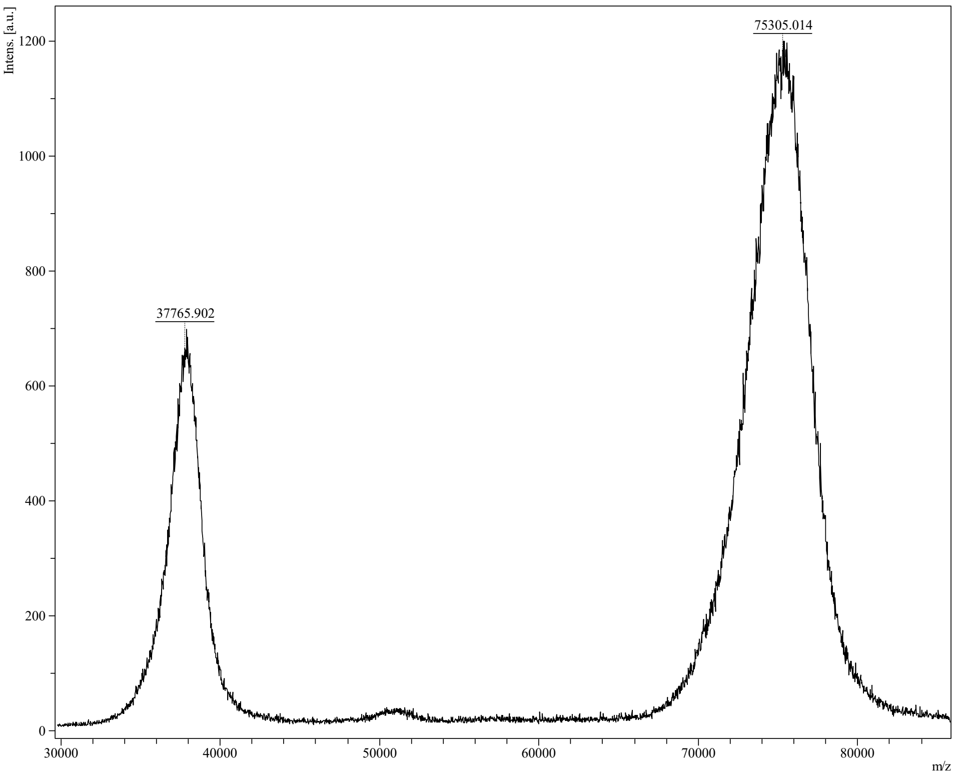

Figure S3. MALDI-TOF MS characterization of BSA-glycans. a) BSA protein only; b) BSA-glycan **1**. The molecular weight of BSA shifted from 66,005 Da to 81,766 Da after conjugation, indicating an average of 17 glycan **1** units per BSA molecule; c) BSA-glycan **2**. The molecular weight of BSA shifted from 66,005 Da to 75,787 Da after conjugation, indicating an average of 10 glycan **2** units per BSA molecule; d) BSA-glycan **3**. The molecular weight of BSA shifted from 66,005 Da to 77,007 Da after conjugation, indicating an average of 13 glycan **3** units per BSA molecule; e) BSA-glycan **4**. The molecular weight of BSA shifted from 66,005 Da to 75,305 Da after conjugation, indicating an average of 20 glycan **4** units per BSA molecule. The difference of MW before and after conjugation divided by the MW of glycans (glycan **1**: 927; glycan **2**: 969; glycan **3**: 754; glycan **4**: 461) gave the average loading copies of glycans per BSA molecule.

a)

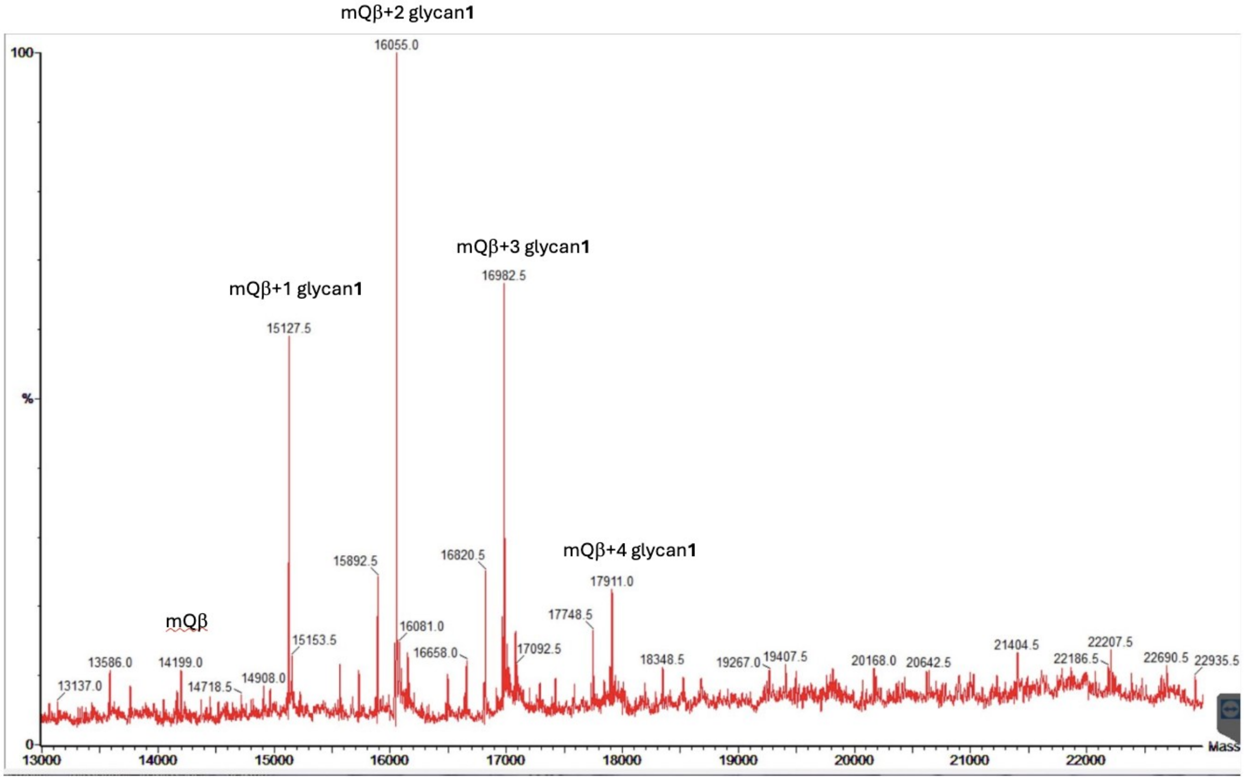
mQβ+2 glycan1
16055.0
mQβ+3 glycan1
16982.5
mQβ+1 glycan1
15127.5
mQβ+4 glycan1
17911.0
mQβ
100
%
0
Mass
13000
14000
15000
16000
17000
18000
19000
20000
21000
22000

b)

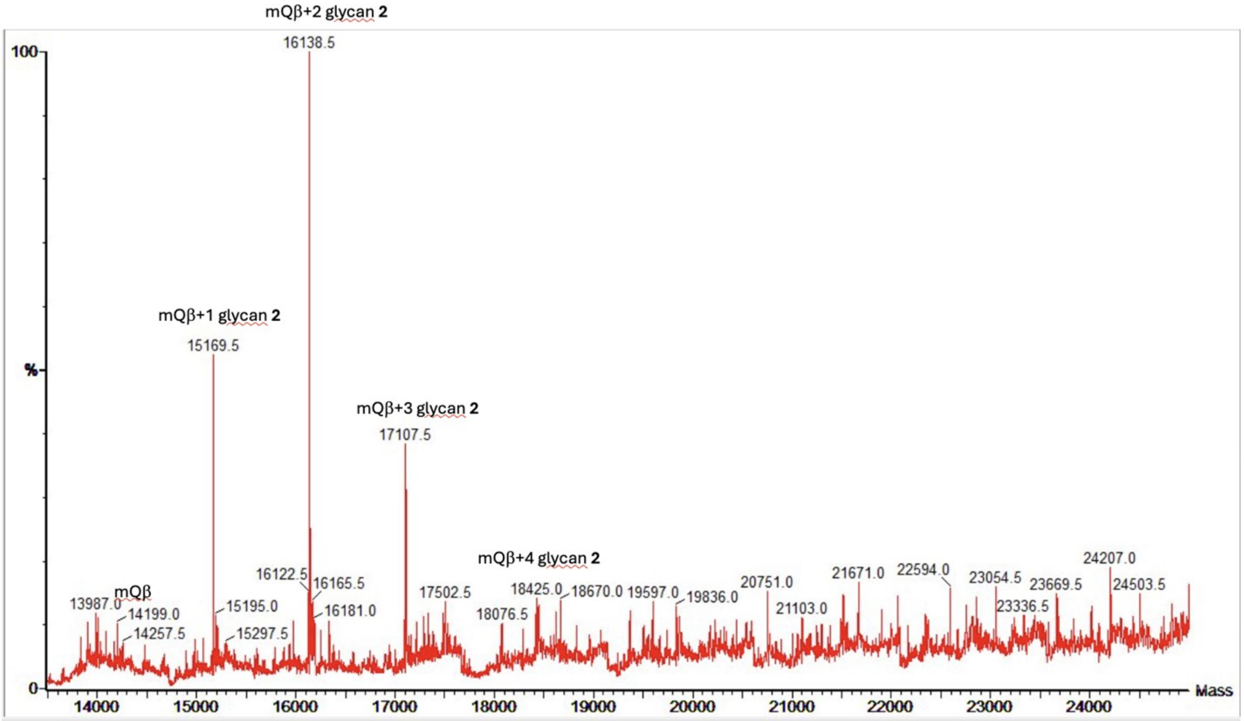
mQβ+2 glycan 2
16138.5
mQβ+1 glycan 2
15169.5
mQβ+3 glycan 2
17107.5
mQβ+4 glycan 2
mQβ
100
%
0
Mass
14000
15000
16000
17000
18000
19000
20000
21000
22000
23000
24000

Figure S4. ESI-TOF HRMS spectra of mQβ-glycan **1** and mQβ-glycan **2** conjugates. a) Mass spectrometry analysis of the mQβ-glycan **1** conjugate showed an average loading of 378 pentasaccharides on each capsid. The peaks observed showed a sequential mass shift of ~928 corresponding to the addition of one unit of glycan **1**. b) Mass spectrometry analysis of mQβ-glycan **2** conjugate showed an average loading of 350 pentasaccharides on each capsid. The peaks observed showed a sequential mass shift of ~969 corresponding to the addition of one unit of glycan **2**. The average loading was calculated using the following formula: (sum of the intensities of peaks of mQβ conjugate multiplied by the number of glycan attached for the peak) / (sum of the intensities of peaks of mQβ conjugate and that of the unmodified mQβ).

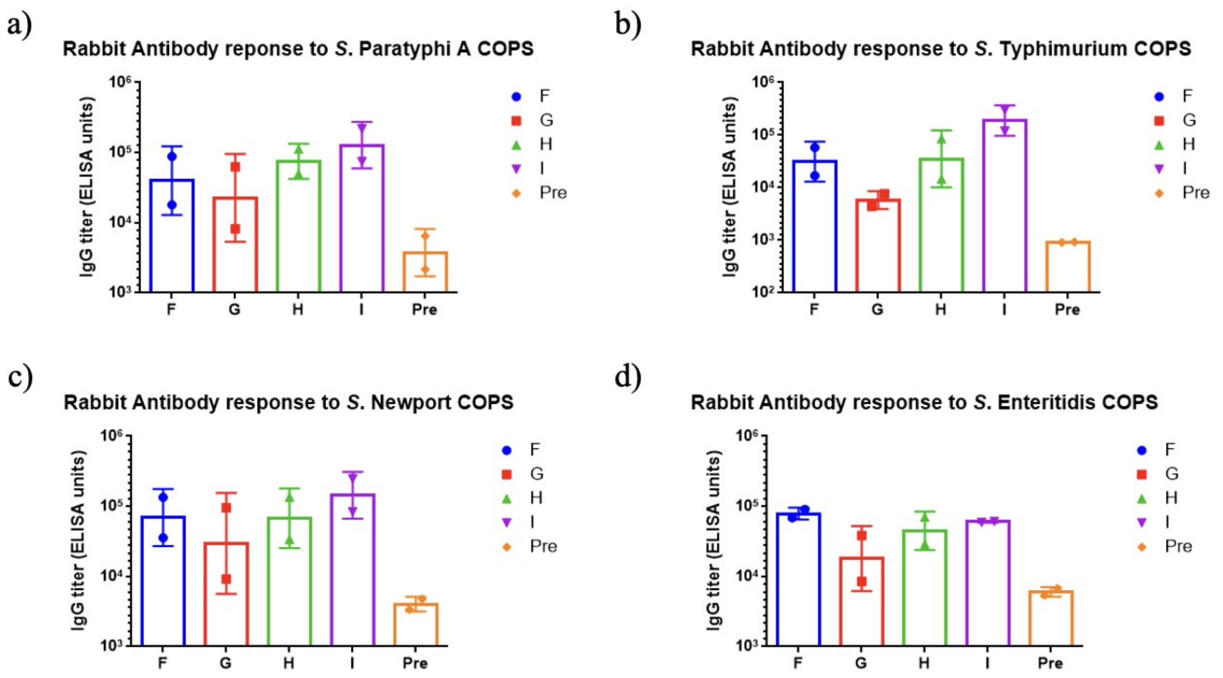

Figure S5. ELISA analysis for antibody binding to native *Salmonella* COPS. Individual anti-sera from rabbits immunized with mQβ-glycan **1** and mQβ-glycan **2** were assessed for COPS binding by ELISA as indicated. Each symbol represents one rabbit. Data are presented as geometric mean values ± standard deviation. X-axis labels: Pre (pre-immunized sera from rabbits); F (F group: day 49 sera from rabbits immunized with mQβ-glycan **1** (5 µg glycan **1** with Alum)); G (G group: day 49 sera from selected two rabbits immunized with mQβ-glycan **1** (15 µg glycan **1** with Alum)); H (H group: day 49 sera from selected two rabbits immunized with mQβ-glycan **2** (5 µg glycan **1** with Alum)); I (I group: day 49 sera from selected two rabbits immunized with mQβ-glycan **2** (5 µg glycan **1** with MPLA)).

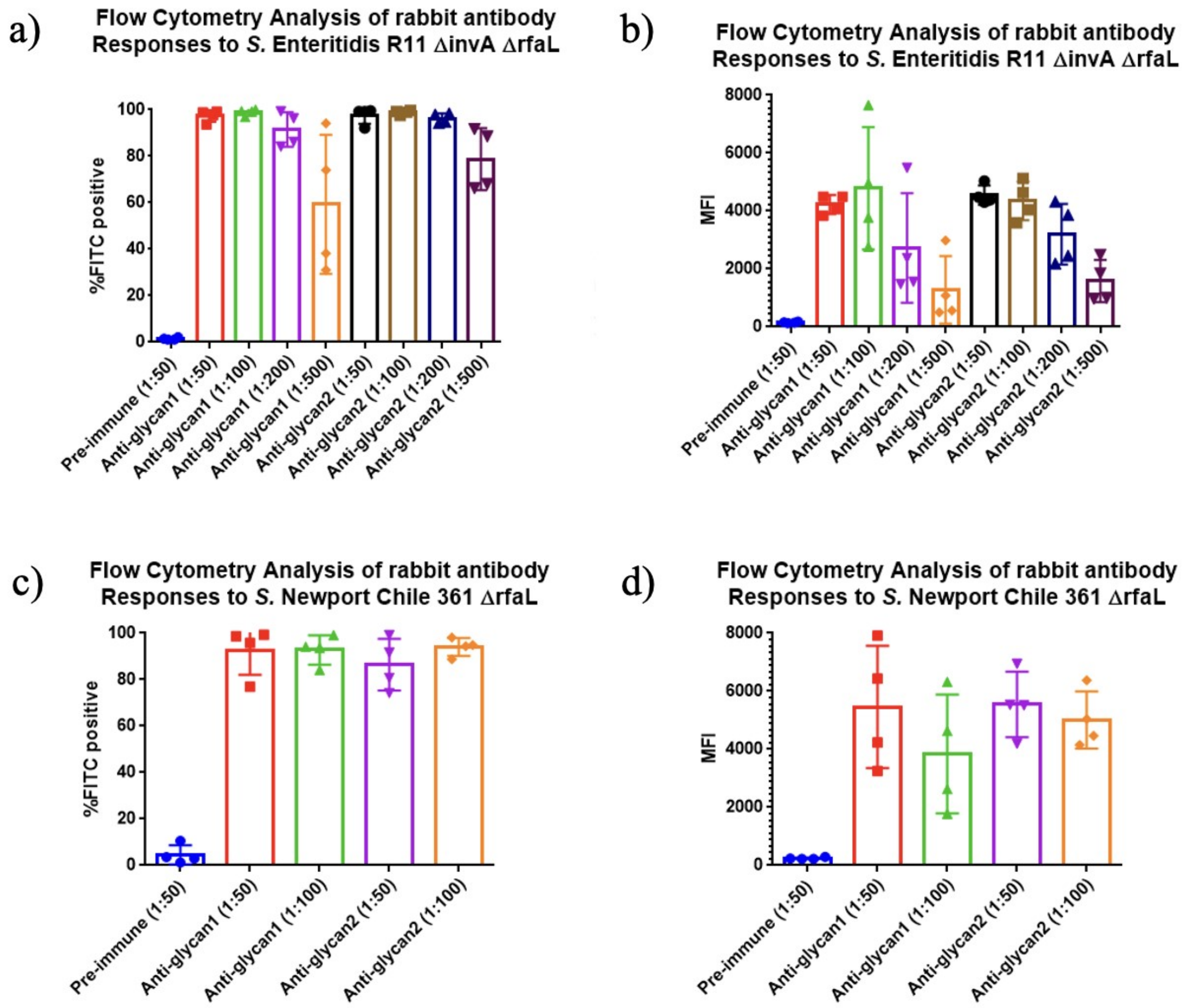

Figure S6. mQβ-glycan **1** and mQβ-glycan **2** induced rabbit antibodies bind *Salmonella* bacteria. Each symbol corresponds to an individual animal. a) percentages of positive *S.* Enteritidis R11 *ΔinvA ΔrfaL* cells stained by various dilutions of sera as quantified by flow cytometry; b) Mean fluorescence intensities (MFI) of *S.* Enteritidis R11 *ΔinvA ΔrfaL* cells; c) percentages of positive *S.* Newport Chile 361 *ΔrfaL* cells stained by various dilutions of sera as quantified by flow cytometry; d) Mean fluorescence intensities (MFI) of *S.* Newport Chile 361 *ΔrfaL* cells. FTIC-conjugated anti rabbit IgG was used as the secondary antibody for detection.

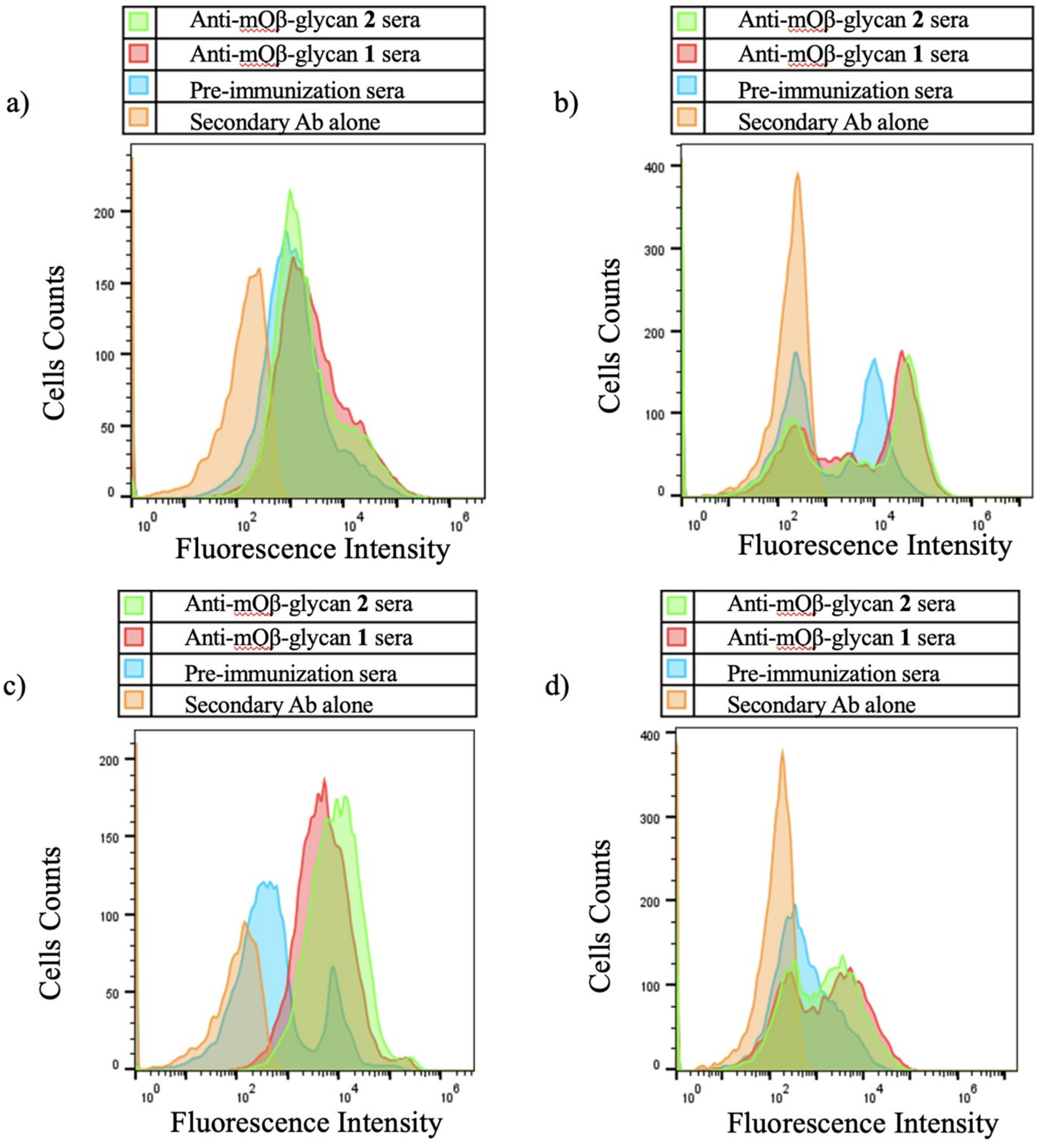

Figure S7. mQβ-glycan **1** and mQβ-glycan **2** induced rabbit antibodies bound thanatin treated *Salmonella* strains. a) Flow cytometry graphs of post-immunization sera (1:50 dilution) binding with *S*. Enteritidis R11 strain treated with thanatin at a final concentration of 0.5 µM, as compared to anti-rabbit IgG secondary antibody alone or pre-immunization rabbit sera (1:50 dilution). b) Flow cytometry graphs of post-immunization sera (1:50 dilution) binding with 0.5 µM thanatin treated *S*. Typhimurium I77 strain, as compared to anti-rabbit IgG secondary antibody alone or pre-immunization rabbit sera (1:50 dilution). c) Flow cytometry graphs of post-immunization sera (1:50 dilution) binding with 0.5 µM thanatin treated *S*. Paratyphi A ATCC 9150 strain, as

compared to anti-rabbit IgG secondary antibody alone or pre-immunization rabbit sera (1:50 dilution). d) Flow cytometry graphs of post-immunization sera (1:50 dilution) binding with 0.2 μM thanatin treated *S*. Newport Chile 361 strain, as compared to anti-rabbit IgG secondary antibody alone or pre-immunization rabbit sera (1:50 dilution).

Table S1. NMR data of compound **10**

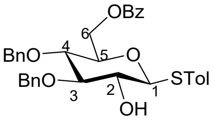

10

Position	^{1}H (ppm)	^{13}C (ppm)	^{1}H-^{1}H COSY	HMBC (H→C)
1	4.46 (d, *J* = 9.3 Hz, 1H)	87.75	H-2	C-2, C-3
2	3.43 (t, *J* = 9.3 Hz, 1H)	72.44	H-1, H-3	C-1, C-3
3	3.68-3.62 (m, 1H)	85.96	H-2, H-4	C-2, C-4
4	3.59-3.55 (m, 1H)	76.99	H-3, H-5	C-5, C-6
5	3.68-3.66 (m, 1H)	77.31	H-4, H-6	C-4
6	4.69 (dd, *J* = 11.9, 2.1 Hz, H-6a, 1H)<br>4.44 (dd, *J* = 11.9, 4.5 Hz, H-6b, 1H)	63.35	H-5	C-5

Table S2. NMR data of compound **12**

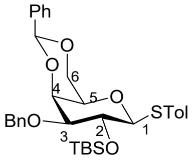

12

Position	^{1}H (ppm)	^{13}C (ppm)	^{1}H-^{1}H COSY	HMBC(H→C)
1	4.52 (d, *J* = 9.0 Hz, 1H)	89.52	H-2	C-2, C-3
2	4.02 (t, *J* = 9.0 Hz, 1H)	69.21	H-1, H-3	C-1, C-3
3	3.42 (dd, *J* = 9.0, 3.5 Hz, 1H)	82.43	H-2, H-4	C-2, C-4
4	4.08 (dd, *J* = 3.5, 1.1 Hz, 1H)	73.01	H-3, H-5	C-3, C-5
5	3.37-3.35 (m, 1H)	69.77	H-4, H-6	C-1, C-6
6	4.32 (dd, *J* = 12.3, 1.8 Hz, H-6a, 1H)<br>3.93 (dd, *J* = 12.3, 1.8 Hz, H-6b, 1H)	69.57	H-5	C-4, C-5

Table S3. NMR data of compound **17α**

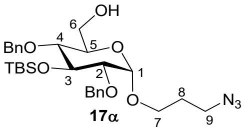

Position	^{1}H (ppm)	^{13}C (ppm)	^{1}H-^{1}H COSY	HMBC (H→C)
1	4.56 (d, J = 3.7 Hz, 1H)	97.34	H-2	C-3, C-5, C-7
2	3.28 (dd, J = 9.3, 3.7 Hz, 1H)	80.72	H-1, H-3	C-3
3	4.03 (t, J = 9.3 Hz, 1H)	73.93	H-2, H-4	C-2, C-4
4	3.43-3.36 (m, 1H)	78.75	H-3, H-5	C-3, C-5
5	3.63-3.53 (m, 1H)	71.11	H-4, H-6	C-6
6	3.71 (dd, J = 11.8, 2.8 Hz, H-6a, 1H);<br>3.71-3.63 (m, H-6b, 1H)	61.96	H-5	C-4, C-5
7	3.68-3.60 (m, H-7a, 1H);<br>3.35-3.27 (m, H-7b, 1H)	64.71	H-1, H-8	C-1, C-8, C-9
8	1.89-1.81 (m, 2H)	28.97	H-7, H-9	C-7, C-9
9	3.43-3.35 (m, 2H)	48.46	H-8	C-7, C-8

Table S4. NMR data of compound **5**

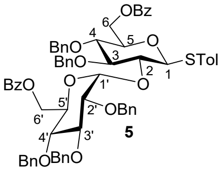

Position	^{1}H (ppm)	^{13}C (ppm)	^{1}H-^{1}H COSY	HMBC (H→C)
1	4.83 (d, J = 9.4 Hz, 1H)	86.97	H-2	C-2, C-3, C-5
2	3.95 (t, J = 9.4 Hz, 1H)	74.28	H-1, H-3	C-1, C-3, C-1'
3	3.85 (t, J = 8.9 Hz, 1H)	84.88	H-2, H-4	C-2, C-4
4	3.72-3.65 (m ,1H)	78.80	H-3, H-5	C-3, C-5, C-6
5	3.77-3.69 (m, 1H)	77.02	H-4, H-6	C-4
6	4.70 (dd, J = 12.0, 2.1 Hz, H-6a, 1H)<br>4.46 (dd, J = 12.0, 2.1 Hz, H-6b, 1H)	63.51	H-5	C-4, C-5
1'	6.05 (d, J = 3.8 Hz, 1H)	95.29	H-2'	C-2, C-2', C-3', C-5'
2'	3.72-3.64 (m, 1H)	80.04	H-1', H-3'	C-3'
3'	4.07 (t, J = 9.3 Hz, 1H)	81.88	H-2', H-4'	C-2', C-4'

4'	3.66-3.58 (m, 1H)	77.80	H-3', H-5'	C-3', C-5', C-6'
5'	4.39-4.34 (m, 1H)	68.96	H-4', H-6'	C-4'
6'	4.31 (d, J = 12.1, 2.1 Hz, H-6a', 1H)<br>3.91 (dd, J = 12.1, 3.6 Hz, H-6b', 1H)	63.26	H-5'	C-5'

Table S5. NMR data of compound **32**

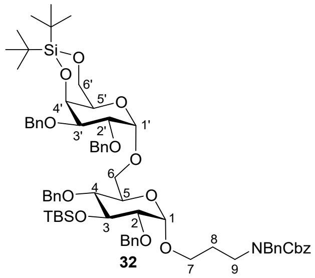

32

Position	^{1}H (ppm)	^{13}C(ppm)	^{1}H-^{1}H COSY	HMBC(H→C)
1	4.52 (1H)	97.02	H-2	
2	3.22-3.14 (m,1H)	80.78	H-1, H-3	
3	4.05-3.98 (m, 1H)	74.42	H-2, H-4	C-2, C-4
4	3.58-3.50 (m, 1H)	79.47	H-3, H-5	C-3, C-5, C-6
5	3.71-3.64 (m, 1H)	70.63	H-4, H-6	C-4
6	3.70-3.63 (m, H-6a, 1H)<br>3.72-3.67 (m, H-6b, 1H)	66.05	H-5	C-4, C-5, C-1'
7	3.62-3.53 (m, H-7a, 1H)<br>3.25-3.18 (m, H-7b, 1H)	65.37, 65.18	H-1, H-8	C-1, C-8, C-9
8	1.84-1.78 (m, 2H)	28.46, 27.77	H-7, H-9	C-7, C-9
9	3.41-3.31 (m, 2H)	51.11, 50.75	H-8	C-7, C-8
1'	5.00 (d, J = 3.5 Hz, 1H)	98.22	H-2'	C-6, C-3', C-5'
2'	4.03-3.96 (m, 1H)	74.03	H-1', H-3'	C-3'
3'	3.79 (dd, J = 10.1, 2.9 Hz, 1H)	77.16	H-2', H-4'	C-2', C-4'
4'	4.48-4.40 (m, 1H)	71.18	H-3', H-5'	C-2', C-3', C-5'
5'	3.47-3.39 (m, 1H)	67.25	H-4', H-6'	C-1', C-4', C-6'
6'	4.07-3.99 (m, 2H)	67.25	H-5'	C-5'

Table S6. NMR data of compound **22**

Position	1H (ppm)	13C(ppm)	1H-1H COSY	HMBC(H-C)
1	4.83 (d, *J* = 9.4 Hz, 1H)	86.97	H-2	C-2, C-3, C-5
2	3.95 (t, *J* = 9.2 Hz, 1H)	74.28	H-1, H-3	C-1. C-3. C-1'
3	3.85 (t, *J* = 8.9 Hz, 1H)	84.88	H-2, H-4	C-2, C-4
4	3.68 (m ,1H)	78.8	H-3, H-5	C-3, C-5, C-6
5	3.73 (m, 1H)	77.02	H-4, H-6	C-4
6	4.70 (dd, *J* = 12.0, 2.1 Hz, H-6a); 4.46 (dd, *J* = 12.0, 2.1 Hz, H-6b)	63.51	H-5	C-4, C-5
1'	6.05 (d, *J* = 3.8 Hz, 1H)	95.29	H-2'	C-2, C-2', C-3', C-5'
2'	3.68 (m, 1H)	80.04	H-1', H-3'	C-3'
3'	4.07 (t, *J* = 9.3 Hz, 1H)	801.88	H-2', H-4'	C-2', C-4'
4'	3.62 (m, 1H)	77.8	H-3', H-5'	C-3'. C-5', C-6'
5'	4.37 (ddd, *J* = 10.3, 3.6, 2.1 Hz, 1H)	68.96	H-4', H-6'	C-4'
6'	4.31 (d, *J* = 12.1, 2.1 Hz, H-6a'); 3.91 (dd, *J* = 12.1, 3.9 Hz, H-6b')	63.26	H-5'	C-5'

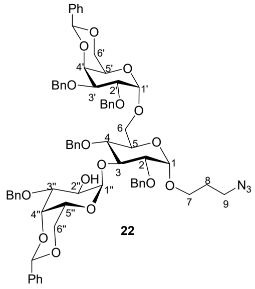

22

Position	^{1}H (ppm)	^{13}C(ppm)	^{1}H-^{1}H COSY	HMBC (H→C)
1	4.83 (1H)	96.38	H-2	C-3, C-5, C-7
2	3.34-3.28 (m, 1H)	78.93	H-1, H-3	C-3
3	4.26-4.19 (m, 1H)	75.20	H-2, H-4	C-2, C-1''
4	3.72-3.68 (m ,1H)	79.34	H-3, H-5	C-2, C-3
5	3.78-3.70 (m, 1H)	70.87	H-4, H-6	C-6
6	3.90-3.82 (m, H-6a, 1H); 3.91-3.73 (m, H-6b, 1H)	66.23	H-5	C-4, C-5, C-1'
7	3.78-3.71 (m, H-7a, 1H); 3.70-3.65 (m, H-7b, 1H)	64.53	H-1, H-8	C-1, C-8, C-9
8	1.88-1.79 (m, 2H)	28.89	H-7, H-9	C-7, C-9
9	3.43-3.35 (m, 2H)	48.29	H-8	C-8
1'	5.19 (d, *J* = 3.5 Hz, 1H)	98.79	H-2'	C-3', C-5', C-6

2'	4.11 (dd, J = 10.0, 3.5 Hz, 1H)	75.83	H-1', H-3'	C-3', C-4'
3'	3.80-3.72 (m, 1H)	75.20	H-2', H-4'	C-2'
4'	3.91-3.74 (m, 1H)	74.79	H-3', H-5'	C-3'
5'	3.54 (s, 1H)	62.87	H-4', H-6'	C-4'
6'	4.21-4.14 (m, H-6a', 1H); 3.98-3.90 (m, H-6b', 1H)	69.49	H-5'	C-4', C-5'
1"	5.60 (d, J = 3.8 Hz, 1H)	98.77	H-2"	C-2", C-3, C-3", C-5"
2"	4.21-4.14 (m, 1H)	67.99	H-1", H-3"	C-3", C-5"
3"	3.80-3.72 (m, 1H)	76.62	H-2", H-4"	C-1", C-4"
4"	3.93-3.84 (m, 1H)	74.76	H-3", H-5"	C-2", C-3"
5"	3.90-3.83 (m, 1H)	62.38	H-4", H-6"	C-1", C-4"
6"	3.98-3.90 (m, H-6a", 1H); 3.30-3.24 (m, H-6b", 1H)	69.45	H-5"	C-5"

Table S7. NMR data of compound **26**

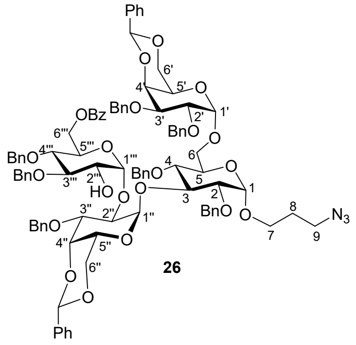

26

Position	^{1}H (ppm)	^{13}C (ppm)	^{1}H-^{1}H COSY	HMBC (H→C)
1	4.74 (1H)	96.27	H-2	C-3, C-5, C-7
2	3.36 (dd, *J* = 9.5, 3.7 Hz, 1H)	80.12	H-1, H-3	C-3
3	4.41-4.37 (m, 1H)	76.91	H-2, H-4	C-2, C-4, C-1''
4	3.91-3.86 (m, 1H)	78.66	H-3, H-5	C-3, C-5
5	3.92-3.83 (m, 1H)	70.07	H-4, H-6	C-3, C-4, C-6
6	3.89-3.81 (m, H-6a, 1H); 3.85-3.77 (m, H-6b, 1H)	66.00	H-5	C-5, C-1'
7	3.79-3.70 (m, H-7a, 1H); 3.47-3.39 (m, H-7b, 1H)	64.41	H-1, H-8	C-1, C-8
8	1.93-1.83 (m, 2H)	28.65	H-7, H-9	C-7, C-8
9	3.50-3.40 (m, 2H)	48.17	H-8	C-8
1'	5.22 (d, *J* = 3.4 Hz,1H)	98.42	H-2'	C-6, C-3', C-5'
2'	4.21-4.12 (m, 1H)	76.43	H-1', H-3'	C-3'
3'	4.08-4.00 (m, 1H)	75.76	H-2', H-4'	C-2'
4'	4.24-4.18 (m, 1H)	75.12	H-3', H-5'	C-3', C-5'
5'	3.54 (s, 1H)	62.66	H-4', H-6'	C-4'
6'	3.98 (d, *J* = 12.2 Hz, H-6a', 1H); 3.86-3.78 (m, H-6b', 1H)	69.40	H-5'	C-5'
1''	5.67 (d, *J* = 3.4 Hz, 1H)	96.80	H-2''	C-3, C-3'', C-5''
2''	4.41 (dd, *J* = 10.2, 3.4 Hz, 1H)	71.95	H-1'', H-3''	C-3'', C-1'''
3''	4.09-4.03 (m, 1H)	73.77	H-2'', H-4''	C-1'', C-4''
4''	4.22-4.15 (m, 1H)	73.27	H-3'', H-5''	C-3'', C-5''
5''	4.09-4.01 (m, 1H)	62.66	H-4'', H-6''	C-4''
6''	4.29-4.21 (m, H-6a'', 1H); 4.26-4.18 (m, H-6b'', 1H)	69.37	H-5''	C-5''
1'''	4.92 (d, *J* = 3.9 Hz, 1H)	96.64	H-2'''	C-2'', C-3''', C-5'''
2'''	3.71-3.63 (m, 1H)	72.73	H-1''', H-3'''	C-3'''

3'''	3.66-3.58 (m, 1H)	79.94	H-2''', H-4'''	C-2''', C-4'''
4'''	3.65-3.56 (m, 1H)	81.56	H-3''', H-5'''	C-3''', C-5'''
5'''	4.40-3.32 (m, 1H)	62.15	H-4''', H-6'''	C-3''', C-6'''
6'''	4.23-4.16 (m, H-6a''', 1H);<br>3.69-3.61 (m, H-6b''', 1H)	68.77	H-5'''	C-4''', C-5'''

Table S8. NMR data of compound **31**

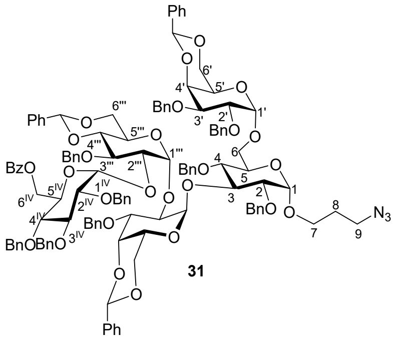

Position	^{1}H (ppm)	^{13}C (ppm)	^{1}H-^{1}H COSY	HMBC (H→C)
1	4.78 (d, *J* = 3.3 Hz, 1H)	96.04	H-2	C-3, C-5, C-7
2	3.47-3.39 (m, 1H)	79.44	H-1, H-3	C-3
3	4.44-4.37 (m, 1H）	73.53	H-2, H-4	C-2, C-4, C-1''
4	3.58-3.51 (m, 1H)	80.50	H-3, H-5	C-3, C-5
5	3.96-3.88 (m, 1H)	69.89	H-4, H-6	C-4, C-6
6	3.56-3.48 (m, 1H);<br>3.51-3.43 (m, 1H）	67.18	H-5	C-5, C-1'
7	3.72-3.64 (m, 1H);<br>3.40-3.32 (m, 1H)	64.47	H-1, H-8	C-1, C-8
8	1.77-1.67 (m, 2H)	28.83	H-7, H-9	C-7, C-8
9	3.33-3.23 (m, 2H)	48.29	H-8	C-7, C-8
1'	4.85 (d, *J* = 3.3 Hz, 1H)	98.38	H-2'	C-6, C-2'
2'	4.08-4.00 (m, 1H)	82.18	H-1', H-3'	C-3', C-4'
3'	3.98-3.90 (m, 1H)	75.86	H-2', H-4'	C-2'
4'	3.97-3.89 (m, 1H)		H-3', H-5'	C-3', C-5'
5'	3.55-3.48 (m, 1H）	62.69	H-4', H-6'	C-4', C-6'
6'	3.97-3.89 (m, H-6a, 1H)<br>4.16-4.08 (m, H-6b, 1H)	69.11	H-5'	C-5'
1''	5.86 (d, *J* = 3.3 Hz, 1H)	96.83	H-2''	C-3, C-5''
2''	4.55-4.47 (m, 1H)		H-1'', H-3''	C-3'', C-1'''
3''	4.02-3.94 (m, 1H)		H-2'', H-4''	C-1'', C-4''
4''	3.84-3.76 (m, 1H)	73.76	H-3'', H-5''	C-3'', C-5''

5"	4.20-4.12 (m, 1H)	62.05	H-4", H-6"	C-4"
6"	3.44-3.36 (m, H-6"a, 1H); 3.74-3.66 (m, H-6"b, 1H)	69.31	H-5"	C-5"
1'''	5.53 (d, J = 3.6 Hz, 1H)	95.51	H-2'''	C-2", C-5'''
2'''	3.82-3.74 (m, 1H)	74.80	H-1''', H-3'''	C-3'''
3'''	4.10-4.02 (m, 1H)	75.90	H-2''', H-4'''	C-2''', C-4'''
4'''	3.62-3.54 (m, 1H)	82.80	H-3''', H-5'''	C-3''', C-5'''
5'''	4.41-3.33 (m, 1H)	62.90	H-4''', H-6'''	C-3''', C-6'''
6'''	4.27-4.19 (m, H-6a, 1H); 4.14-4.06 (m, H-6b, 1H)	63.43	H-5'''	C-4''', C-5'''
1^{IV}	5.10 (1H)	94.65		
2^{IV}				
3^{IV}				
4^{V}				
5^{IV}	4.26-4.18 (m, 1H)	69.28	$H-6^{IV}$	$C-6^{IV}$
6^{IV}	4.33-4.23 (m, H-6a, H-6b, 2H)	69.53	$H-5^{IV}$	$C-5^{IV}$

Table S9. NMR data of compound **3**

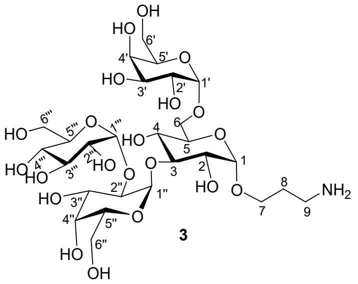

3

Position	^{1}H (ppm)	^{13}C (ppm)	^{1}H-^{1}H COSY	HMBC (H→C)
1	4.91 (d, J = 3.8 Hz, 1H)	98.50	H-2	C-2, C-3, C-7
2	3.68-3.60 (m, 1H)	69.30	H-1, H-3	C-1, C-3
3	3.83-3.75 (m, 1H)	80.32	H-2, H-4	C-2, C-4, C-5, C-1"
4	3.86-3.79 (m ,1H)	69.19	H-3, H-5	C-3, C-5
5	3.81-3.73 (m, 1H)	68.30	H-4, H-6	C-3, C-4
6	4.01-3.93 (m, 1H); 3.67-3.59 (m, 1H)	65.08	H-5	C-5, C-1'
7	3.94-3.86 (m, 1H); 3.62-3.54 (m, 1H)	65.93	H-1, H-8	C-1, C-8
8	1.97-1.87 (m, 2H)	26.40	H-7, H-9	C-7, C-8

9	3.07-2.97 (m, 2H)	37.72	H-8	C-8
1'	4.97-4.87 (d, *J* = 3.7 Hz, 1H)	97.99	H-2'	C-6, C-3', C-5'
2'	3.82-3.74 (m, 1H)	70.06	H-1', H-3'	C-3'
3'	3.70-3.62 (m ,1H)	69.50	H-2', H-4'	C-4'
4'	3.76-3.68 (m, 1H)	69.06	H-3', H-5'	C-3'. C-5'
5'	3.95-3.88 (m ,1H)	70.91	H-4', H-6'	C-4'
6'	3.74-3.66 (m, 2H)	61.01	H-5'	C-5'
1''	5.46 (d, *J* = 3.3 Hz, 1H)	96.75	H-2''	C-3, C-3'', C-5''
2''	3.98-3.90 (m ,1H)	72.47	H-1'', H-3''	C-3'', C-4'', C-1'''
3''	3.99-3.91 (m, 1H)	67.39	H-2'', H-4''	C-1'', C-2'', C-4''
4''	4.00 (dd, *J* = 3.0, 1.3 Hz, 1H)	69.10	H-3'', H-5''	C-3'', C-5''
5''	4.29-4.21 (m, 1H)	70.51	H-4''	C-4'', C-6''
6''	3.79-3.71 (m, 2H)	60.67	H-5''	C-4'', C-5''
1'''	5.12 (d, *J* = 3.9 Hz, 1H)	95.70	H-2'''	C-2'', C-3''', C-5'''
2'''	3.54 (dd, *J* = 9.8, 3.9 Hz, 1H)	71.26	H-1''', H-3'''	C-3'''
3'''	3.76-3.69 (m, 1H)	72.79	H-2''', H-4'''	C-2''', C-4'''
4'''	3.35 (dd, *J* =10.1, 9.2 Hz, 1H)	69.29	H-3''', H-5'''	C-3''', C-5'''
5'''	3.89-3.81 (m, 1H)	71.65	H-4''', H-6'''	C-3''', C-6'''
6'''	3.83-3.75 (m, 2H)	60.28	H-5'''	C-4''', C-5'''

Table S10. NMR data of compound **4**

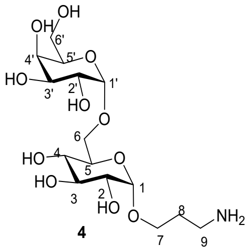

4

Position	^{1}H (ppm)	^{13}C (ppm)	^{1}H-^{1}H COSY	HMBC (H→C)
1	4.90 (d, *J* = 3.8 Hz, 1H)	98.26	H-2	C-3, C-5, C-7
2	3.60-3.52 (m, 1H)	70.89	H-1, H-3	C-3, C-4, C-6
3	3.64 (t, *J* = 9.4 Hz, 1H)	73.09	H-2, H-4	C-2, C-4
4	3.48 (t, *J* = 9.8 Hz, 1H)	69.22	H-3, H-5	C-3, C-5, C-6
5	3.82-3.74 (m, 1H)	70.30	H-4, H-6	C-6
6	3.98-3.90 (m, H-6a);<br>3.73-3.64 (m, H-6b)	65.42	H-5	C-4, C-5, C-1'
7	3.91-3.83 (m, H-7a);<br>3.61-3.53 (m, H-7b)	65.80	H-1, H-8	C-1, C-8, C-9
8	2.02-1.92 (m, 2H)	26.38	H-7, H-9	C-7, C-9

9	3.16-3.06 (m, 2H)	37.65	H-8	C-7, C-8
1'	4.93 (d, *J* = 3.6 Hz, 1H)	97.95	H-2'	C-3', C-4', C-6
2'	3.83-3.75 (m, 1H)	68.25	H-1', H-3'	C-3', C-4'
3'	3.85-3.77 (m, 1H)	69.34	H-2', H-4'	C-2', C-4'
4'	3.96-3.88 (m, 1H)	70.85	H-3', H-5'	C-1', C-3', C-5'
5'	3.98-3.90 (m, 1H)	69.07	H-4', H-6'	C-3', C-6'
6'	3.73-3.65 (m, 2H)	60.97	H-5'	C-4', C-5'

Table S11. NMR data of compound **1**

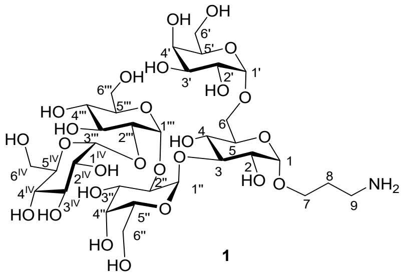

1

Position	^{1}H (ppm)	^{13}C (ppm)	^{1}H-^{1}H COSY	HMBC (H→C)
1	4.92 (d, *J* = 3.8 Hz, 1H)	98.32	H-2	C-3, C-5, C-7
2	3.76-3.68 (m ,1H)	69.36	H-1, H-3	C-3
3	3.98-3.90 (m, 1H)	77.15	H-2, H-4	C-1, C-2
4	3.88-3.80 (m, 1H)	70.34	H-3, H-5	C-6
5	3.73-3.65 (m, 1H)	70.37	H-4, H-6	C-1, C-6
6	3.76-3.68 (m, 2H)	65.77	H-5	C-5
7	3.94-3.86 (m, H-7a);<br>3.63-3.55 (m, H-7b)	65.94	H-8	C-1, C-7, C-8
8	2.03-1.93 (m, 2H)	26.43	H-7, H-9	C-7, C-9
9	3.18-3.10 (m, H-8a);<br>3.14-3.06 (m, H-8b)	37.79	H-8	C-7, C-8
1'	4.94 (d, *J* = 2.2 Hz, 1H)	98.16	H-2'	C-6, C-2', C-5'
2'	3.83-3.75 (m, 1H)	68.23	H-1', H-3'	C-1', C-3'
3'	3.50-3.43 (t, *J* = 9.5 Hz, 1H)	69.18	H-2', H-4'	C-2'
4'	3.88-3.80 (m, 1H)	69.36	H-3', H-5'	C-5'
5'	3.95-3.87 (m, 1H)	71.03	H-4', H-6'	C-1', C-6'
6'	3.78-3.70 (m, 2H)	60.17	H-5'	C-4', C-5'
1''	5.69 (d, *J* = 3.8 Hz, 1H)	95.20	H-2''	C-3, C-2'', C-3'', C-5''
2''	3.97-3.89 (m, 1H)	72.52	H-1'', H-3''	C-3''

3''	4.03 (dd, *J* = 9.3, 3.3 Hz, 1H)	67.59	H-2'', H-4''	C-2'', C-4''
4''	3.99 (d, *J* = 3.3 Hz, 1H)	69.10	H-3'', H-5''	C-3'', C-4''
5''	4.23 (t, *J* = 6.4 Hz, 1H)	70.56	H-4'', H-6''	C1'', C-4'', C-6''
6''	3.74-3.66 (m, 2H)	61.04	H-5''	C-4'', C-5''
1'''	5.10 (d, *J* = 3.8 Hz, 1H)	95.82	H-2'''	C-2'', C-3''', C-5'''
2'''	3.51 (dd, *J* = 9.9, 3.8 Hz, 1H)	71.43	H-1''', H-3'''	C-3''', C-4''', C-1 IV
3'''	3.80-3.72 (m, 1H)	72.81	H-2''', H-4'''	C-2'', C-4'', C-5''
4'''	3.45-3.67 (t, *J* = 9.7 Hz, 1H)	69.34	H-3''', H-5'''	C-3''', C-5''', C-6'''
5'''	3.96-3.88 (m, 1H)	71.82	H-4''', H-6'''	C-1'', C-4'', C-6''
6'''	3.85-3.77 (m, 2H)	60.29	H-5'''	C-4'', C-5''
1 IV	5.39 (d, *J* = 3.5 Hz, 1H)	92.80	H-2 IV	C-2''', C-5 IV
2 IV	3.73-3.65 (m, 1H)	74.61	H-1 IV, H-3 IV	C-3 IV
3 IV	3.89-3.81 (m, 1H)	71.00	H-2 IV, H-4 IV	C-2 IV, C-4 IV
4 IV	3.99-3.91 (m, 1H)	69.10	H-3 IV, H-5 IV	C-3 IV
5 IV	3.96-3.88 (m, 1H)	71.81	H-4 IV, H-6 IV	C-4 IV, C-6 IV
6 IV	3.75-3.68 (m, 2H)	61.13	H-5 IV	C-4 IV, C-5 IV

Table S12. NMR data of compound **2**

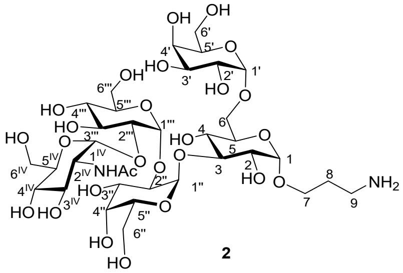

2

Position	^{1}H (ppm)	^{13}C (ppm)	^{1}H-^{1}H COSY	HMBC (H→C)
1	4.86 (d, J = 3.9 Hz, 1H)	98.35	H-2	C-3, C-5, C-7
2	3.65-3.58 (m, 1H)	69.36	H-1, H-3	C-1, C-3
3	3.91-3.83 (m, 1H)	76.23	H-2, H-4	C-1", C-2, C-4
4	3.40-3.32 (m, 1H)	71.10	H-3, H-5	C-3, C-5
5	3.81-3.73 (m, 1H)	70.36	H-4, H-6	C-4
6	3.87-3.79 (m, H-6a);<br>3.75-3.67 (m, H-6b)	66.18	H-5	C-5, C-1'
7	3.88-3.80 (m, H-7b);<br>3.59-3.51 (m, H-7a)	65.80	H-8	C-1, C-7, C-8
8	1.99-1.89 (m, 2H)	26.41	H-7, H-9	C-7, C-9
9	3.14-3.04 (m, 2H)	37.72	H-8	C-7, C-8
1'	4.90 (d, J = 3.9 Hz)	98.28	H-2'	C-6, C-2', C-3'
2'	3.79-3.71 (m, 1H)	70.90	H-1', H-3'	C-3'
3'	3.70-3.63 (m, 1H)	69.47	H-2', H-4'	C-2'
4'	3.96-3.88 (m, 1H)	68.86	H-3', H-5'	C-2', C-3'
5'	3.93-3.85 (m, 1H)	68.27	H-4', H-6'	C-1', C-6'
6'	3.70-3.62 (m, 2H)	61.09	H-5'	C-5'
1"	5.59 (d, J = 3.6 Hz, 1H)	94.96	H-2"	C-3", C-5"
2"	3.89-3.81 (m, 1H)	71.84	H-1", H-3"	C-3"
3"	3.85-3.78 (m, 1H)	67.91	H-2", H-4"	C-4"
4"	3.99-3.91 (m, 1H)	68.77	H-3", H-5"	C-3"
5"	4.33 (t, J = 6.4 Hz, 1H)	70.11	H-4", H-6"	C1", C-4", C-6"
6"	3.69-3.61 (m, 2H)	60.89	H-5"	C-5"
1'''	5.53(d, J = 3.7 Hz, 1H)	90.16	H-2'''	C-2"
2'''	3.69-3.61 (m, 1H)	72.90	H-1''', H-3'''	C-1v
3'''	3.87-3.79 (m, 1H)	71.40	H-2''', H-4'''	C-5"
4'''	3.48-3.40 (m, 1H)	69.11	H-3''', H-5'''	C-3''', C-6'''
5'''	3.70-3.62 (m, 1H)	68.77	H-4''', H-6'''	C-3", C-4", C-6"
6'''	3.83-3.75 (m, H-6'''a);	60.12	H-5'''	C-4", C-5"

	3.74-3.66 (m, H-6'''b)			
1^{IV}	5.04 (d, J = 3.7 Hz, 1H)	93.03	H-2^{IV}	C-2''', C-5^{IV}
2^{IV}	3.93-3.85 (m, 1H)	70.96	H-1^{IV}, H-3^{IV}	C-3^{IV}
3^{IV}	3.76-3.68 (m,1H)	72.09	H-2^{IV}, H-4^{IV}	C-2^{IV}
4^{IV}	3.39-3.31 (m ,1H)	69.68	H-3^{IV}, H-5^{IV}	
5^{IV}	3.84-3,76 (m, 1H)	69.88	H-4^{IV}, H-6^{IV}	C-4^{IV}, C-6^{IV}
6^{IV}	3.87-3.79 (m, 2H)	60.65	H-5^{IV}	C-5^{IV}

References:

1. C. E. Doneanu, W. Chen and J. C. Gebler, *Anal. Chem.*, 2009, **81**, 3485-3499.
2. K. Bock and C. Pedersen, *J. Chem. Soc., Perkin Trans. 2*, 1974, 293-297.
3. C. W. Chen, C. C. Wang, X. R. Li, H. Witek and K. T. Mong, *Org. Biomol. Chem.*, 2020, **18**, 3135-3141.
4. C. C. Wang, J. C. Lee, S. Y. Luo, S. S. Kulkarni, Y. W. Huang, C. C. Lee, K. L. Chang and S. C. Hung, *Nature*, 2007, **446**, 896-899.
5. Q. Zhao, H. Zhang, Y. Zhang, S. Zhou and J. Gao, *Org. Biomol. Chem.*, 2020, **18**, 6549-6557.
6. Z. Y. Zhang, I. R. Ollmann, X. S. Ye, R. Wischnat, T. Baasov and C. H. Wong, *J. Am. Chem. Soc.*, 1999, **121**, 734-753.
7. D. K. Njeri and J. R. Ragains, *Eur. J. Org. Chem.*, 2022, **2022**, e202201261.
8. Y. Hsu, X.-A. Lu, M. M. L. Zulueta, C.-M. Tsai, K.-I. Lin, S.-C. Hung and C.-H. Wong, *J. Am. Chem. Soc.*, 2012, **134**, 4549-4552.
9. Y. Pan, Q. Q. He, N. Wimmer and V. Ferro, *Carbohydr. Res.*, 2024, **545**, 109270.
10. Citation | Gaussian.com. https://gaussian.com/citation/ (accessed 2023-11-17).
11. A. D. Becke, *Phys Rev A*, 1988, **38**, 3098-3100.
12. J. P. Perdew, *Phys Rev B*, 1986, **33**, 8822-8824.
13. S. M. Tennant, J. Y. Wang, J. E. Galen, R. Simon, M. F. Pasetti, O. Gat and M. M. Levine, *Infect. Immun.*, 2011, **79**, 4175-4185.
14. R. Simon, S. M. Tennant, J. Y. Wang, P. J. Schmidlein, A. Lees, R. K. Ernst, M. F. Pasetti, J. E. Galen and M. M. Levine, *Infect. Immun.*, 2011, **79**, 4240-4249.
15. D. Dhara, S. M. Baliban, C. X. Huo, Z. Rashidijahanabad, K. T. Sears, S. T. Nick, A. K. Misra, S. M. Tennant and X. Huang, *Chem. Eur. J.*, 2020, **26**, 15953-15968.
16. F. J. Fuche, J. A. Jones, G. Ramachandran, E. E. Higginson, R. Simon and S. M. Tennant, *Hum. Vaccines Immunother.*, 2019, **15**, 1427-1435.
17. T. C. Darton, S. M. Baliban, B. Curtis, D. Toema, S. M. Tennant, M. M. Levine, M. F. Pasetti and R. Simon, *PLoS Negl. Trop. Dis.*, 2018, **12**, e0006522.

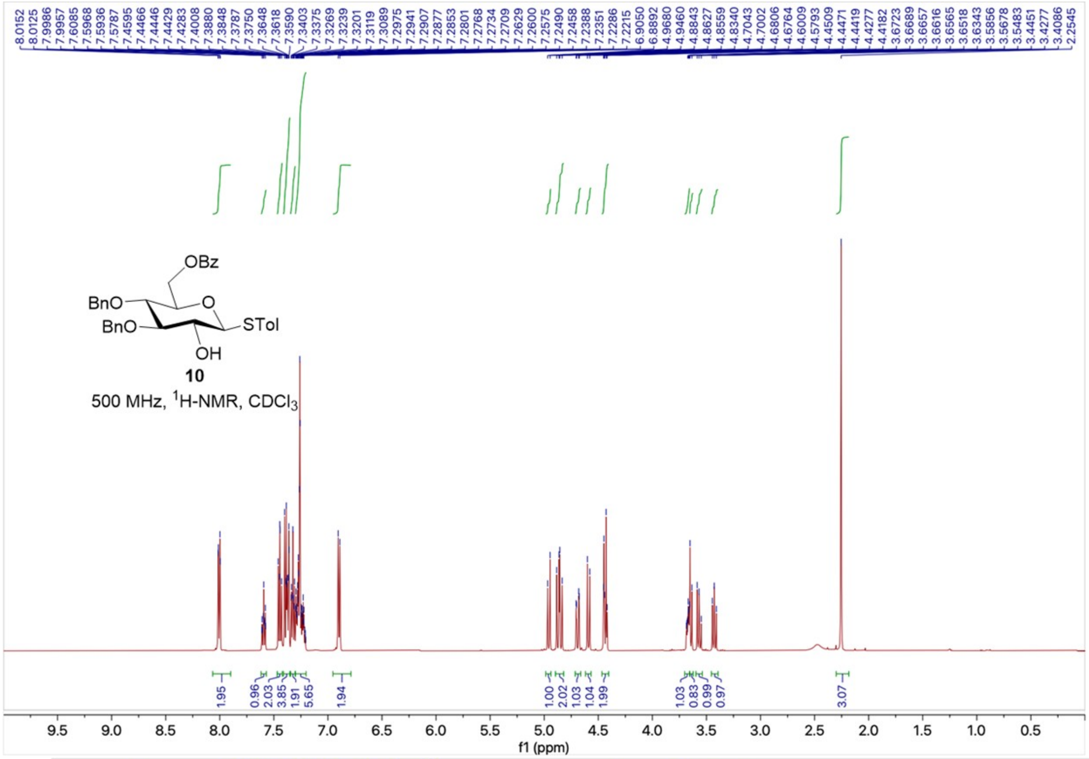
OBz
BnO
BnO
O
STol
OH
10
500 MHz, 1H-NMR, CDCl3
f1 (ppm)

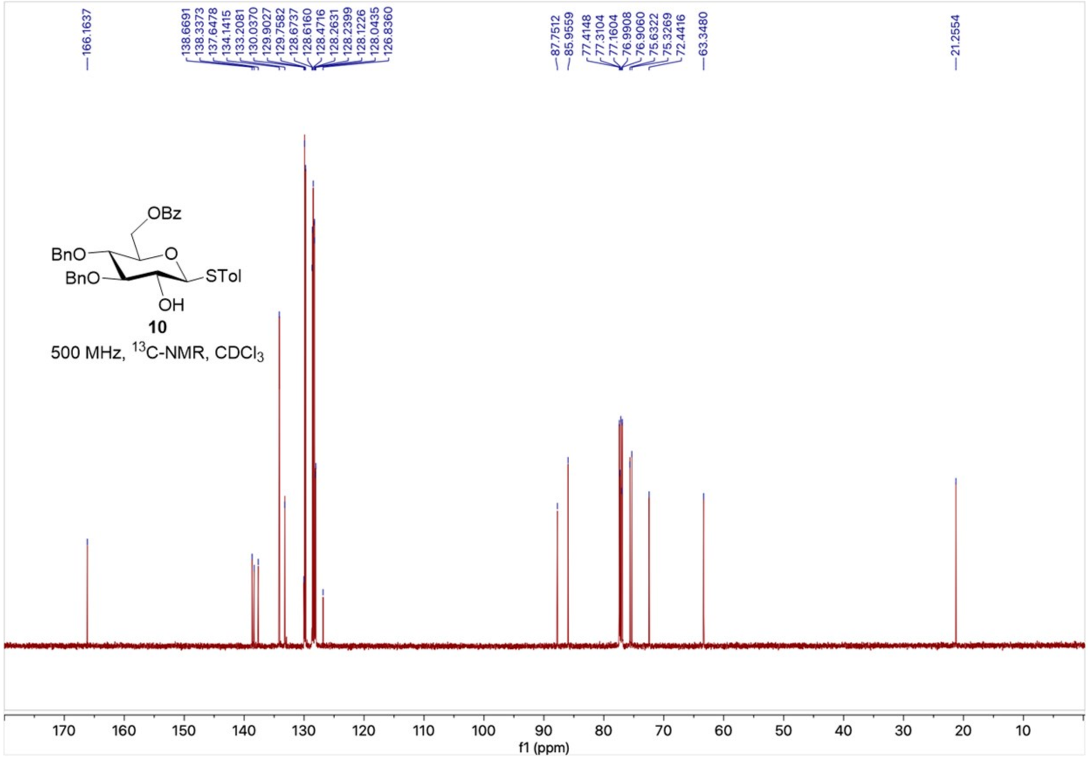
OBz
BnO
BnO
O
STol
OH
10
500 MHz, 13C-NMR, CDCl3
f1 (ppm)

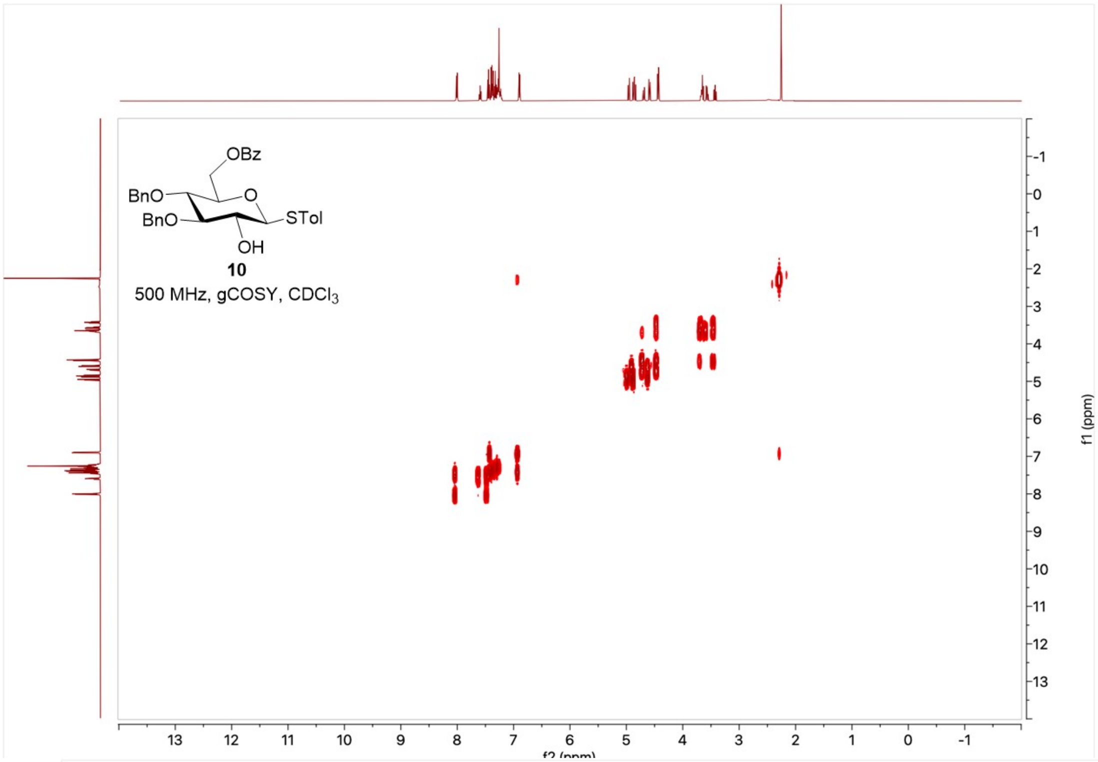

OBz
BnO
O
BnO
STol
OH
10
500 MHz, gCOSY, CDCl3
f1 (ppm)
f2 (ppm)

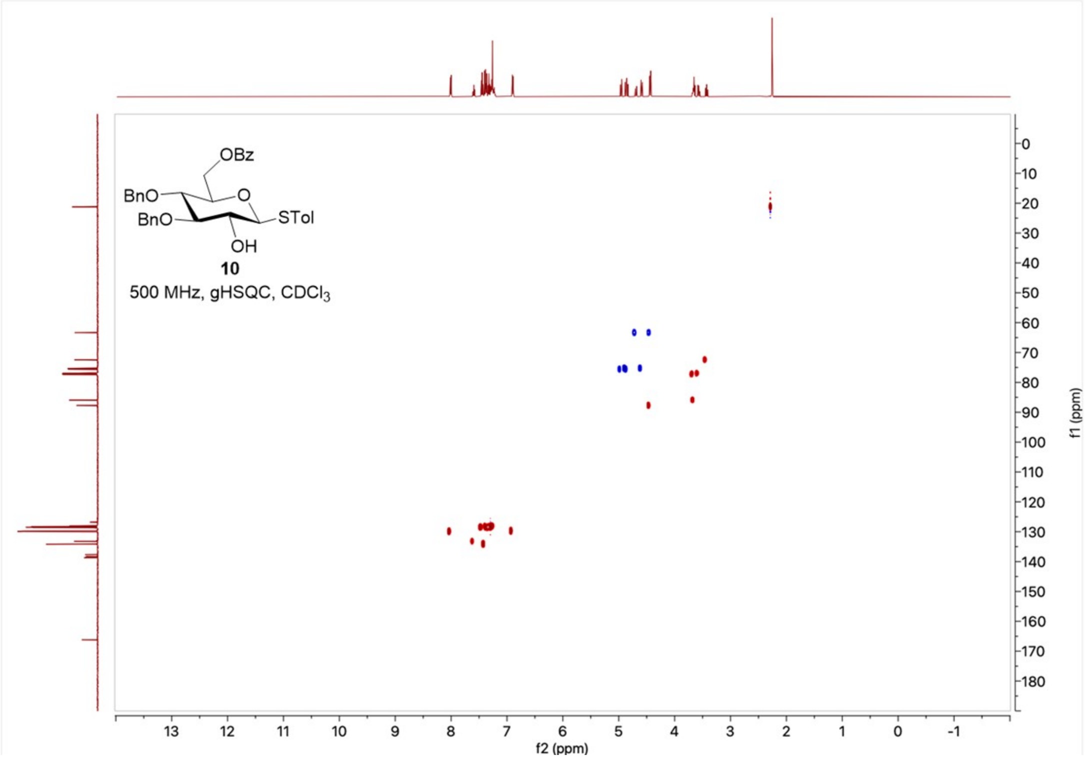

OBz
BnO
O
BnO
STol
OH
10
500 MHz, gHSQC, CDCl3
f1 (ppm)
f2 (ppm)

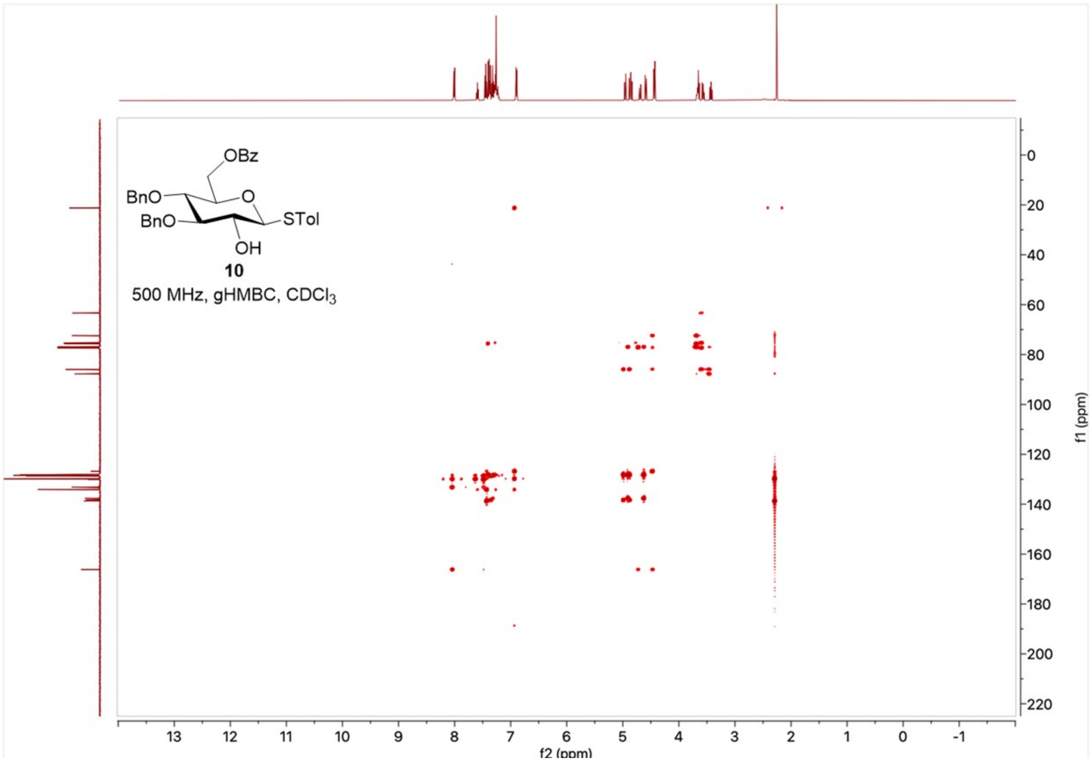
OBz
BnO
BnO
O
STol
OH
10
500 MHz, gHMBC, CDCl3
f1 (ppm)
f2 (ppm)

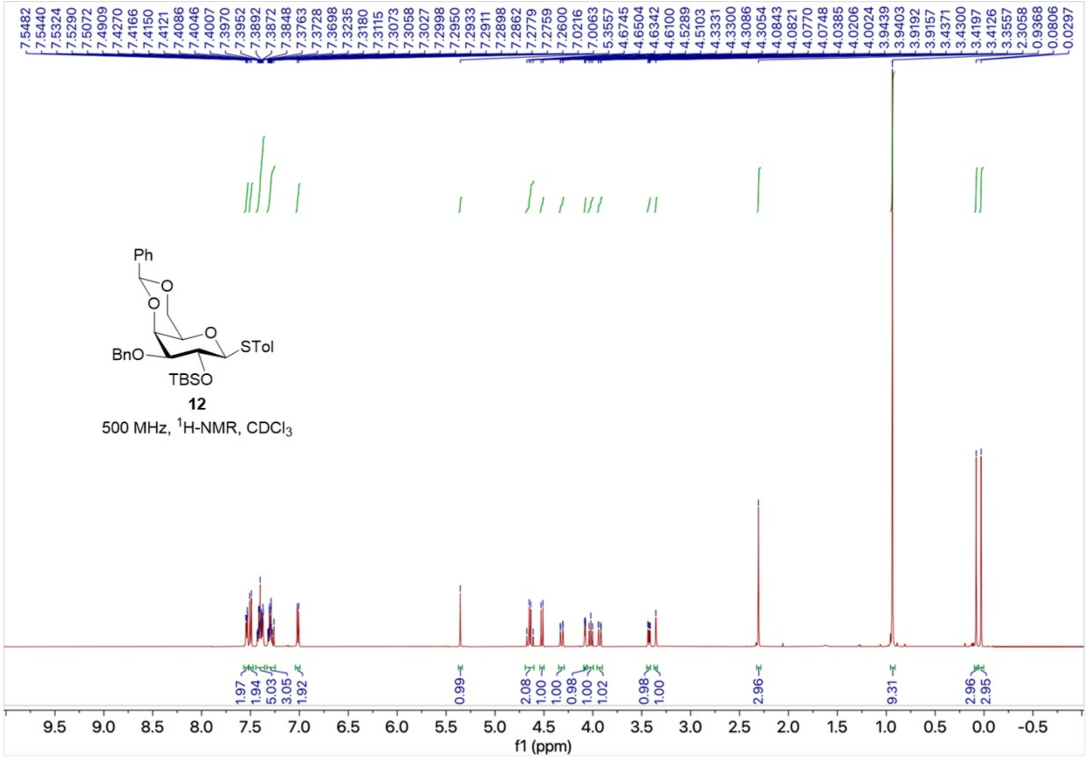

7.5482 7.5440 7.5324 7.5290 7.5072 7.4909 7.4270 7.4166 7.4150 7.4121 7.4086 7.4046 7.4007 7.3970 7.3952 7.3892 7.3872 7.3848 7.3763 7.3728 7.3698 7.3235 7.3180 7.3115 7.3073 7.3058 7.3027 7.2998 7.2950 7.2933 7.2911 7.2898 7.2862 7.2779 7.2759 7.2600 7.0216 7.0063 5.3557 4.6745 4.6504 4.6342 4.6100 4.5289 4.5103 4.3331 4.3300 4.3086 4.3054 4.0843 4.0821 4.0770 4.0748 4.0385 4.0206 4.0024 3.9439 3.9403 3.9192 3.9157 3.4371 3.4300 3.4197 3.4126 3.3557 2.3058 0.9368 0.0806 0.0297
Ph
O
O
BnO
O
STol
TBSO
12
500 MHz, ^{1}H-NMR, $CDCl_3$
1.97 1.94 5.03 3.05 1.92 0.99 2.08 1.00 1.00 0.98 1.00 1.02 0.98 1.00 2.96 9.31 2.96 2.95
9.5 9.0 8.5 8.0 7.5 7.0 6.5 6.0 5.5 5.0 4.5 4.0 3.5 3.0 2.5 2.0 1.5 1.0 0.5 0.0 -0.5
f1 (ppm)

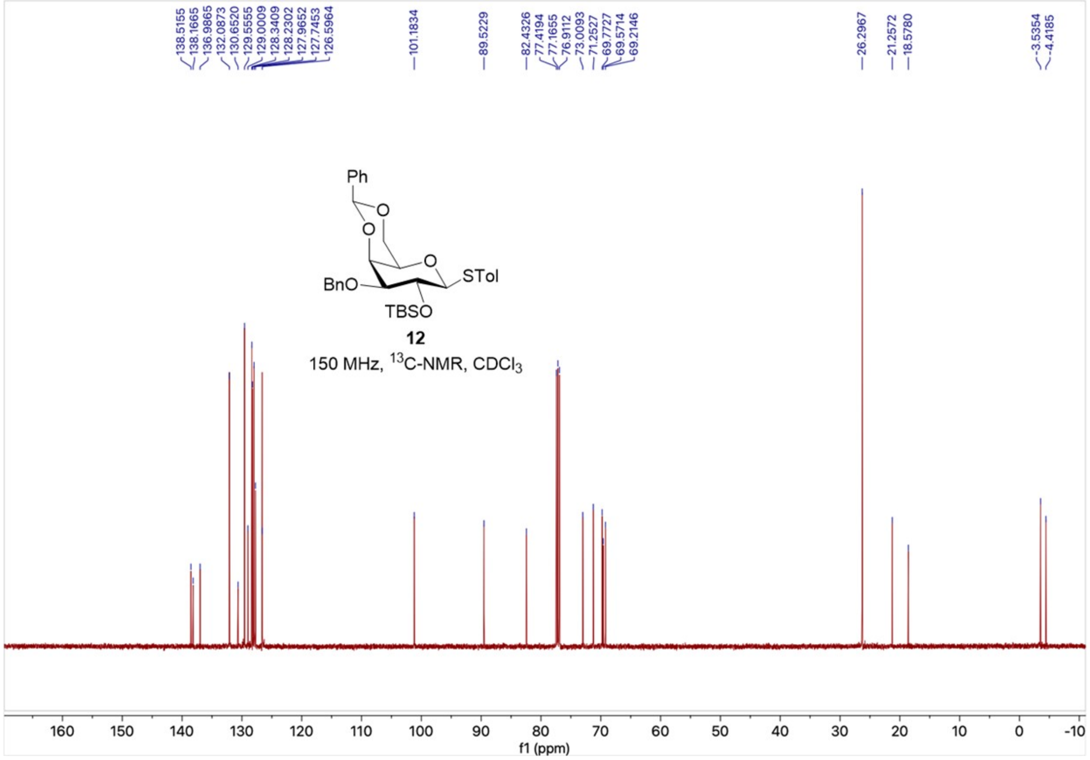

138.5155 138.1665 136.9865 132.0873 130.6520 129.5555 129.0009 128.3409 128.2302 127.9652 127.7453 126.5964 101.1834 89.5229 82.4326 77.4194 77.1655 76.9112 73.0093 71.2527 69.7727 69.5714 69.2146 26.2967 21.2572 18.5780 -3.5354 -4.4185
Ph
O
O
BnO
O
STol
TBSO
12
150 MHz, ^{13}C-NMR, $CDCl_3$
160 150 140 130 120 110 100 90 80 70 60 50 40 30 20 10 0 -10
f1 (ppm)

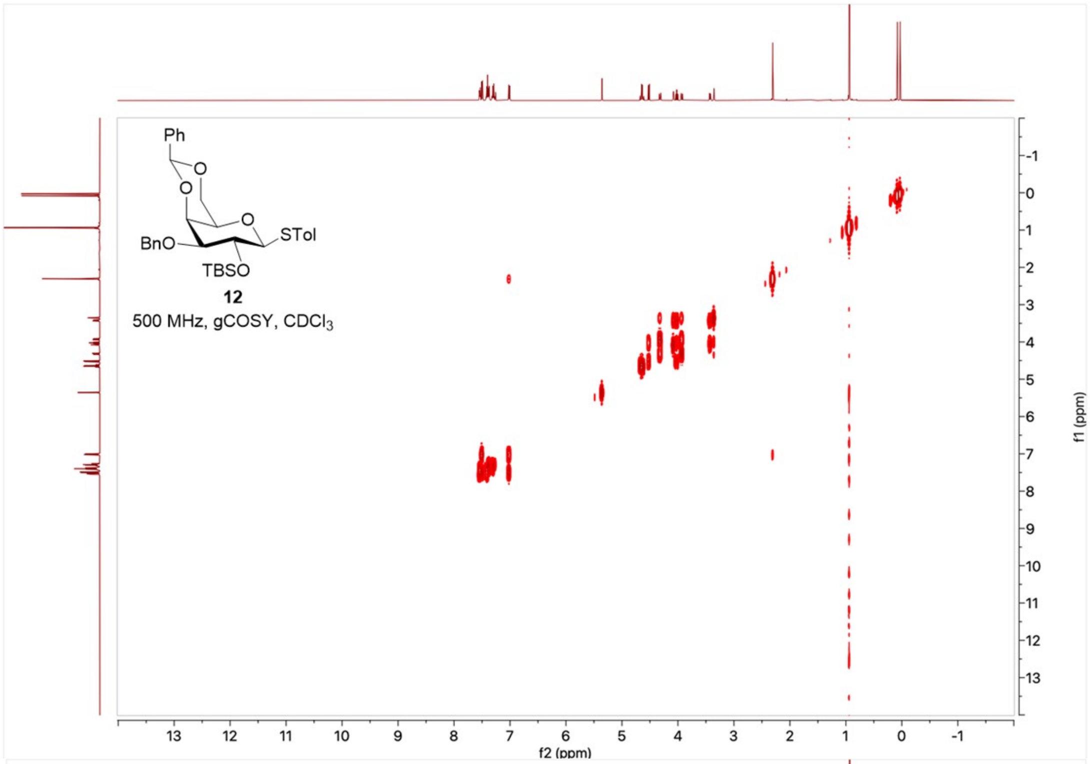

Ph
O
O
O
STol
BnO
TBSO
12
500 MHz, gCOSY, CDCl3
f1 (ppm)
f2 (ppm)

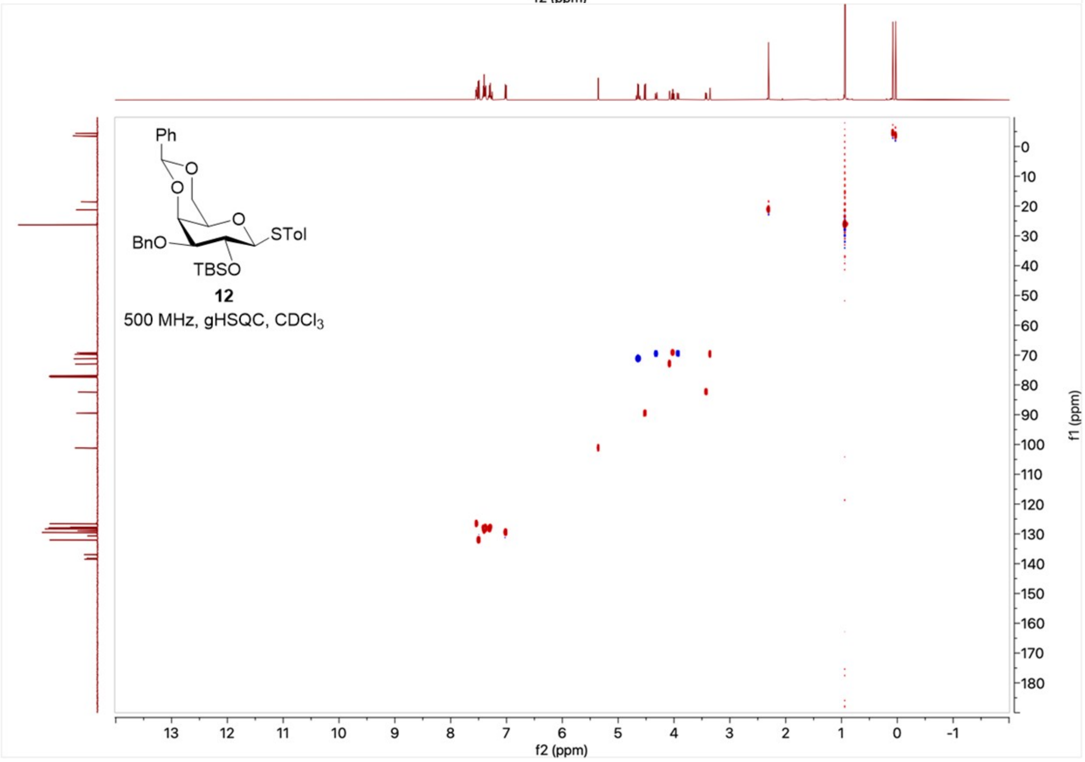

Ph
O
O
O
STol
BnO
TBSO
12
500 MHz, gHSQC, CDCl3
f1 (ppm)
f2 (ppm)

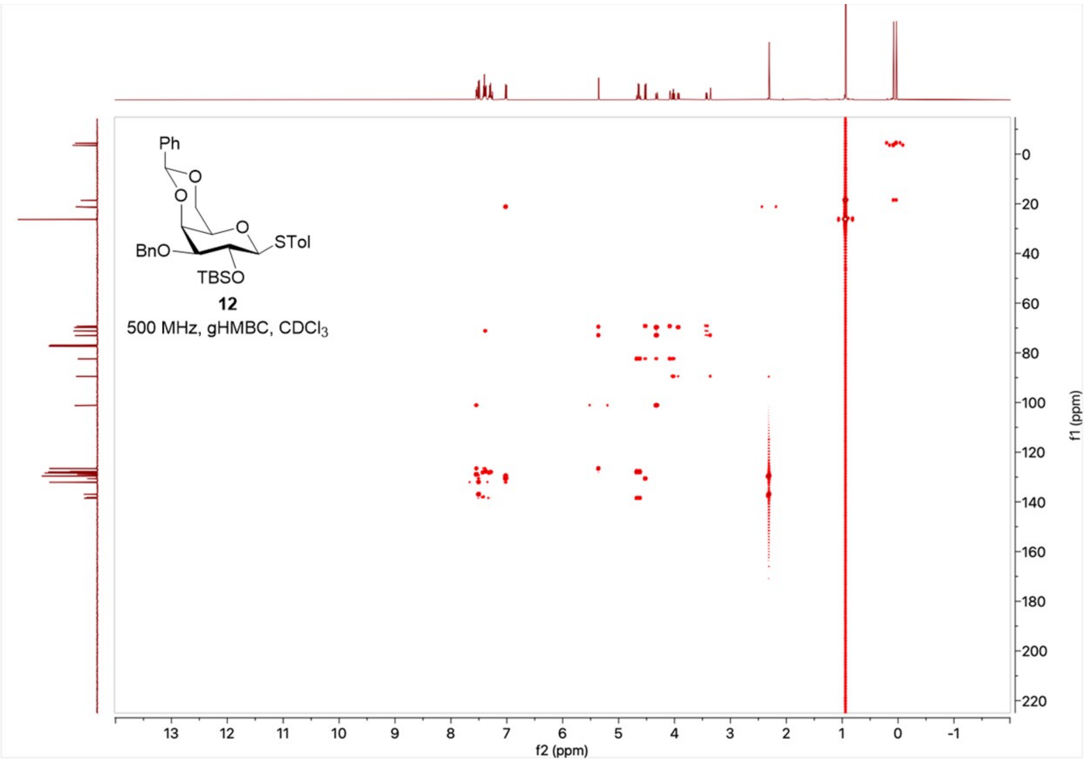
Ph
O
O
O
STol
BnO
TBSO
12
500 MHz, gHMBC, CDCl3
f1 (ppm)
f2 (ppm)

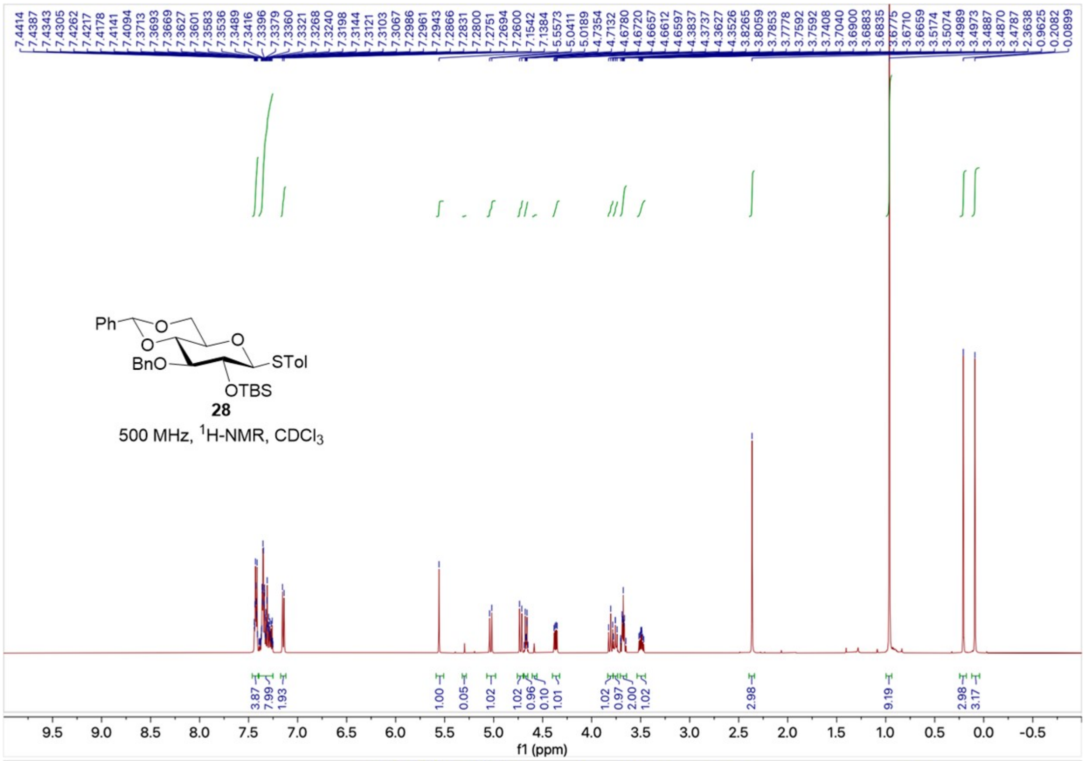

Ph
O
O
O
BnO
STol
OTBS
28
500 MHz, ¹H-NMR, CDCl₃
f1 (ppm)

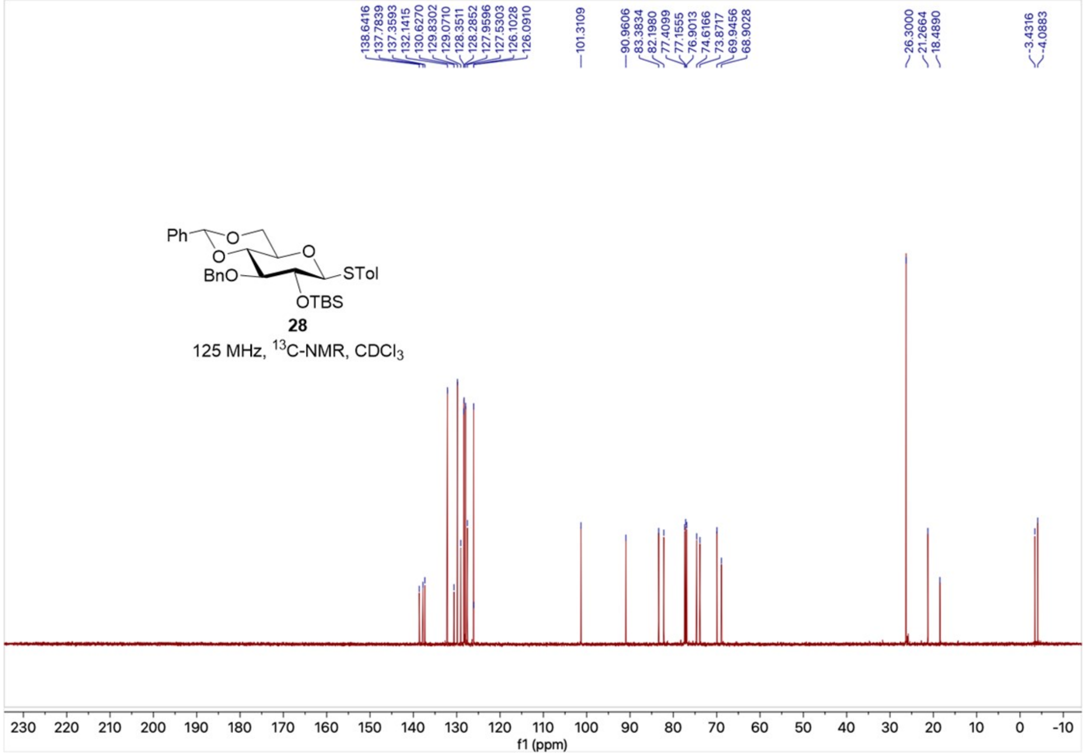

Ph
O
O
O
BnO
STol
OTBS
28
125 MHz, ¹³C-NMR, CDCl₃
f1 (ppm)

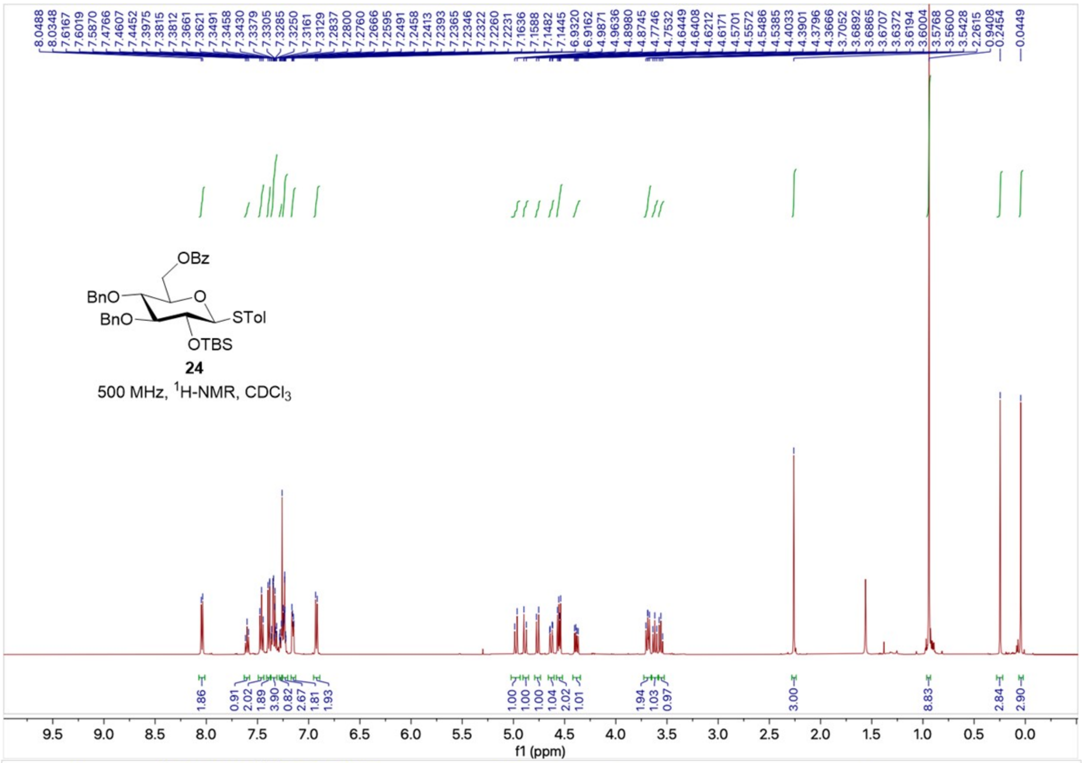
24
500 MHz, 1H-NMR, CDCl3
f1 (ppm)

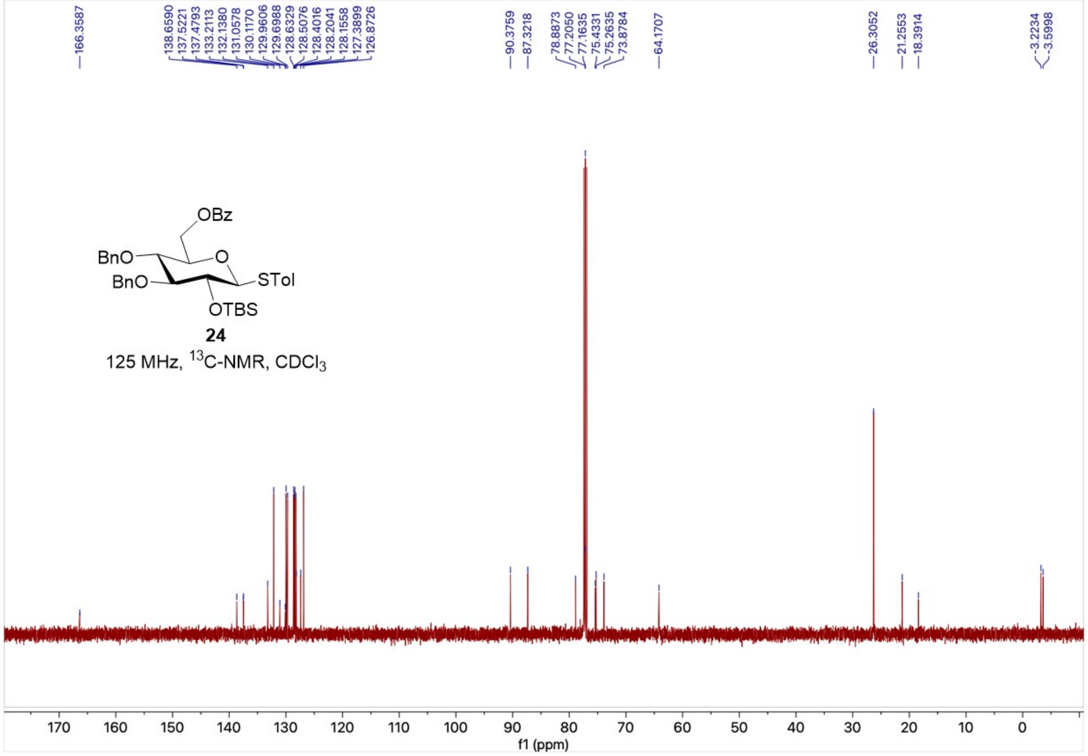
24
125 MHz, 13C-NMR, CDCl3
f1 (ppm)

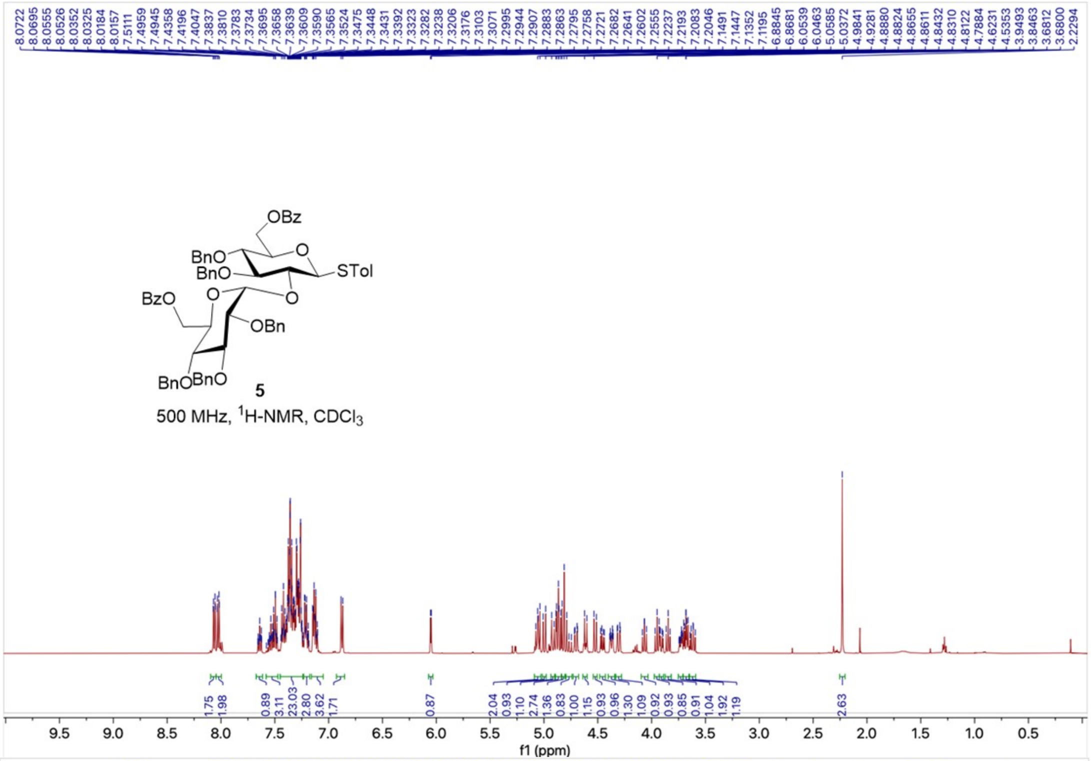
5
500 MHz, ¹H-NMR, CDCl3
f1 (ppm)

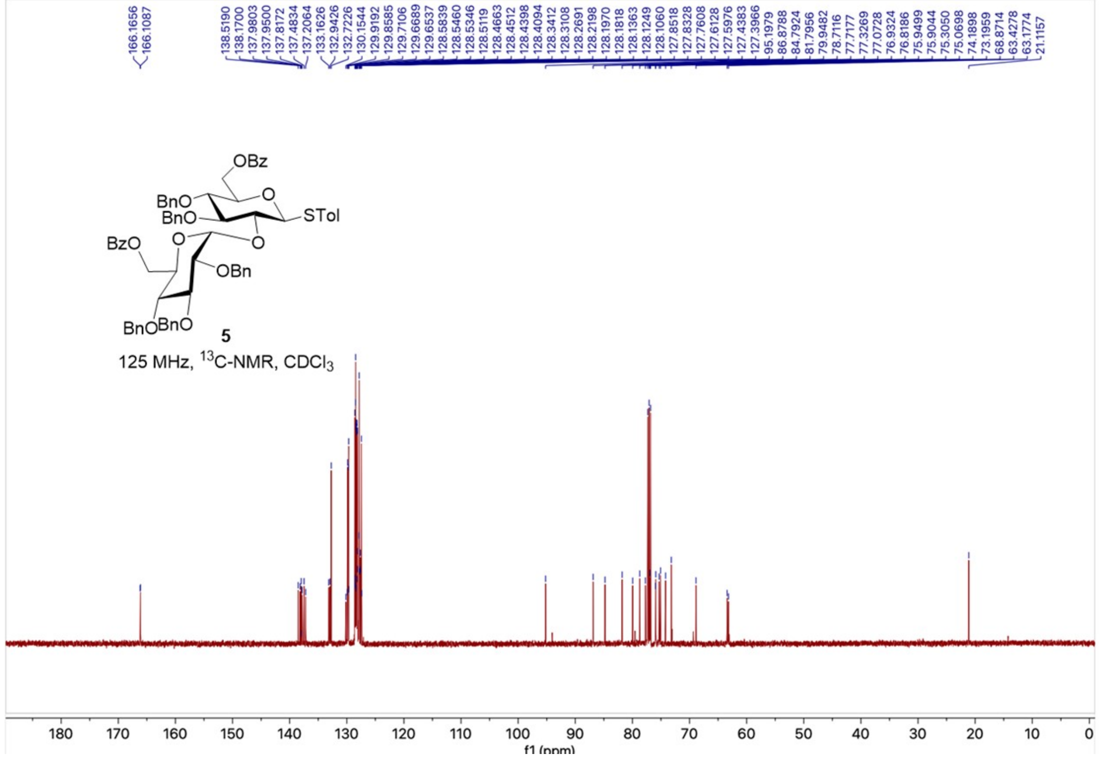
5
125 MHz, ¹³C-NMR, CDCl3
f1 (ppm)

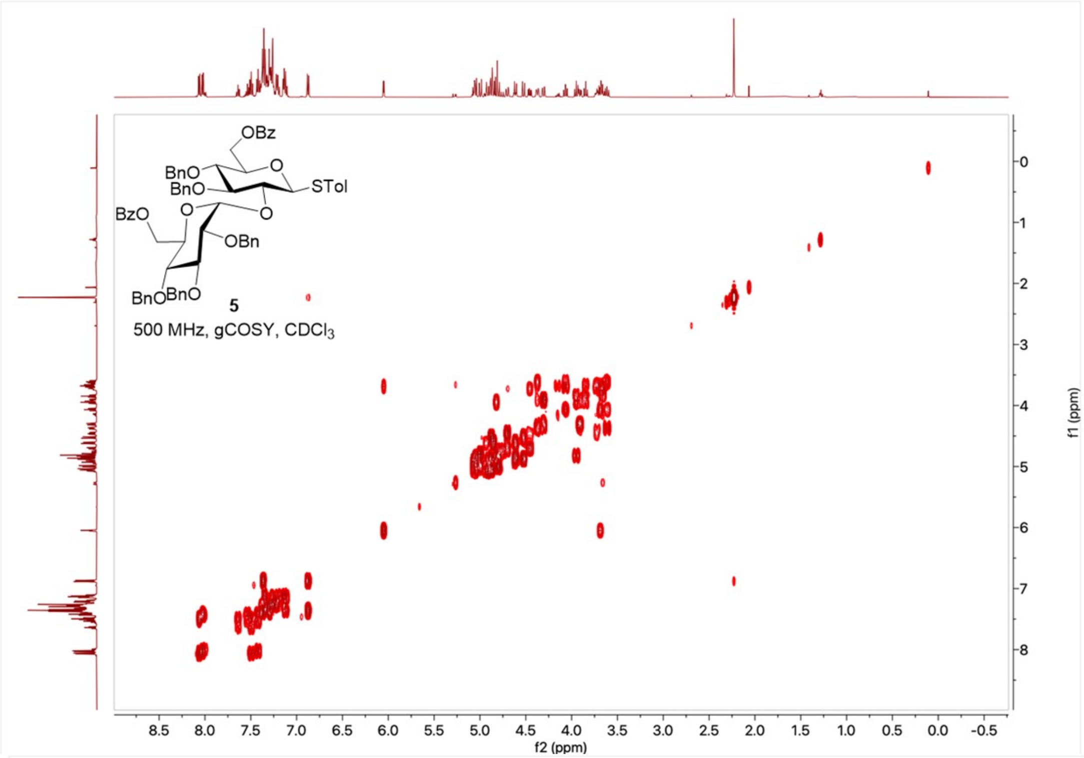
OBz
BnO
BnO
STol
BzO
OBn
BnO BnO
5
500 MHz, gCOSY, CDCl3
f1 (ppm)
f2 (ppm)

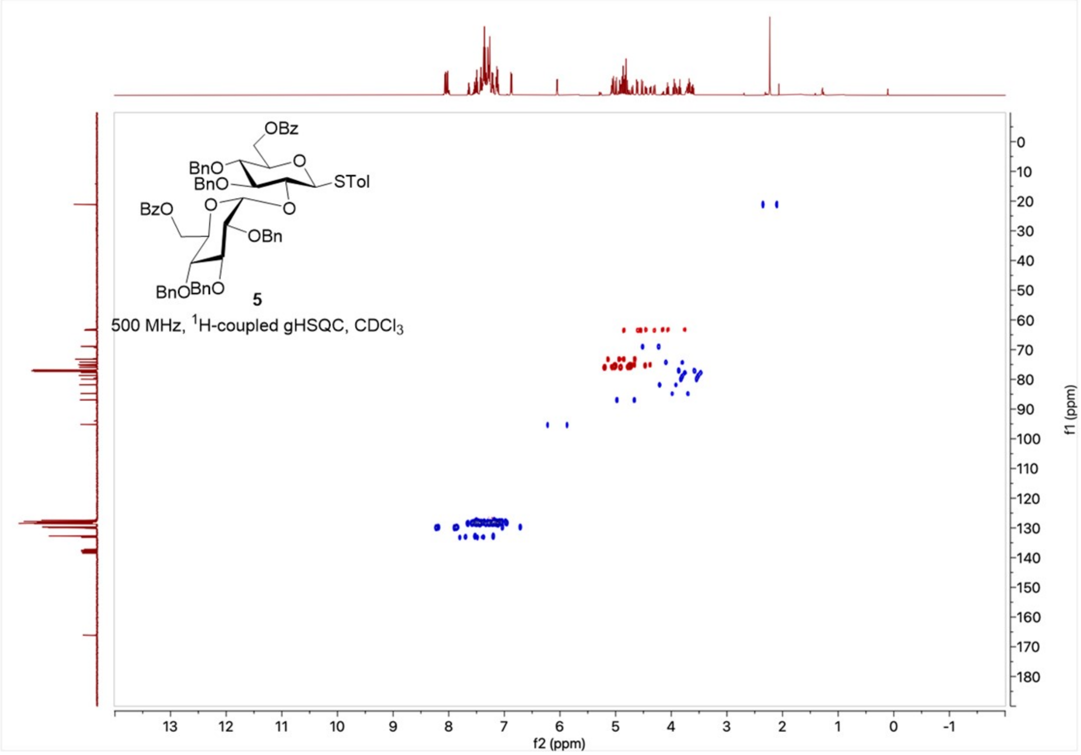
OBz
BnO
BnO
STol
BzO
OBn
BnO BnO
5
500 MHz, 1H-coupled gHSQC, CDCl3
f1 (ppm)
f2 (ppm)

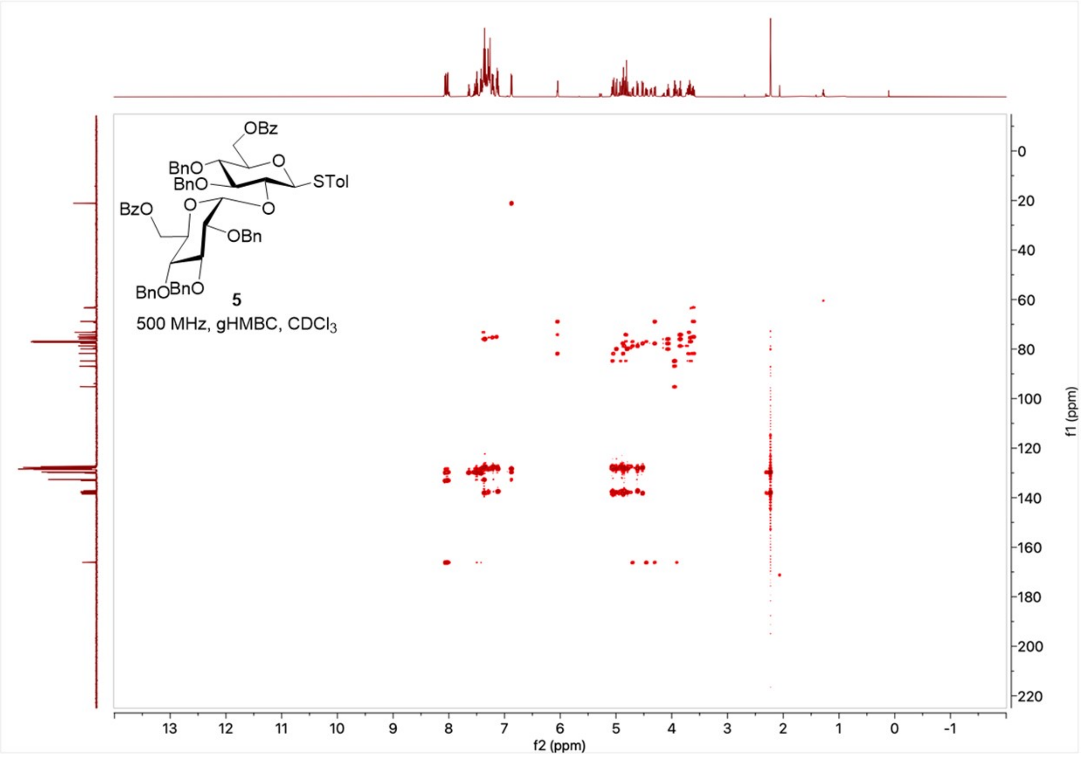
OBz
BnO
BnO
STol
BzO
OBn
BnO
BnO
5
500 MHz, gHMBC, CDCl3
f1 (ppm)
f2 (ppm)

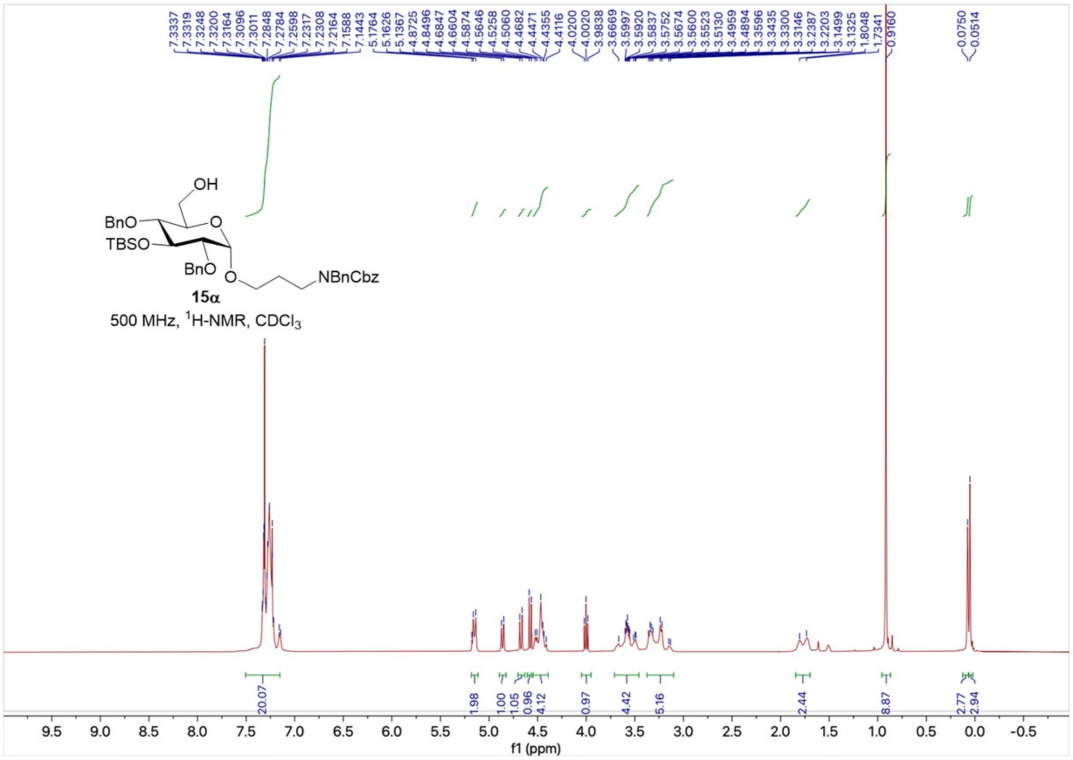

7.3337
7.3319
7.3248
7.3200
7.3164
7.3096
7.3011
7.2848
7.2784
7.2598
7.2317
7.2308
7.2164
7.1588
7.1443
5.1764
5.1626
5.1367
4.8725
4.8496
4.6847
4.6604
4.5874
4.5646
4.5258
4.5060
4.4682
4.4471
4.4355
4.4116
4.0200
4.0020
3.9838
3.6669
3.5997
3.5920
3.5837
3.5752
3.5674
3.5600
3.5523
3.5130
3.4959
3.4894
3.3596
3.3435
3.3300
3.3146
3.2387
3.2203
3.1499
3.1325
1.8048
1.7341
0.9160
0.0750
0.0514
OH
BnO
TBSO
BnO
O
O
NBnCbz
15α
500 MHz, ¹H-NMR, CDCl₃
20.07
1.98
1.00
1.05
0.96
4.12
0.97
4.42
5.16
2.44
8.87
2.77
2.94
f1 (ppm)

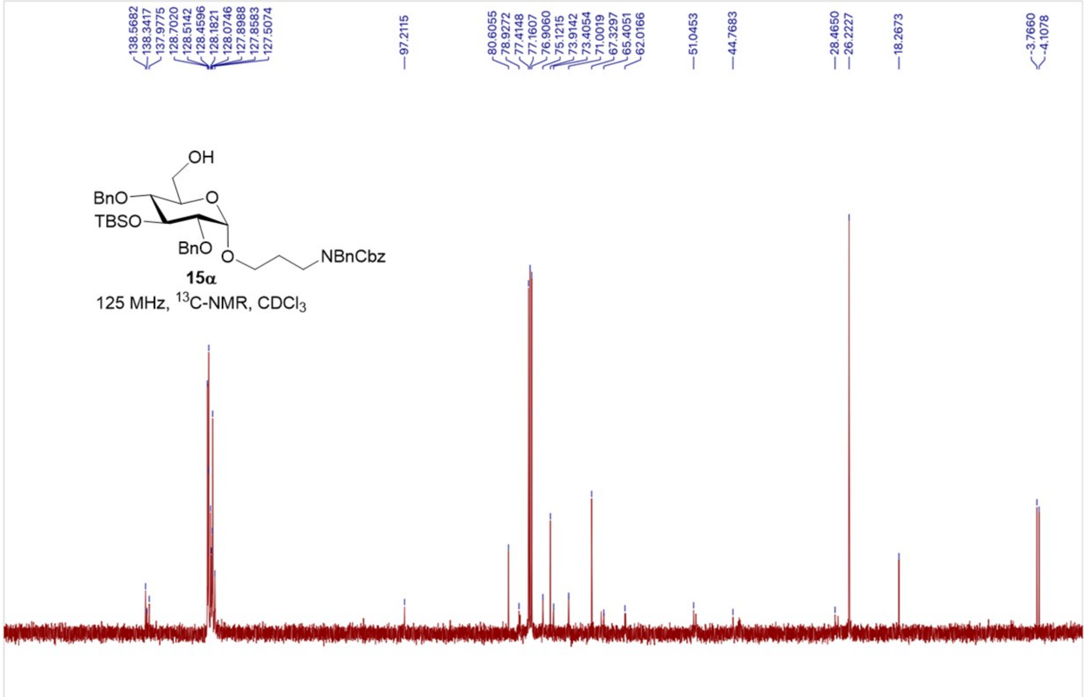

138.5682
138.3417
137.9775
128.7020
128.5142
128.4596
128.1821
128.0746
127.8988
127.8583
127.5074
97.2115
80.6055
78.9272
77.4148
77.1607
76.9060
75.1215
73.9142
73.4054
71.0019
67.3297
65.4051
62.0166
51.0453
44.7683
28.4650
26.2227
18.2673
-3.7660
-4.1078
OH
BnO
TBSO
BnO
O
O
NBnCbz
15α
125 MHz, ¹³C-NMR, CDCl₃
f1 (ppm)

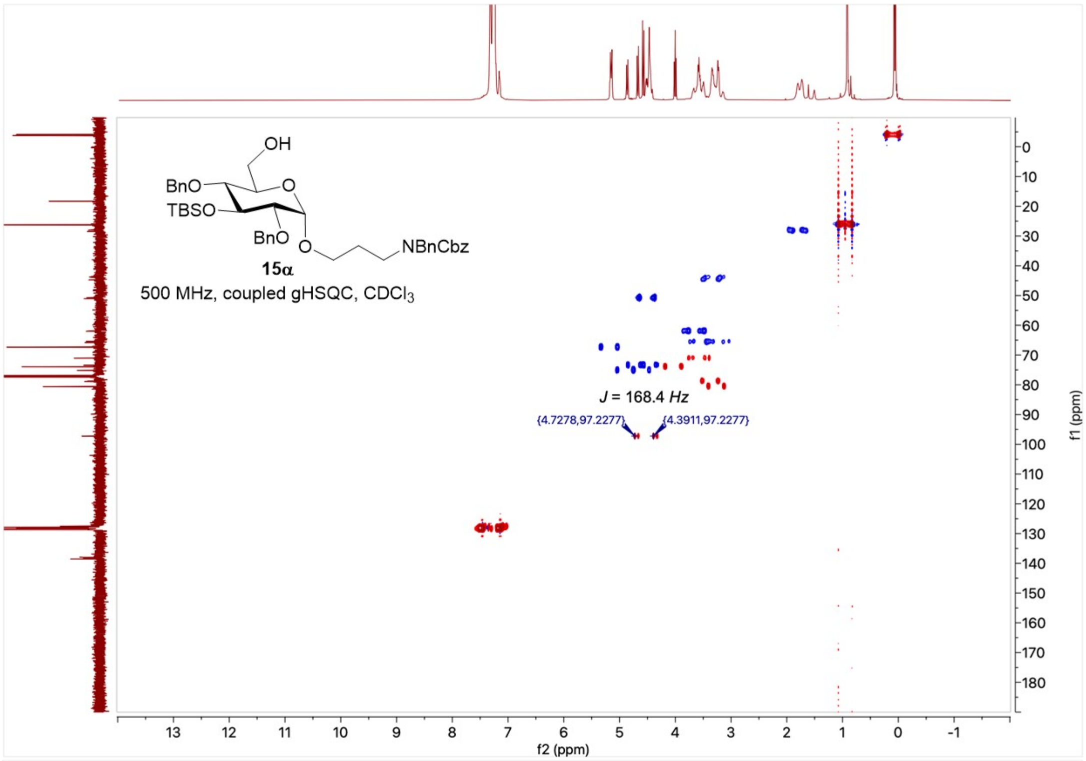

OH
BnO
TBSO
BnO
O
NBnCbz
15α
500 MHz, coupled gHSQC, CDCl3
J = 168.4 Hz
{4.7278,97.2277}
{4.3911,97.2277}
f2 (ppm)
f1 (ppm)

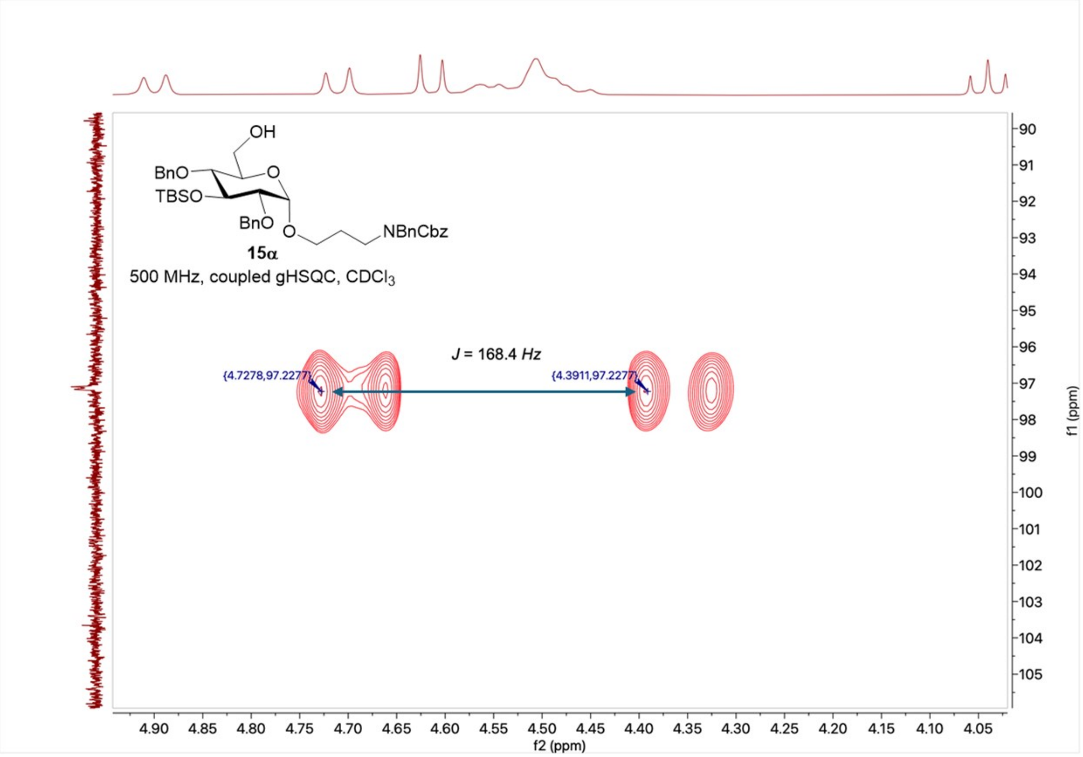

OH
BnO
TBSO
BnO
O
NBnCbz
15α
500 MHz, coupled gHSQC, CDCl3
J = 168.4 Hz
{4.7278,97.2277}
{4.3911,97.2277}
f2 (ppm)
f1 (ppm)

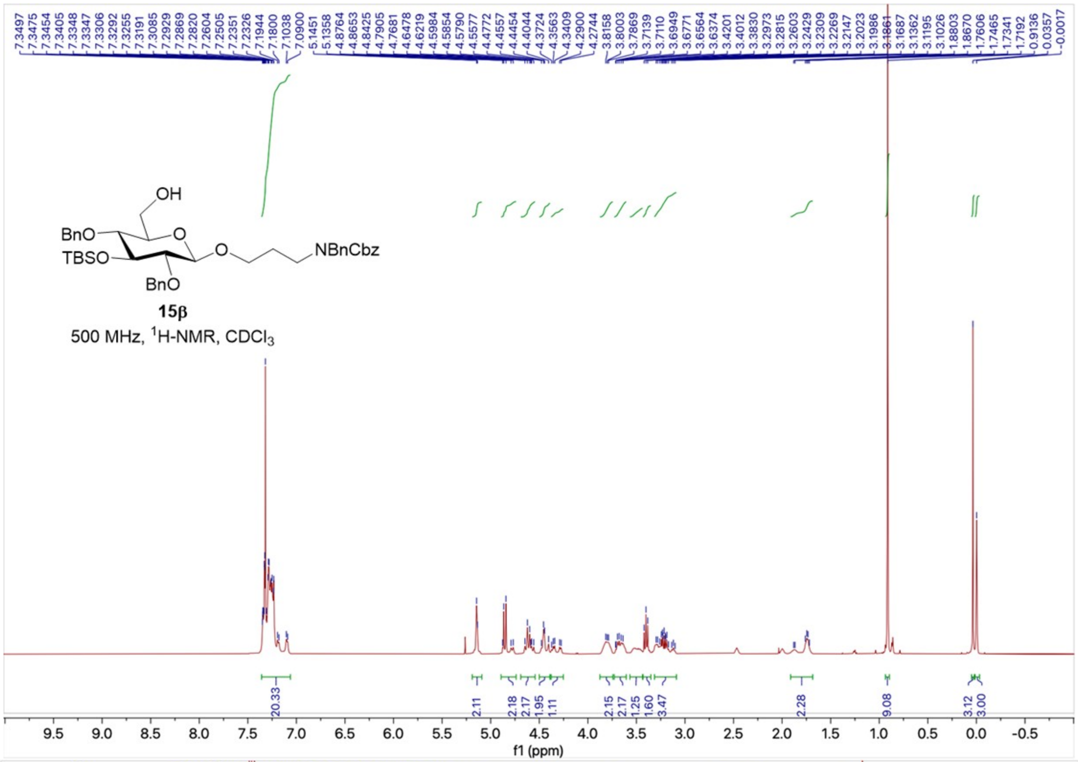
BnO
TBSO
BnO
OH
O
NBnCbz
15β
500 MHz, ¹H-NMR, CDCl₃
f1 (ppm)

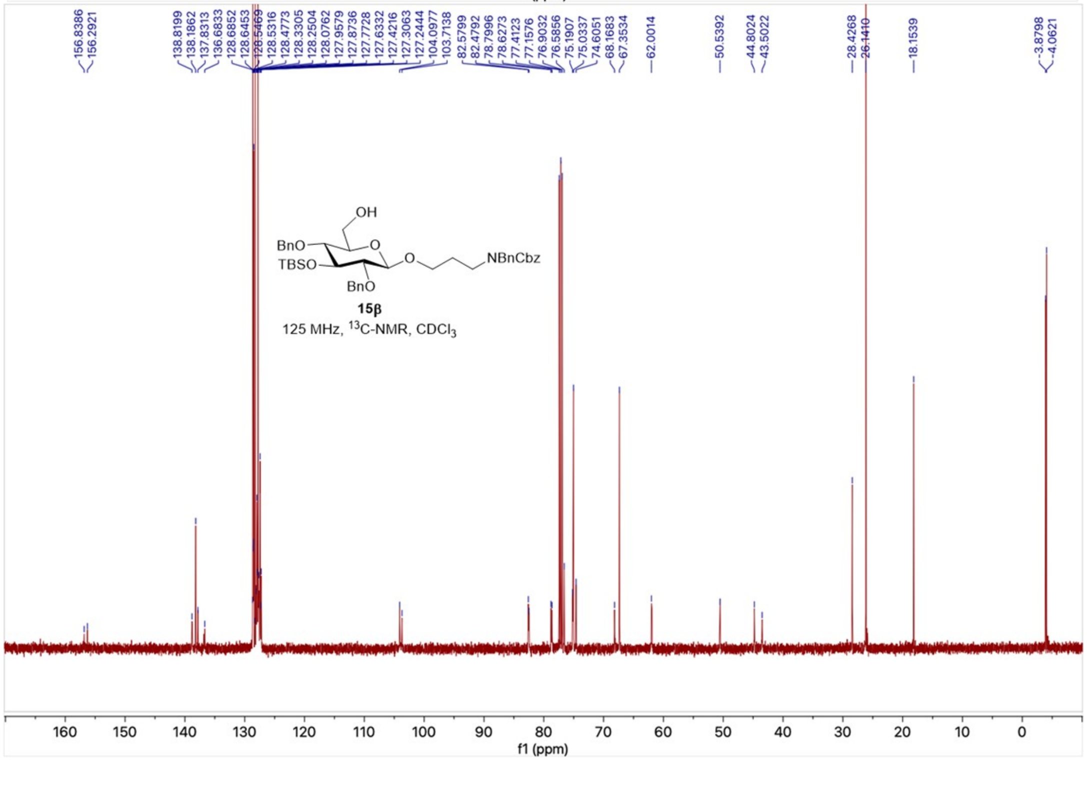
BnO
TBSO
BnO
OH
O
NBnCbz
15β
125 MHz, ¹³C-NMR, CDCl₃
f1 (ppm)

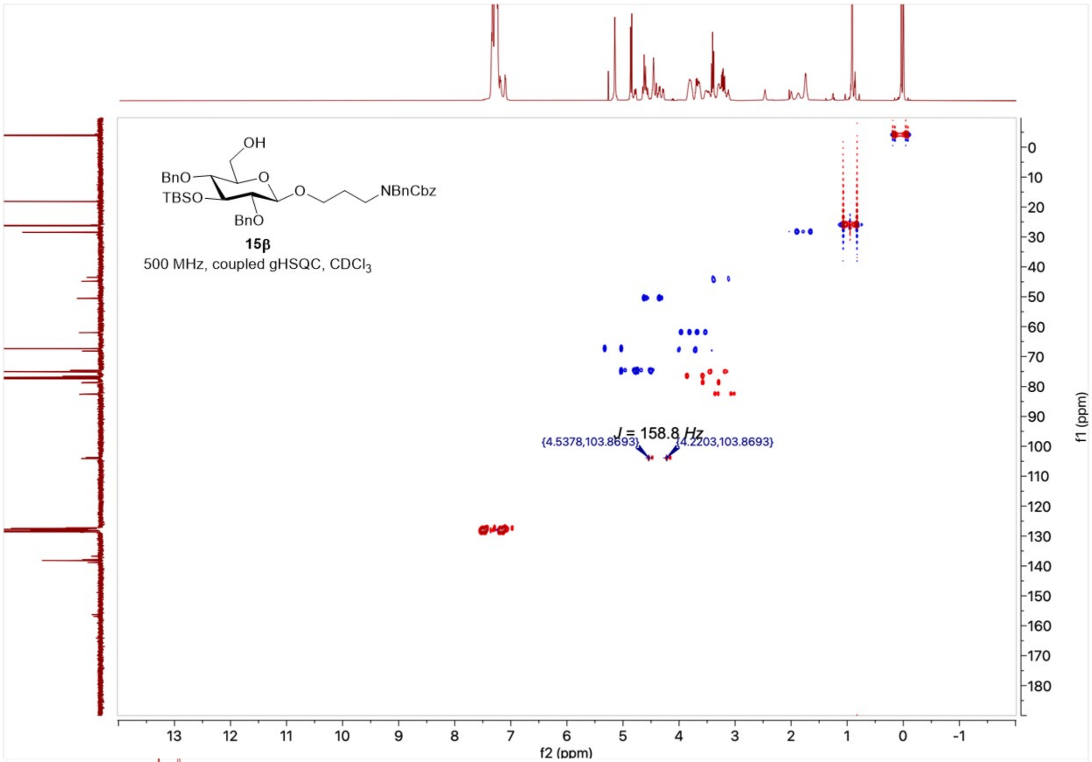
15β
500 MHz, coupled gHSQC, CDCl3
J = 158.8 Hz
{4.5378,103.8693}
{4.2203,103.8693}
f2 (ppm)
f1 (ppm)

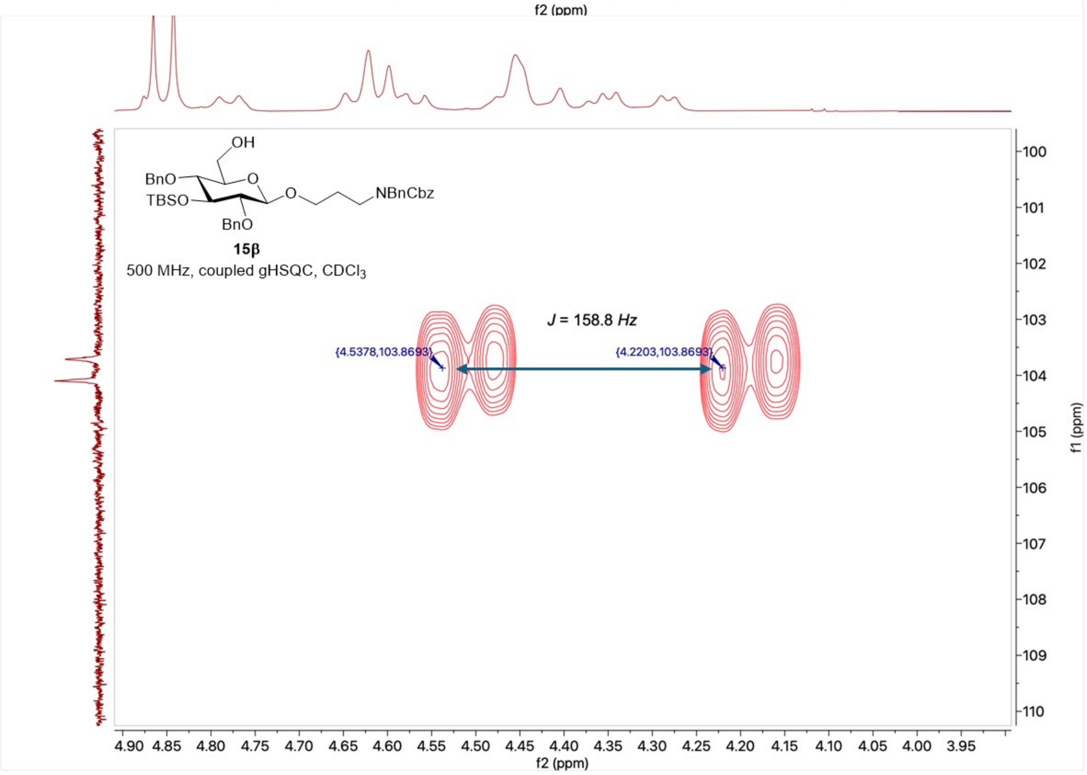
15β
500 MHz, coupled gHSQC, CDCl3
J = 158.8 Hz
{4.5378,103.8693}
{4.2203,103.8693}
f2 (ppm)
f1 (ppm)

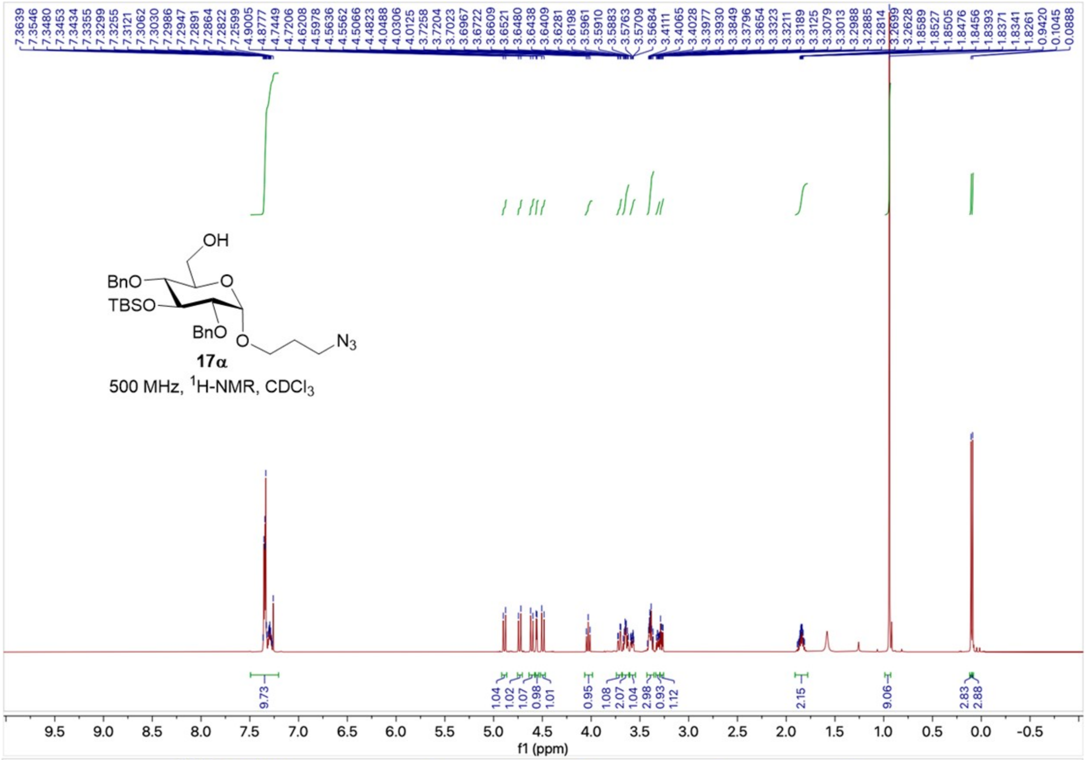
7.3639 7.3546 7.3480 7.3453 7.3434 7.3355 7.3299 7.3255 7.3121 7.3062 7.3030 7.2986 7.2947 7.2891 7.2864 7.2822 7.2599 4.9005 4.8777 4.7449 4.7206 4.6208 4.5978 4.5636 4.5562 4.5066 4.4823 4.0488 4.0306 4.0125 3.7258 3.7204 3.7023 3.6967 3.6722 3.6609 3.6521 3.6480 3.6438 3.6409 3.6281 3.6198 3.5961 3.5910 3.5883 3.5763 3.5709 3.5684 3.4111 3.4065 3.4028 3.3977 3.3930 3.3849 3.3796 3.3654 3.3323 3.3211 3.3189 3.3125 3.3079 3.3013 3.2988 3.2885 3.2814 3.2699 3.2628 1.8589 1.8527 1.8505 1.8476 1.8456 1.8393 1.8371 1.8341 1.8261 0.9420 0.1045 0.0888
OH
BnO
O
TBSO
BnO
O
N3
17α
500 MHz, 1H-NMR, CDCl3
9.73 1.04 1.02 1.07 0.98 1.01 0.95 1.08 2.07 1.04 2.98 0.93 1.12 2.15 9.06 2.83 2.88
9.5 9.0 8.5 8.0 7.5 7.0 6.5 6.0 5.5 5.0 4.5 4.0 3.5 3.0 2.5 2.0 1.5 1.0 0.5 0.0 -0.5
f1 (ppm)

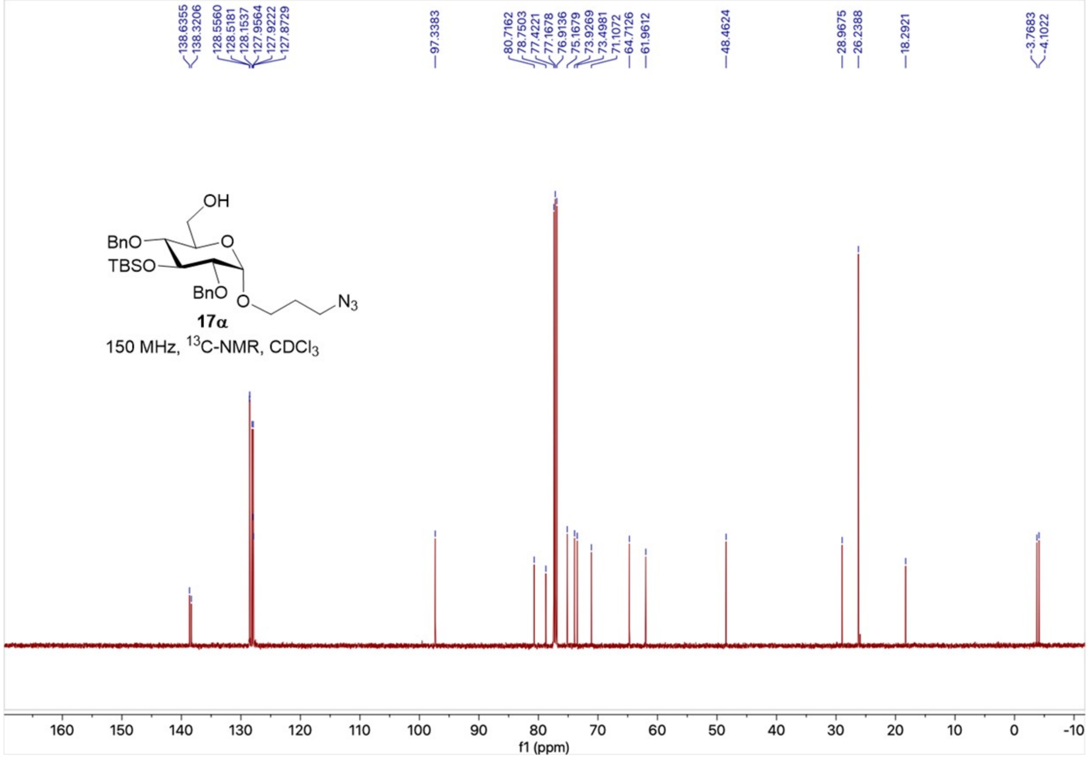
138.6355 138.3206 128.5560 128.5181 128.1537 127.9564 127.9222 127.8729 97.3383 80.7162 78.7503 77.4221 77.1678 76.9136 75.1679 73.9269 73.4981 71.1072 64.7126 61.9612 48.4624 28.9675 26.2388 18.2921 -3.7683 -4.1022
OH
BnO
O
TBSO
BnO
O
N3
17α
150 MHz, 13C-NMR, CDCl3
160 150 140 130 120 110 100 90 80 70 60 50 40 30 20 10 0 -10
f1 (ppm)

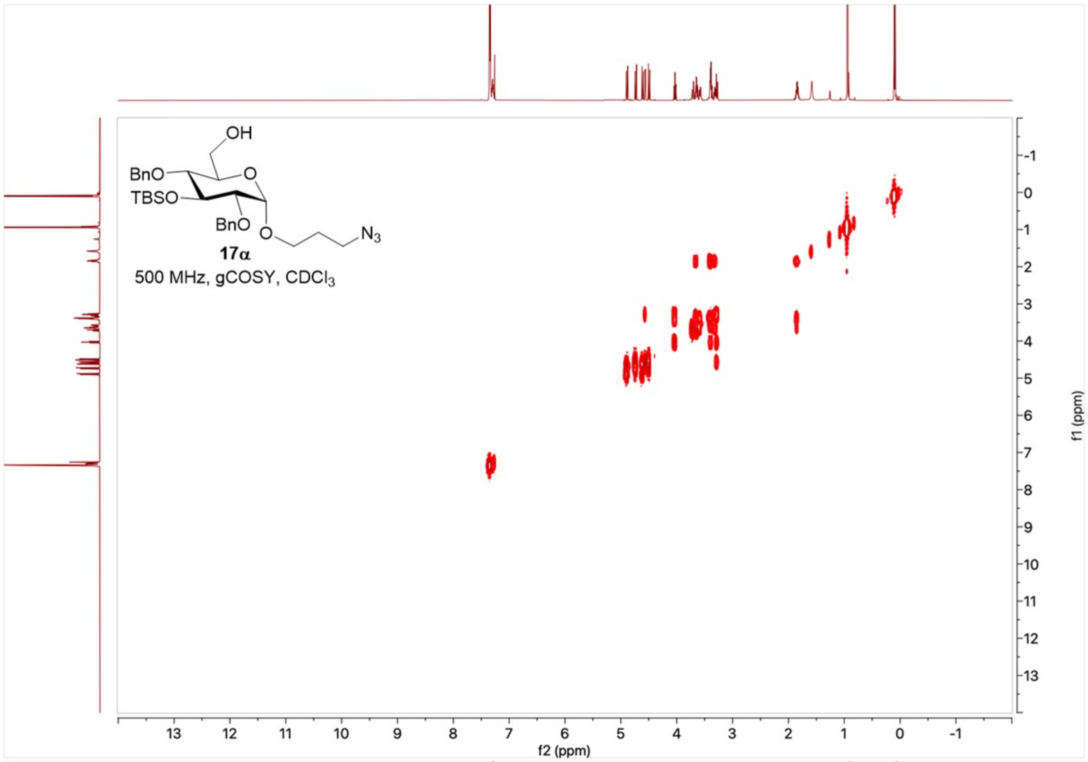
OH
BnO
O
TBSO
BnO
O
N3
17α
500 MHz, gCOSY, CDCl3
f1 (ppm)
f2 (ppm)

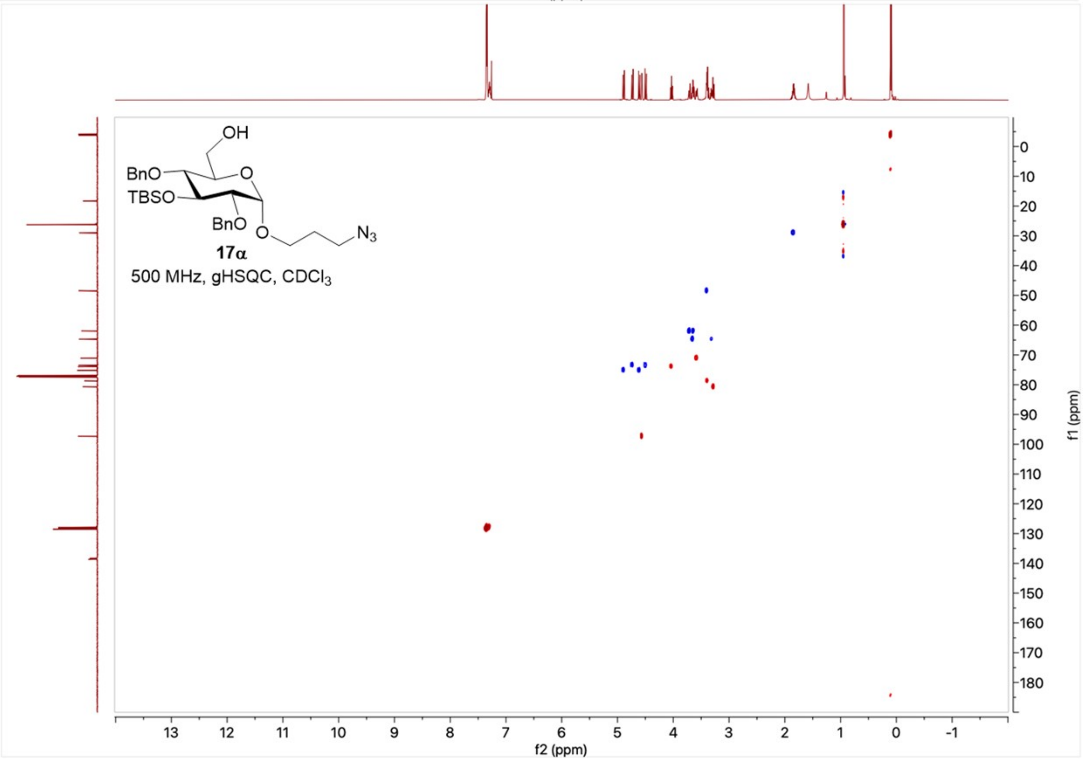
OH
BnO
O
TBSO
BnO
O
N3
17α
500 MHz, gHSQC, CDCl3
f1 (ppm)
f2 (ppm)

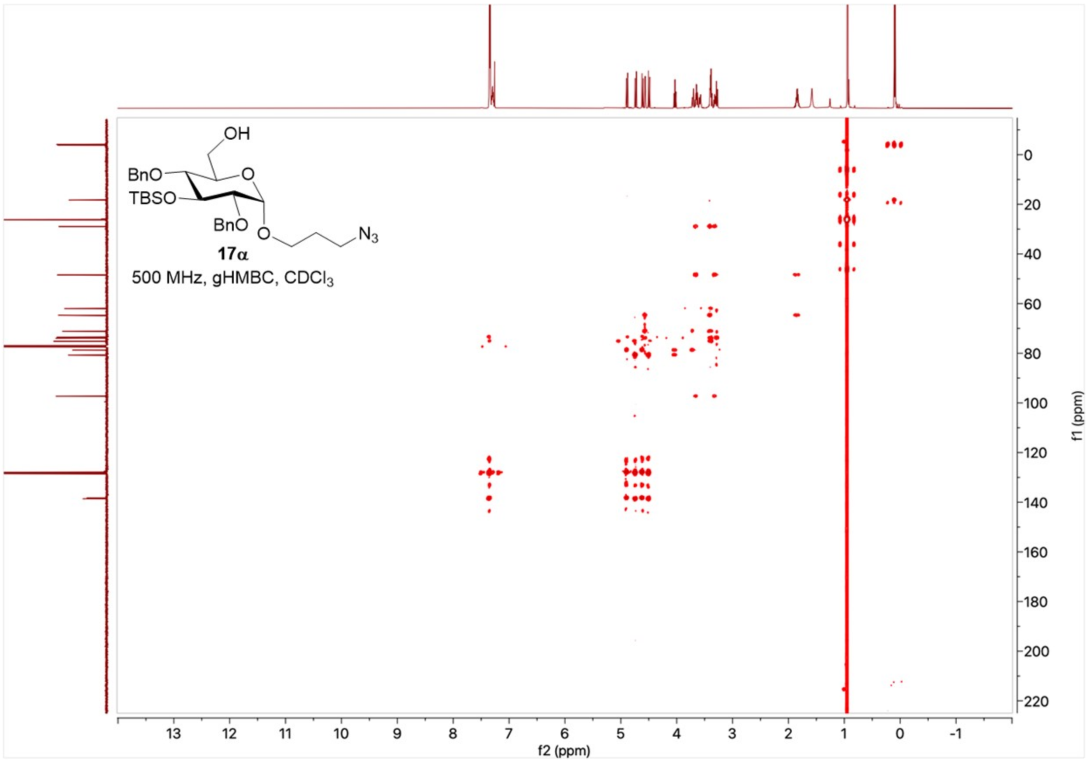

OH
BnO
O
TBSO
BnO
O
N3
17α
500 MHz, gHMBC, CDCl3
f1 (ppm)
f2 (ppm)

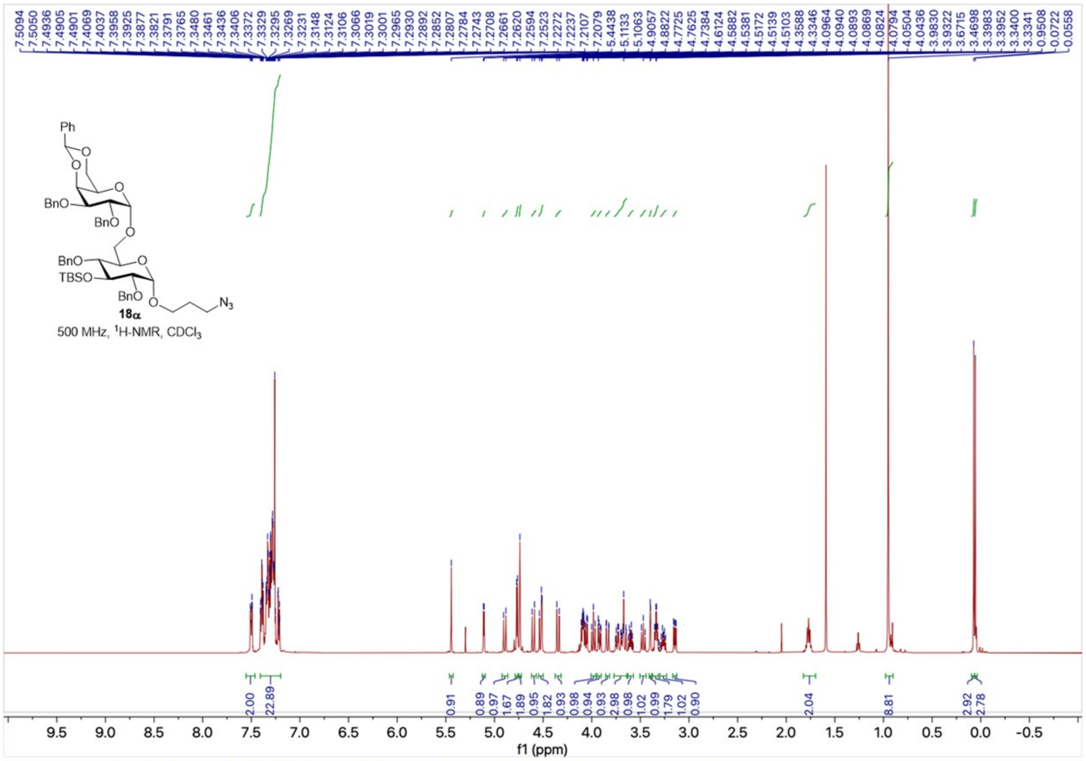

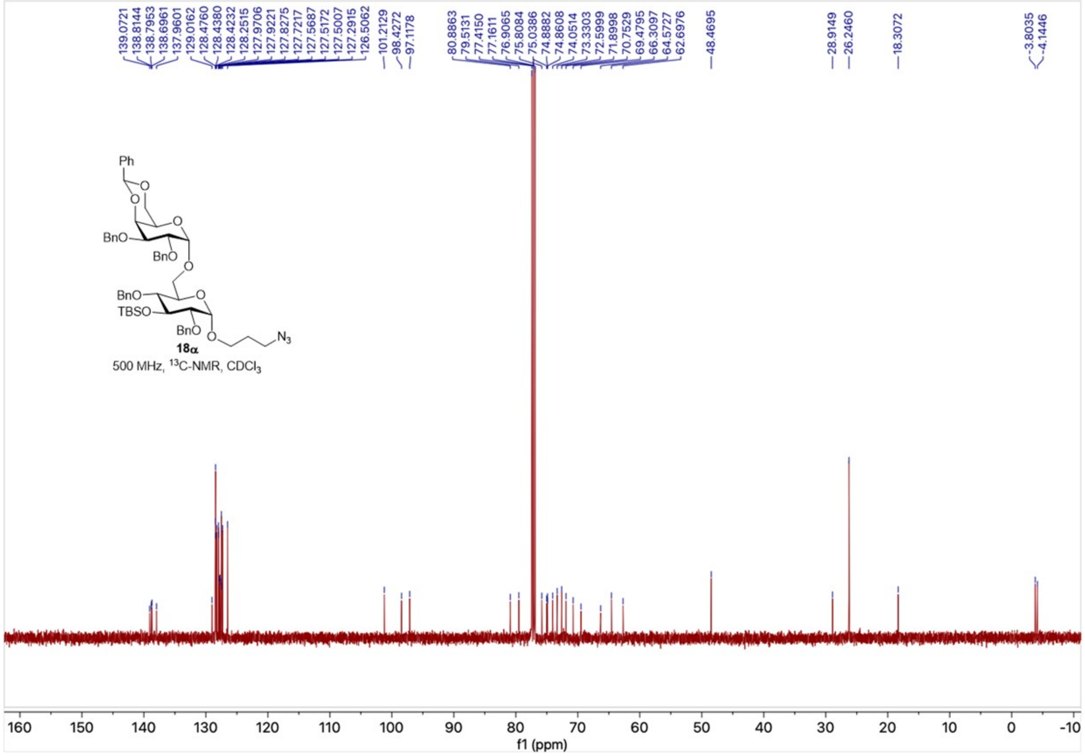

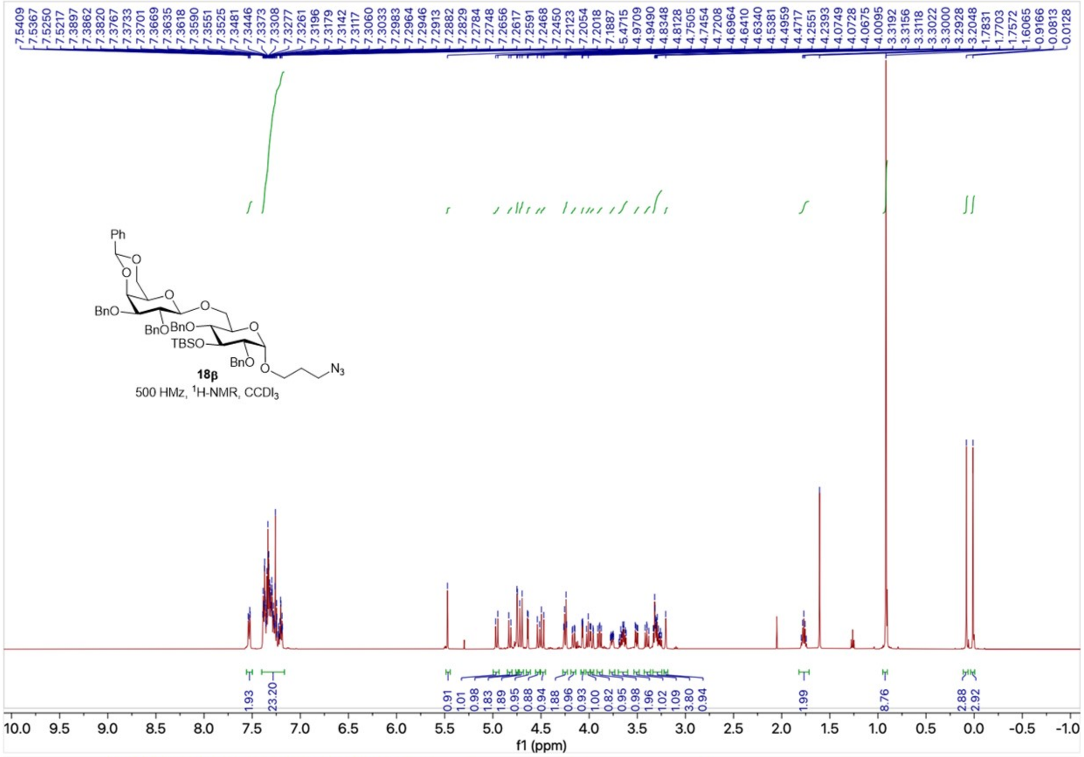
18β
500 HMz, 1H-NMR, CCDl3
f1 (ppm)

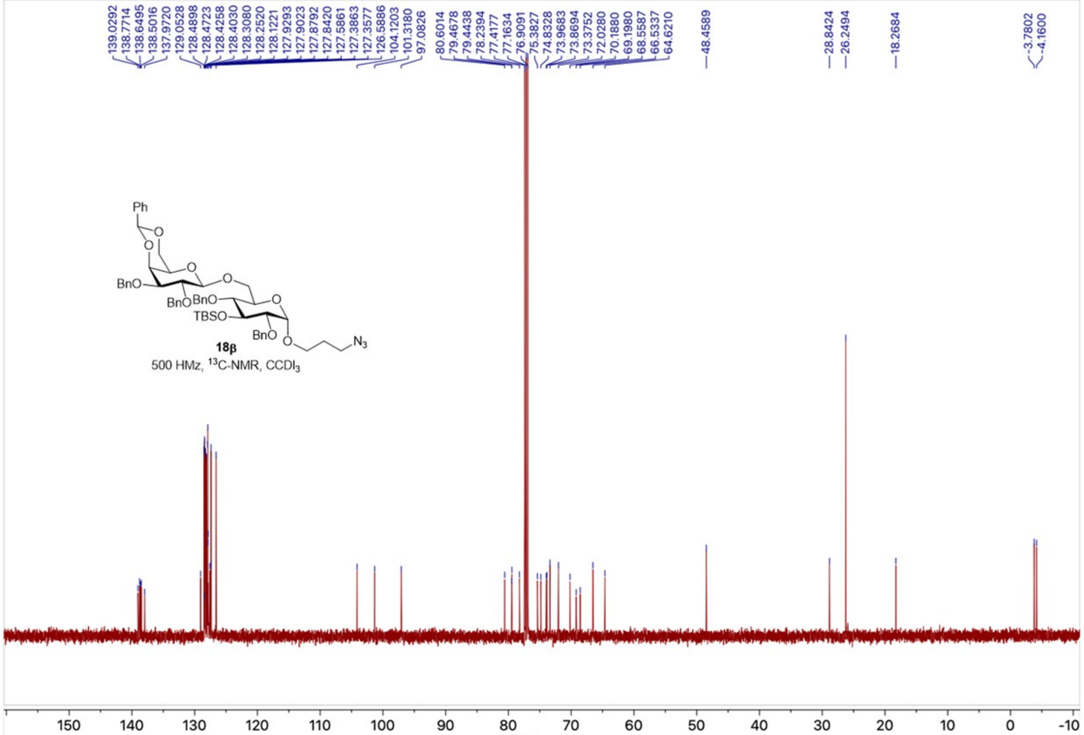
18β
500 HMz, 13C-NMR, CCDl3
f1 (ppm)

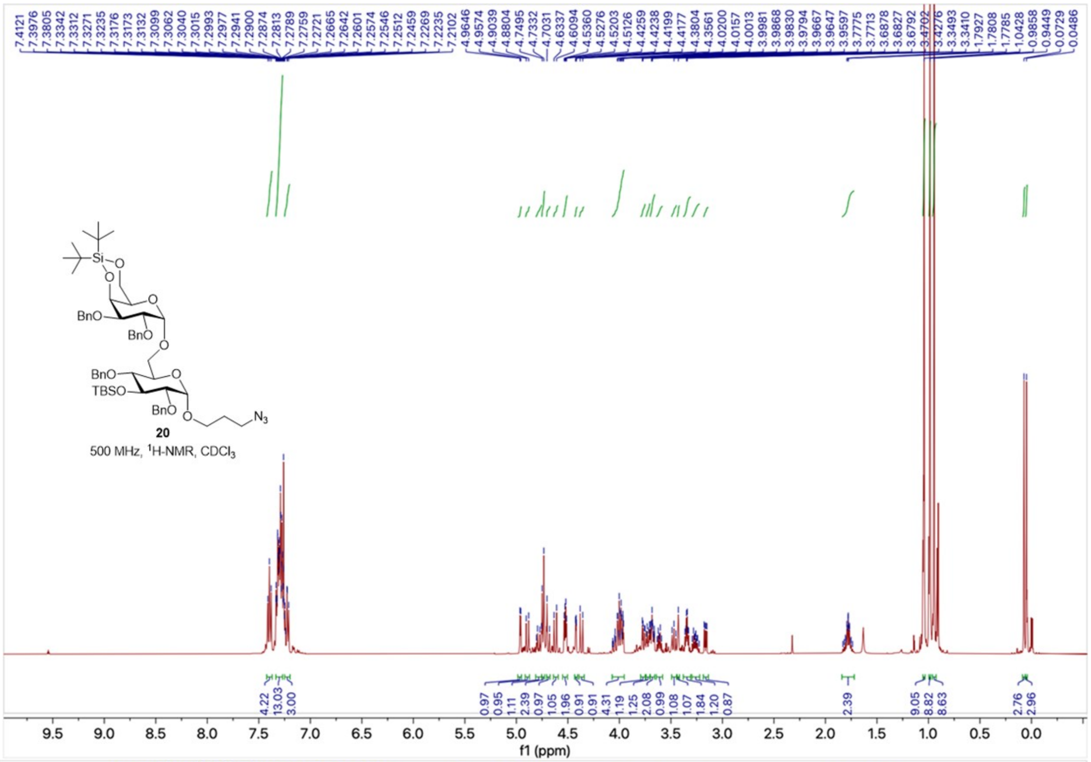

20
500 MHz, ^{1}H-NMR, $CDCl_3$

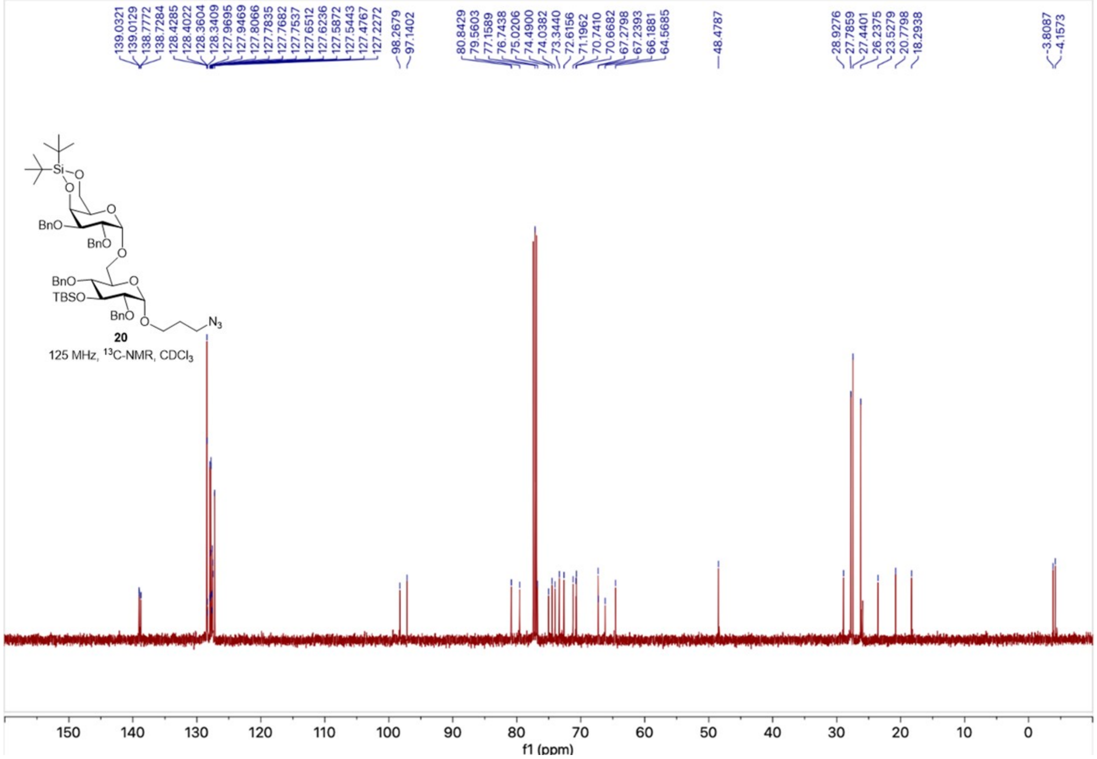

20
125 MHz, ^{13}C-NMR, $CDCl_3$

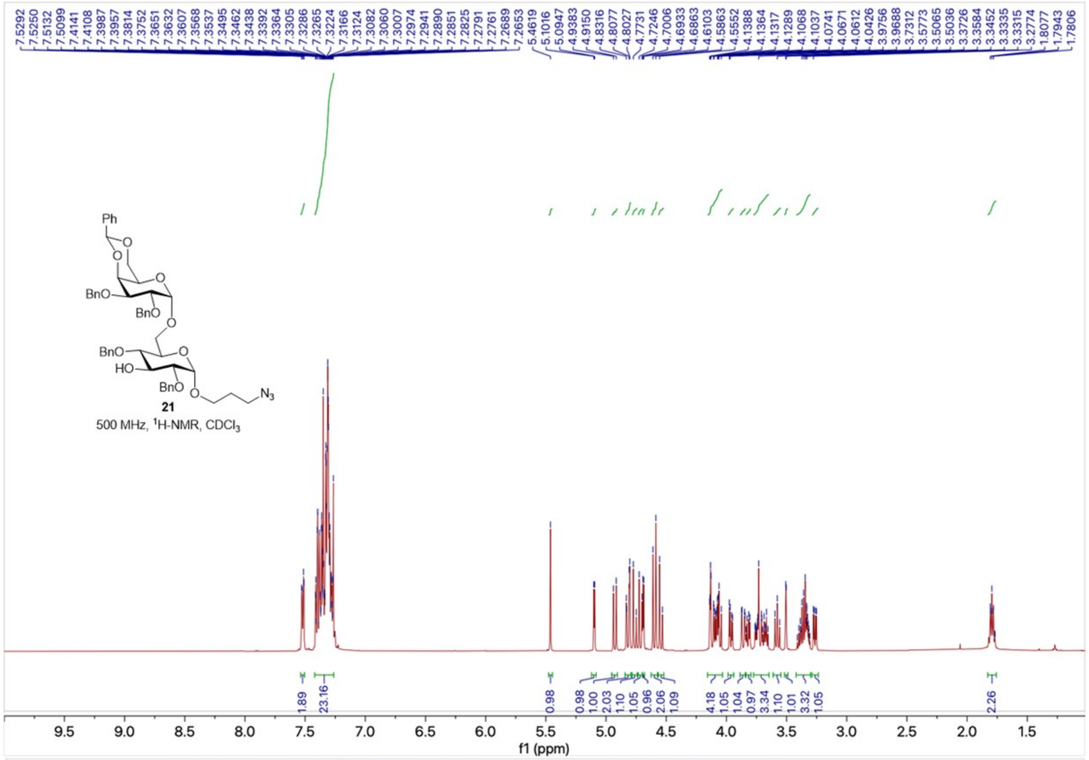

21
500 MHz, ^{1}H-NMR, CDCl$_3$
f1 (ppm)

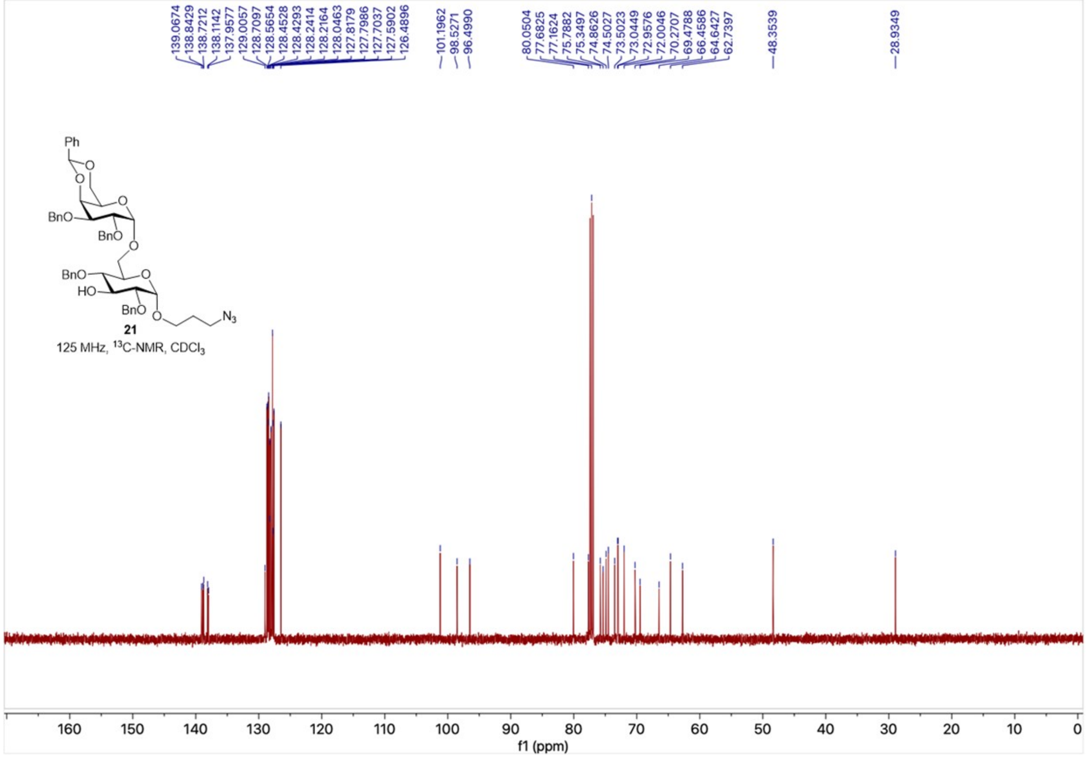

21
125 MHz, ^{13}C-NMR, CDCl$_3$
f1 (ppm)

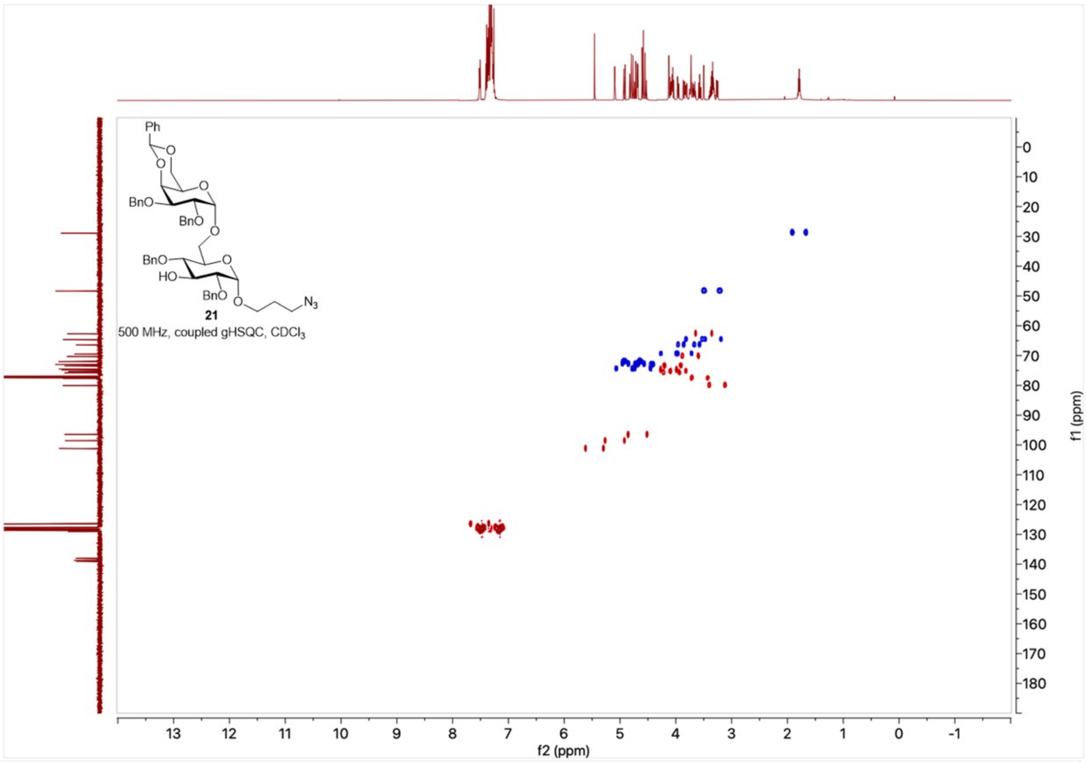
Ph
BnO
BnO
BnO
HO
BnO
N3
21
500 MHz, coupled gHSQC, CDCl3
f1 (ppm)
f2 (ppm)

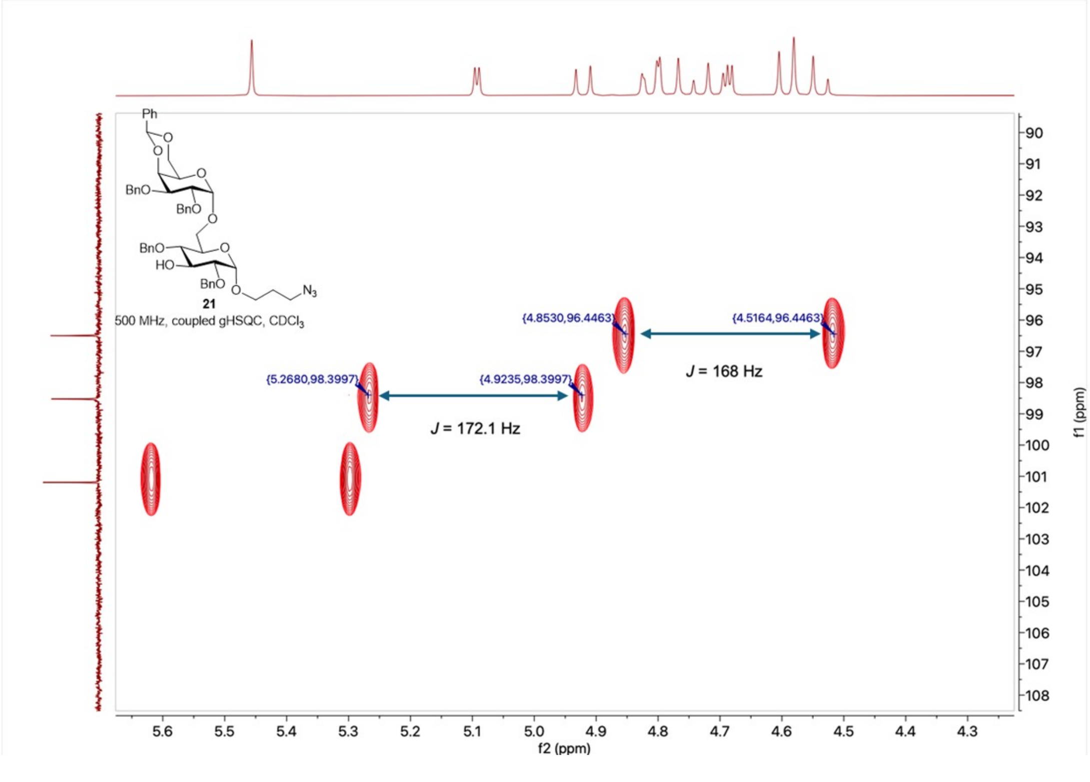
Ph
BnO
BnO
BnO
HO
BnO
N3
21
500 MHz, coupled gHSQC, CDCl3
{5.2680,98.3997}
{4.9235,98.3997}
{4.8530,96.4463}
{4.5164,96.4463}
J = 172.1 Hz
J = 168 Hz
f1 (ppm)
f2 (ppm)

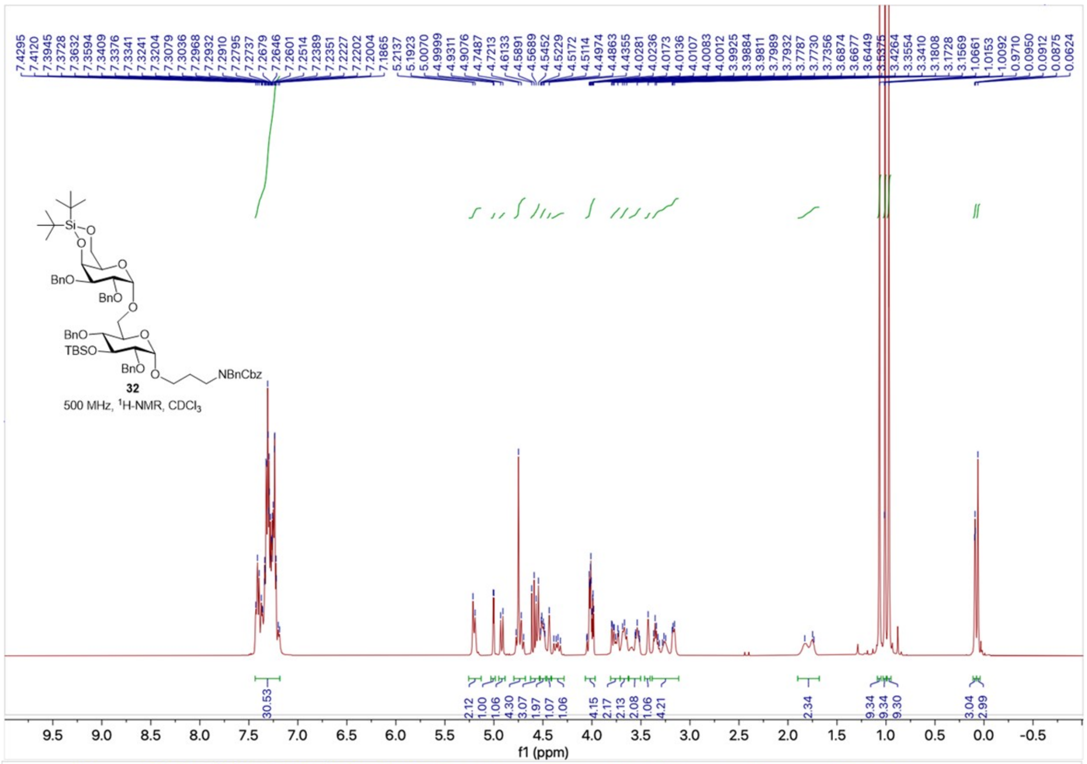
BnO
BnO
BnO
TBSO
BnO
NBnCbz
32
500 MHz, ¹H-NMR, CDCl₃
f1 (ppm)

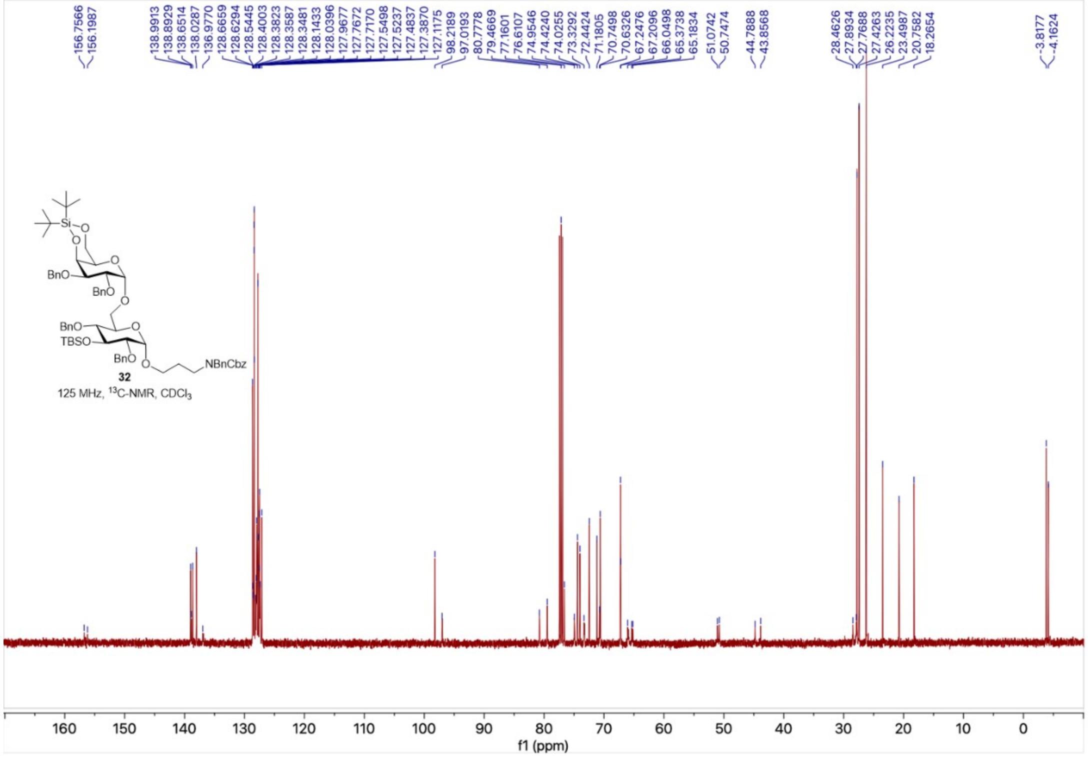
BnO
BnO
BnO
TBSO
BnO
NBnCbz
32
125 MHz, ¹³C-NMR, CDCl₃
f1 (ppm)

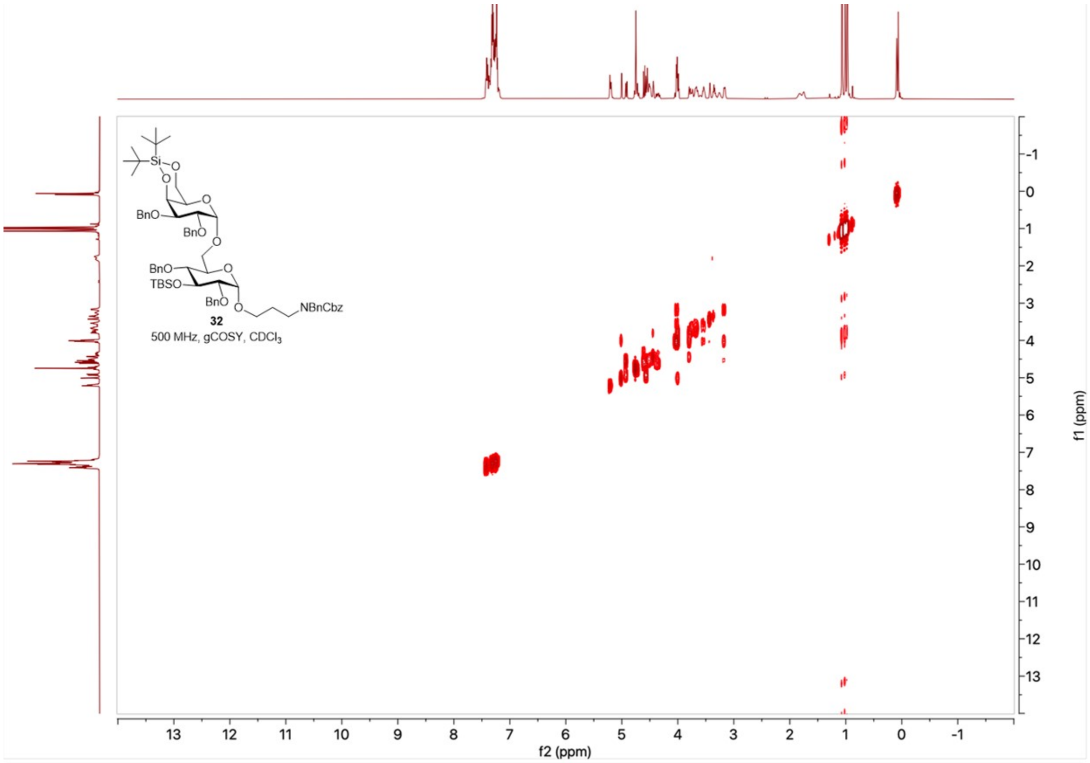
32
500 MHz, gCOSY, CDCl3
f1 (ppm)
f2 (ppm)

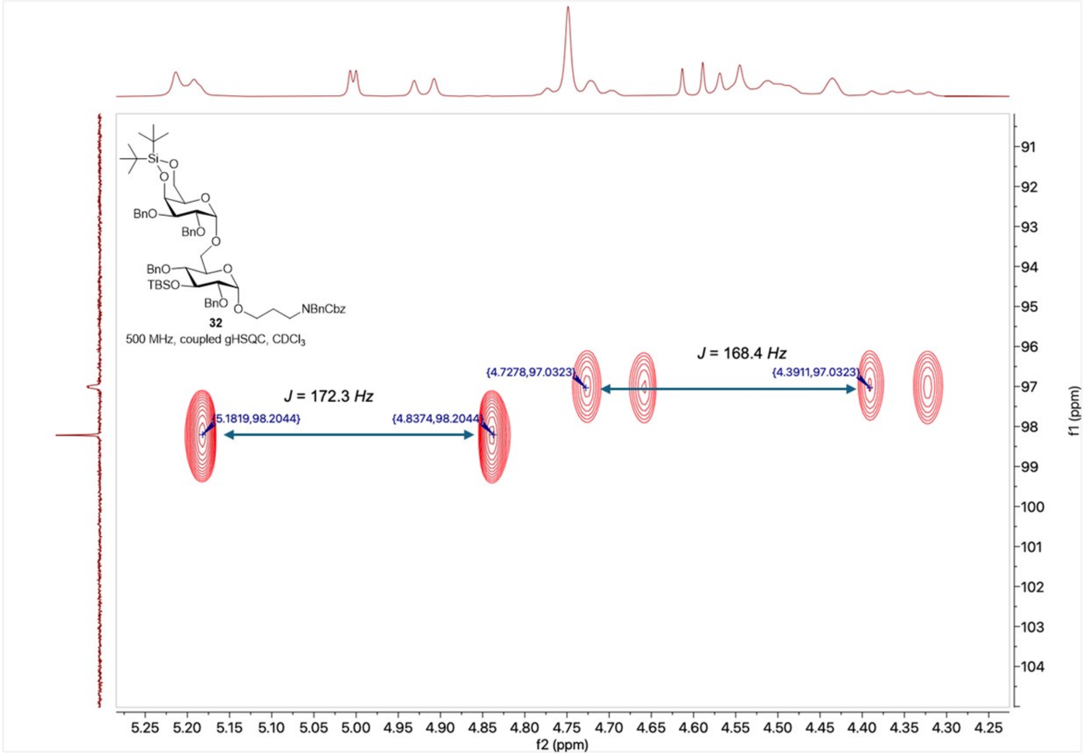
32
500 MHz, coupled gHSQC, CDCl3
J = 172.3 Hz
J = 168.4 Hz
{5.1819,98.2044}
{4.8374,98.2044}
{4.7278,97.0323}
{4.3911,97.0323}
f1 (ppm)
f2 (ppm)

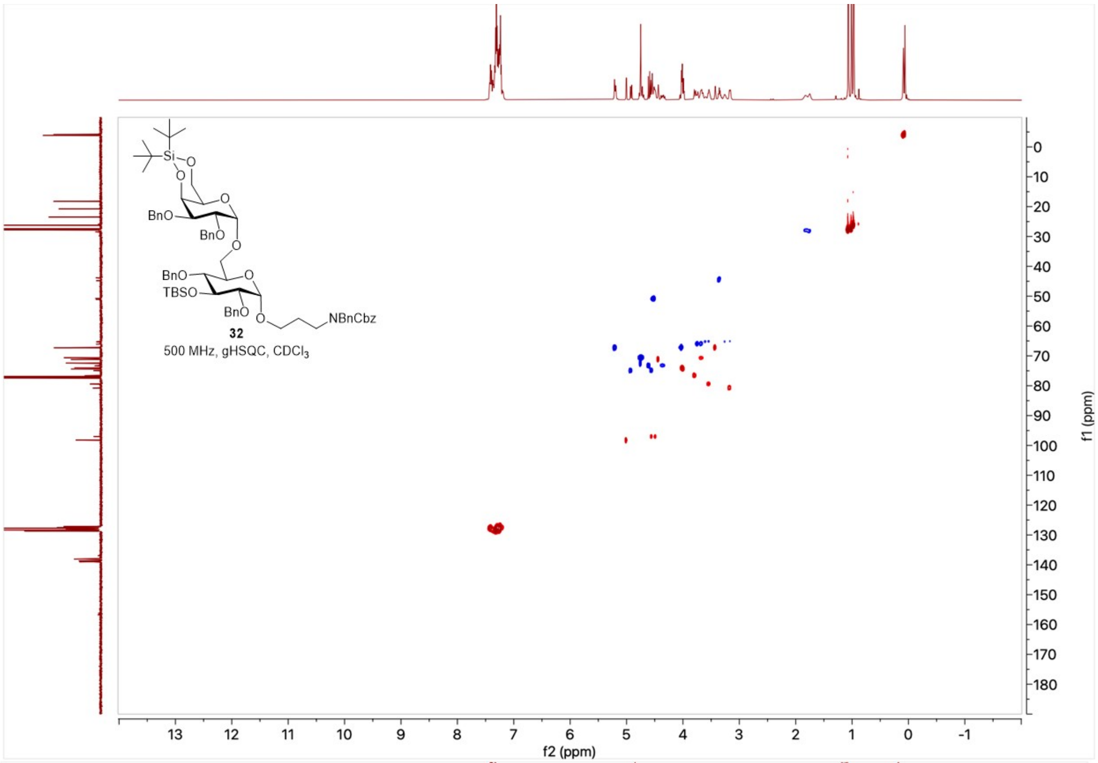
BnO
BnO
BnO
TBSO
BnO
NBnCbz
32
500 MHz, gHSQC, CDCl3
f1 (ppm)
f2 (ppm)

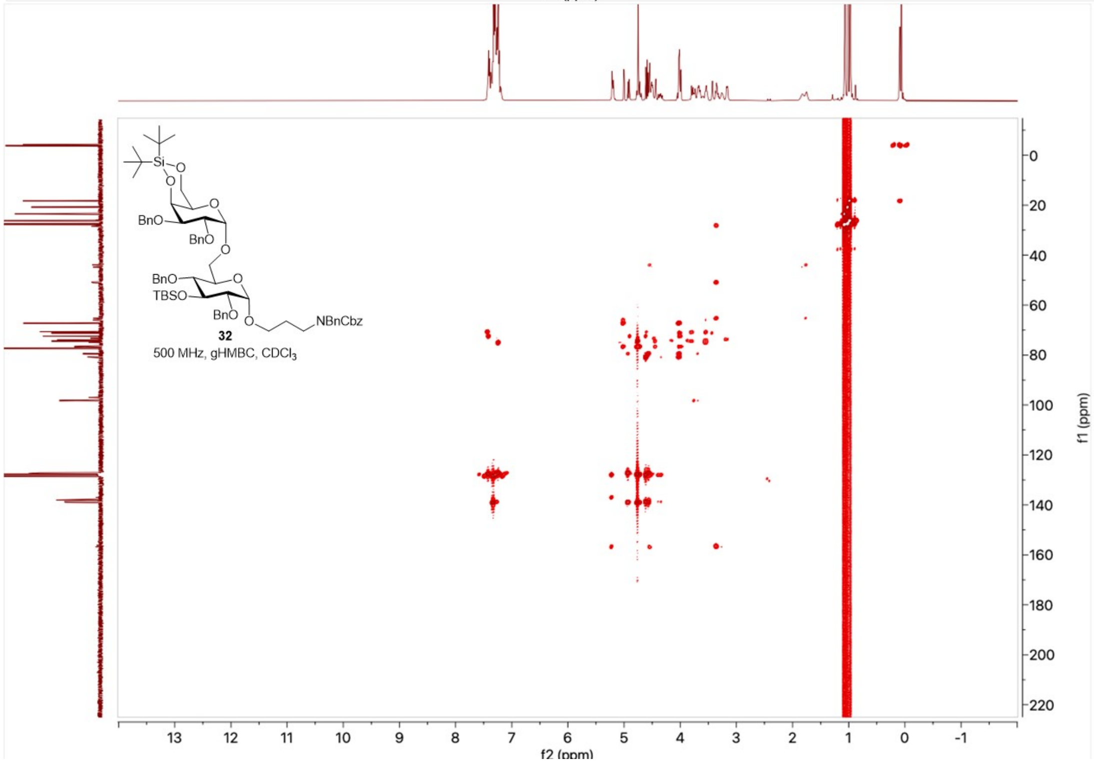
BnO
BnO
BnO
TBSO
BnO
NBnCbz
32
500 MHz, gHMBC, CDCl3
f1 (ppm)
f2 (ppm)

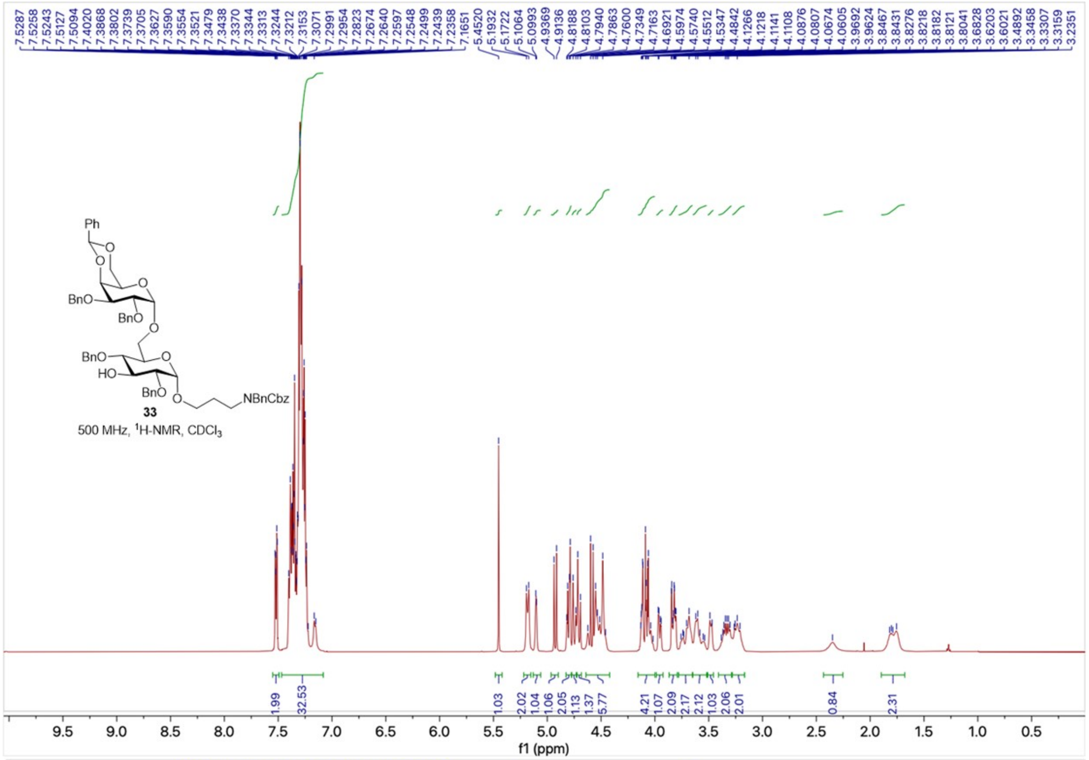

33
500 MHz, ^{1}H-NMR, $CDCl_3$
f1 (ppm)

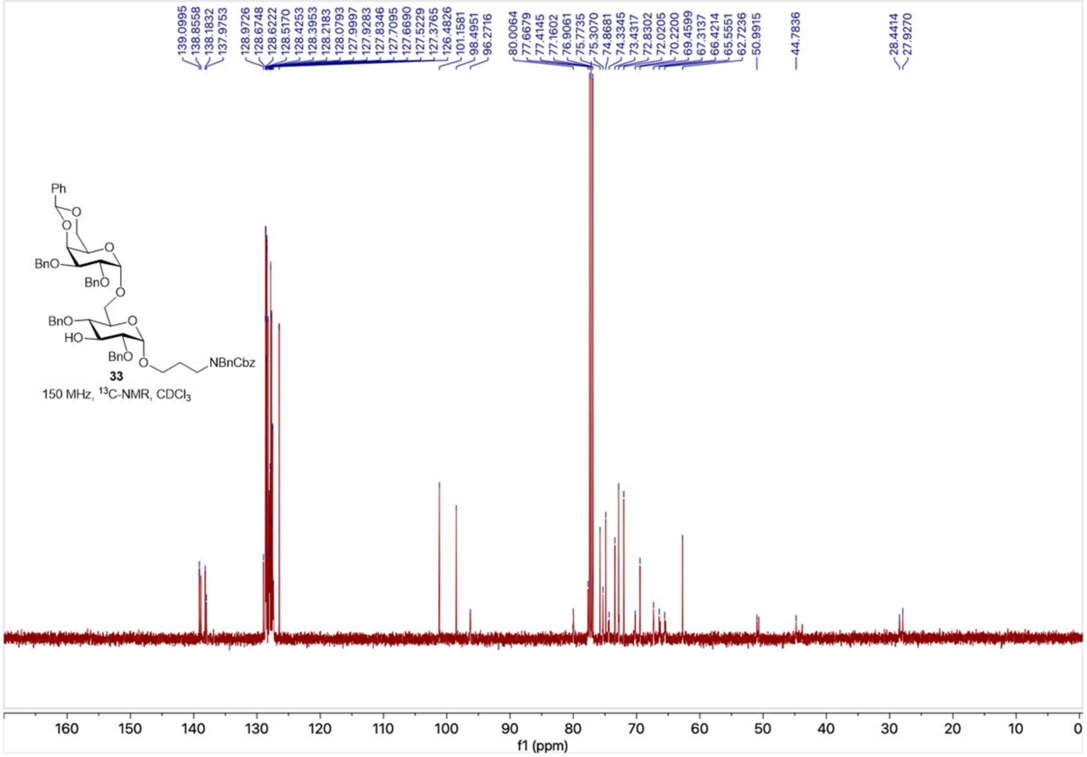

33
150 MHz, ^{13}C-NMR, $CDCl_3$
f1 (ppm)

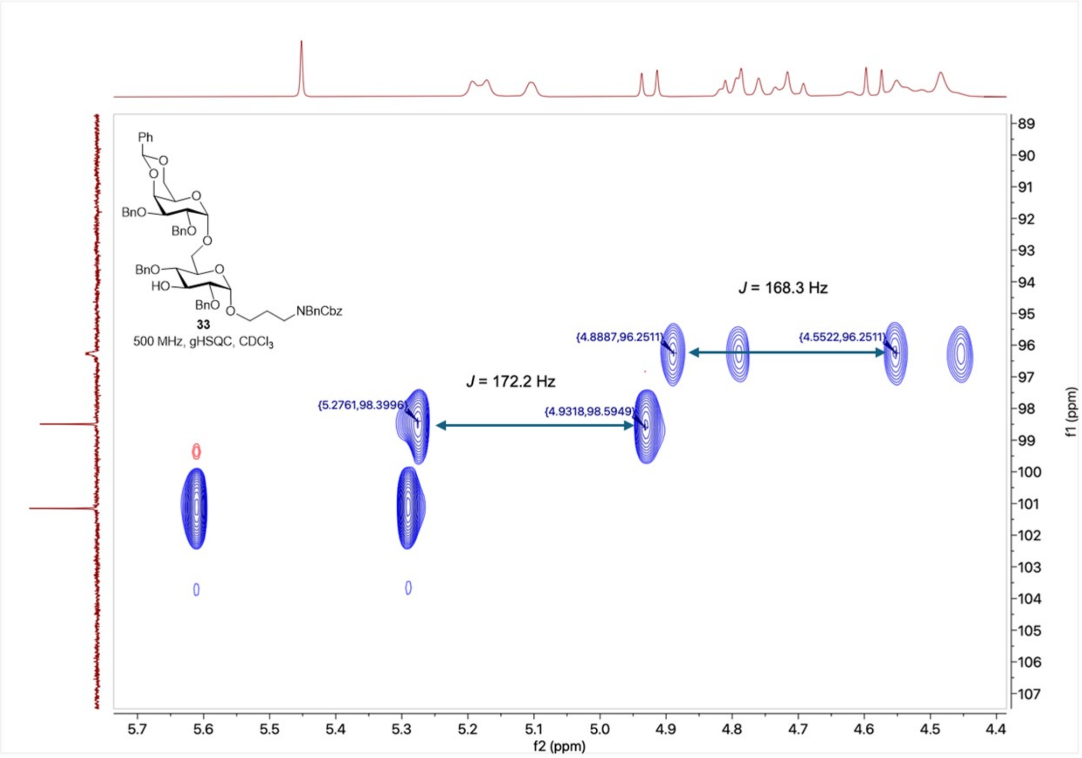
Ph
BnO
BnO
BnO
HO
BnO
NBnCbz
33
500 MHz, gHSQC, CDCl3
J = 168.3 Hz
J = 172.2 Hz
{4.8887,96.2511}
{4.5522,96.2511}
{5.2761,98.3996}
{4.9318,98.5949}
f2 (ppm)
f1 (ppm)

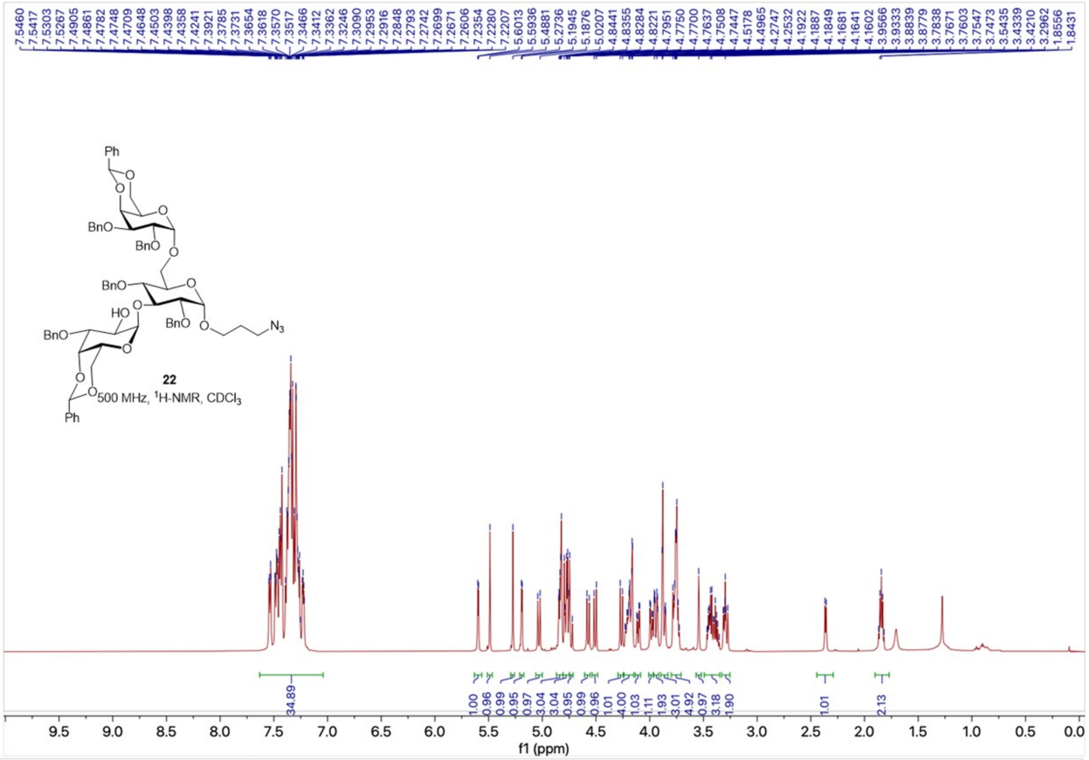
7.5460
7.5417
7.5303
7.5267
7.4905
7.4861
7.4782
7.4748
7.4709
7.4648
7.4503
7.4398
7.4358
7.4241
7.3921
7.3785
7.3731
7.3654
7.3618
7.3570
7.3517
7.3466
7.3412
7.3362
7.3246
7.3090
7.2953
7.2916
7.2848
7.2793
7.2742
7.2699
7.2671
7.2606
7.2354
7.2280
7.2207
5.6013
5.5936
5.4881
5.2736
5.1945
5.1876
5.0207
4.8441
4.8355
4.8284
4.8221
4.7951
4.7750
4.7637
4.7508
4.7447
4.5178
4.4965
4.2747
4.2532
4.1922
4.1887
4.1849
4.1681
4.1641
4.1602
3.9566
3.9333
3.8839
3.8779
3.7838
3.7671
3.7603
3.7547
3.7473
3.5435
3.4339
3.4210
3.2962
1.8556
1.8431
Ph
BnO
BnO
BnO
HO
BnO
BnO
N3
22
500 MHz, 1H-NMR, CDCl3
Ph
34.89
1.00
0.96
0.99
0.95
0.97
3.04
3.04
0.95
0.99
0.96
1.01
4.00
1.03
1.11
1.93
3.01
4.92
0.97
3.18
1.90
1.01
2.13
9.5
9.0
8.5
8.0
7.5
7.0
6.5
6.0
5.5
5.0
4.5
4.0
3.5
3.0
2.5
2.0
1.5
1.0
0.5
0.0
f1 (ppm)

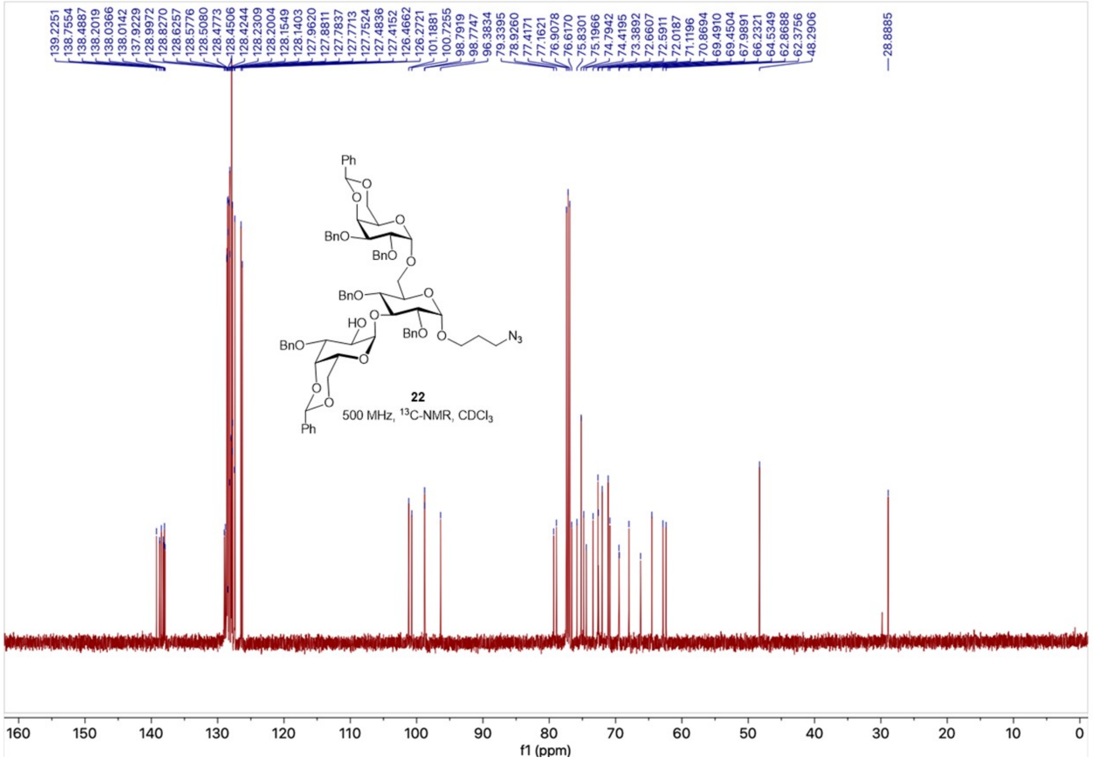
139.2251
138.7554
138.4887
138.2019
138.0366
138.0142
137.9229
128.9972
128.8270
128.6257
128.5776
128.5080
128.4773
128.4506
128.4244
128.2309
128.2004
128.1549
128.1403
127.9620
127.8811
127.7837
127.7713
127.7524
127.4836
127.4152
126.4662
126.2721
101.1881
100.7255
98.7919
98.7747
96.3834
79.3395
78.9260
77.4171
77.1621
76.9078
76.6170
75.8301
75.1966
74.7942
74.4195
73.3892
72.6607
72.5911
72.0187
71.1196
70.8694
69.4910
69.4504
67.9891
66.2321
64.5349
62.8688
62.3756
48.2906
28.8885
Ph
BnO
BnO
BnO
HO
BnO
BnO
N3
22
500 MHz, 13C-NMR, CDCl3
Ph
160
150
140
130
120
110
100
90
80
70
60
50
40
30
20
10
0
f1 (ppm)

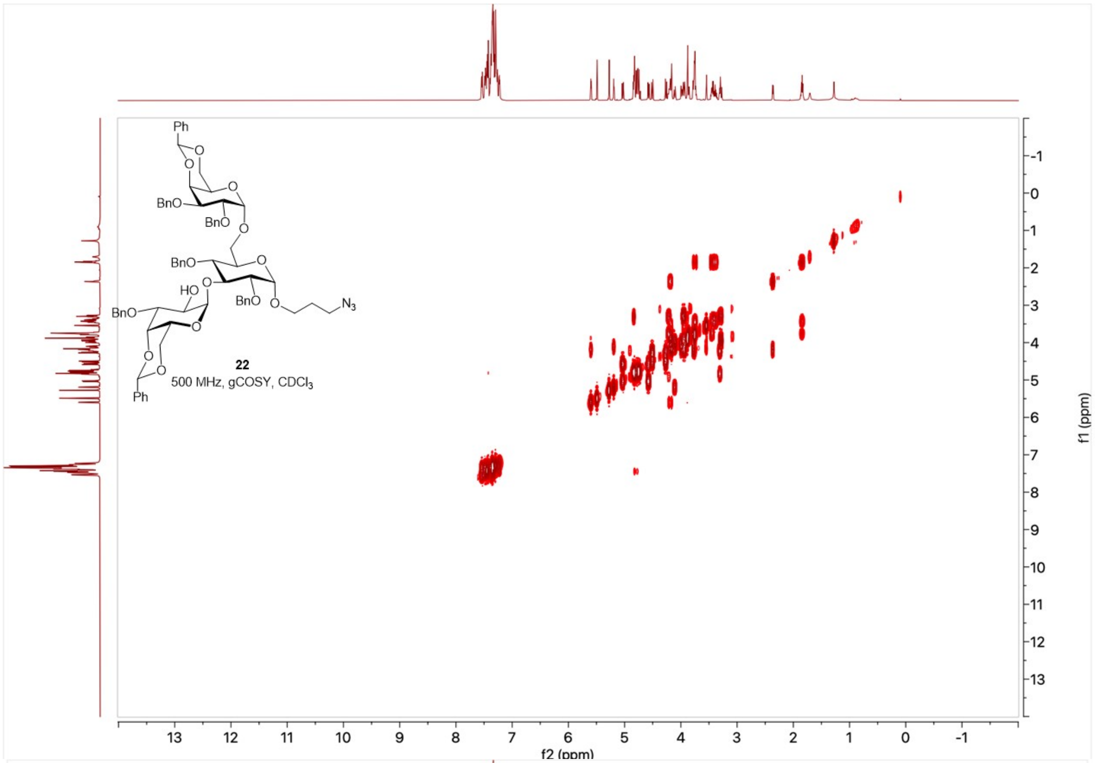

22
500 MHz, gCOSY, CDCl3
f1 (ppm)
f2 (ppm)

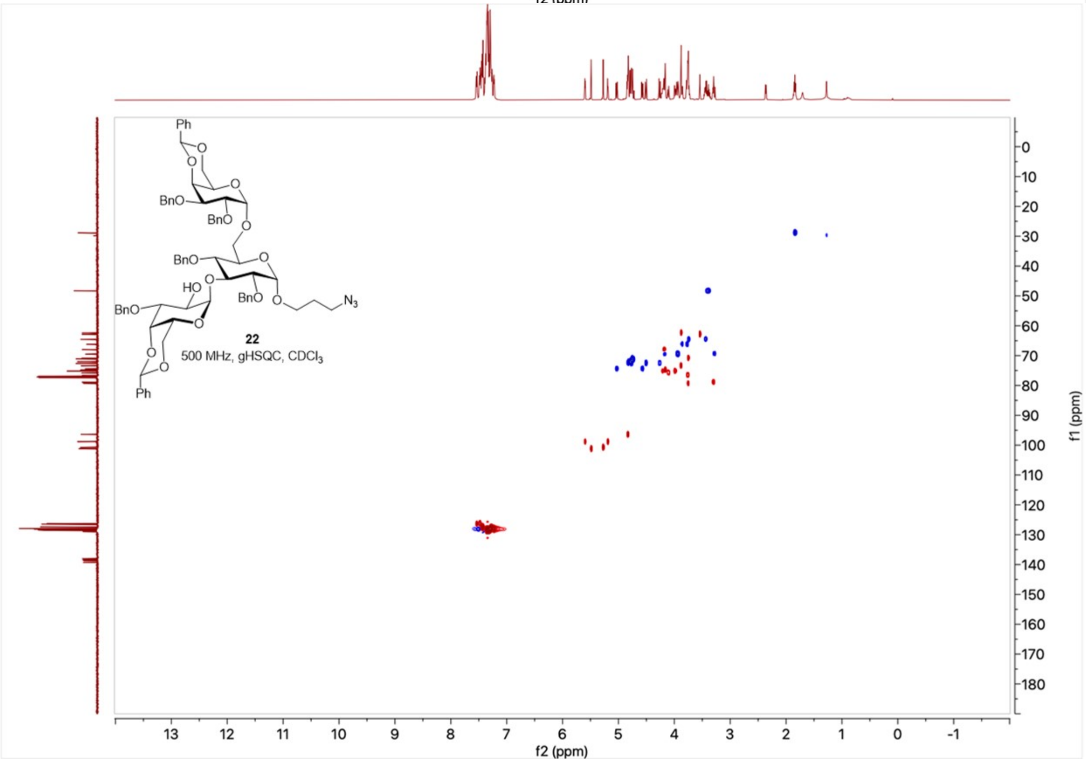

22
500 MHz, gHSQC, CDCl3
f1 (ppm)
f2 (ppm)

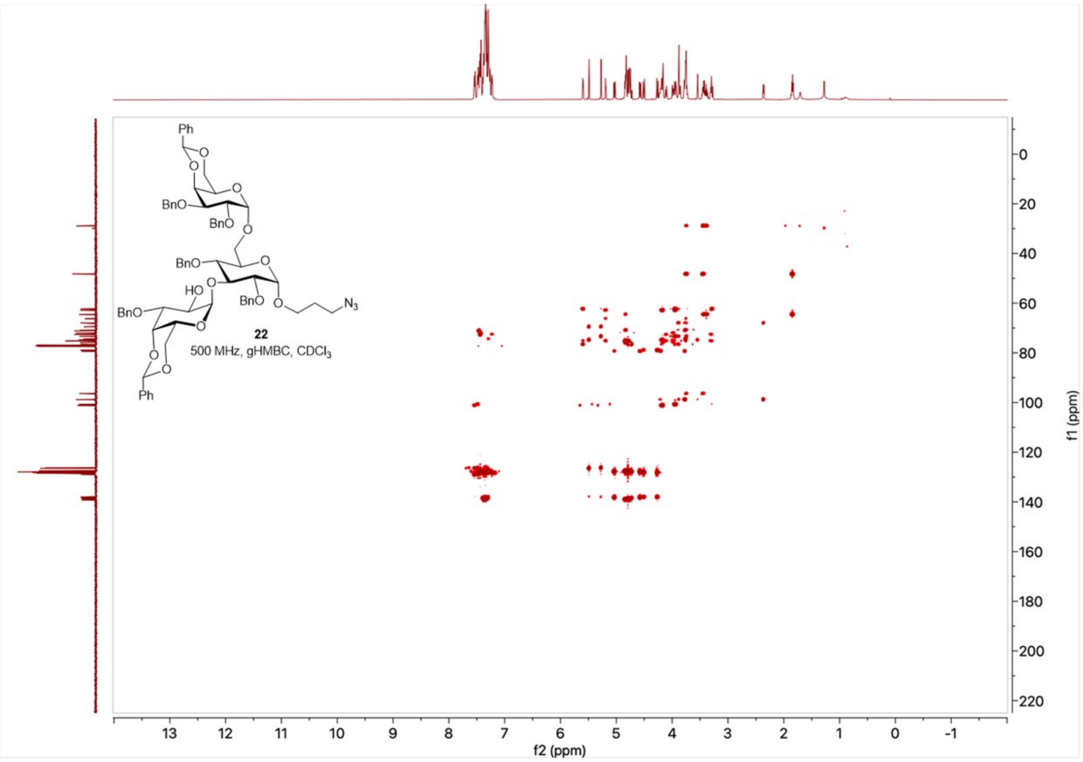
22
500 MHz, gHMBC, CDCl3
f2 (ppm)
f1 (ppm)

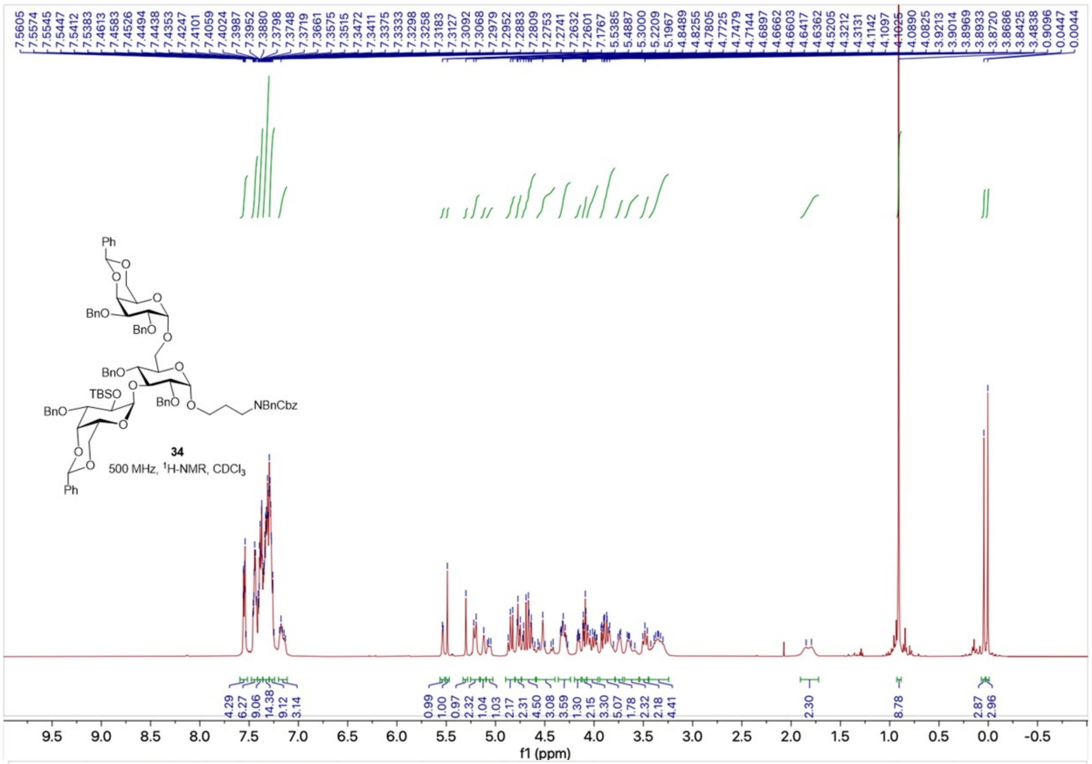
34
500 MHz, 1H-NMR, CDCl3
f1 (ppm)

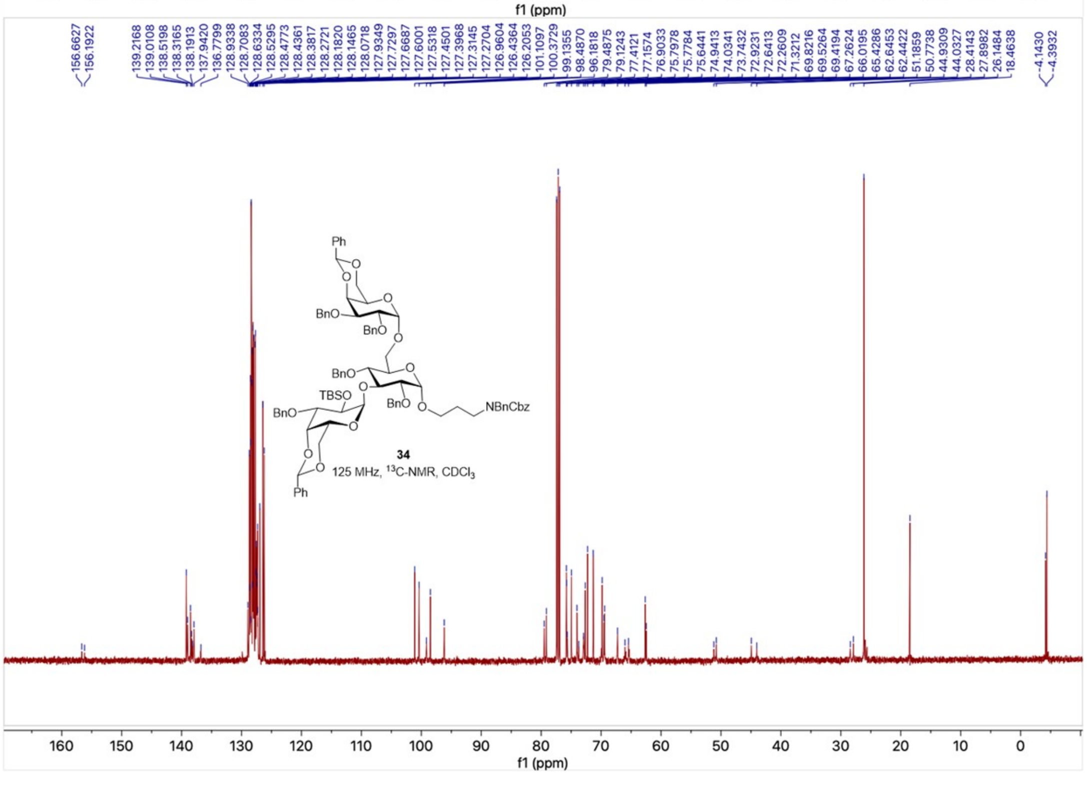
34
125 MHz, 13C-NMR, CDCl3
f1 (ppm)

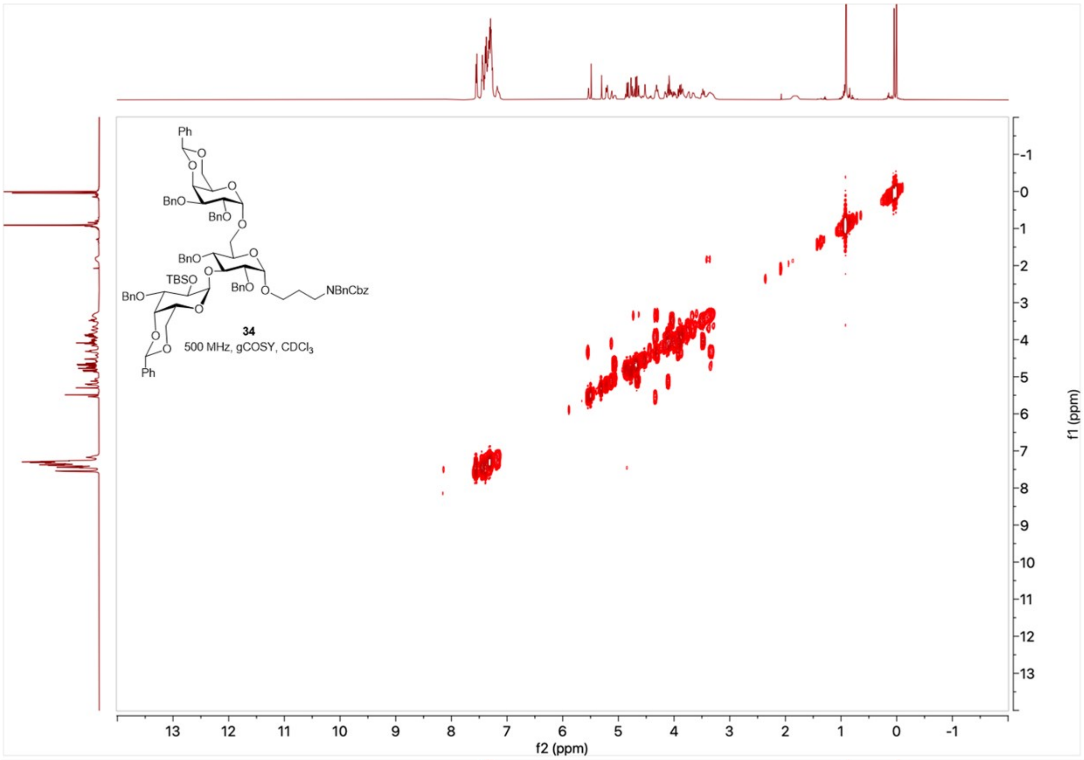
Ph
BnO
BnO
BnO
TBSO
BnO
NBnCbz
BnO
Ph
34
500 MHz, gCOSY, CDCl3
f1 (ppm)
f2 (ppm)

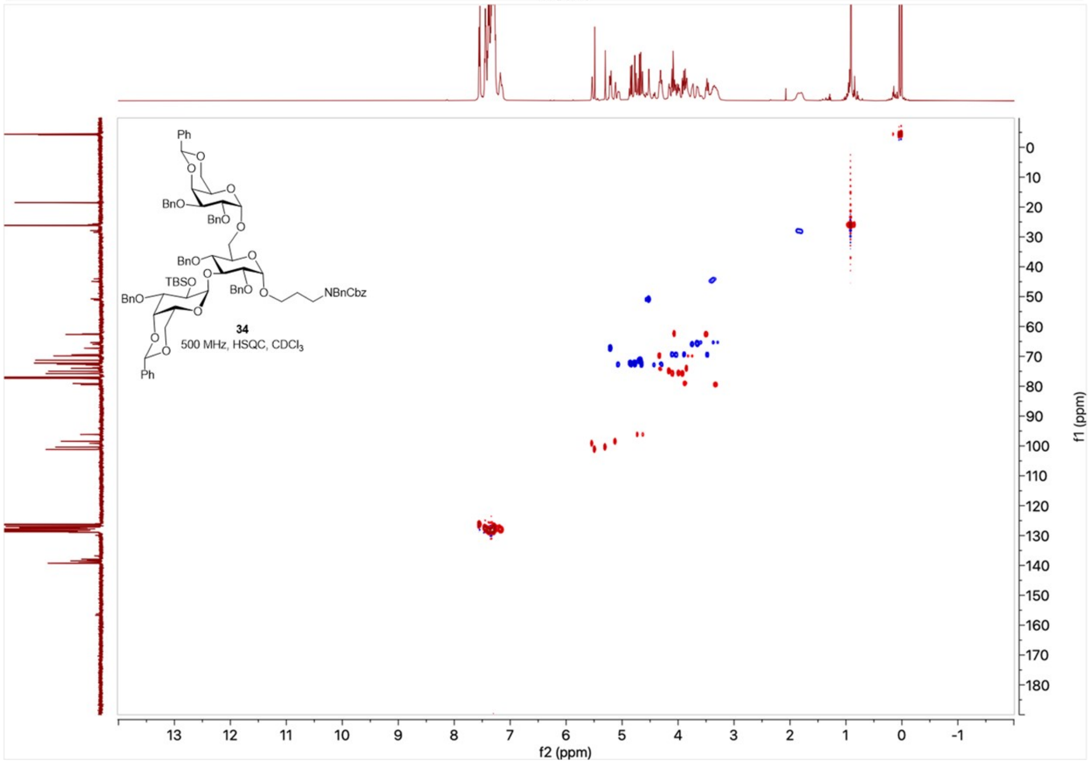
Ph
BnO
BnO
BnO
TBSO
BnO
NBnCbz
BnO
Ph
34
500 MHz, HSQC, CDCl3
f1 (ppm)
f2 (ppm)

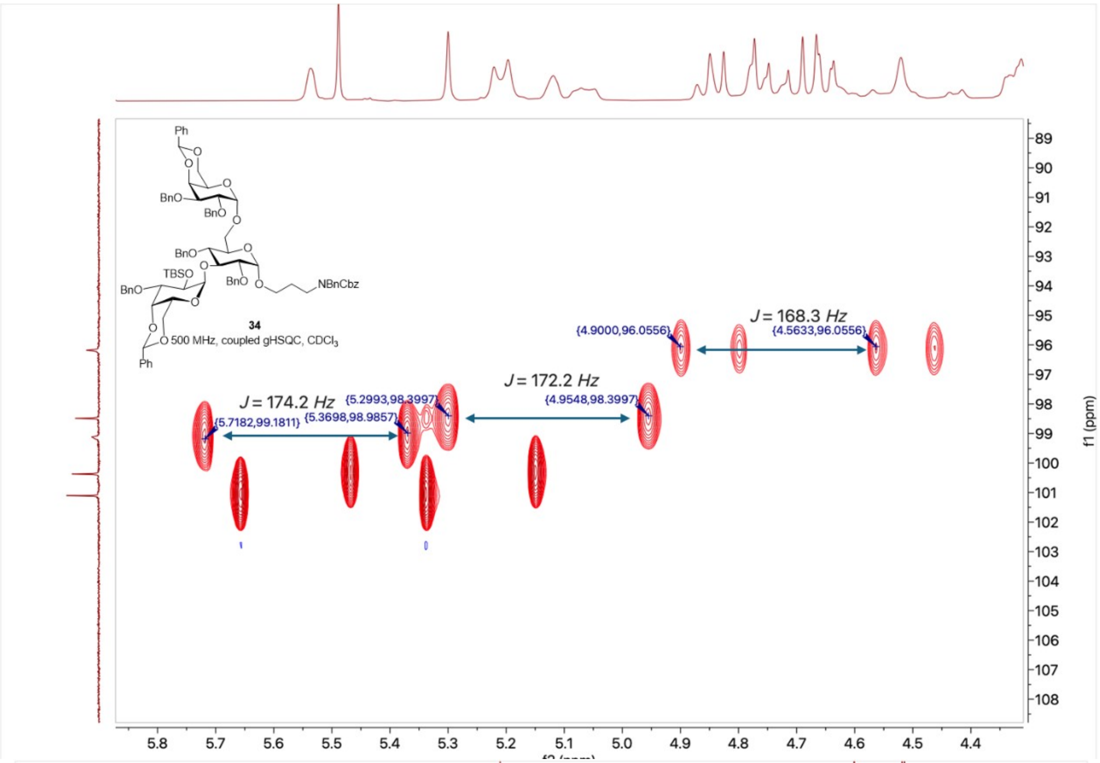
34
500 MHz, coupled gHSQC, CDCl₃
J = 174.2 Hz
J = 172.2 Hz
J = 168.3 Hz
{5.7182,99.1811}
{5.3698,98.9857}
{5.2993,98.3997}
{4.9548,98.3997}
{4.9000,96.0556}
{4.5633,96.0556}
f1 (ppm)
f2 (ppm)

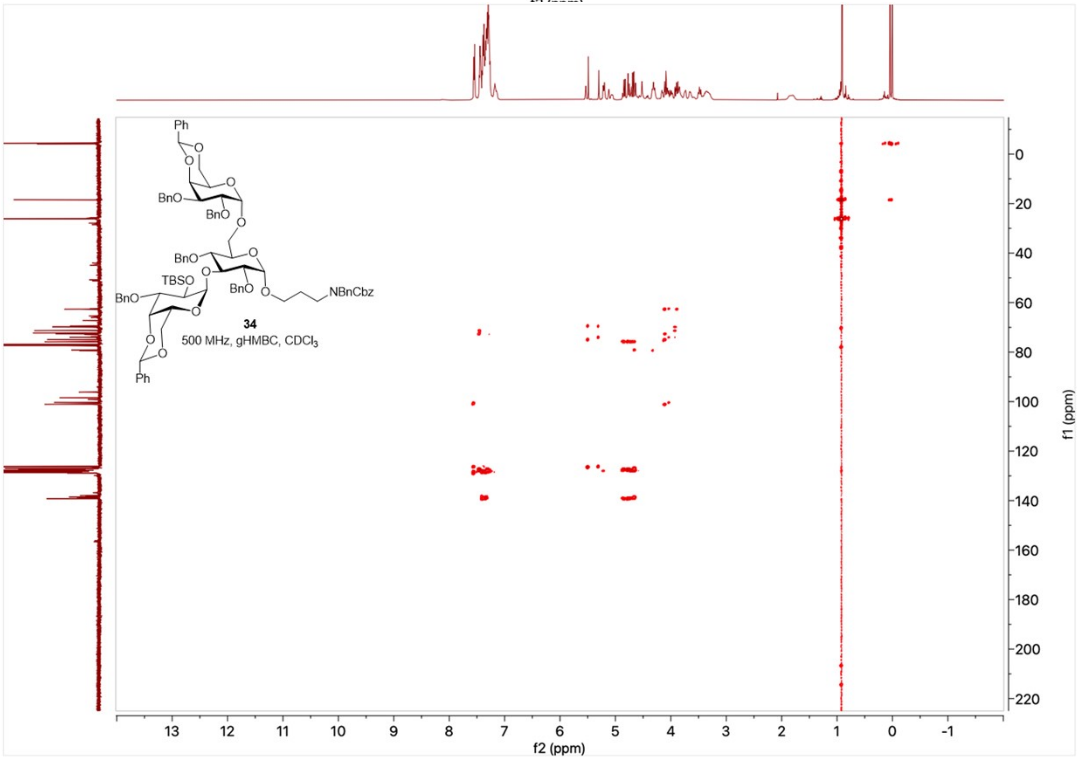
34
500 MHz, gHMBC, CDCl₃
f1 (ppm)
f2 (ppm)

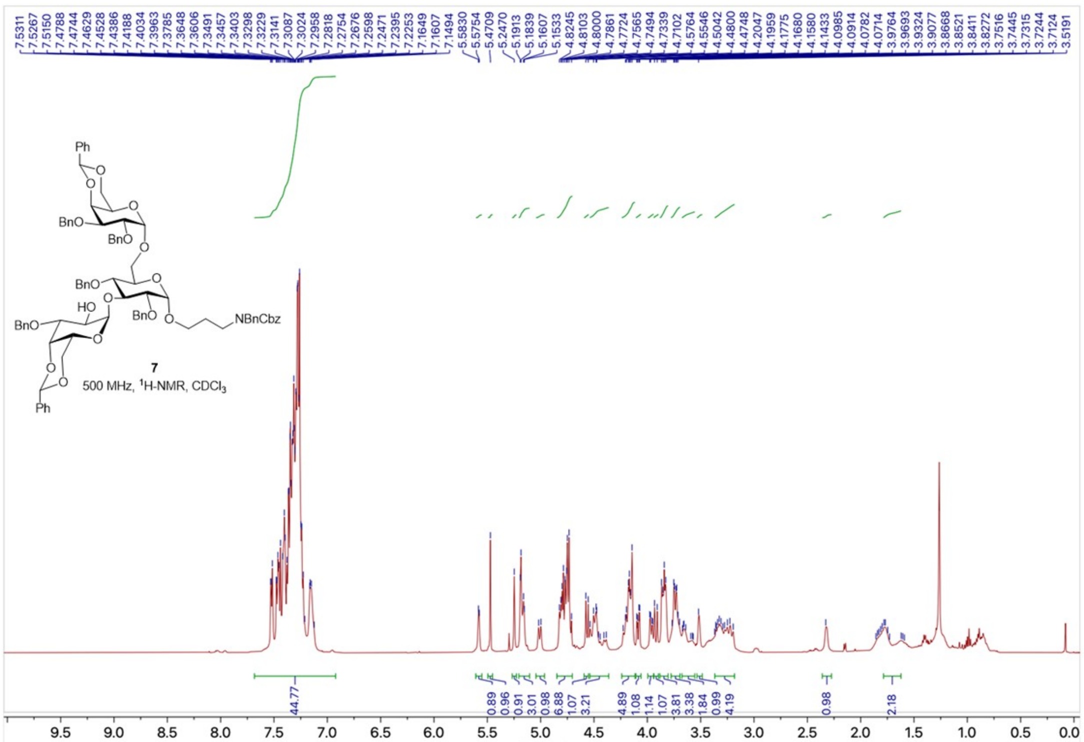
Ph
BnO
BnO
HO
NBnCbz
7
500 MHz, ¹H-NMR, CDCl₃
f1 (ppm)

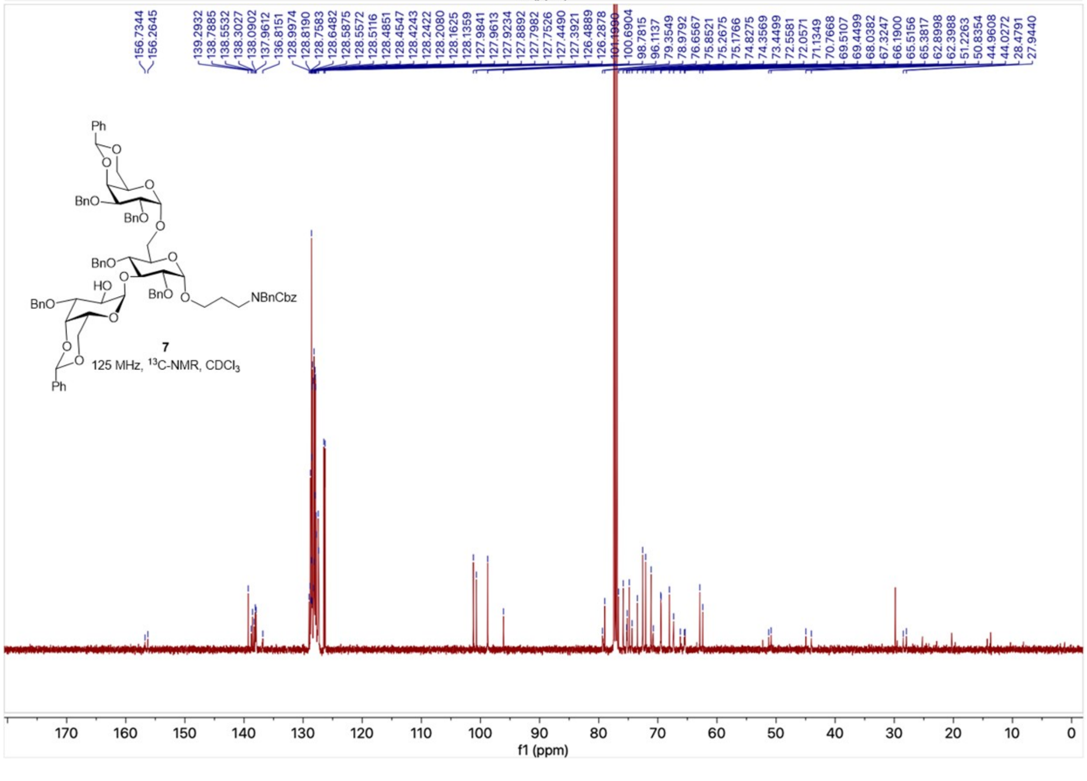
Ph
BnO
BnO
HO
NBnCbz
7
125 MHz, ¹³C-NMR, CDCl₃
f1 (ppm)

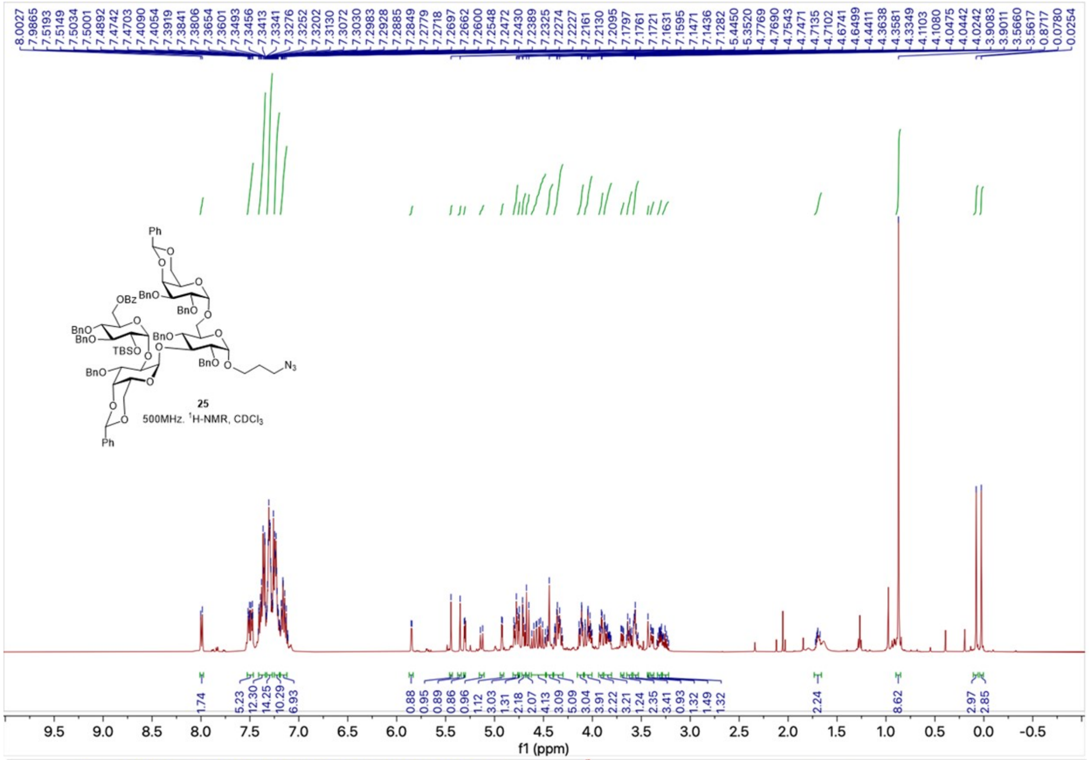

25
500MHz. 1H-NMR, CDCl3
f1 (ppm)

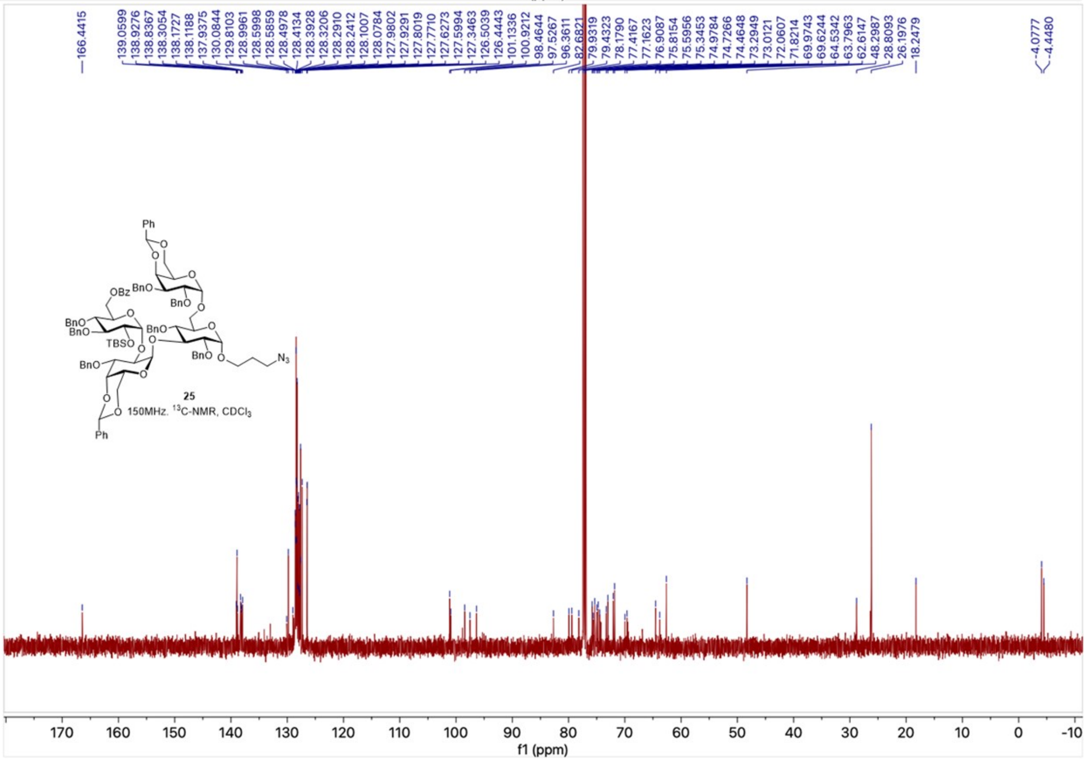

25
150MHz. 13C-NMR, CDCl3
f1 (ppm)

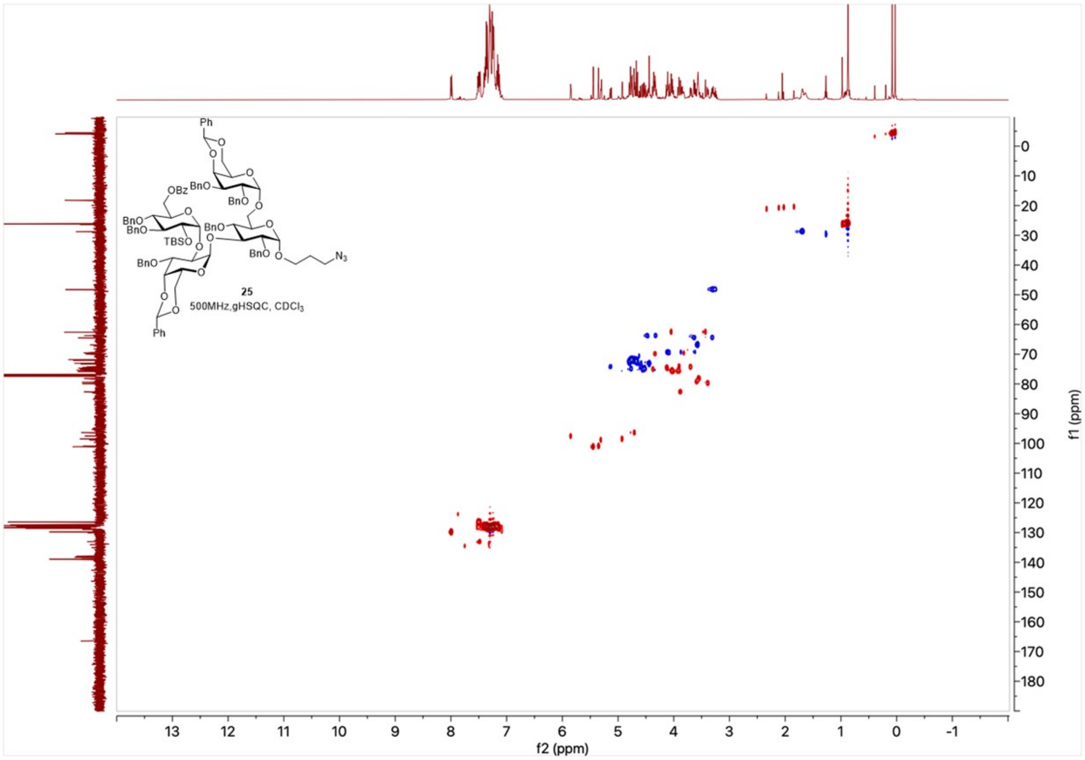

25
500MHz,gHSQC, CDCl$_3$
f2 (ppm)
f1 (ppm)

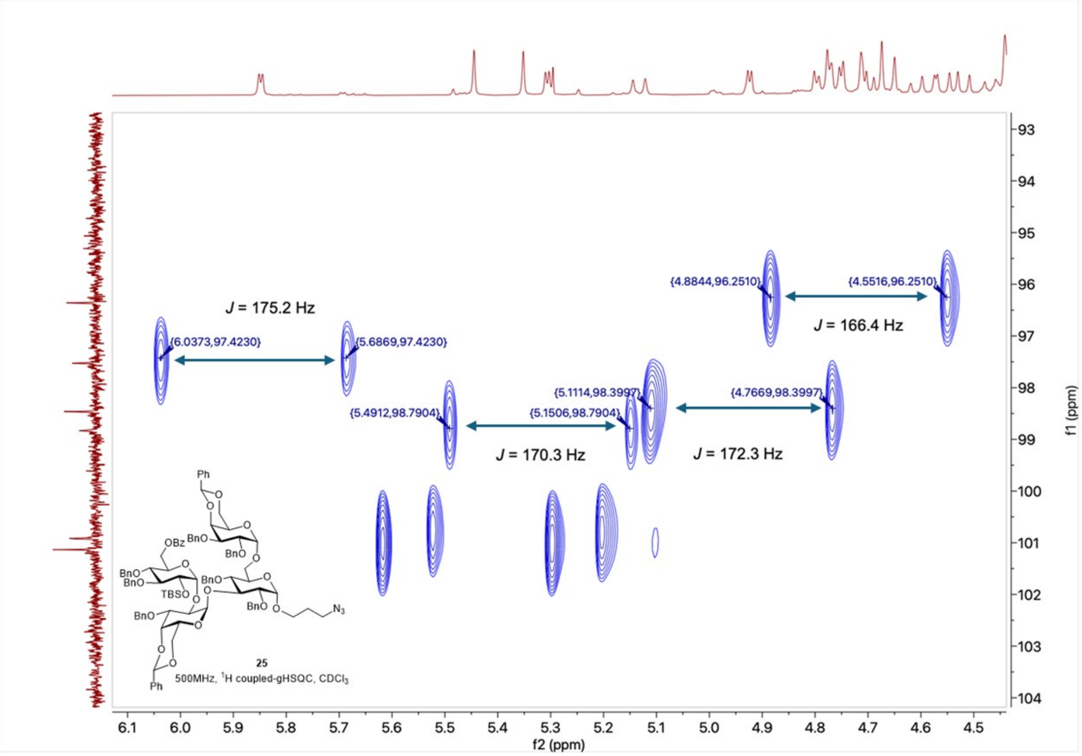

J = 175.2 Hz
J = 166.4 Hz
J = 170.3 Hz
J = 172.3 Hz
{6.0373,97.4230}
{5.6869,97.4230}
{4.8844,96.2510}
{4.5516,96.2510}
{5.1114,98.3997}
{4.7669,98.3997}
{5.4912,98.7904}
{5.1506,98.7904}
25
500MHz, ^{1}H coupled-gHSQC, CDCl$_3$
f2 (ppm)
f1 (ppm)

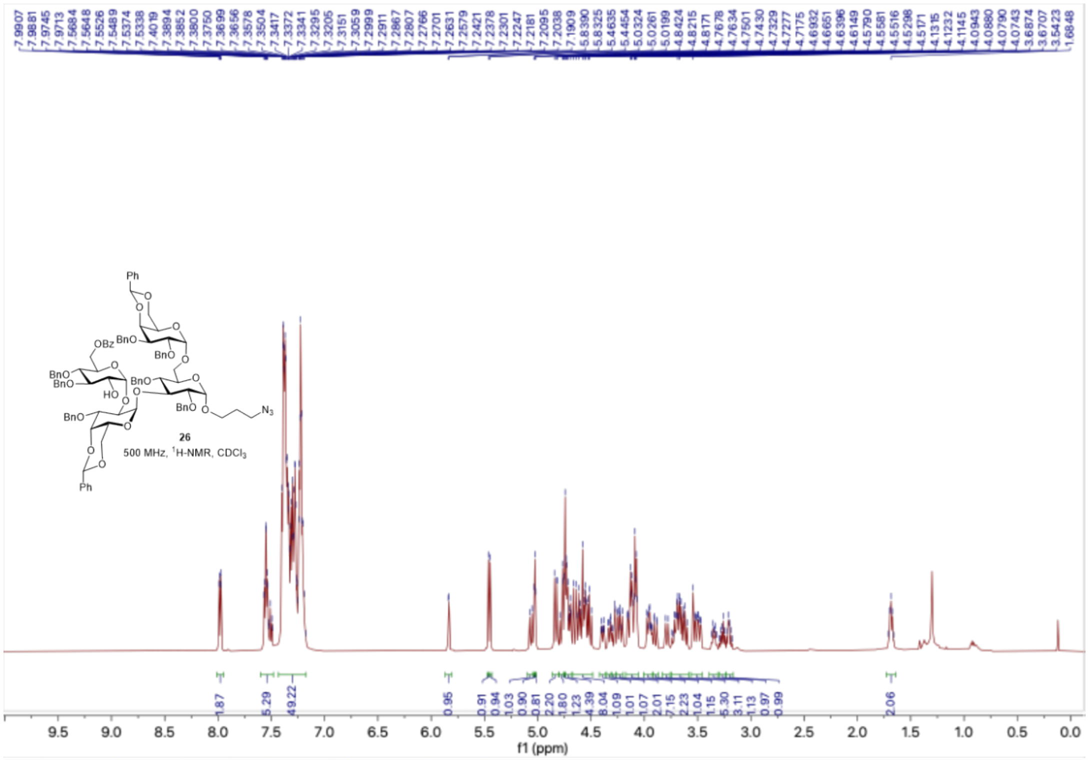
26
500 MHz, ^{1}H-NMR, CDCl$_3$
f1 (ppm)

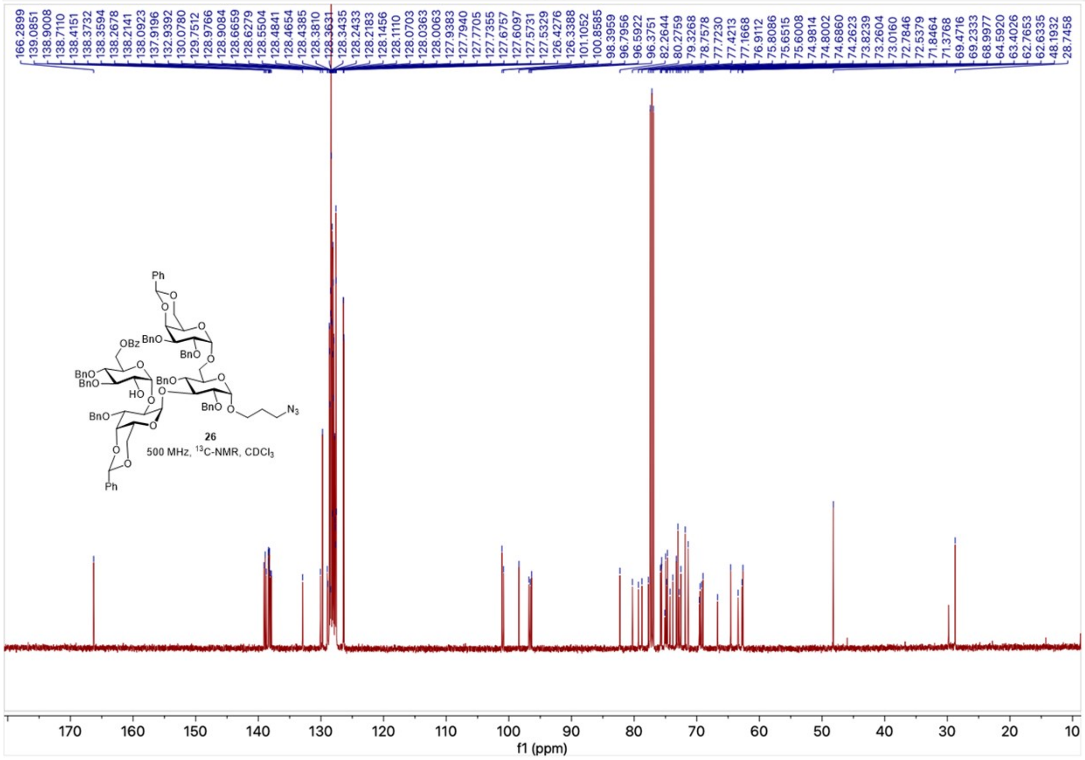
26
500 MHz, ^{13}C-NMR, CDCl$_3$
f1 (ppm)

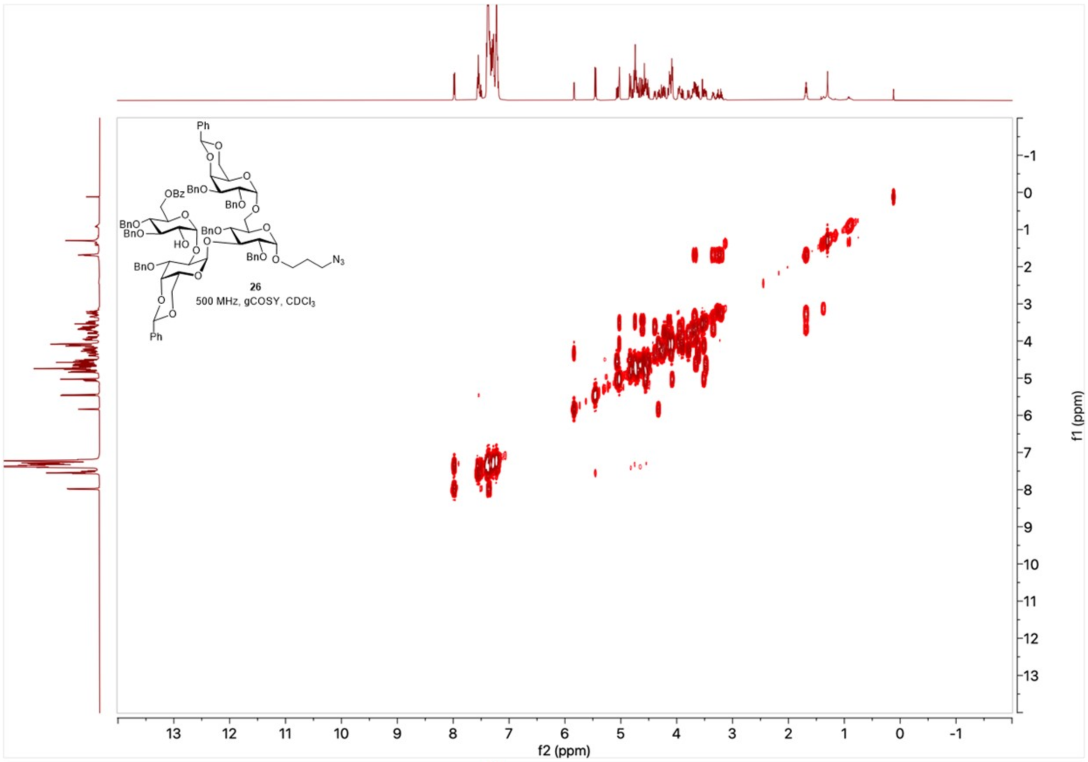
26
500 MHz, gCOSY, CDCl3
f1 (ppm)
f2 (ppm)

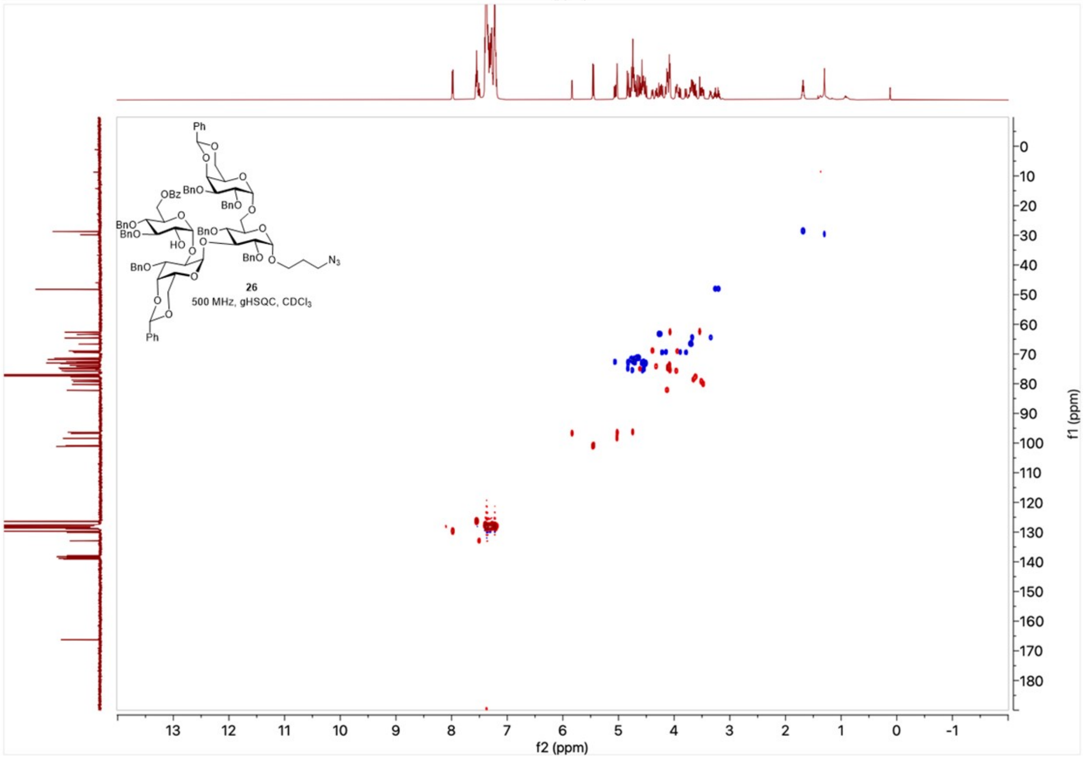
26
500 MHz, gHSQC, CDCl3
f1 (ppm)
f2 (ppm)

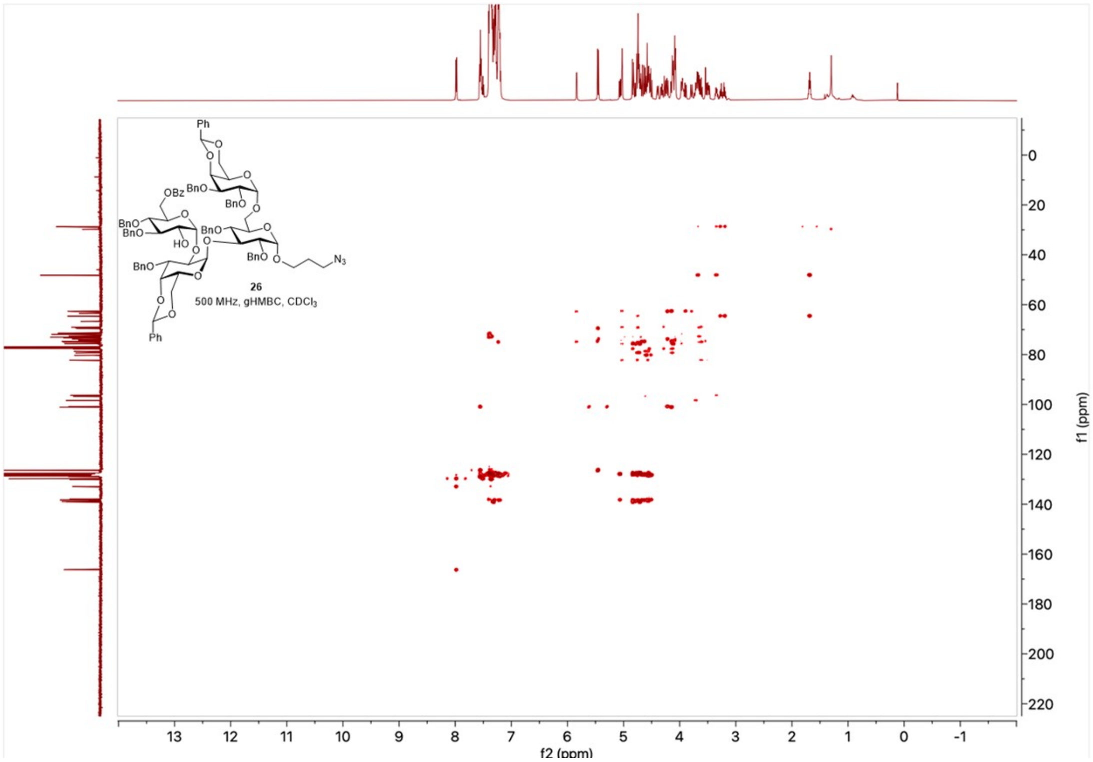
Ph
BnO
BnO
OBz
BnO
BnO
HO
BnO
BnO
BnO
N3
BnO
Ph
26
500 MHz, gHMBC, CDCl3
f1 (ppm)
f2 (ppm)

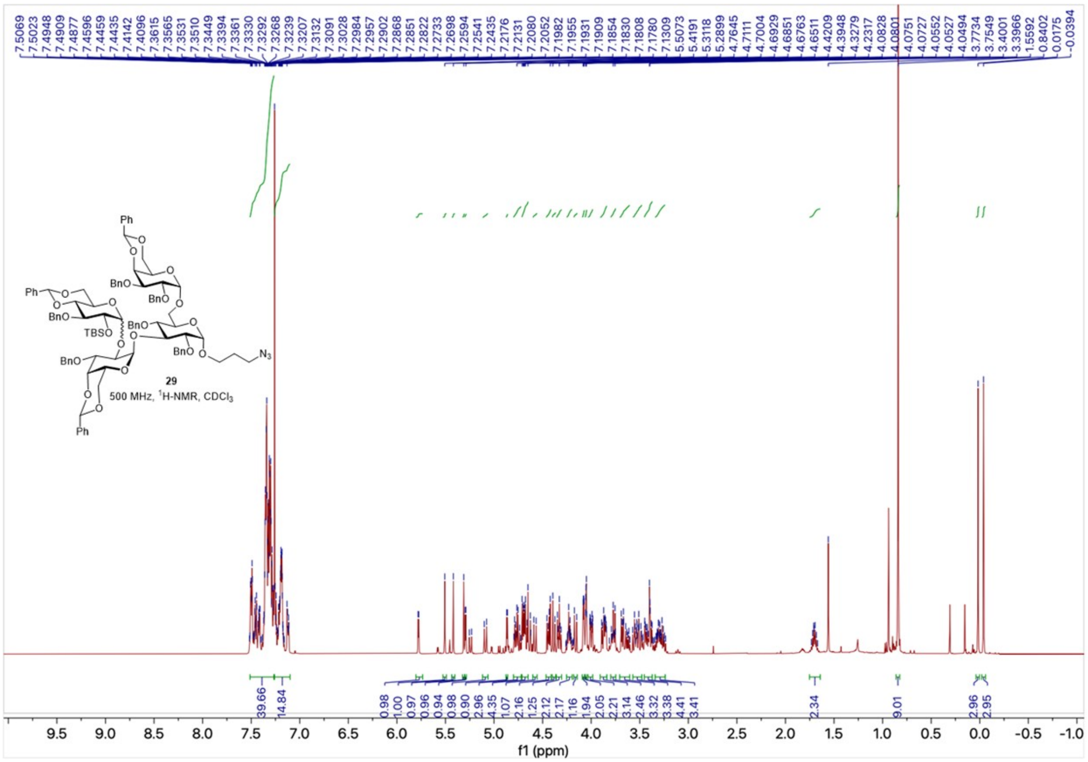
29
500 MHz, ¹H-NMR, CDCl₃
f1 (ppm)

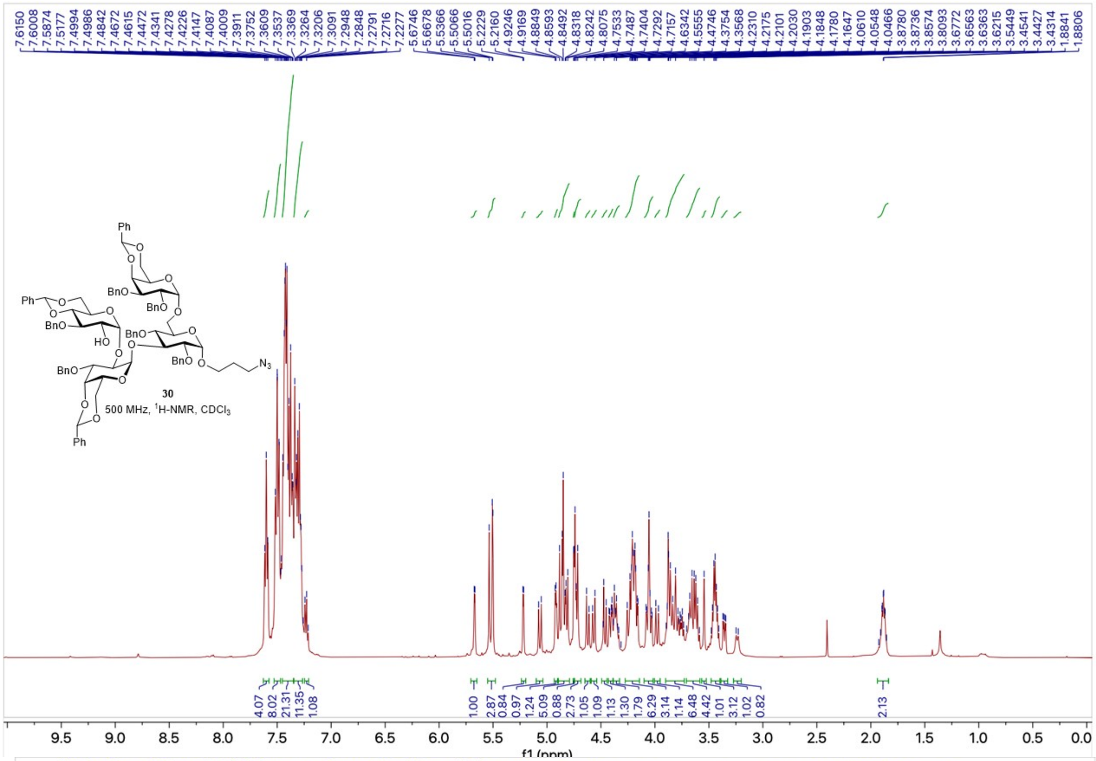
7.6150
7.6008
7.5874
7.5177
7.4994
7.4986
7.4842
7.4672
7.4615
7.4472
7.4341
7.4278
7.4226
7.4147
7.4087
7.4009
7.3911
7.3752
7.3609
7.3537
7.3369
7.3264
7.3206
7.3091
7.2948
7.2848
7.2791
7.2716
7.2277
5.6746
5.6678
5.5366
5.5066
5.5016
5.2229
5.2160
4.9246
4.9169
4.8849
4.8593
4.8492
4.8318
4.8242
4.8075
4.7533
4.7487
4.7404
4.7292
4.7157
4.6342
4.5555
4.4746
4.3754
4.3568
4.2310
4.2175
4.2101
4.2030
4.1903
4.1848
4.1780
4.1647
4.0610
4.0548
4.0466
3.8780
3.8736
3.8574
3.8093
3.6772
3.6563
3.6363
3.6215
3.5449
3.4541
3.4427
3.4314
1.8841
1.8806
Ph
BnO
BnO
Ph
BnO
HO
BnO
BnO
BnO
O
N3
BnO
Ph
30
500 MHz, 1H-NMR, CDCl3
4.07
8.02
21.31
11.35
1.08
1.00
2.87
0.84
0.97
1.24
5.09
0.88
2.73
1.05
1.09
1.13
1.30
1.79
6.29
3.14
1.14
6.48
4.42
1.01
3.12
1.02
0.82
2.13
9.5 9.0 8.5 8.0 7.5 7.0 6.5 6.0 5.5 5.0 4.5 4.0 3.5 3.0 2.5 2.0 1.5 1.0 0.5 0.0
f1 (ppm)

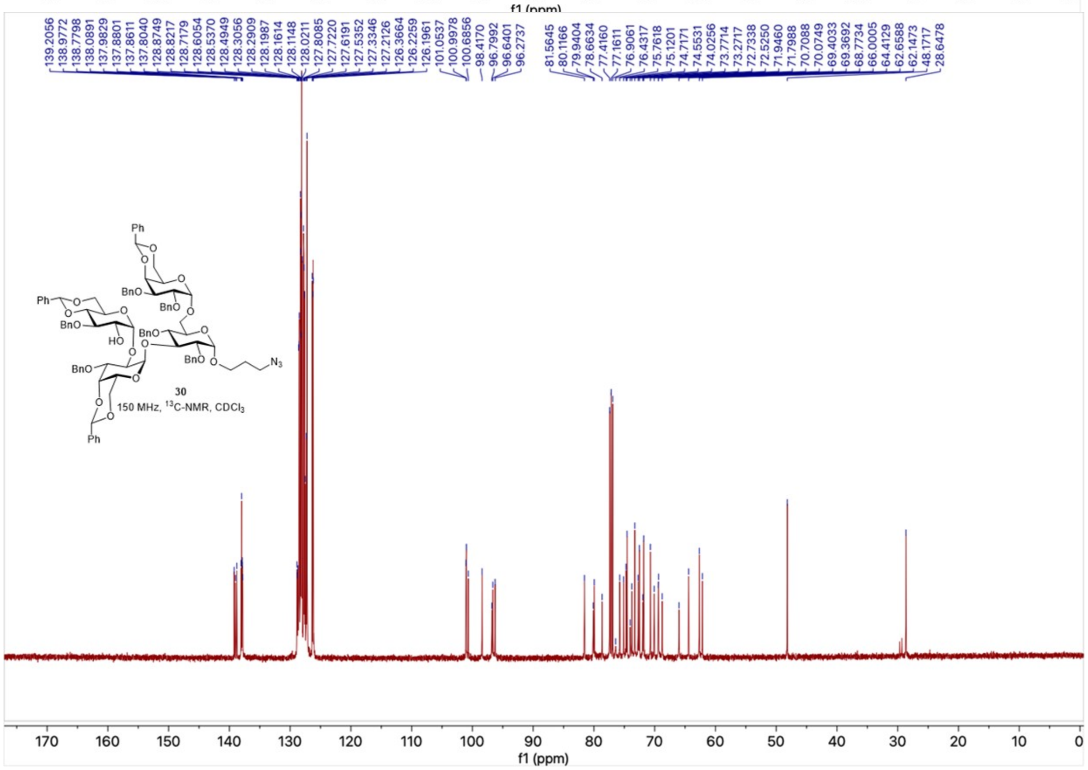
139.2056
138.9772
138.7798
138.0891
137.9829
137.8801
137.8611
137.8040
128.8749
128.8217
128.7179
128.6054
128.5370
128.4949
128.3056
128.2909
128.1987
128.1614
128.1148
128.0211
127.8085
127.7220
127.6191
127.5352
127.3346
127.2126
126.3664
126.2259
126.1961
101.0537
100.9978
100.6856
98.4170
96.7992
96.6401
96.2737
81.5645
80.1166
79.9404
78.6634
77.4160
77.1611
76.9061
76.4317
75.7618
75.1201
74.7171
74.5531
74.0256
73.7714
73.2717
72.7338
72.5250
71.9460
71.7988
70.7088
70.0749
69.4033
69.3692
68.7734
66.0005
64.4129
62.6588
62.1473
48.1717
28.6478
Ph
BnO
BnO
Ph
BnO
HO
BnO
BnO
BnO
N3
BnO
Ph
30
150 MHz, 13C-NMR, CDCl3
170 160 150 140 130 120 110 100 90 80 70 60 50 40 30 20 10 0
f1 (ppm)

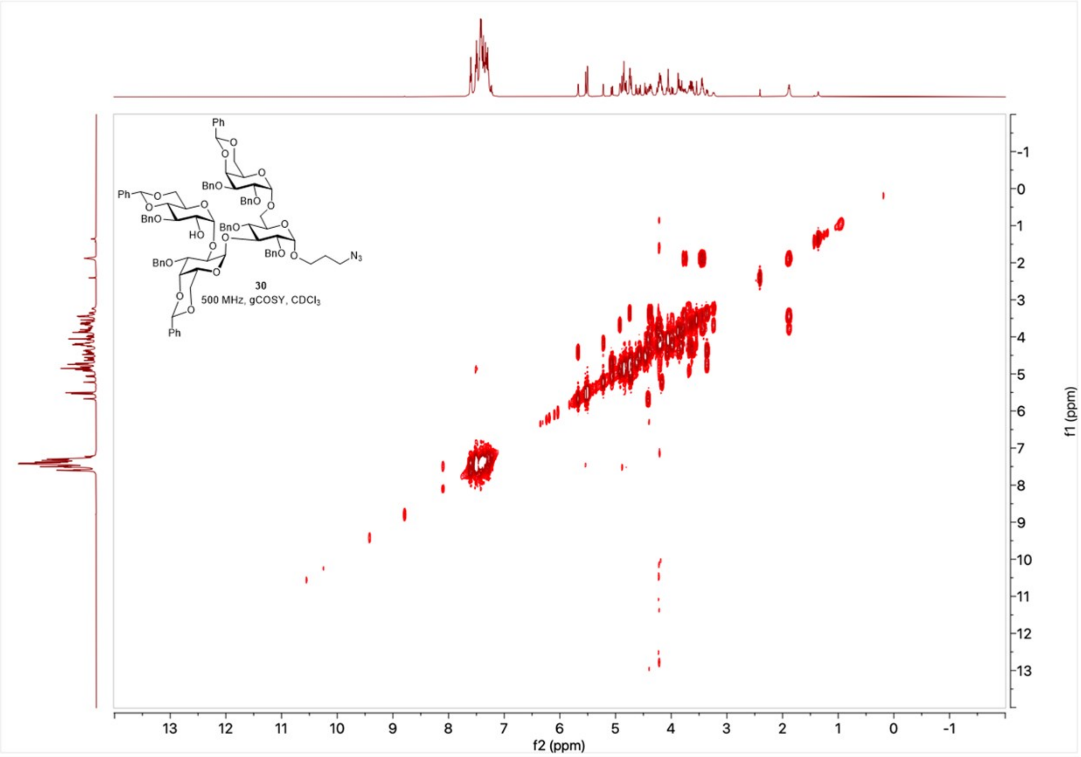
Ph
BnO
BnO
Ph
BnO
HO
BnO
BnO
N3
BnO
30
500 MHz, gCOSY, CDCl3
Ph
f1 (ppm)
f2 (ppm)

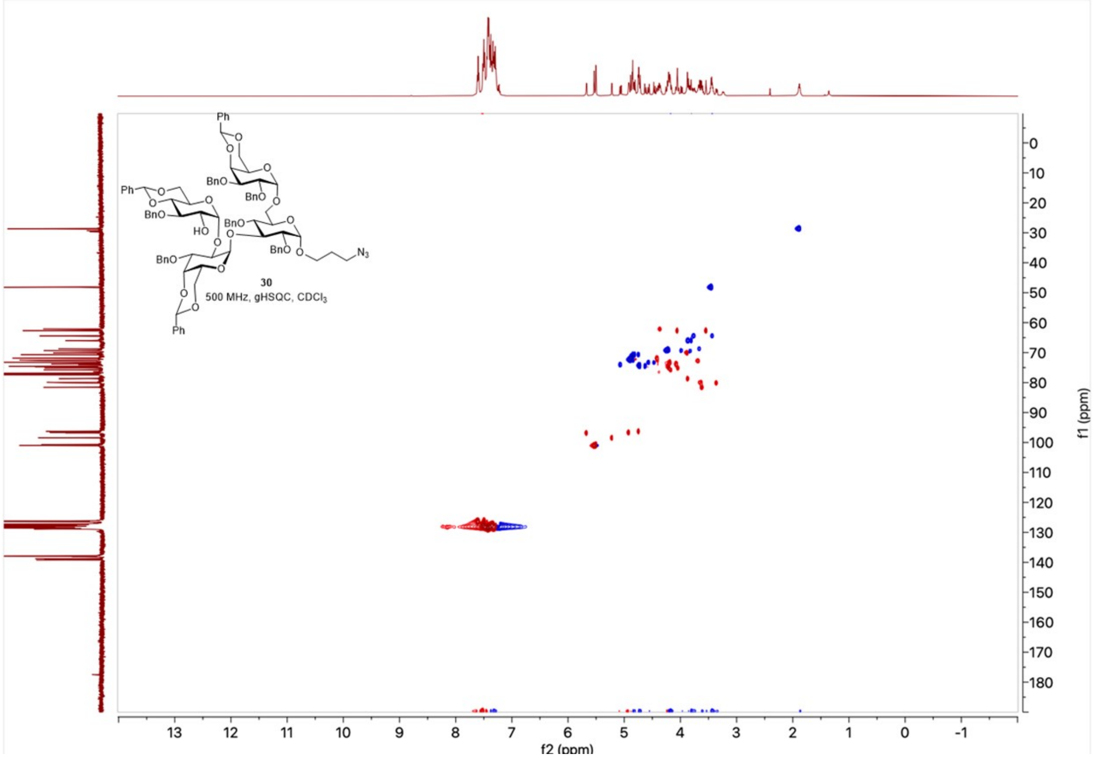
Ph
BnO
BnO
Ph
BnO
HO
BnO
BnO
N3
BnO
30
500 MHz, gHSQC, CDCl3
Ph
f1 (ppm)
f2 (ppm)

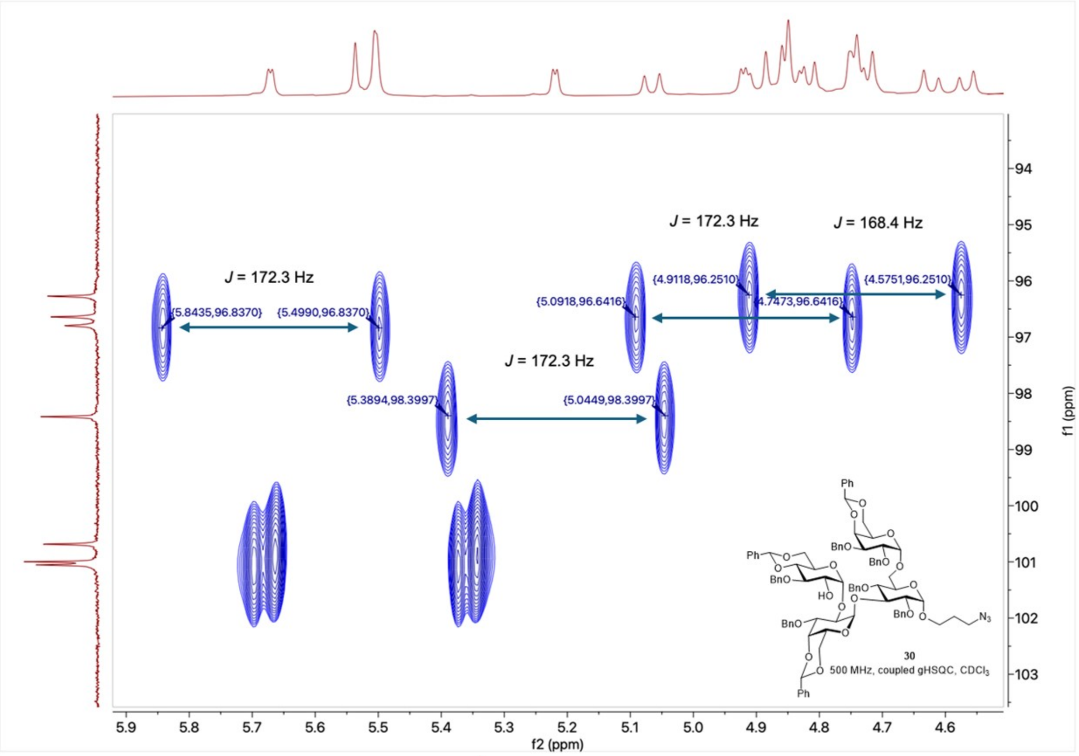
J = 172.3 Hz
J = 172.3 Hz
J = 168.4 Hz
J = 172.3 Hz
{5.8435,96.8370}
{5.4990,96.8370}
{5.0918,96.6416}
{4.9118,96.2510}
{4.7473,96.6416}
{4.5751,96.2510}
{5.3894,98.3997}
{5.0449,98.3997}
30
500 MHz, coupled gHSQC, CDCl3
f2 (ppm)
f1 (ppm)

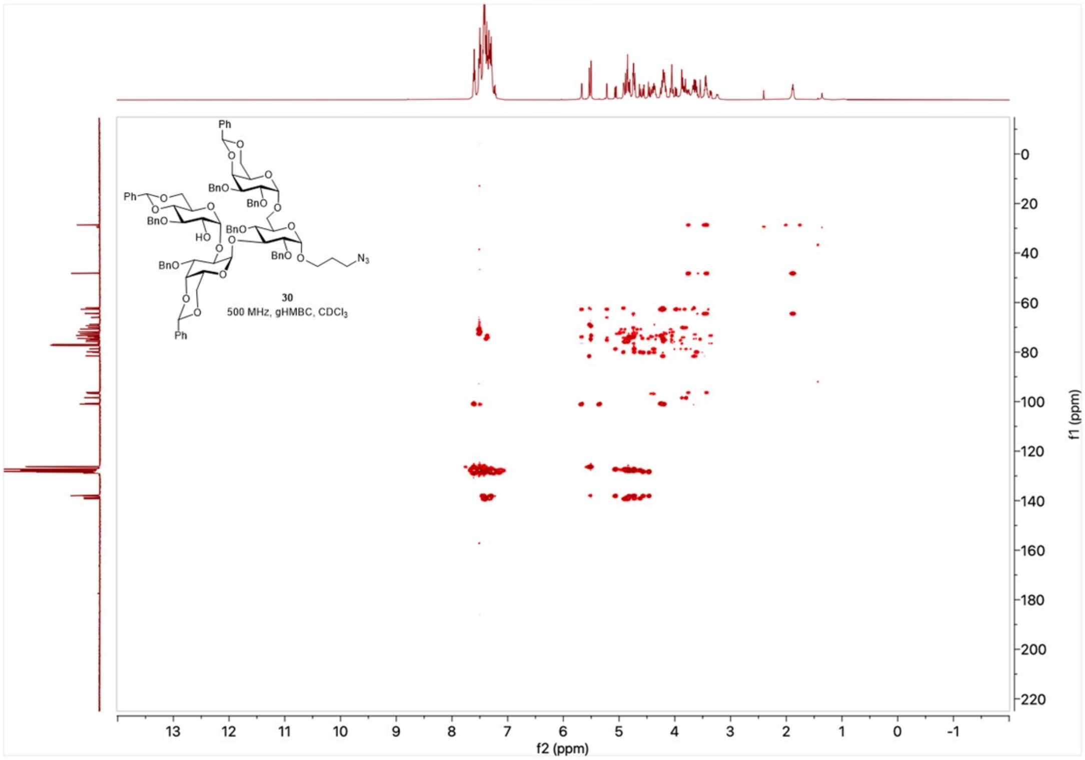
30
500 MHz, gHMBC, CDCl3
f2 (ppm)
f1 (ppm)

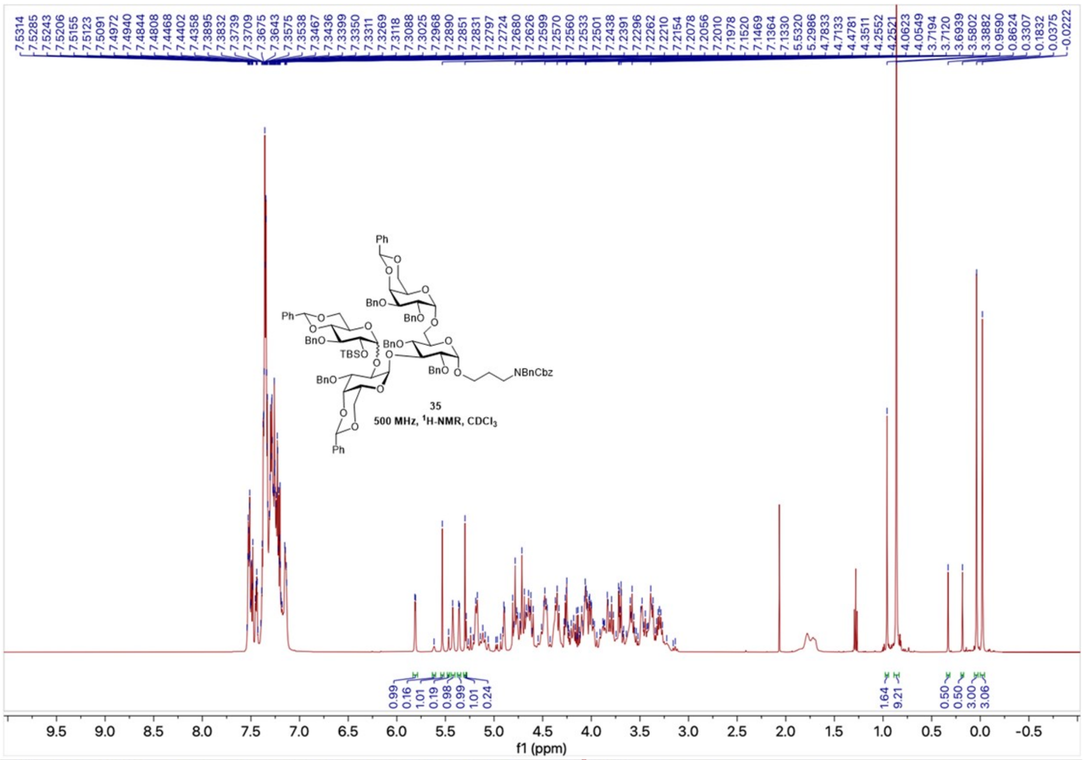
7.5314
7.5285
7.5206
7.5155
7.5123
7.5091
7.4972
7.4940
7.4844
7.4808
7.4468
7.4402
7.4358
7.3895
7.3832
7.3739
7.3709
7.3675
7.3643
7.3575
7.3538
7.3467
7.3436
7.3399
7.3350
7.3311
7.3269
7.3118
7.3088
7.3025
7.2968
7.2890
7.2851
7.2831
7.2797
7.2724
7.2680
7.2626
7.2599
7.2570
7.2560
7.2533
7.2501
7.2438
7.2391
7.2296
7.2262
7.2210
7.2154
7.2078
7.2056
7.2010
7.1978
7.1520
7.1469
7.1364
7.1330
5.5320
5.2986
4.7833
4.7133
4.4781
4.3511
4.2552
4.2521
4.0623
4.0549
3.7194
3.7120
3.6939
3.5802
3.3882
0.9590
0.8624
0.3307
0.1832
0.0375
-0.0222
Ph
BnO
BnO
TBSO
NBnCbz
35
500 MHz, 1H-NMR, CDCl3
0.99
0.16
1.01
0.19
0.98
0.99
1.01
0.24
1.64
9.21
0.50
0.50
3.00
3.06
9.5 9.0 8.5 8.0 7.5 7.0 6.5 6.0 5.5 5.0 4.5 4.0 3.5 3.0 2.5 2.0 1.5 1.0 0.5 0.0 -0.5
f1 (ppm)

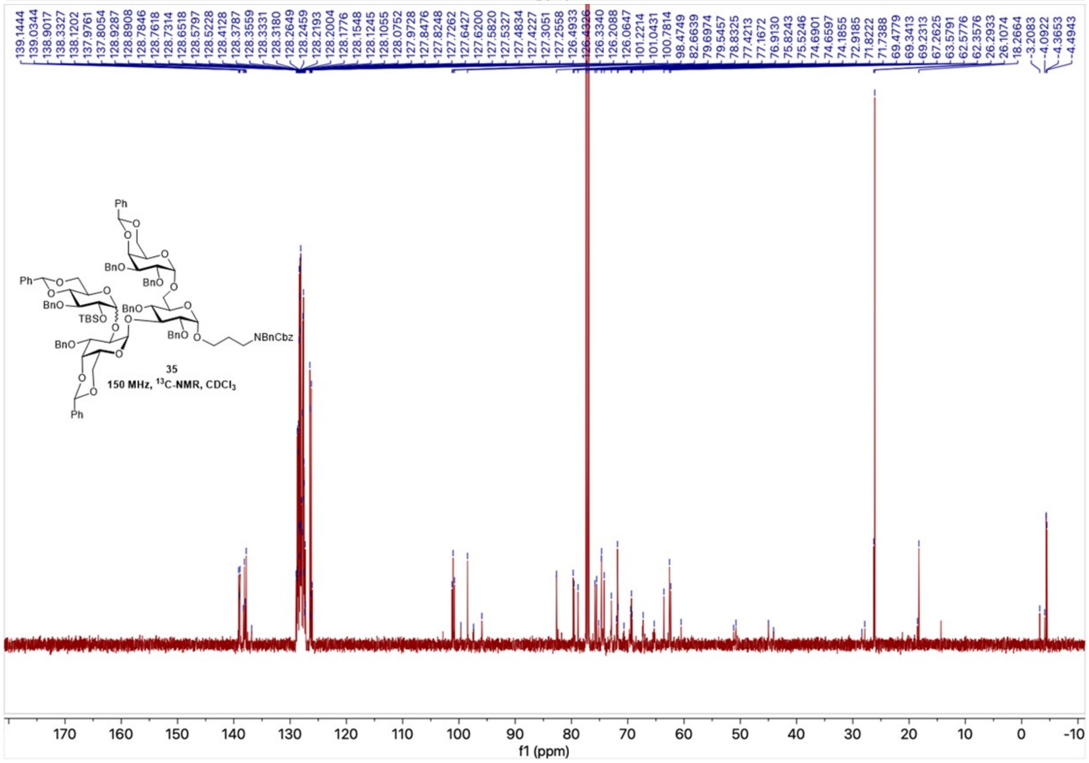
139.1444
139.0344
138.9017
138.3327
138.1202
137.9761
137.8054
128.9287
128.8908
128.7846
128.7618
128.7314
128.6518
128.5797
128.5228
128.4128
128.3787
128.3559
128.3331
128.3180
128.2649
128.2459
128.2193
128.2004
128.1776
128.1548
128.1245
128.1055
128.0752
127.9728
127.8476
127.8248
127.7262
127.6427
127.6200
127.5820
127.5327
127.4834
127.4227
127.3051
127.2558
126.4933
126.3340
126.2088
126.0647
101.2214
101.0431
100.7814
98.4749
82.6639
79.6974
79.5457
78.8325
77.4213
77.1672
76.9130
75.8243
75.5246
74.6901
74.6597
74.1855
72.9185
71.8222
71.7388
69.4779
69.3413
69.2313
67.2625
63.5791
62.5776
62.3576
26.2933
26.1074
18.2664
-3.2083
-4.0922
-4.3653
-4.4943
Ph
BnO
BnO
TBSO
NBnCbz
35
150 MHz, 13C-NMR, CDCl3
170 160 150 140 130 120 110 100 90 80 70 60 50 40 30 20 10 0 -10
f1 (ppm)

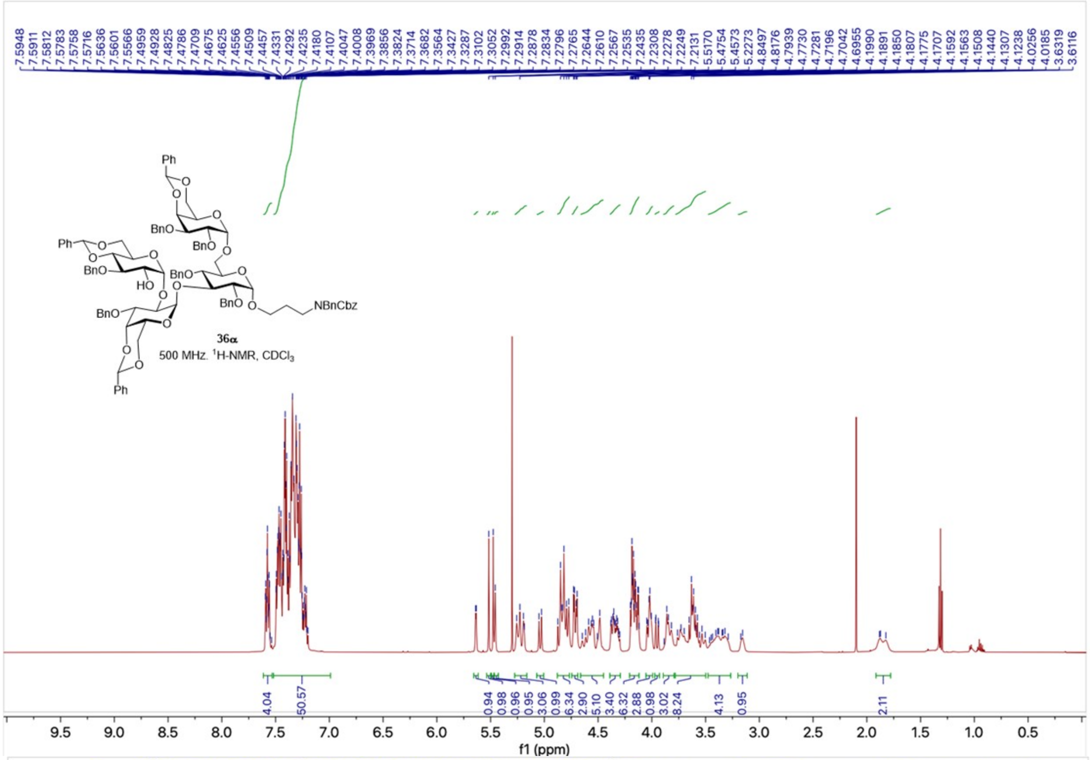
36α
500 MHz. ¹H-NMR, CDCl₃
f1 (ppm)

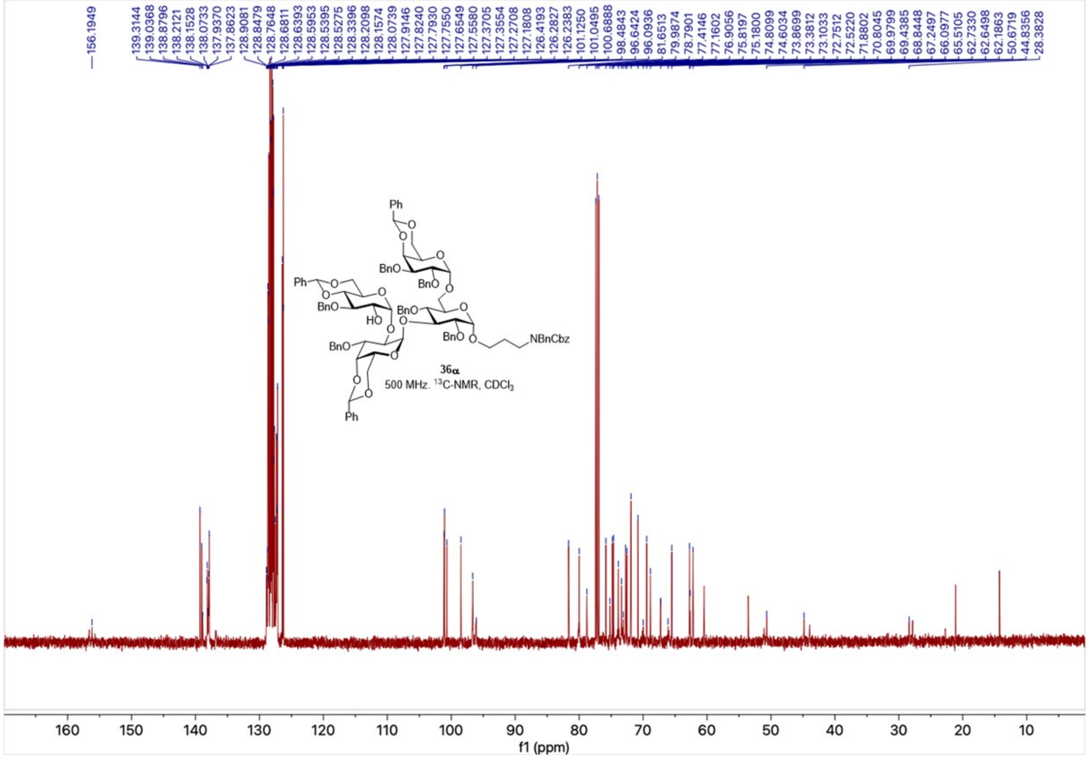
36α
500 MHz. ¹³C-NMR, CDCl₃
f1 (ppm)

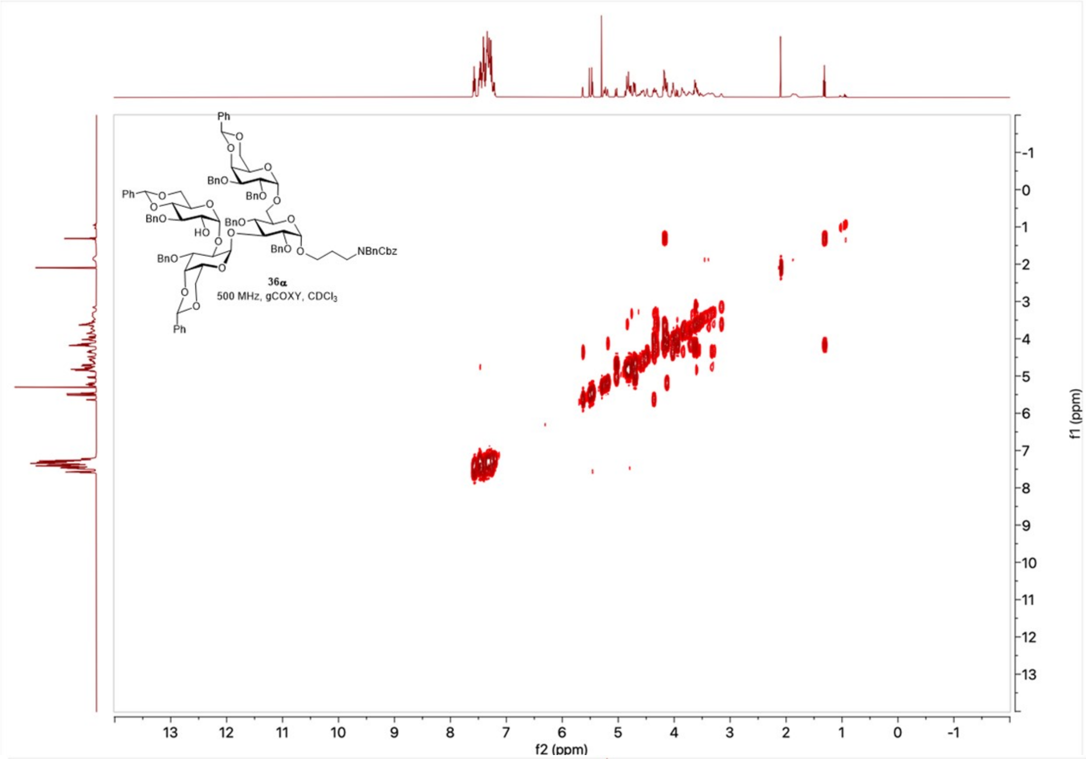
36α
500 MHz, gCOXY, CDCl3
f2 (ppm)
f1 (ppm)

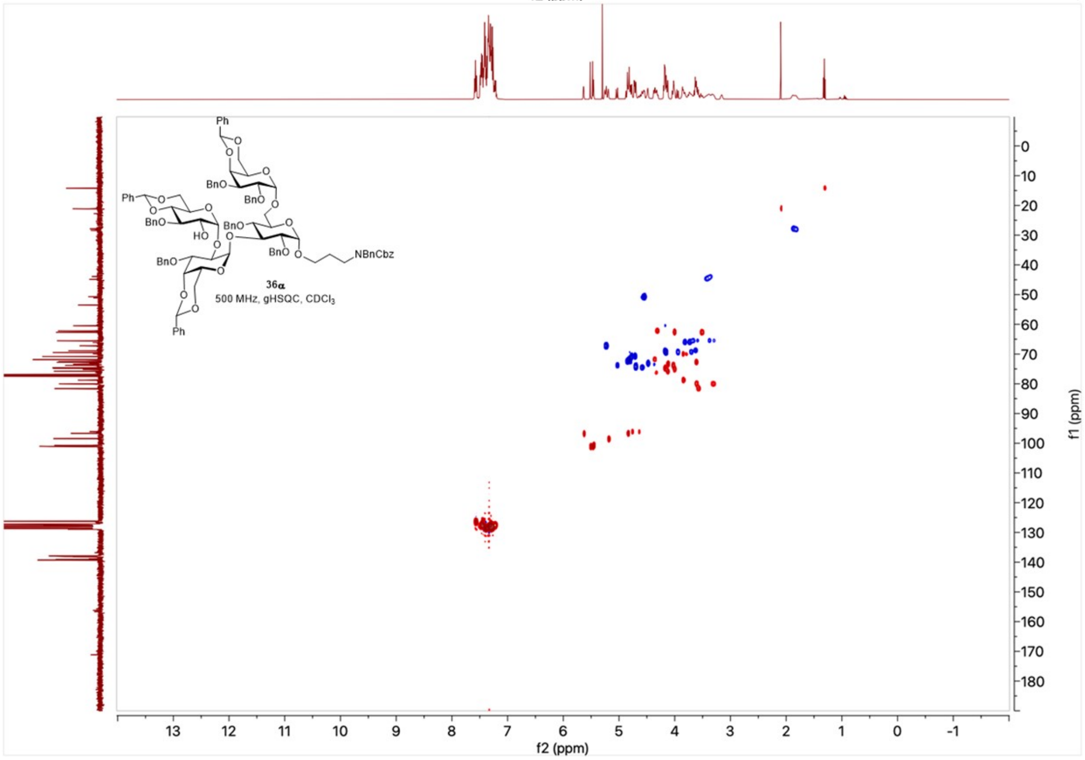
36α
500 MHz, gHSQC, CDCl3
f2 (ppm)
f1 (ppm)

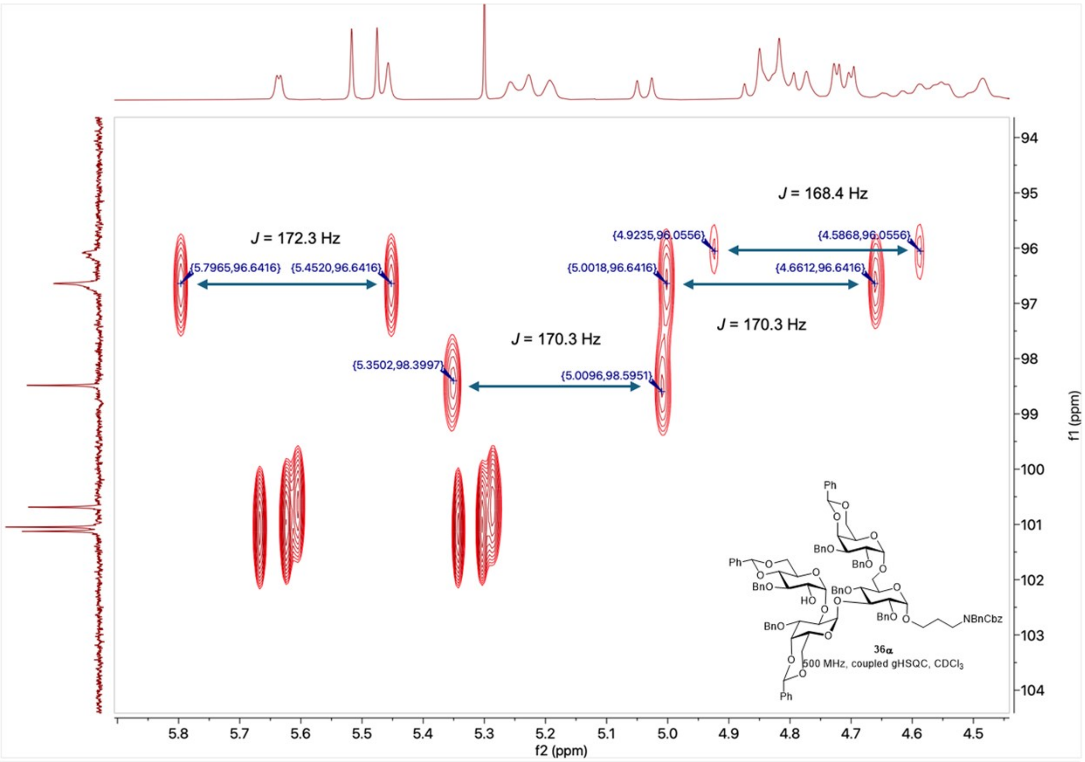
J = 172.3 Hz
J = 170.3 Hz
J = 170.3 Hz
J = 168.4 Hz
{5.7965,96.6416}
{5.4520,96.6416}
{5.3502,98.3997}
{5.0096,98.5951}
{5.0018,96.6416}
{4.6612,96.6416}
{4.9235,96.0556}
{4.5868,96.0556}
36α
500 MHz, coupled gHSQC, CDCl3
f2 (ppm)
f1 (ppm)

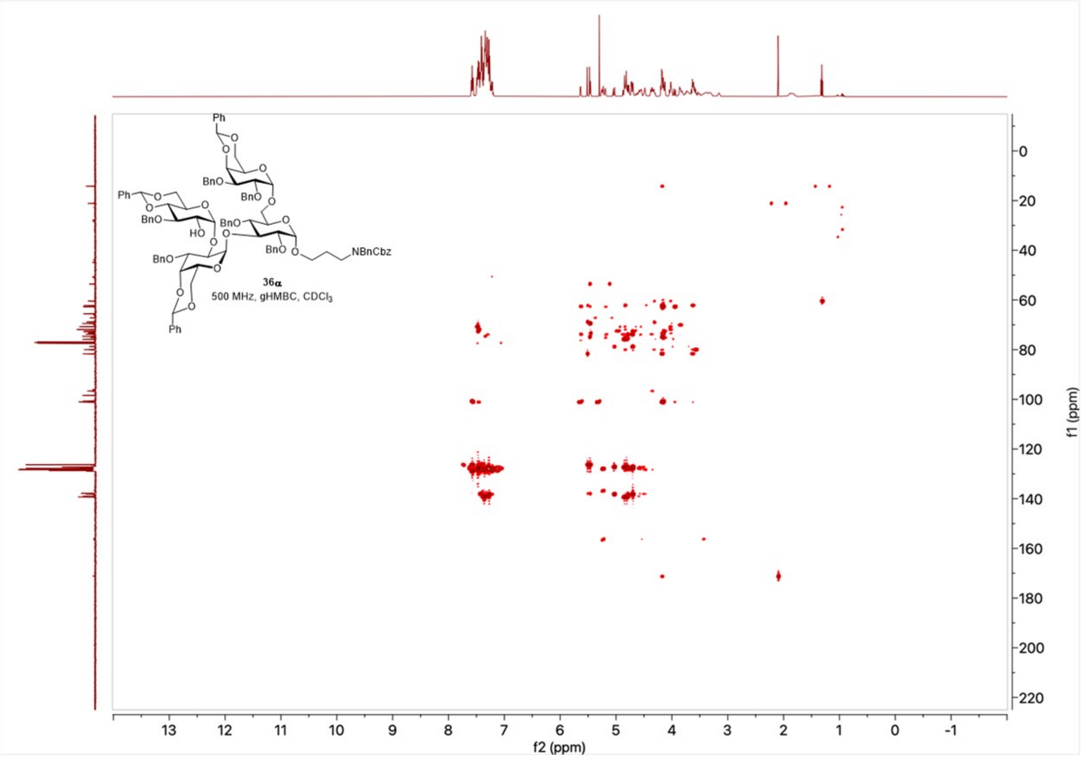
36α
500 MHz, gHMBC, CDCl3
f2 (ppm)
f1 (ppm)

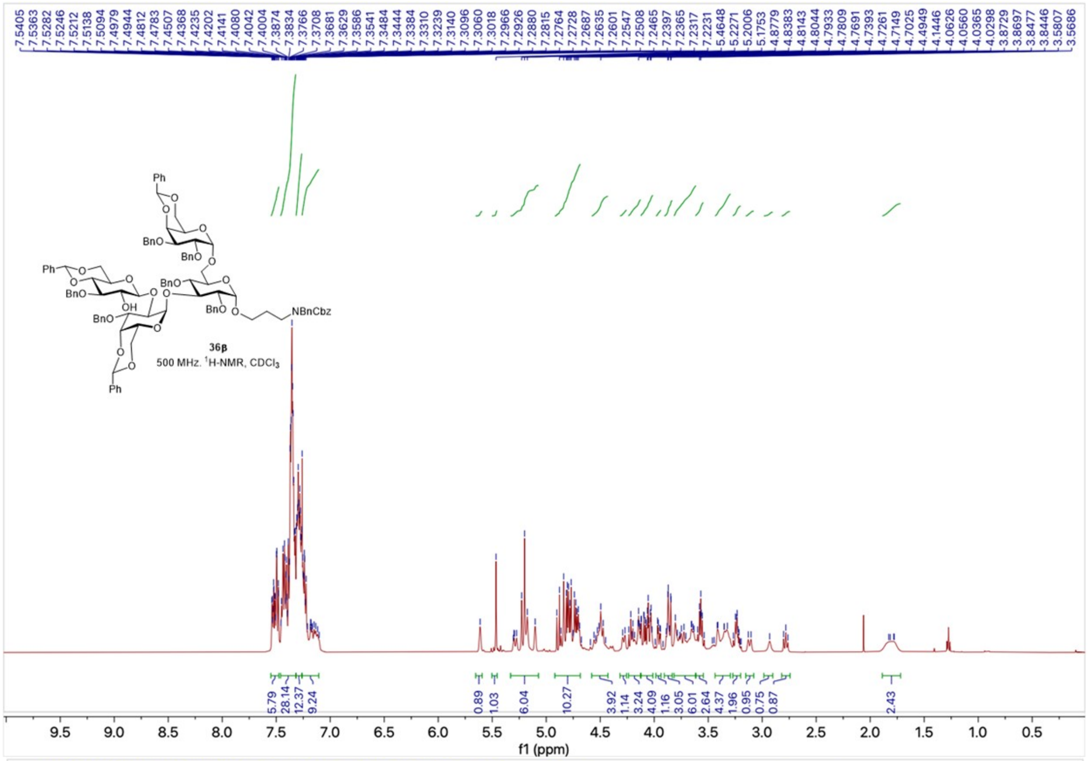

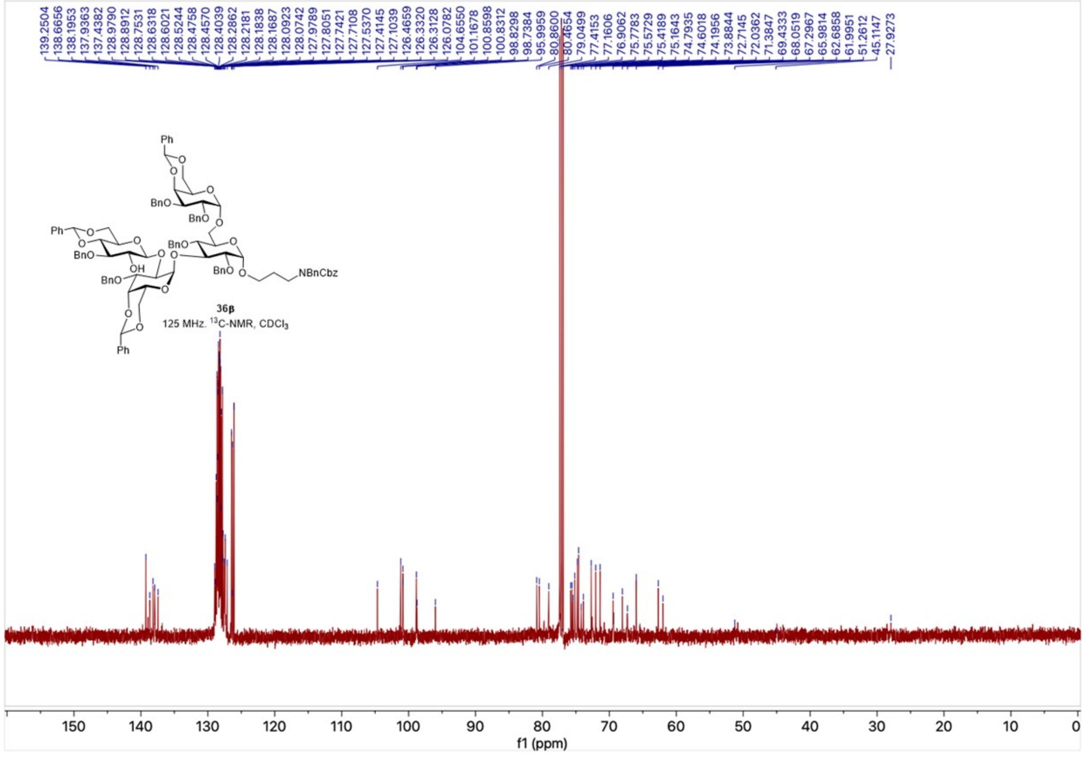

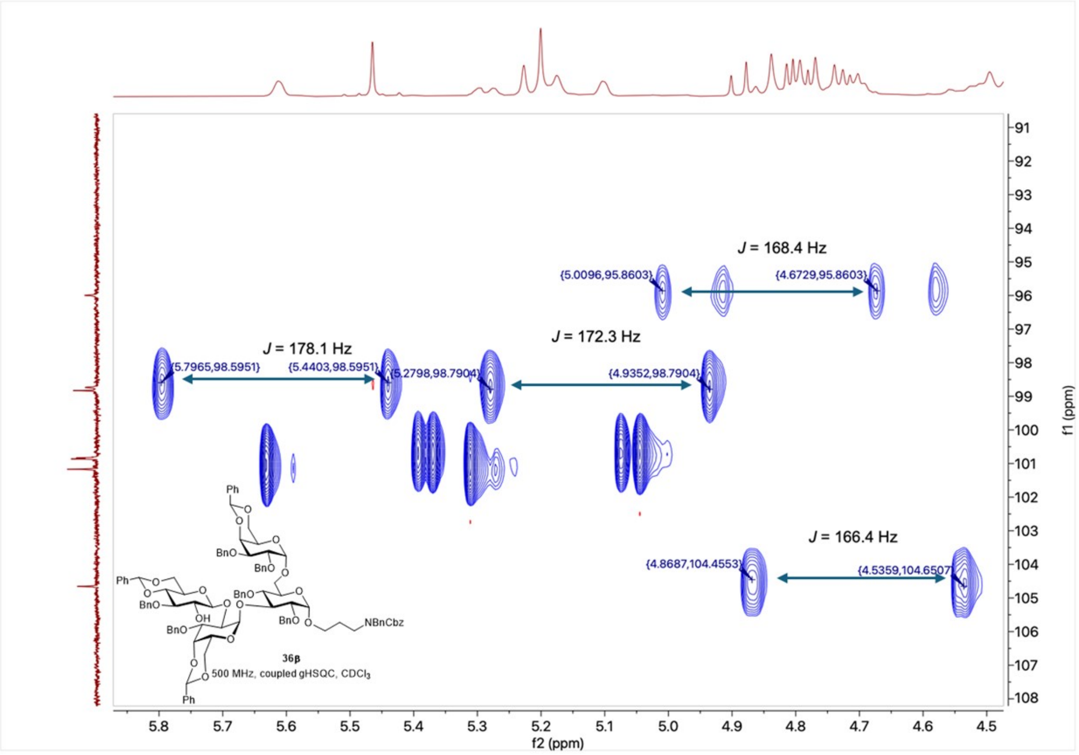

J = 168.4 Hz
{5.0096,95.8603}
{4.6729,95.8603}
J = 178.1 Hz
J = 172.3 Hz
{5.7965,98.5951}
{5.4403,98.5951}
{5.2798,98.7904}
{4.9352,98.7904}
J = 166.4 Hz
{4.8687,104.4553}
{4.5359,104.6507}
36β
500 MHz, coupled gHSQC, CDCl3
f2 (ppm)
f1 (ppm)

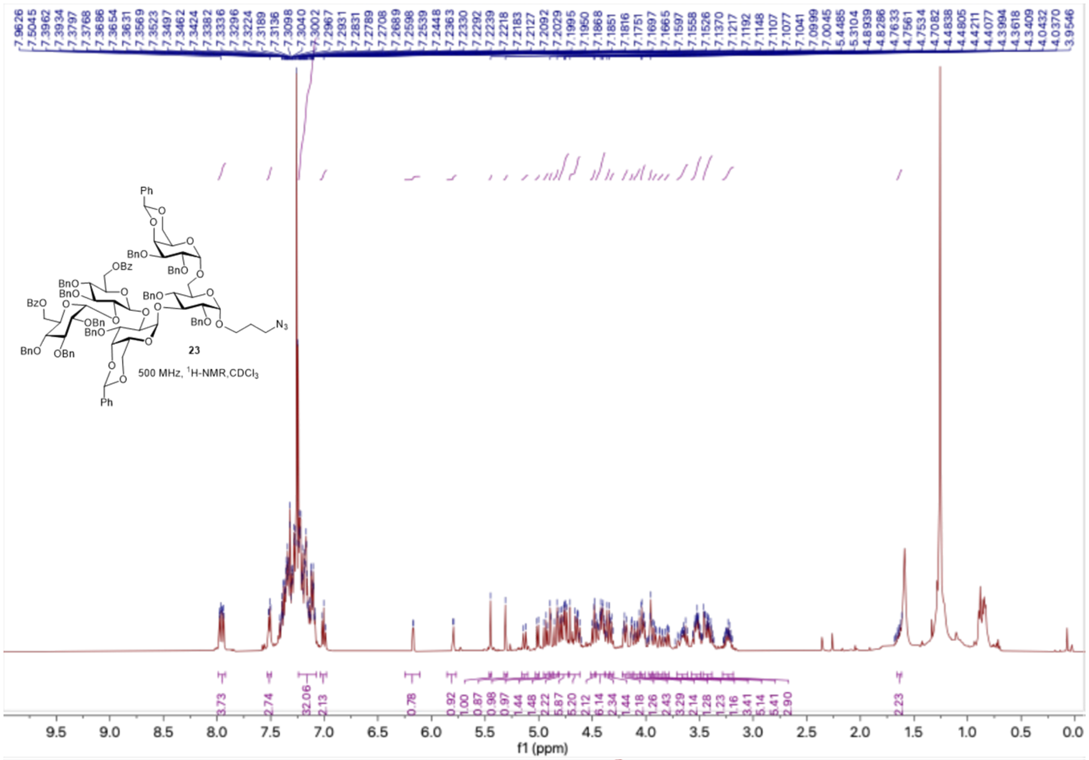

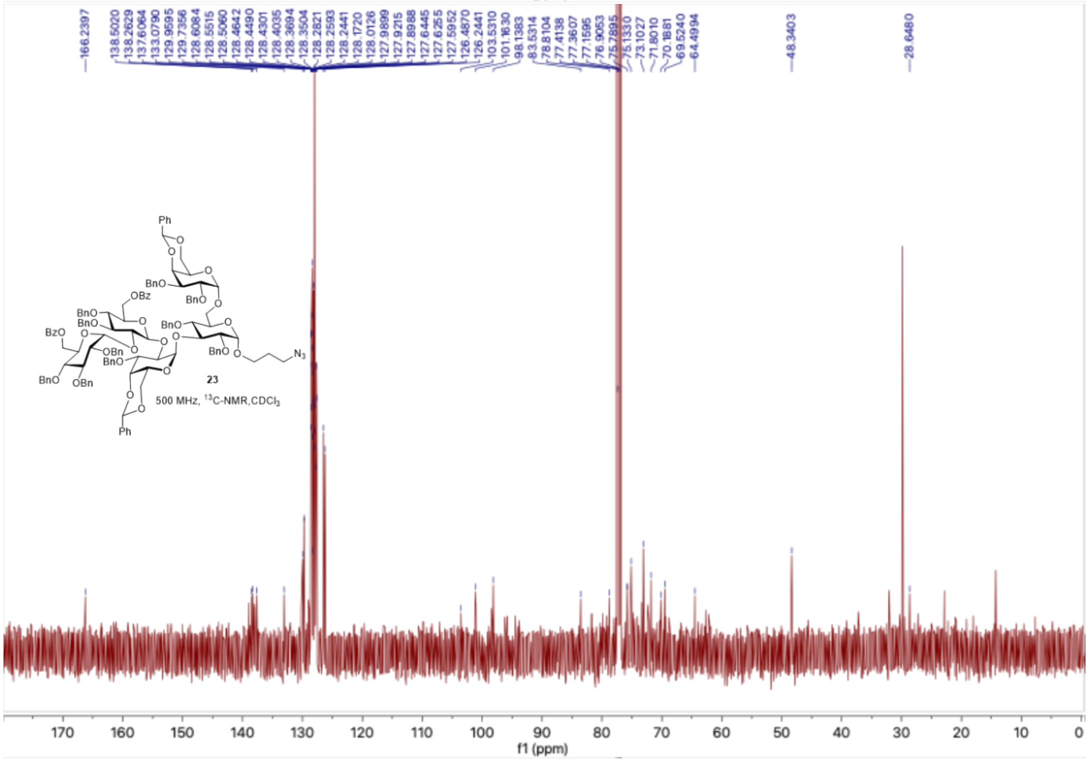

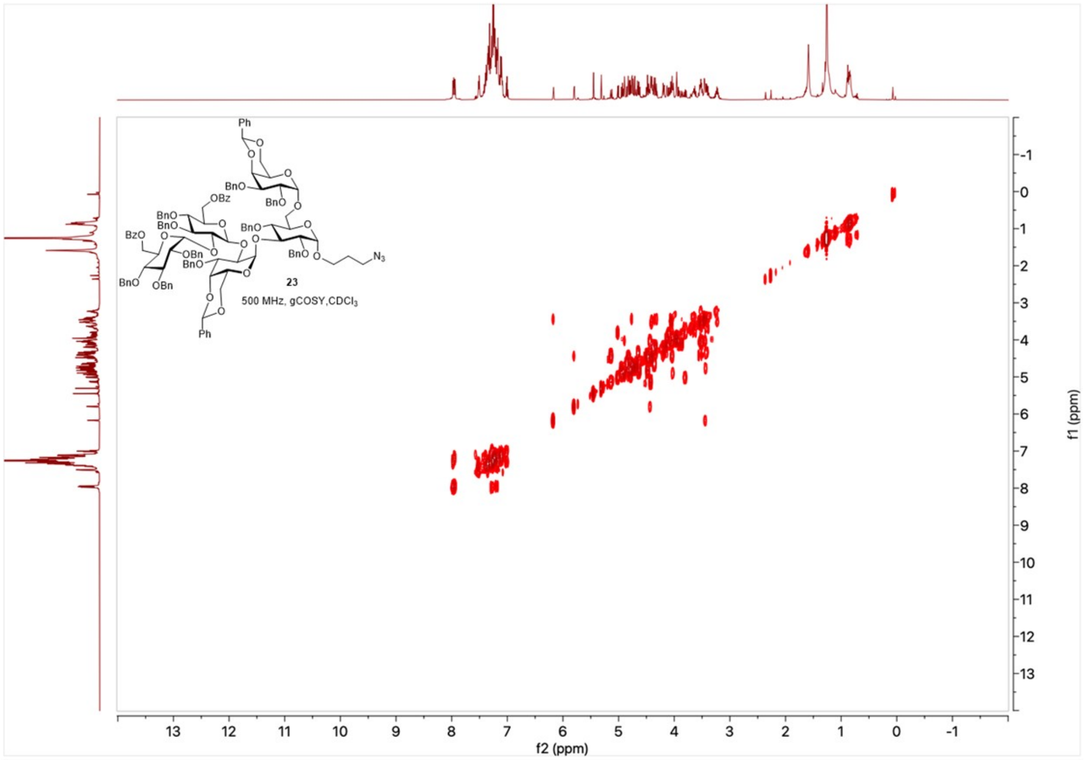
23
500 MHz, gCOSY,CDCl3
f1 (ppm)
f2 (ppm)

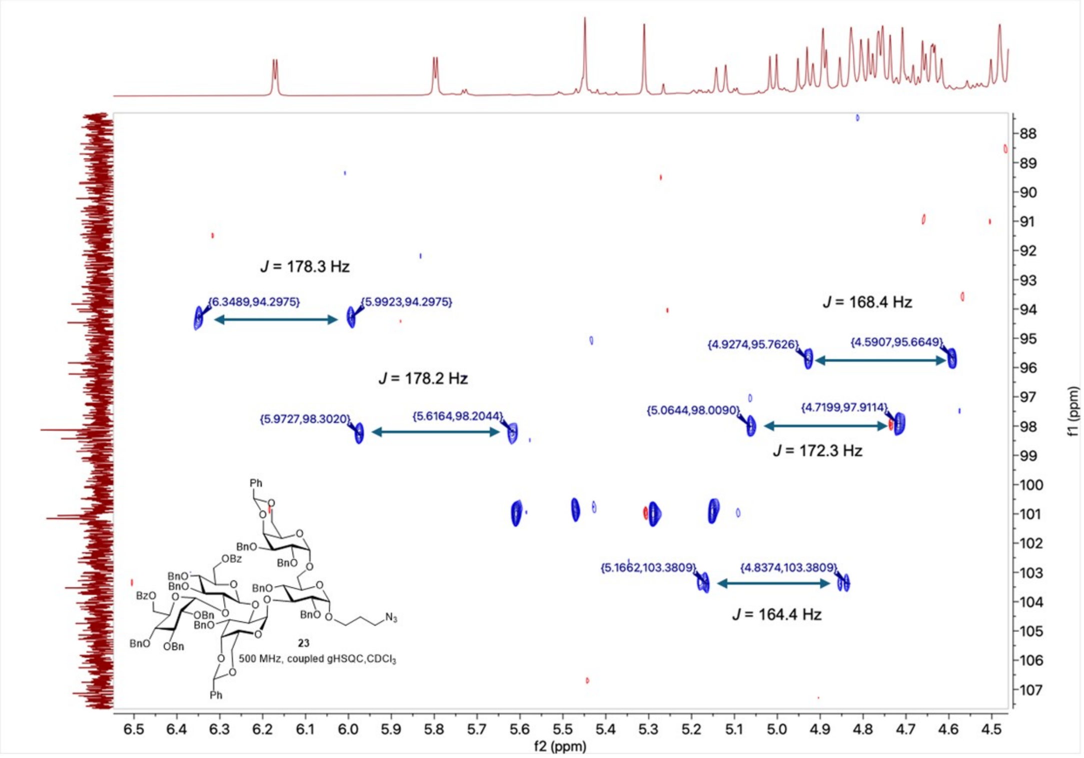
J = 178.3 Hz
{6.3489,94.2975}
{5.9923,94.2975}
J = 168.4 Hz
{4.9274,95.7626}
{4.5907,95.6649}
J = 178.2 Hz
{5.9727,98.3020}
{5.6164,98.2044}
{5.0644,98.0090}
{4.7199,97.9114}
J = 172.3 Hz
{5.1662,103.3809}
{4.8374,103.3809}
J = 164.4 Hz
23
500 MHz, coupled gHSQC,CDCl3
f1 (ppm)
f2 (ppm)

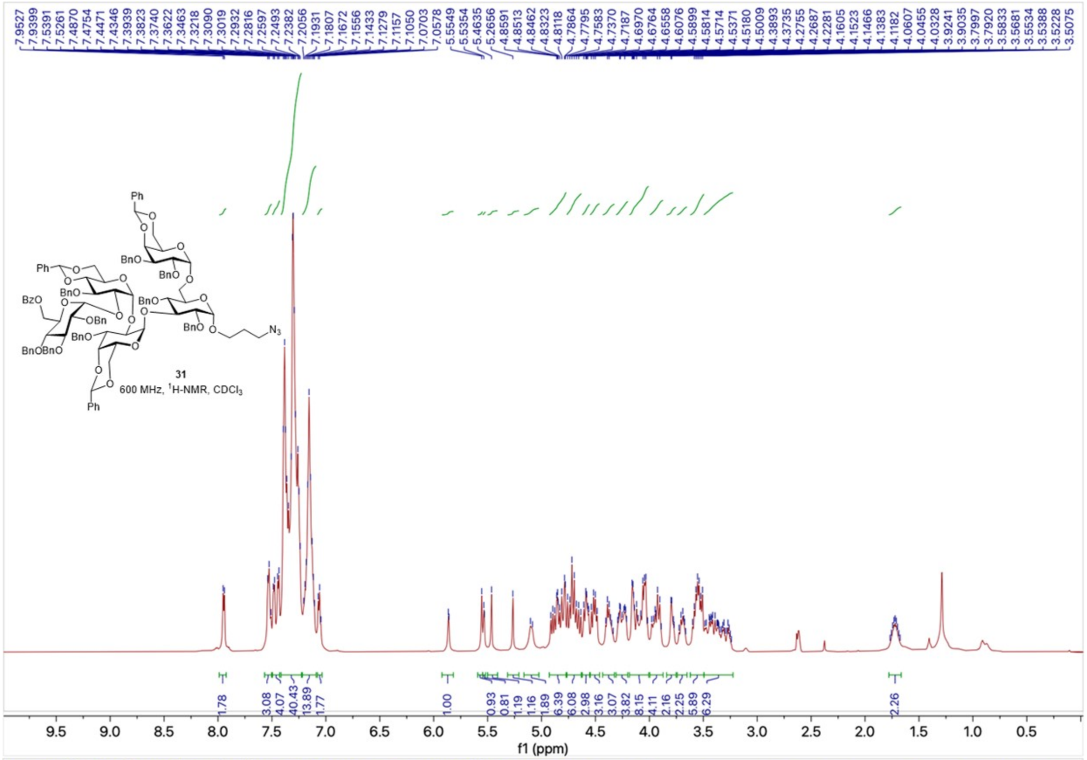

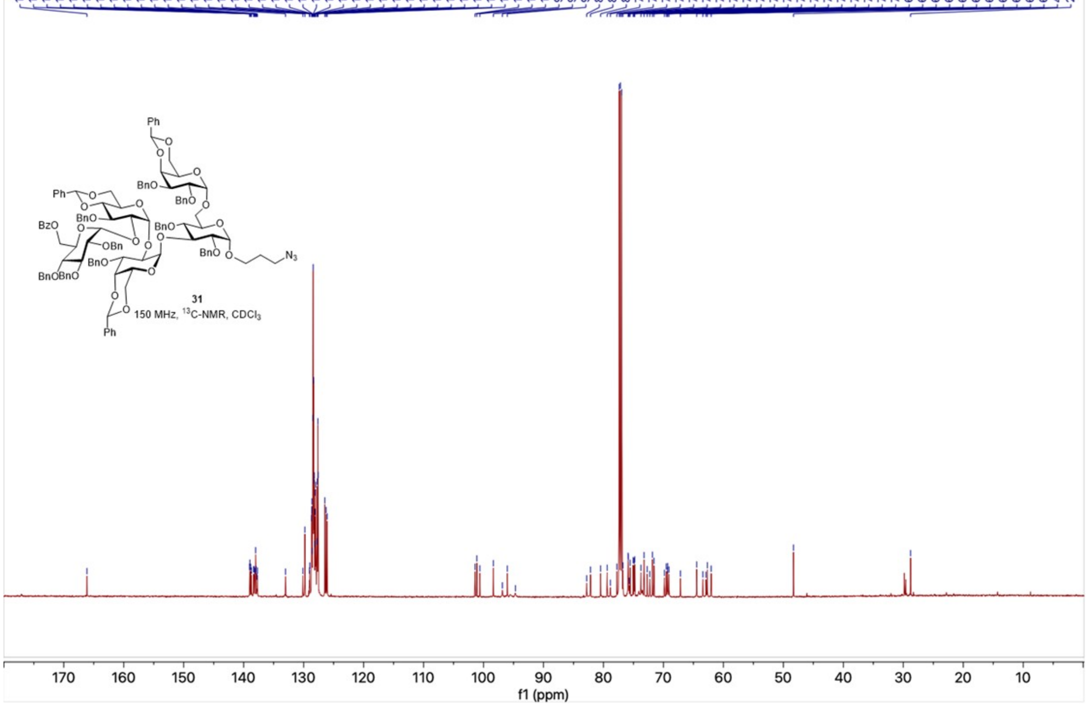

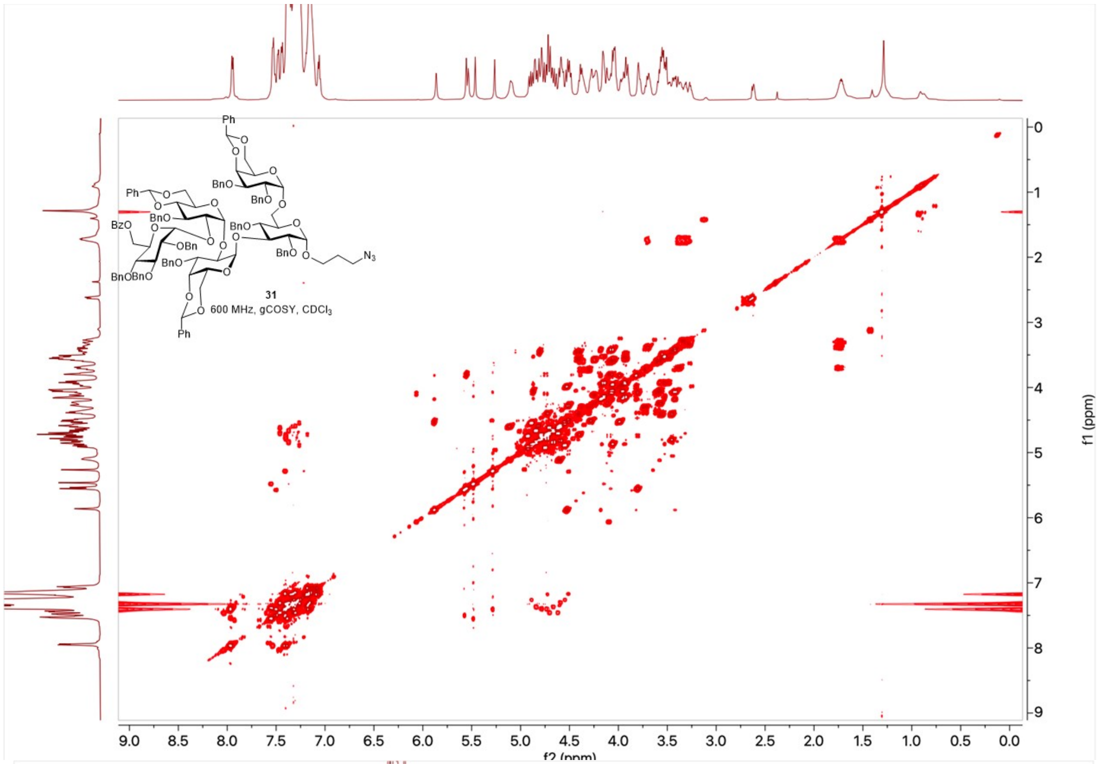
31
600 MHz, gCOSY, CDCl3
f2 (ppm)
f1 (ppm)

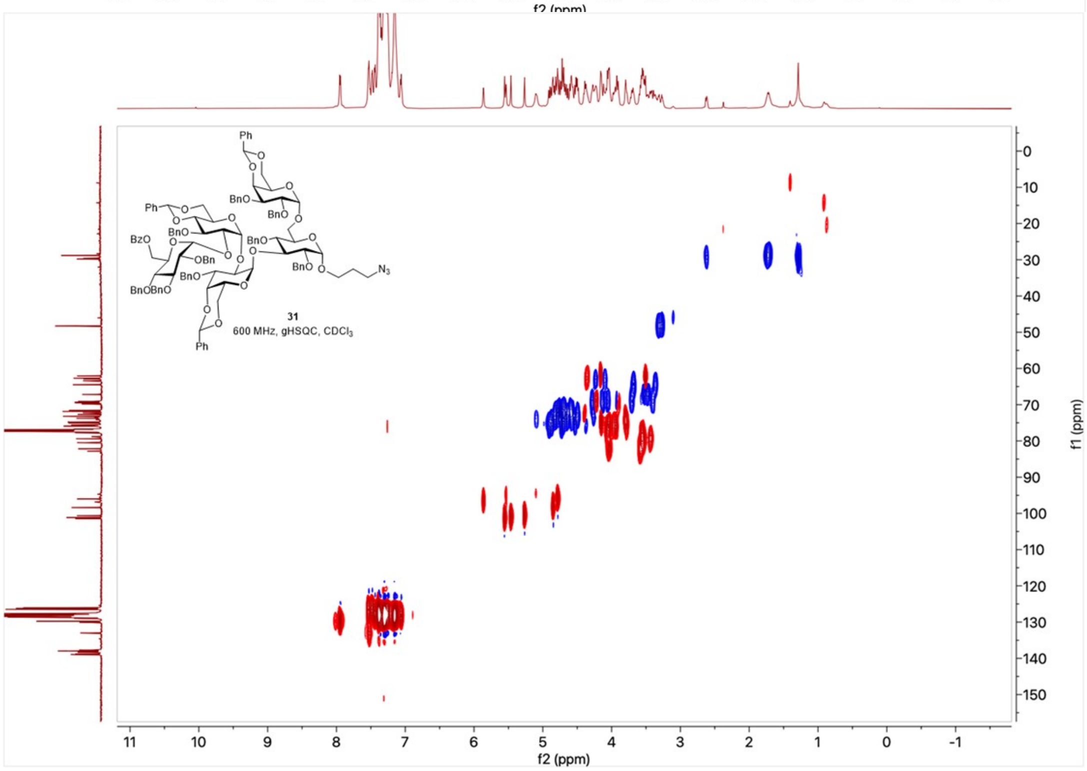
31
600 MHz, gHSQC, CDCl3
f2 (ppm)
f1 (ppm)

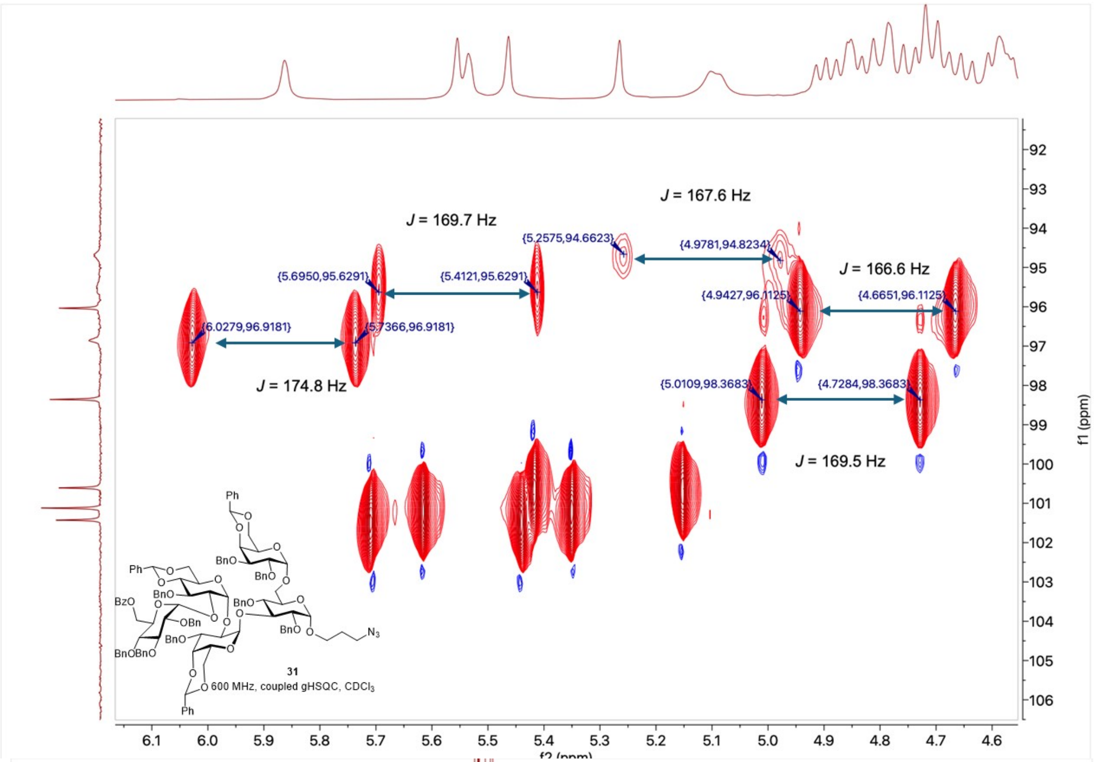
J = 169.7 Hz
J = 167.6 Hz
J = 166.6 Hz
J = 174.8 Hz
J = 169.5 Hz
{6.0279,96.9181}
{5.7366,96.9181}
{5.6950,95.6291}
{5.4121,95.6291}
{5.2575,94.6623}
{4.9781,94.8234}
{4.9427,96.1125}
{4.6651,96.1125}
{5.0109,98.3683}
{4.7284,98.3683}
31
600 MHz, coupled gHSQC, CDCl3
f1 (ppm)
f2 (ppm)

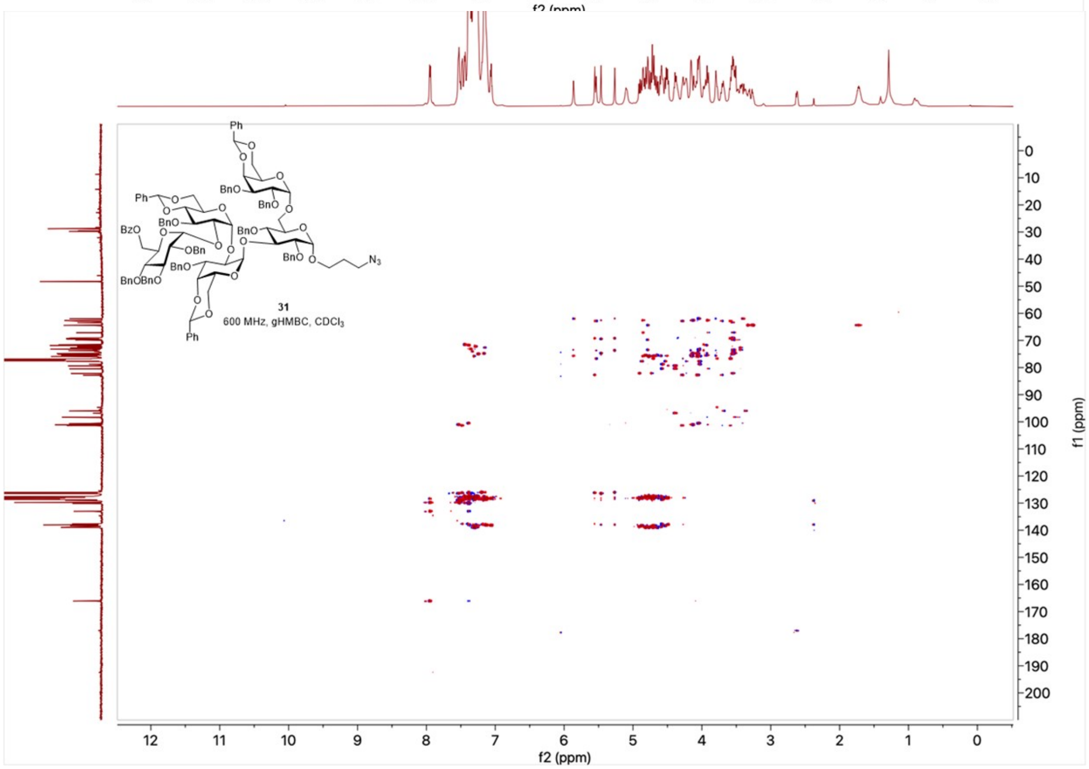
31
600 MHz, gHMBC, CDCl3
f1 (ppm)
f2 (ppm)

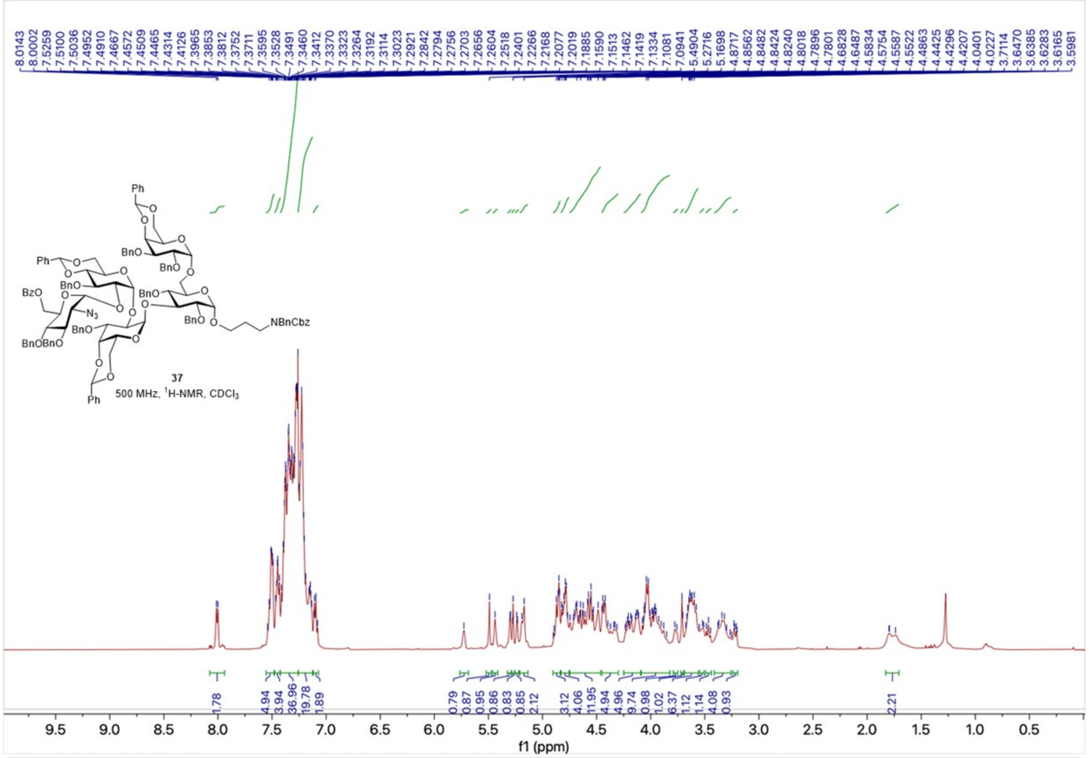

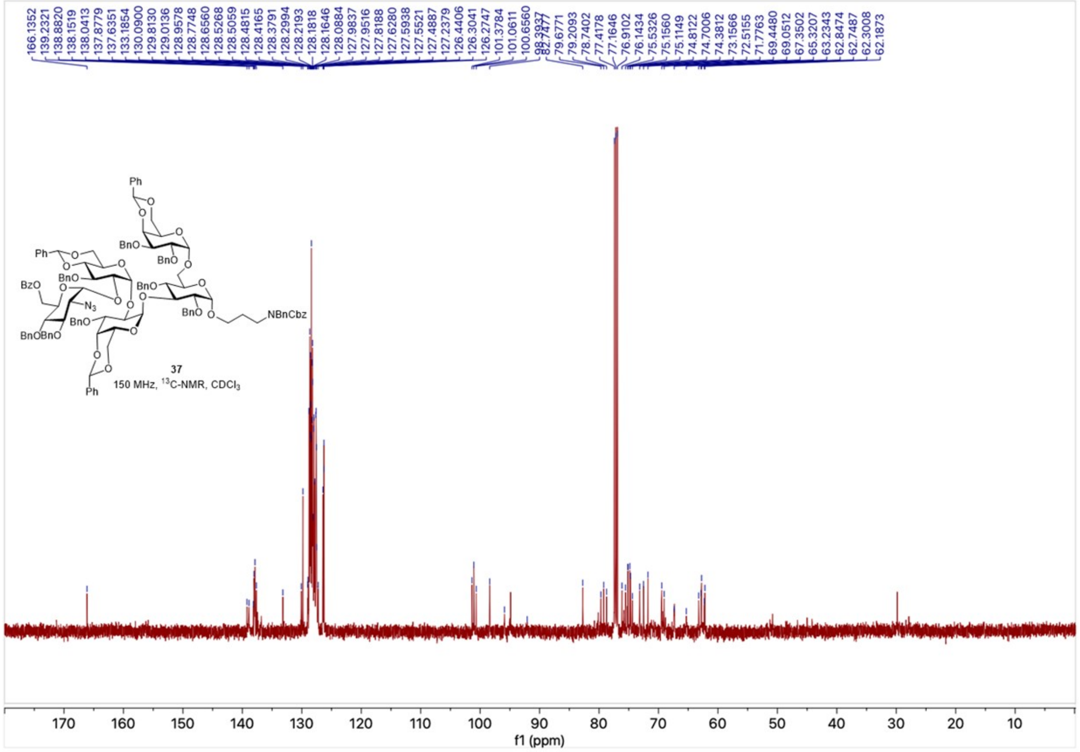

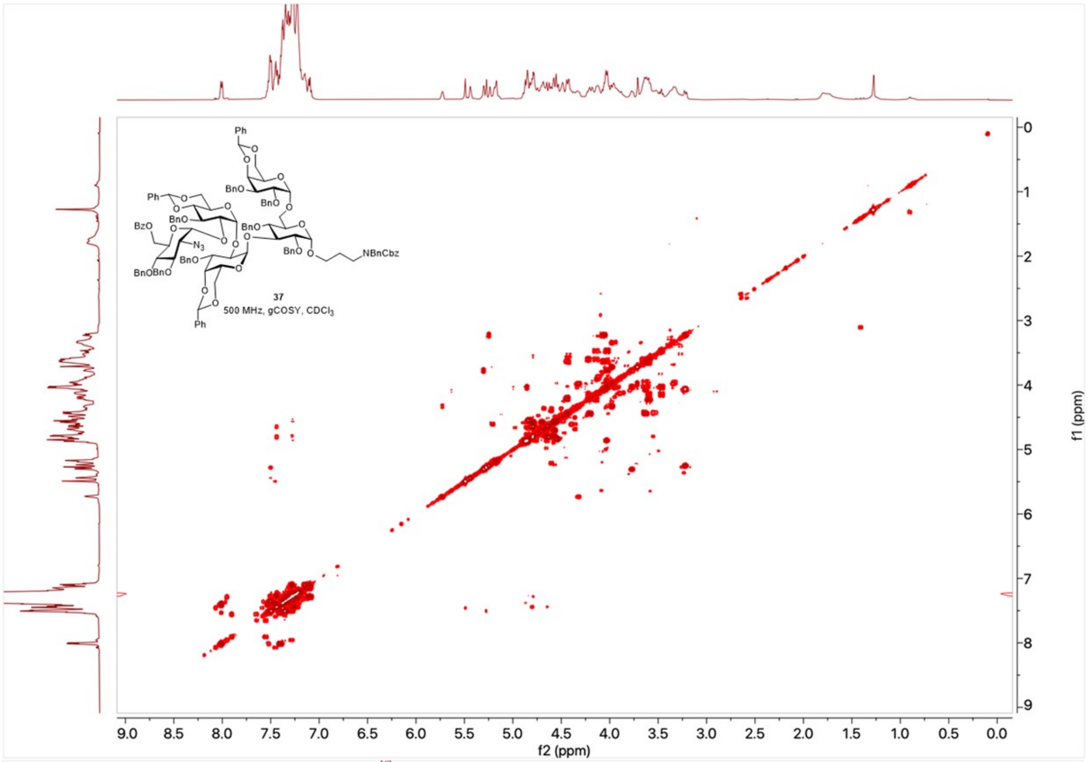
Ph
BnO
BnO
Ph
BzO
BnO
BnO
N3
BnO
BnO
BnO
BnO
BnO
BnOBnO
NBnCbz
Ph
37
500 MHz, gCOSY, CDCl3
f1 (ppm)
f2 (ppm)

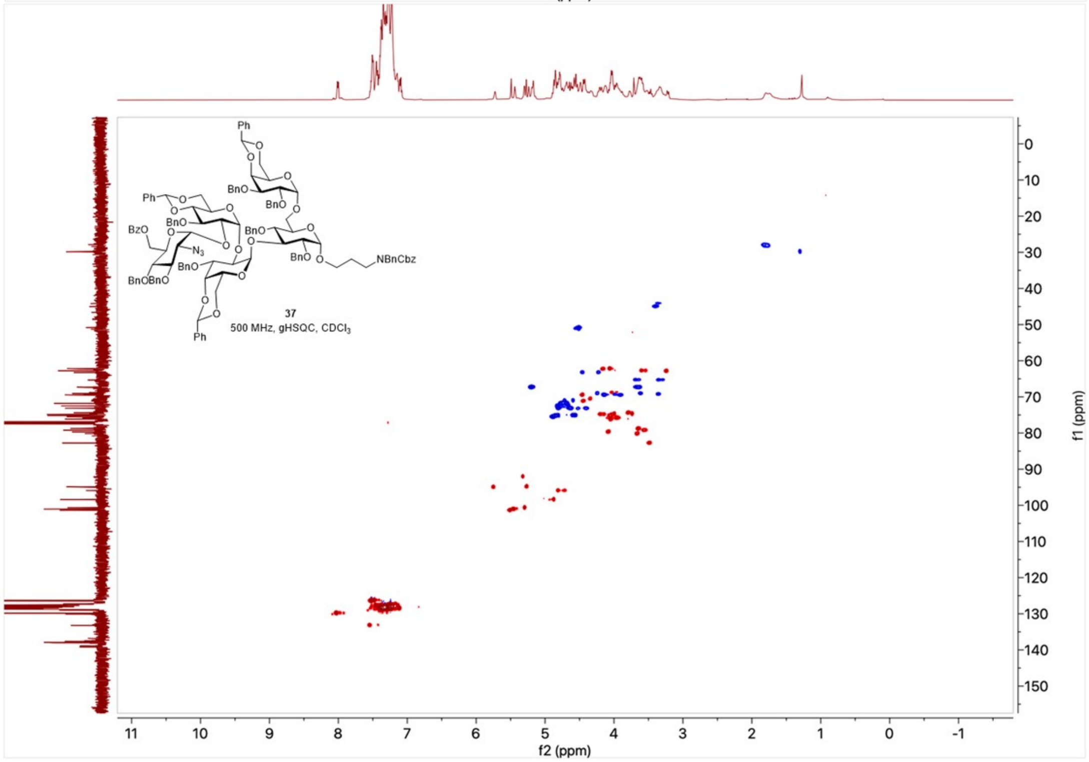
Ph
BnO
BnO
Ph
BzO
BnO
BnO
N3
BnO
BnO
BnO
BnOBnO
NBnCbz
Ph
37
500 MHz, gHSQC, CDCl3
f1 (ppm)
f2 (ppm)

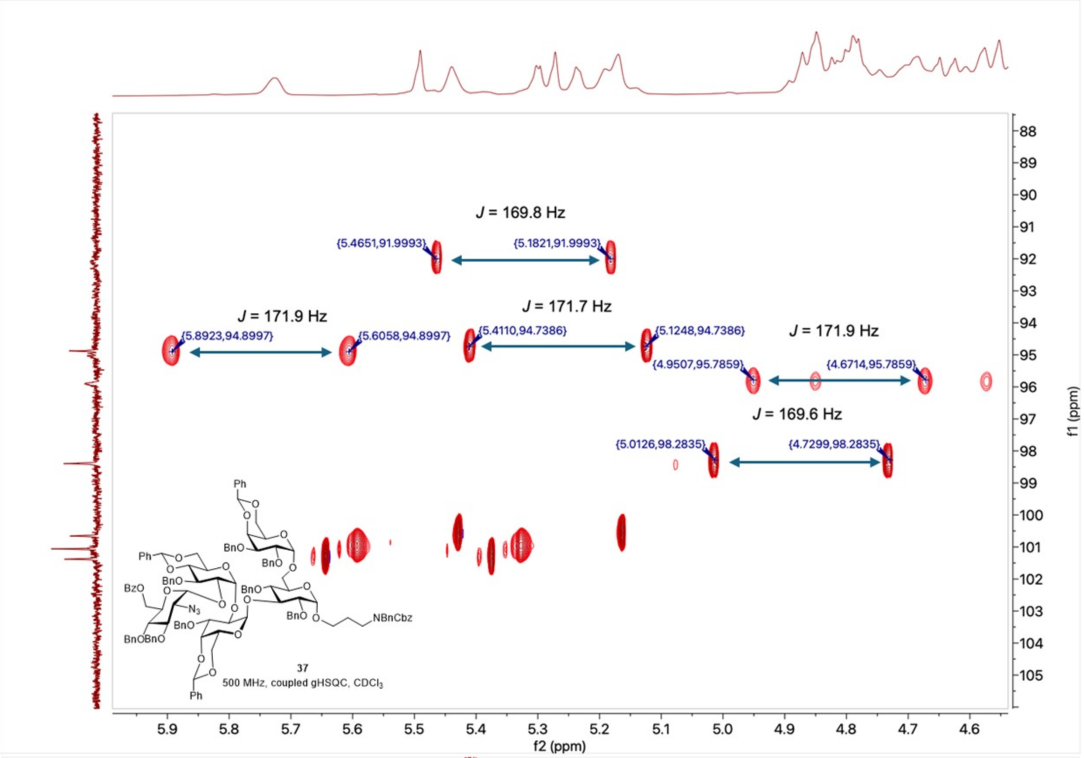
J = 169.8 Hz
{5.4651,91.9993}
{5.1821,91.9993}
J = 171.9 Hz
{5.8923,94.8997}
{5.6058,94.8997}
J = 171.7 Hz
{5.4110,94.7386}
{5.1248,94.7386}
J = 171.9 Hz
{4.9507,95.7859}
{4.6714,95.7859}
J = 169.6 Hz
{5.0126,98.2835}
{4.7299,98.2835}
37
500 MHz, coupled gHSQC, CDCl3
f2 (ppm)
f1 (ppm)

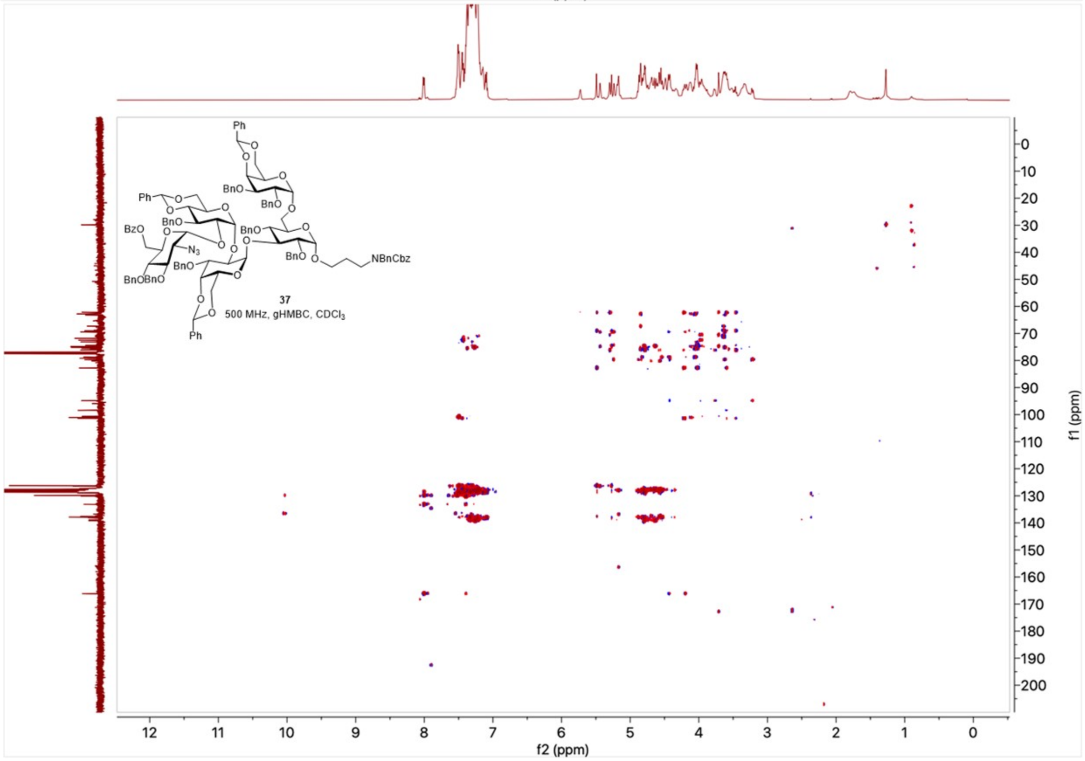
37
500 MHz, gHMBC, CDCl3
f2 (ppm)
f1 (ppm)

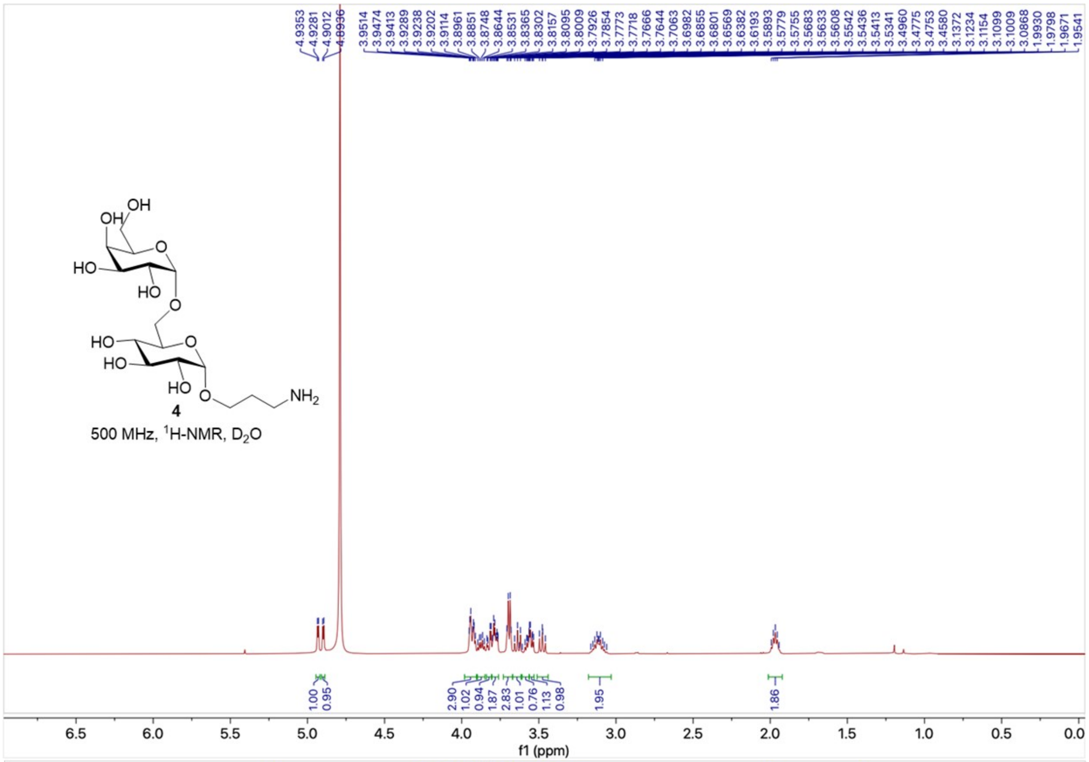
4.9353
4.9281
4.9012
4.8936
3.9514
3.9474
3.9413
3.9289
3.9238
3.9202
3.9114
3.8961
3.8851
3.8748
3.8644
3.8531
3.8365
3.8302
3.8157
3.8095
3.8009
3.7926
3.7854
3.7773
3.7718
3.7666
3.7644
3.7063
3.6982
3.6855
3.6801
3.6569
3.6382
3.6193
3.5893
3.5779
3.5755
3.5683
3.5633
3.5608
3.5542
3.5436
3.5413
3.5341
3.4960
3.4775
3.4753
3.4580
3.1372
3.1234
3.1154
3.1099
3.1009
3.0868
1.9930
1.9798
1.9671
1.9541
OH
OH
O
HO
HO
O
HO
O
HO
HO
O
NH2
4
500 MHz, 1H-NMR, D2O
1.00
0.95
2.90
1.02
0.94
1.87
2.83
1.01
0.76
1.13
0.98
1.95
1.86
6.5
6.0
5.5
5.0
4.5
4.0
3.5
3.0
2.5
2.0
1.5
1.0
0.5
0.0
f1 (ppm)

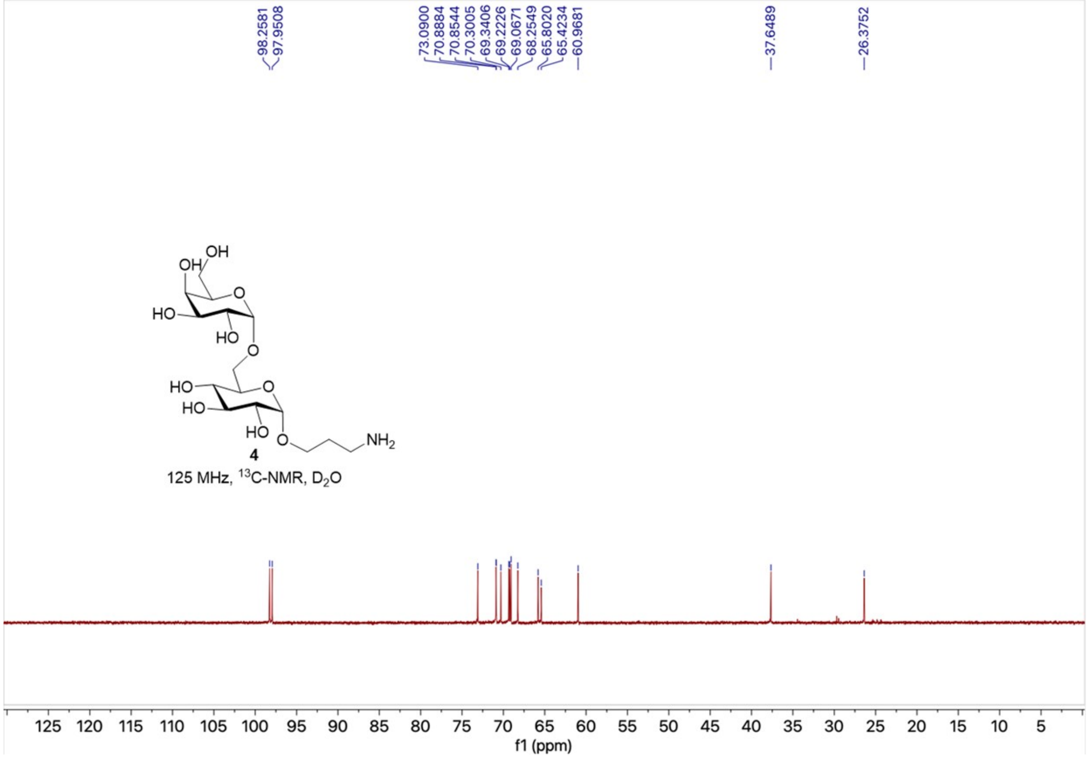
98.2581
97.9508
73.0900
70.8884
70.8544
70.3005
69.3406
69.2226
69.0671
68.2549
65.8020
65.4234
60.9681
37.6489
26.3752
OH
OH
O
HO
HO
O
HO
O
HO
HO
O
NH2
4
125 MHz, 13C-NMR, D2O
125
120
115
110
105
100
95
90
85
80
75
70
65
60
55
50
45
40
35
30
25
20
15
10
5
f1 (ppm)

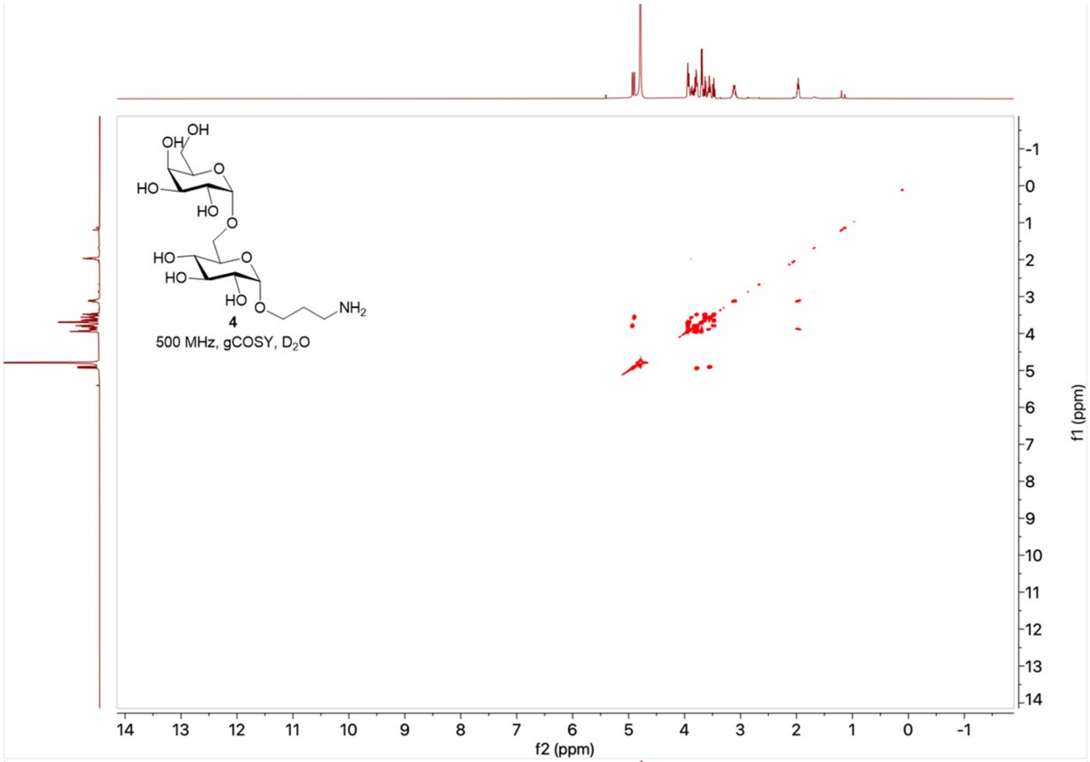

4
500 MHz, gCOSY, D2O
f2 (ppm)
f1 (ppm)

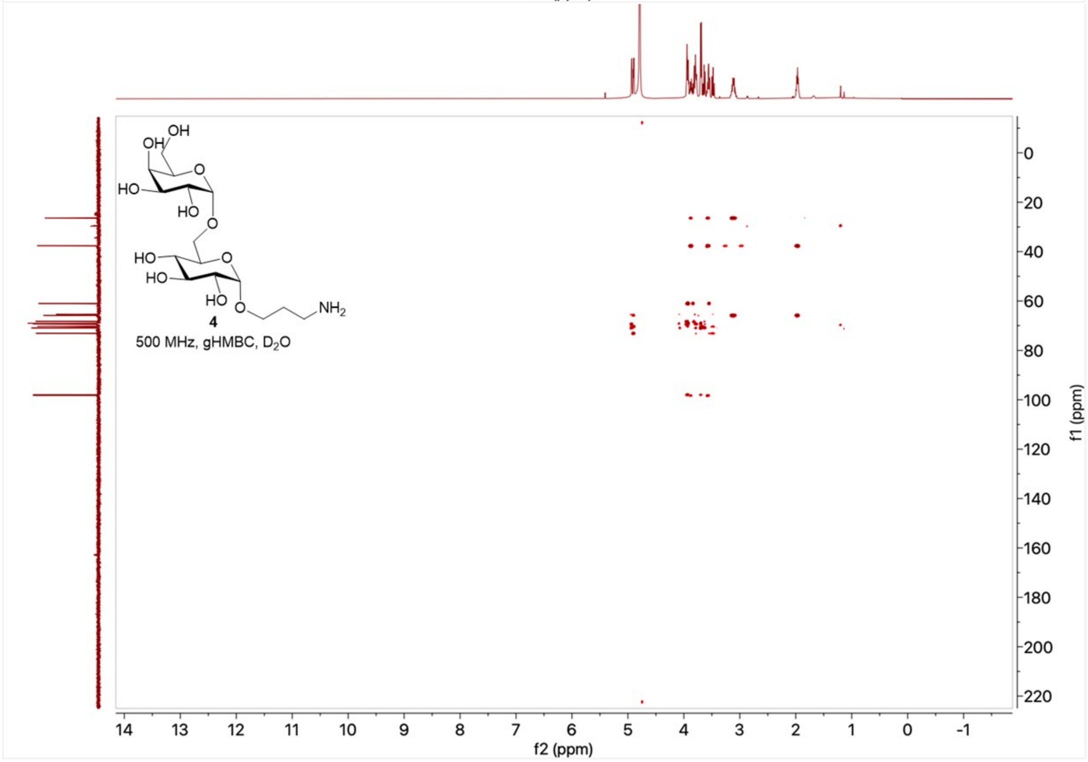

4
500 MHz, gHMBC, D2O
f2 (ppm)
f1 (ppm)

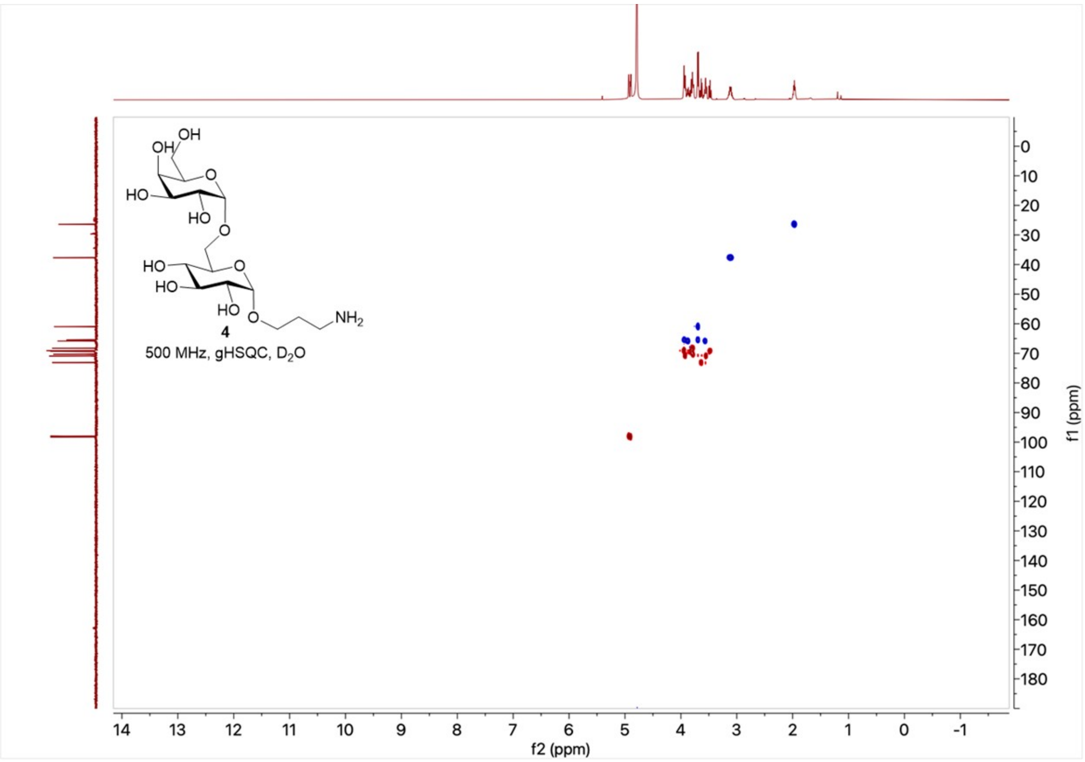

OH
OH
O
HO
HO
O
HO
O
HO
HO
O
NH2
4
500 MHz, gHSQC, D2O
f1 (ppm)
f2 (ppm)

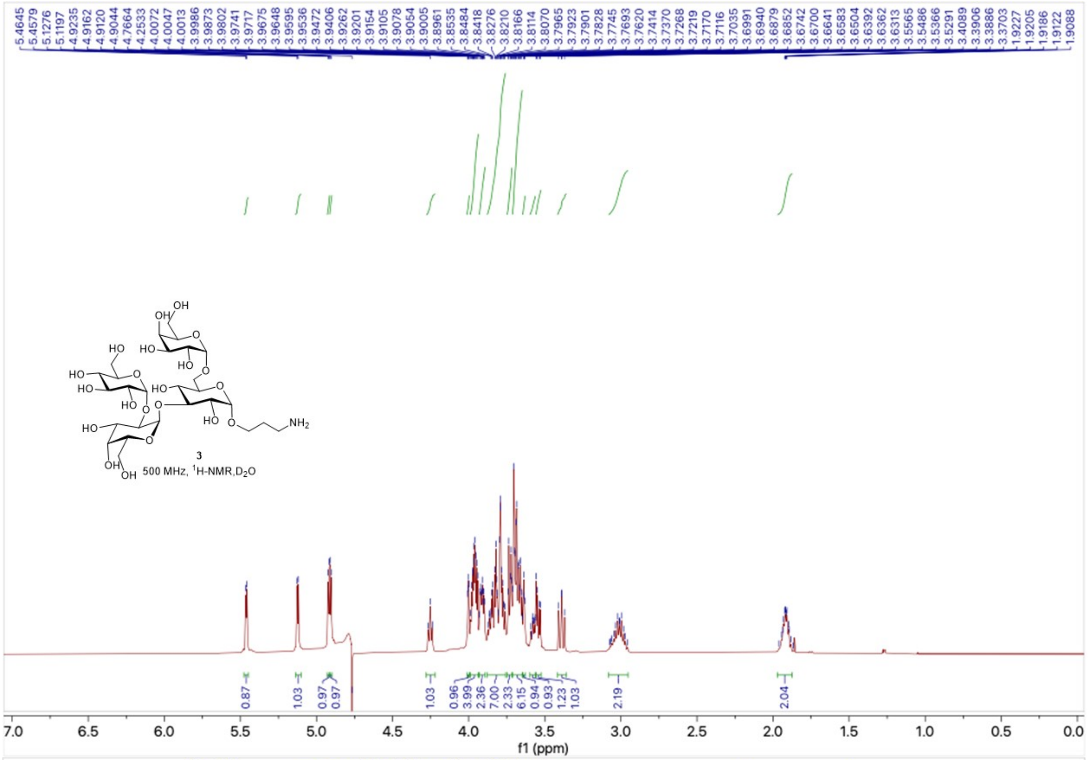
500 MHz, 1H-NMR,D2O
f1 (ppm)

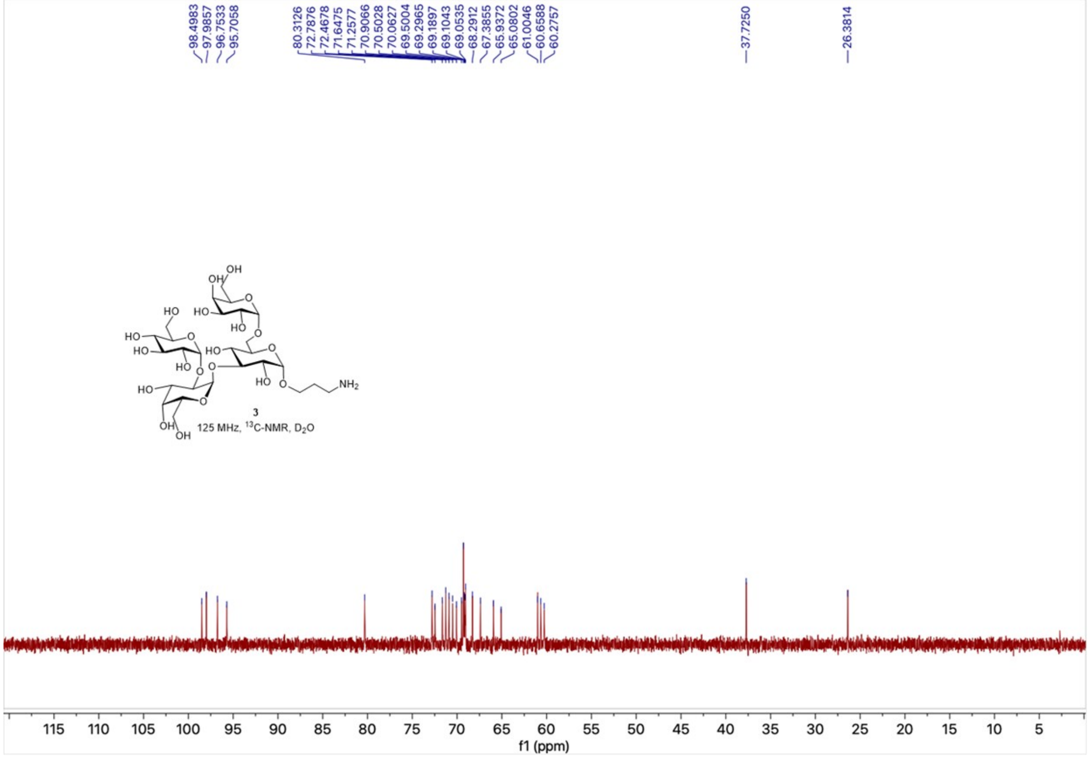
125 MHz, 13C-NMR, D2O
f1 (ppm)

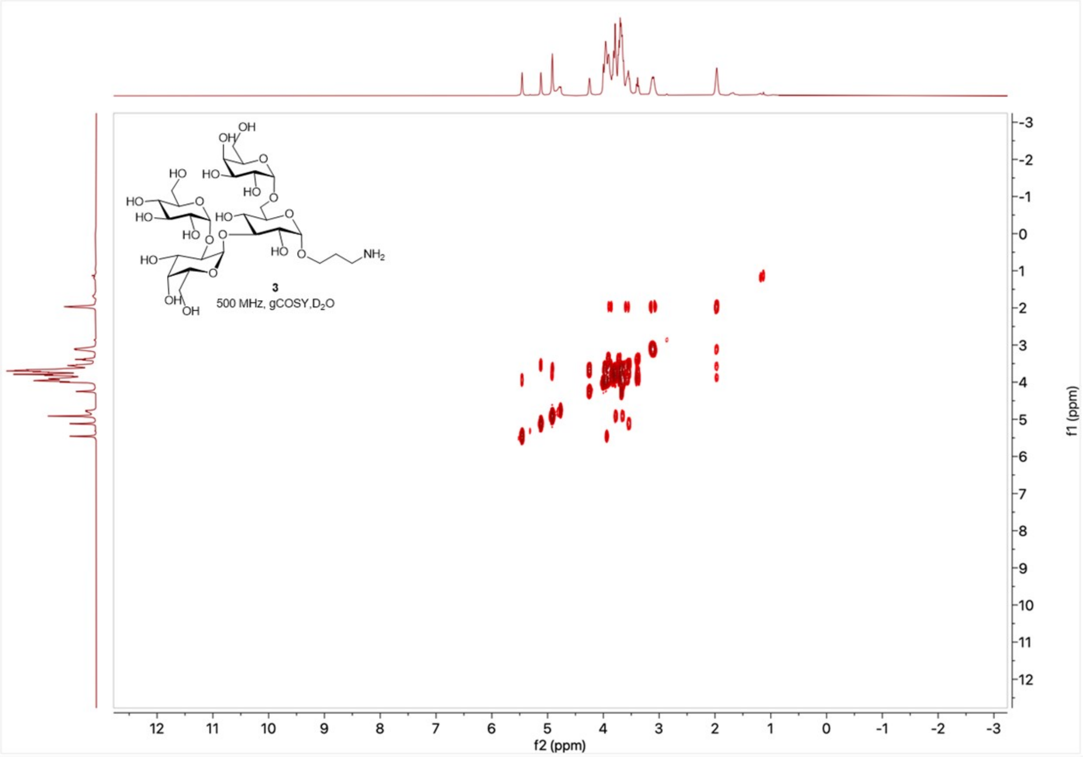
3
500 MHz, gCOSY,D2O
f1 (ppm)
f2 (ppm)

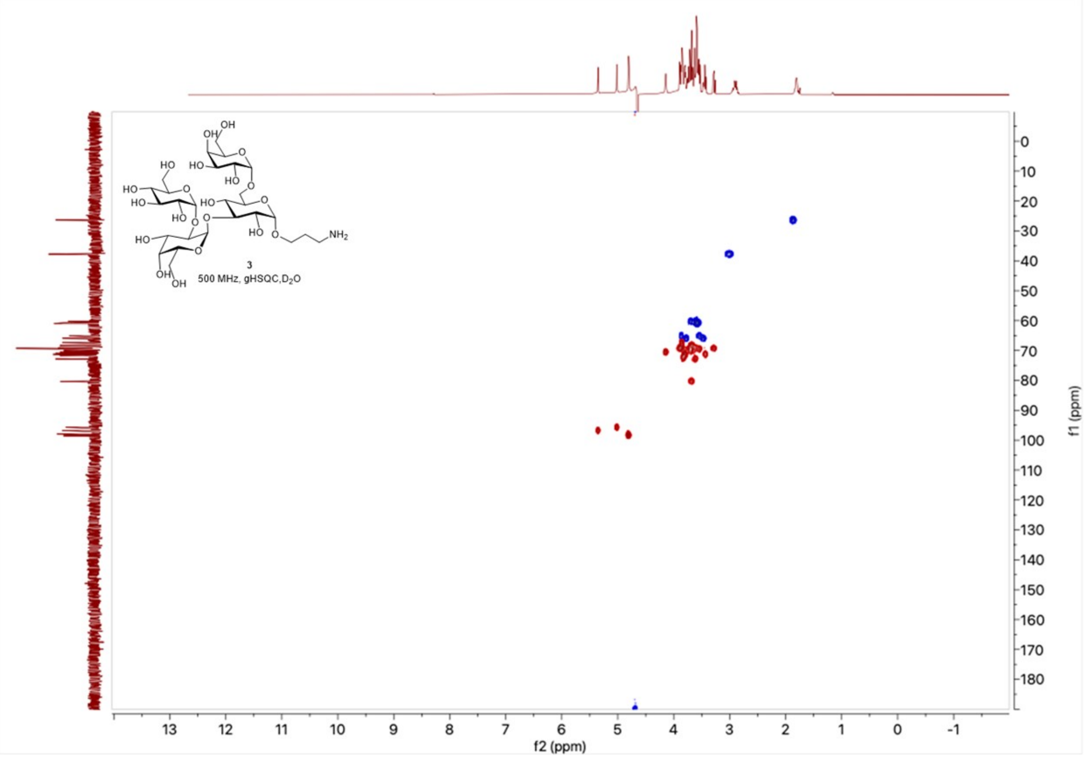
3
500 MHz, gHSQC,D2O
f1 (ppm)
f2 (ppm)

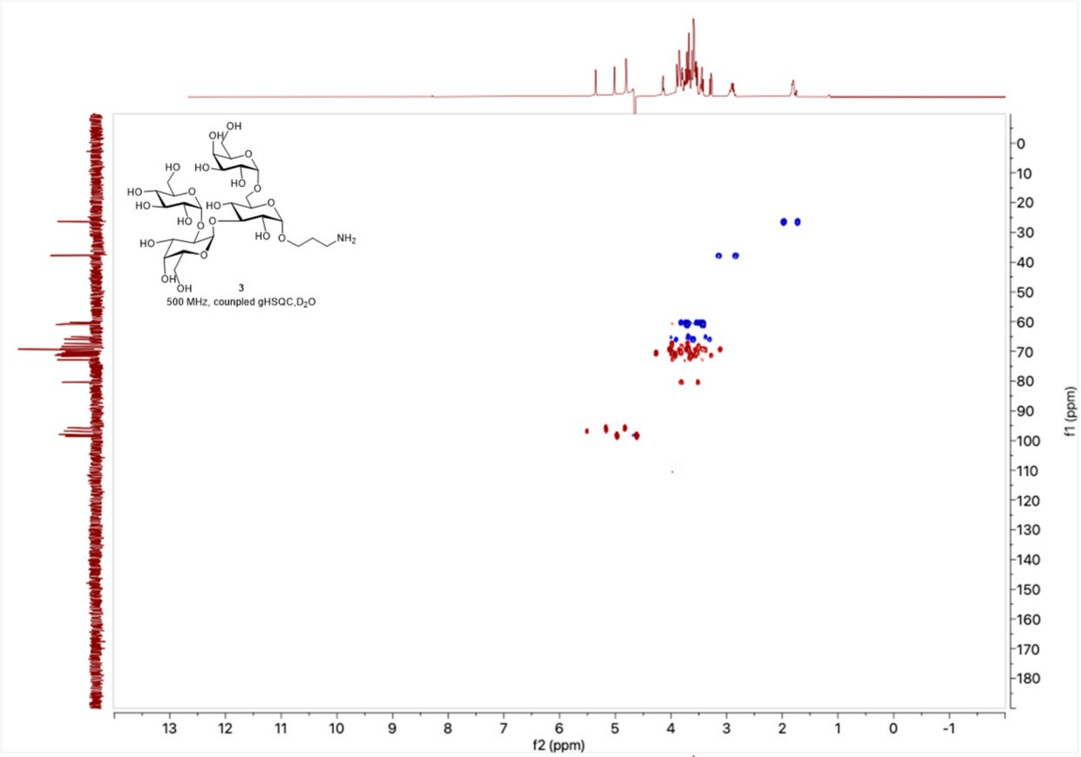
3
500 MHz, counpled gHSQC,D$_2$O
f1 (ppm)
f2 (ppm)

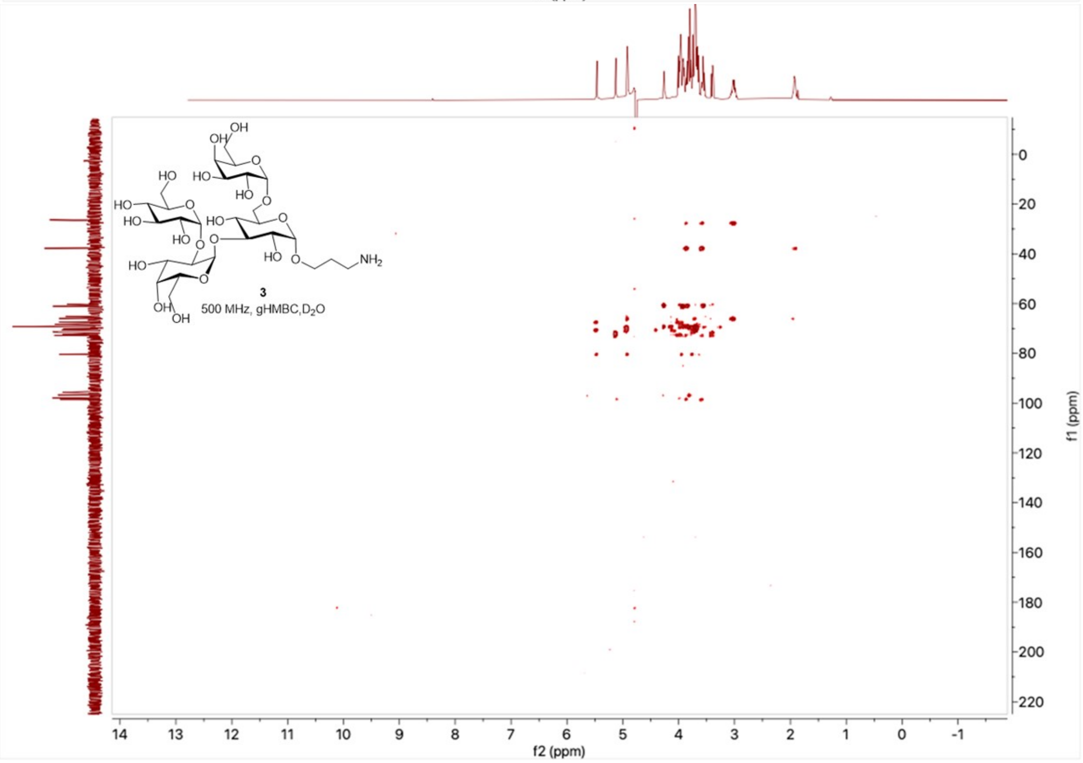
3
500 MHz, gHMBC,D$_2$O
f1 (ppm)
f2 (ppm)

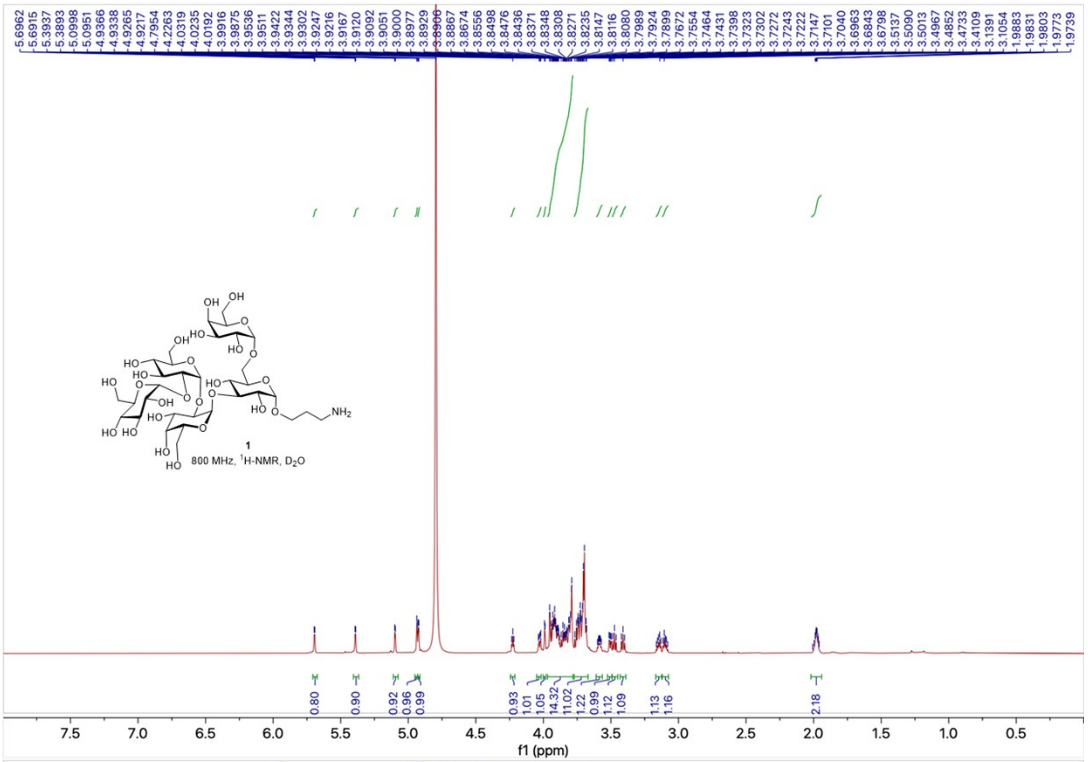
1
800 MHz, ¹H-NMR, D₂O
f1 (ppm)

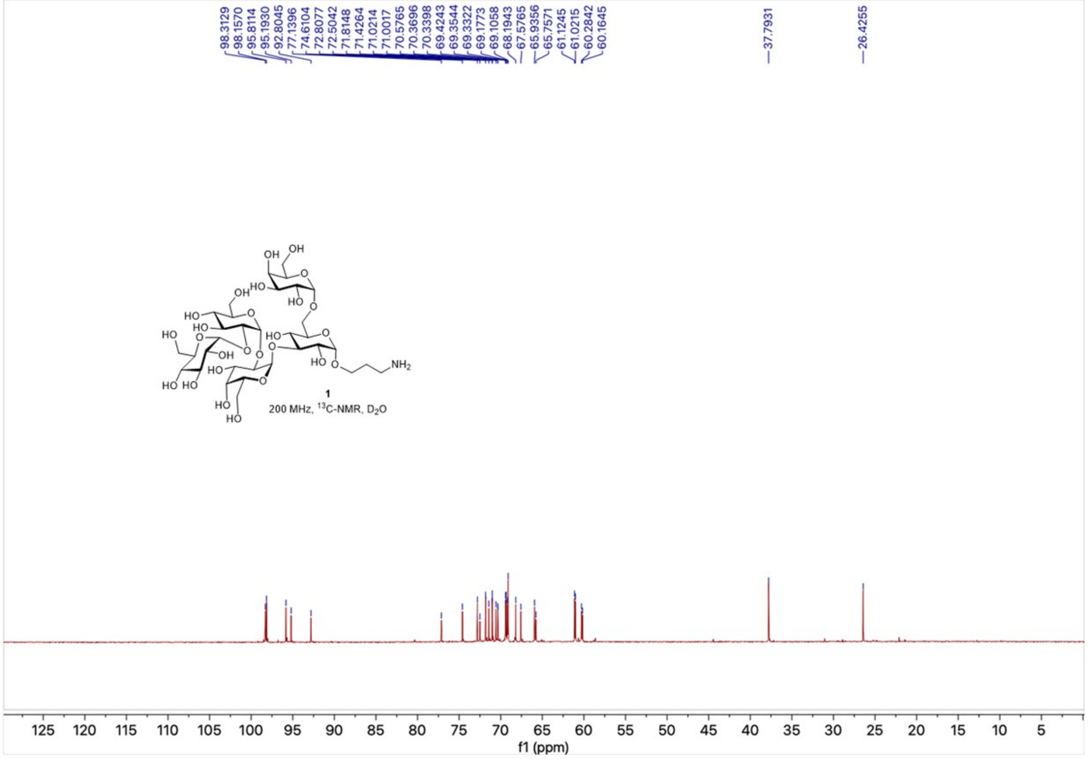
1
200 MHz, ¹³C-NMR, D₂O
f1 (ppm)

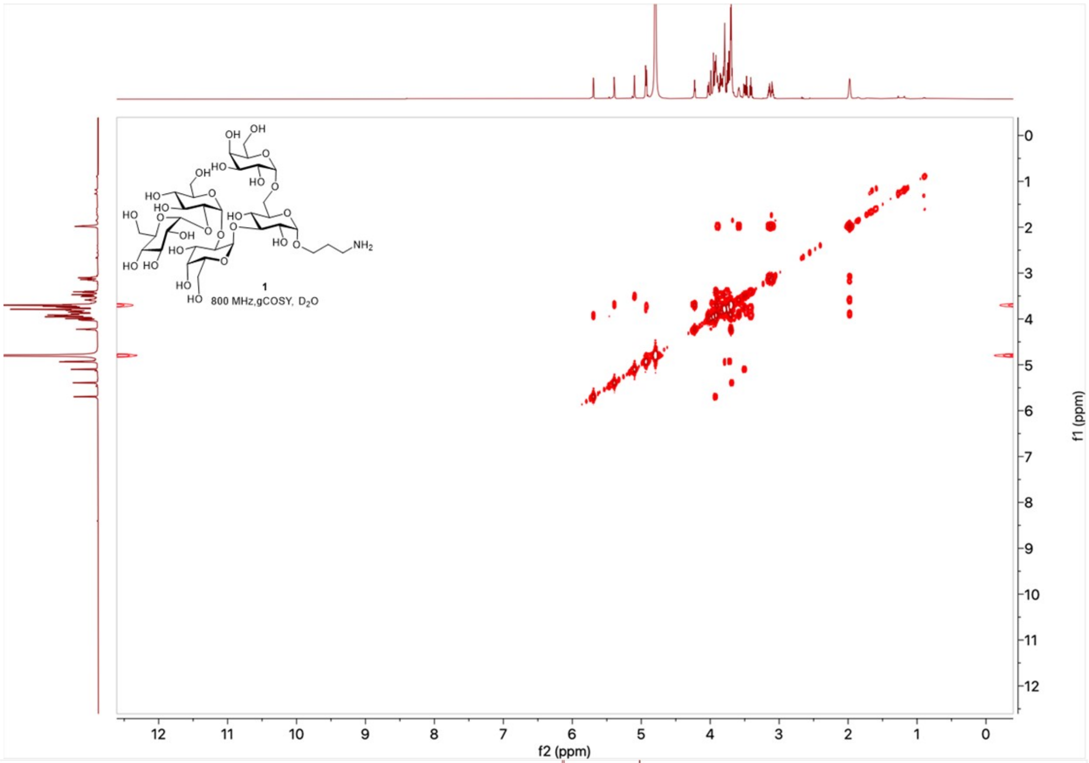

1
800 MHz,gCOSY, D2O
f1 (ppm)
f2 (ppm)

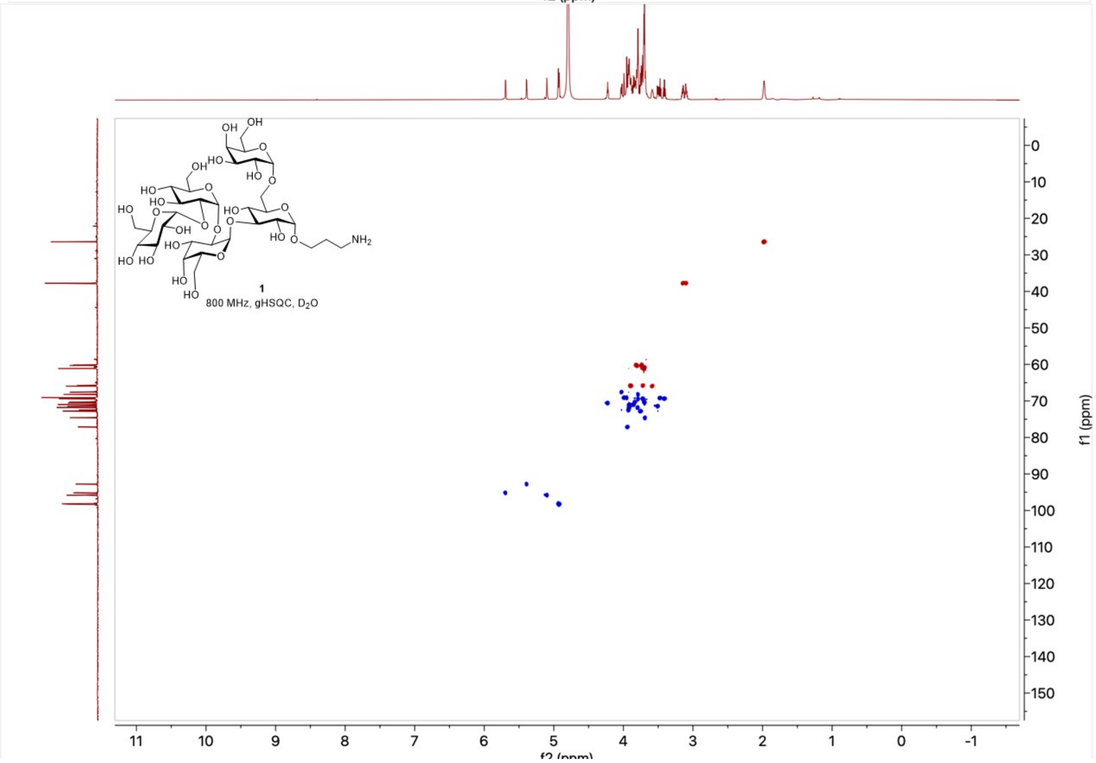

1
800 MHz, gHSQC, D2O
f1 (ppm)

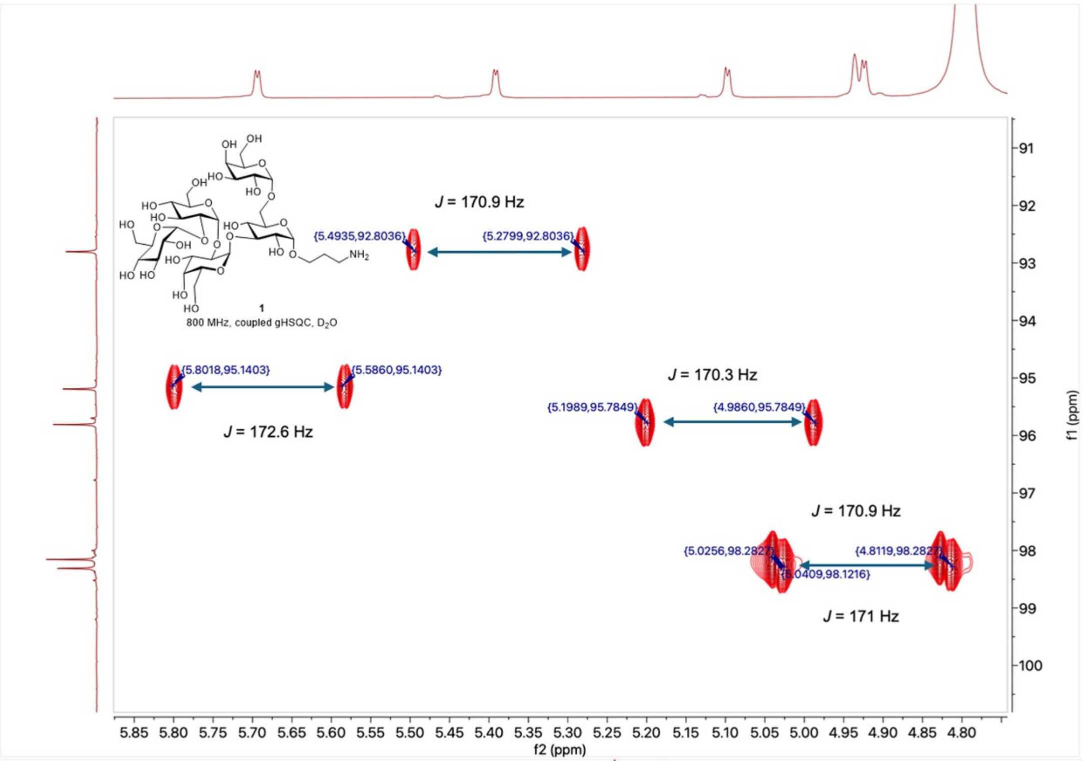

1
800 MHz, coupled gHSQC, D2O
J = 170.9 Hz
{5.4935,92.8036}
{5.2799,92.8036}
{5.8018,95.1403}
{5.5860,95.1403}
J = 172.6 Hz
J = 170.3 Hz
{5.1989,95.7849}
{4.9860,95.7849}
J = 170.9 Hz
{5.0256,98.2827}
{5.0409,98.1216}
{4.8119,98.2827}
J = 171 Hz
f1 (ppm)
f2 (ppm)

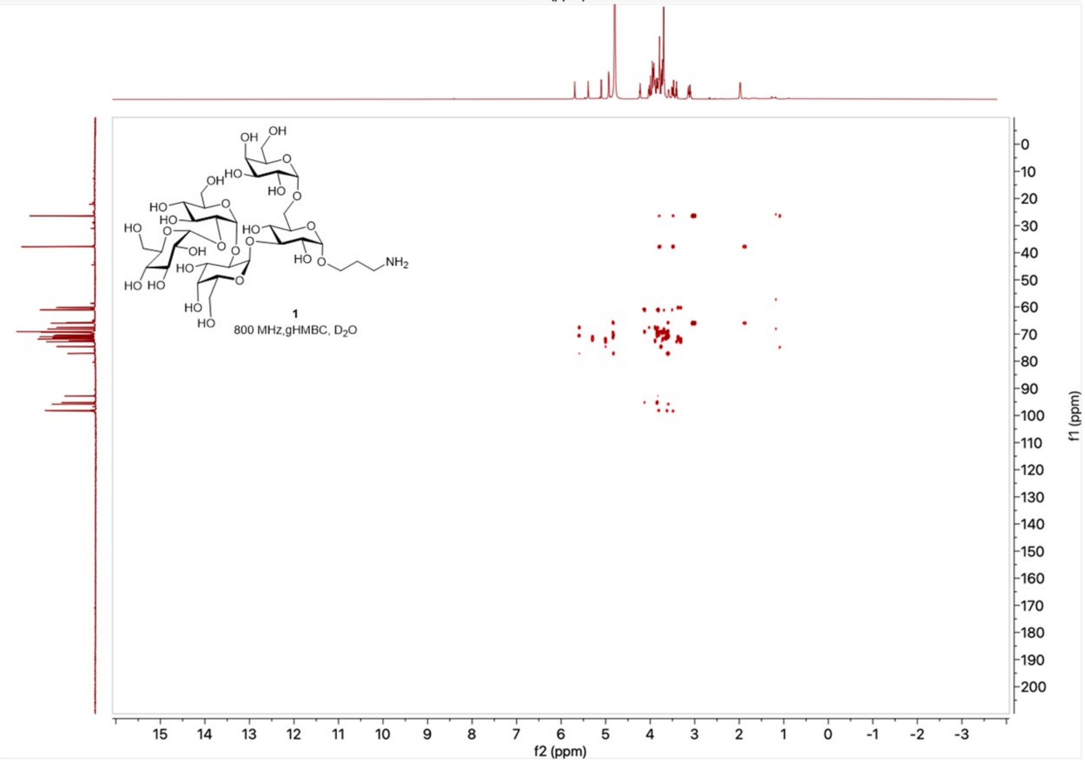

1
800 MHz,gHMBC, D2O
f1 (ppm)
f2 (ppm)

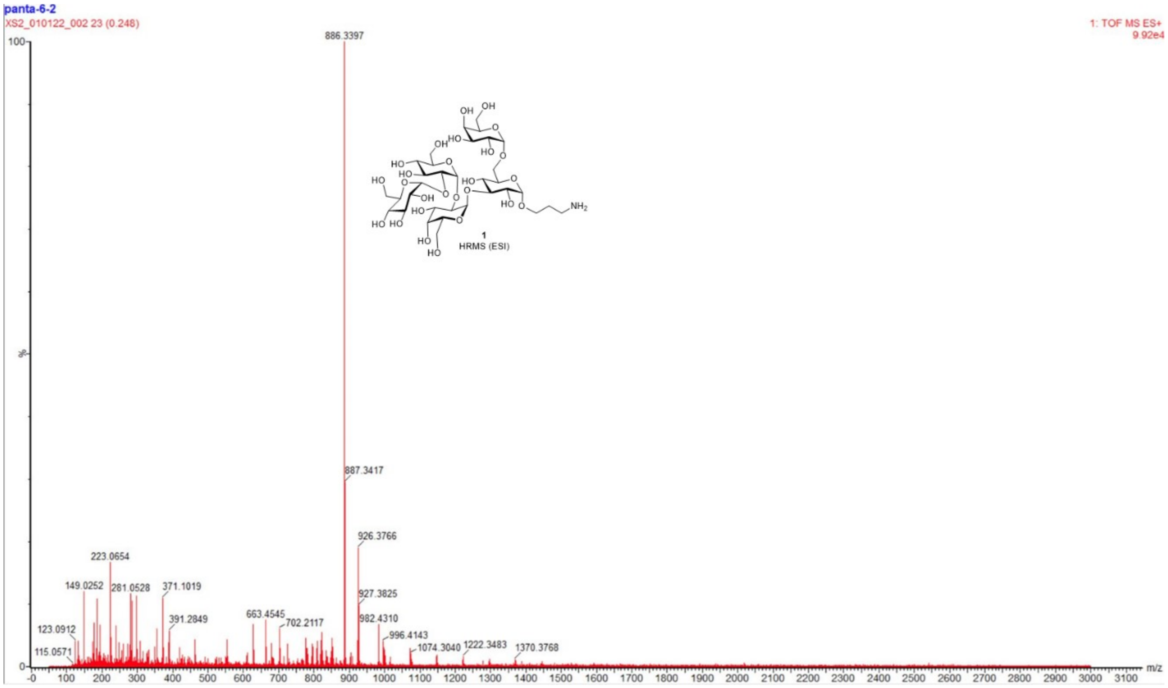
panta-6-2
XS2_010122_002 23 (0.248)
1: TOF MS ES+
9.92e4
886.3397
887.3417
926.3766
927.3825
982.4310
996.4143
1074.3040
1222.3483
1370.3768
223.0654
149.0252
281.0528
371.1019
391.2849
663.4545
702.2117
123.0912
115.0571
1
HRMS (ESI)
m/z
%

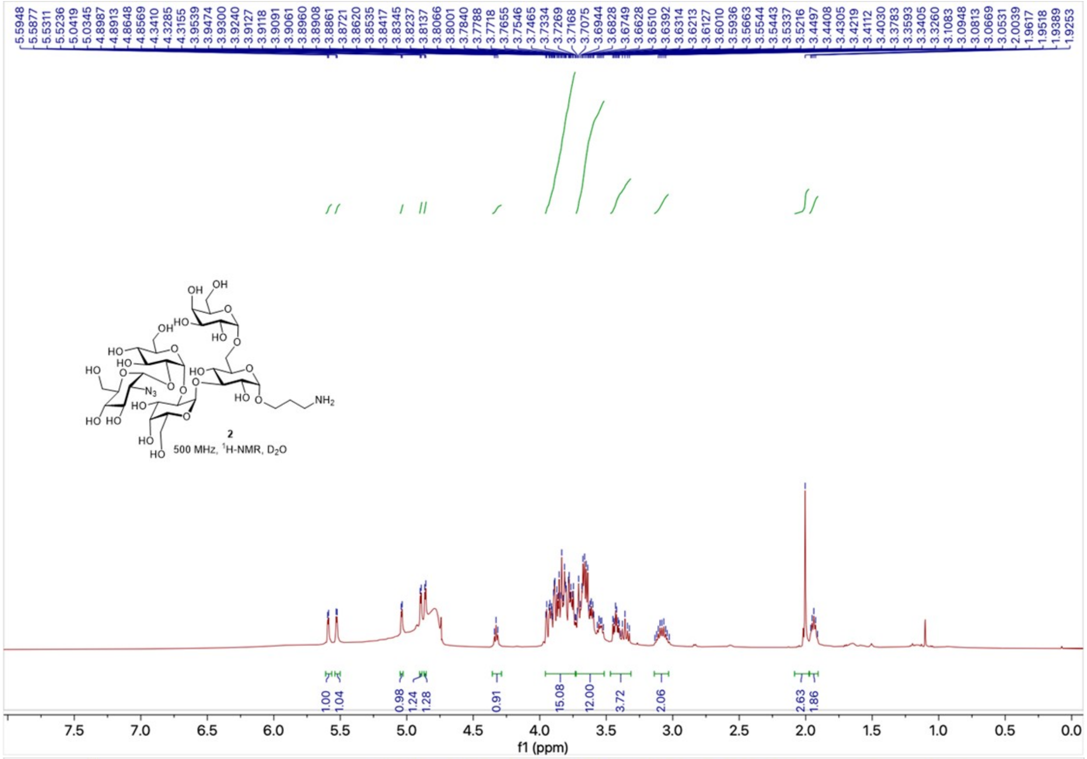

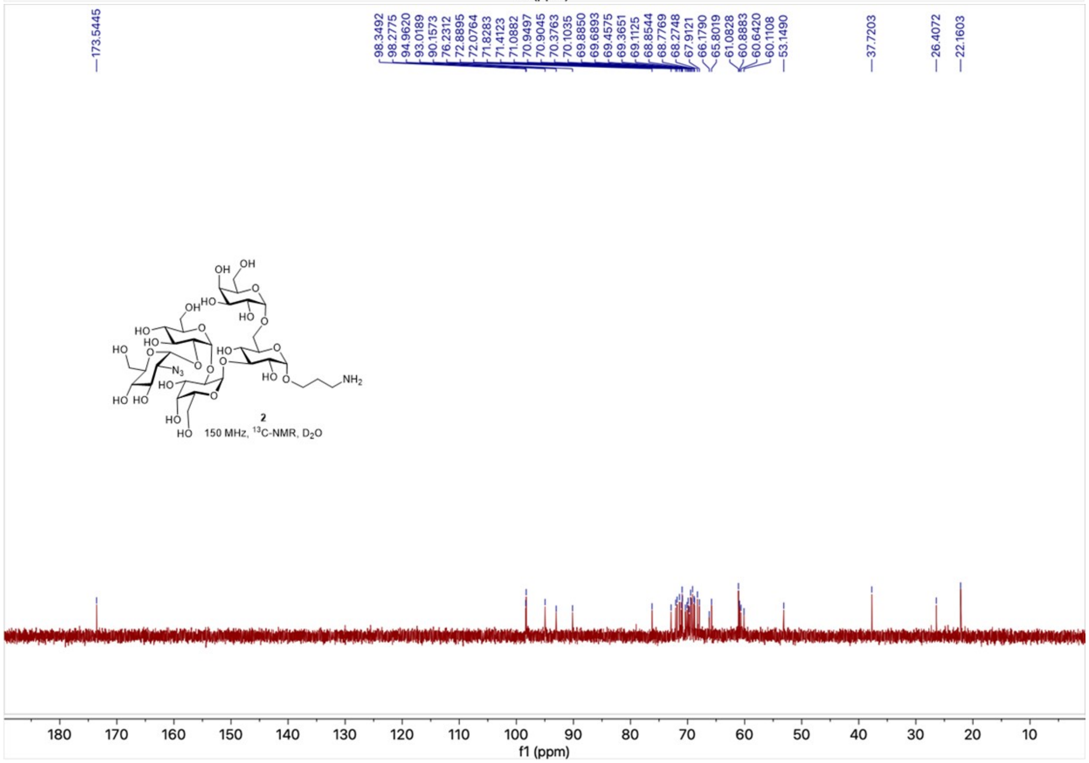

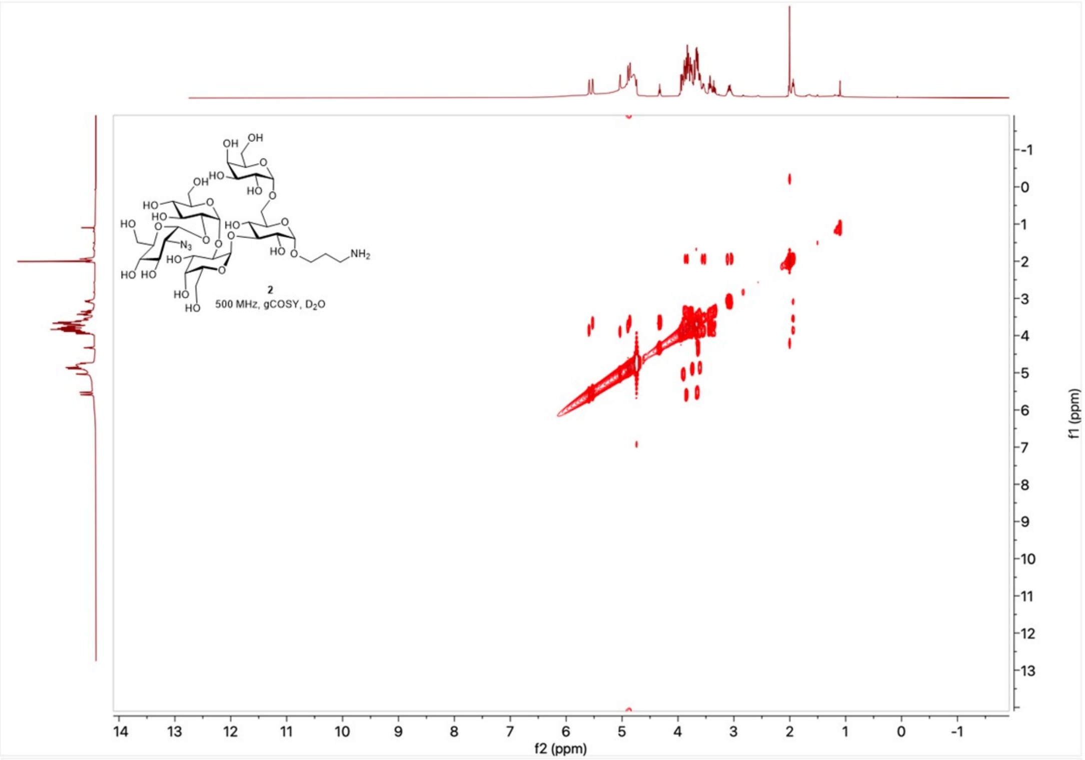
NH2
2
500 MHz, gCOSY, D2O
f2 (ppm)
f1 (ppm)

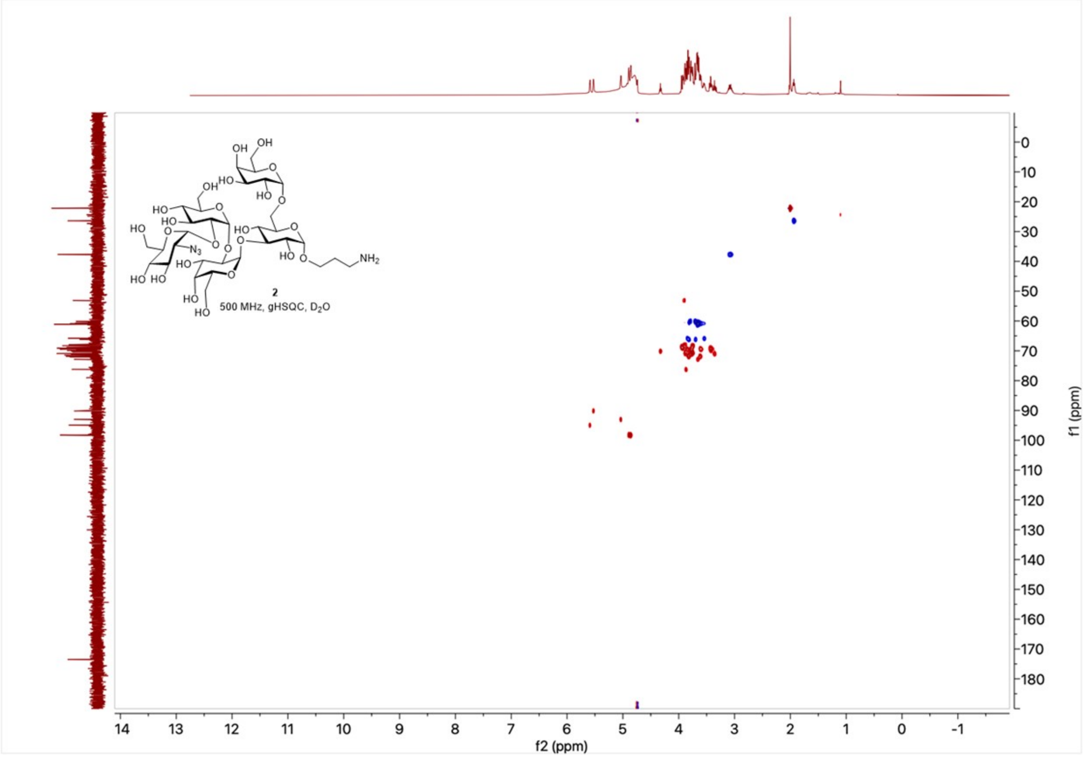
NH2
2
500 MHz, gHSQC, D2O
f2 (ppm)
f1 (ppm)

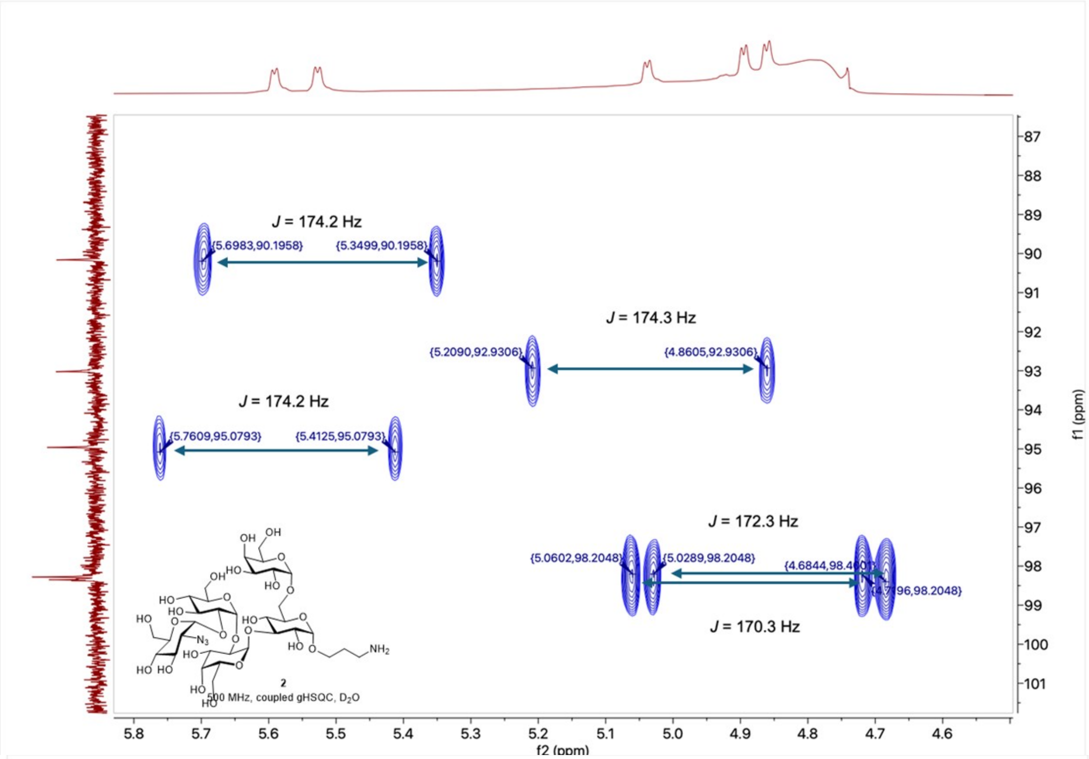

J = 174.2 Hz
{5.6983,90.1958}
{5.3499,90.1958}
J = 174.3 Hz
{5.2090,92.9306}
{4.8605,92.9306}
J = 174.2 Hz
{5.7609,95.0793}
{5.4125,95.0793}
J = 172.3 Hz
{5.0602,98.2048}
{5.0289,98.2048}
J = 170.3 Hz
2
500 MHz, coupled gHSQC, D_2O
f1 (ppm)
f2 (ppm)

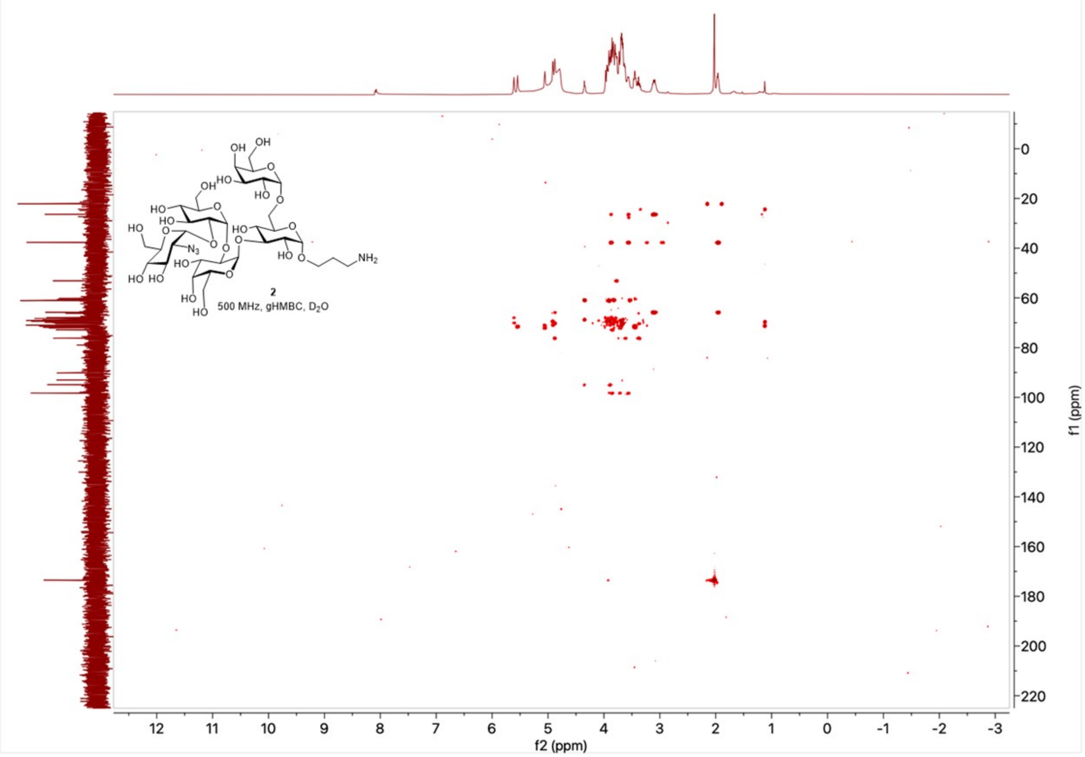

2
500 MHz, gHMBC, D_2O
f1 (ppm)
f2 (ppm)

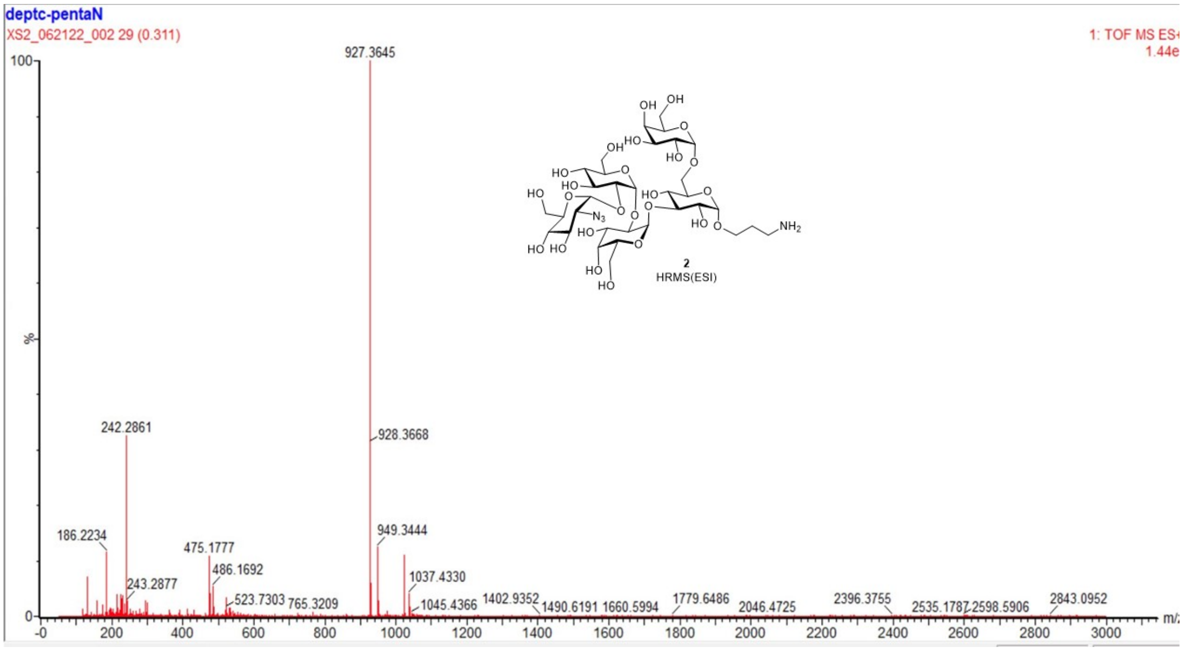

deptc-pentaN
XS2_062122_002 29 (0.311)
1: TOF MS ES+
1.44e
927.3645
928.3668
949.3444
1037.4330
1045.4366
242.2861
186.2234
243.2877
475.1777
486.1692
523.7303
765.3209
1402.9352
1490.6191
1660.5994
1779.6486
2046.4725
2396.3755
2535.1787
2598.5906
2843.0952
100
%
0
m/z
2
HRMS(ESI)
